# Total Synthesis of (+)-Rubriflordilactone A

**DOI:** 10.1002/anie.201506366

**Published:** 2015-09-01

**Authors:** Shermin S Goh, Guilhem Chaubet, Birgit Gockel, Marie-Caroline A Cordonnier, Hannah Baars, Andrew W Phillips, Edward A Anderson

**Affiliations:** Chemistry Research Laboratory, University of Oxford 12 Mansfield Road, Oxford, OX1 3TA (UK) E-mail: edward.anderson@chem.ox.ac.uk; Institute of Organic Chemistry, RWTH Aachen University Landoltweg 1, 52074 Aachen (Germany)

**Keywords:** cyclotrimerization, domino reactions, natural products, total synthesis, transition metal catalysis

## Abstract

Two enantioselective total syntheses of the nortriterpenoid natural product rubriflordilactone A are described, which use palladium- or cobalt-catalyzed cyclizations to form the CDE rings, and converge on a late-stage synthetic intermediate. These key processes are set up through the convergent coupling of a common diyne component with appropriate AB-ring aldehydes, a strategy that sets the stage for the synthetic exploration of other members of this family of natural products.

Chinese herbal plants of the *Schisandra* and *Kadsura* genera have afforded a rich diversity of structurally related nortriterpenoid natural products, which are characterized by complex fused ring systems, a high degree of oxygenation, and densely arrayed stereochemistry.[1] Many of these have been found to exhibit bioactive properties, including promising levels of anti-HIV activity. Their attractive architectures also represent a formidable synthetic challenge, first met in 2011 by Yang and co-workers in their synthesis of schindilactone A.[2] This landmark achievement has recently been complemented by an elegant asymmetric synthesis of rubriflordilactone A (**1**, Scheme 1) by Li et al., where a 6π-electrocyclization was used to assemble the challenging pentasubstituted D-ring arene;[3] and syntheses of the related family members schilancitrilactones B and C, and propindilactone G.[4] Herein, we describe two convergent enantioselective total syntheses of rubriflordilactone A,[5] which are distinct from previous work in that the CDE ring system at the heart of the natural product framework is formed in a single tricyclization step.[6, 7] The two syntheses differ in the method used to construct this CDE framework, which is achieved under either palladium or cobalt catalysis; the products of these key cyclizations converge on a common late-stage intermediate.

**Scheme 1:**
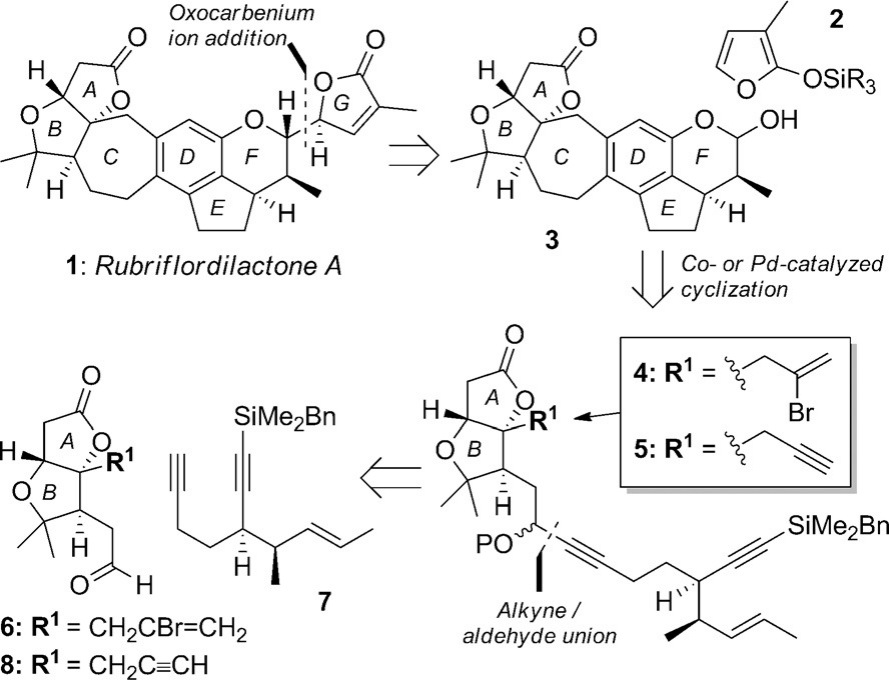
Retrosynthetic analysis of rubriflordilactone A.

Both strategies (Scheme 1) envisaged introduction of the butenolide G ring through addition of a siloxyfuran nucleophile **2** to an oxocarbenium ion in the final step. The latter synthon would arise from lactol **3**, which in turn would be constructed from a bromoendiyne (**4**) or triyne (**5**) under palladium or cobalt catalysis, respectively. Bromoendiyne **4** could be formed from the union of aldehyde **6**[6a] with diyne **7**, the latter of which features two challenging contiguous stereocenters. The synthesis of triyne **5** would also require **7** and the alkyne-bearing aldehyde **8**. These two strategies present different challenges: whilst palladium-catalyzed bromoendiyne cyclizations are established as efficient methods for tricycle synthesis,[8, 9] no applications of this method to natural product total synthesis have been reported; and whilst cobalt-catalyzed alkyne cyclotrimerization has a rich synthetic history,[10] its use in the formation of seven-membered rings (as in the C ring of rubriflordilactone A) is rare.

The synthesis of diyne **7** began with esterification of (*S*,*E*)-pent-3-en-2-ol[11] with carboxylic acid **9** (Scheme 2).[12] Ireland–Claisen rearrangement of the resultant ester **10** led to acid **11**, a reaction that gave higher yield and diastereoselectivity with the free lithium enolate (96 %, >20:1 d.r.)[13] than with the silyl ketene acetal (92 %, 9:1 d.r.).[12] Sequential manipulations to convert the carboxylic acid in **11** into benzyldimethylsilyl alkyne **12** (where Stork–Zhao olefination[14]/elimination proved the most effective means for alkynylation), and then the *para*-methoxybenzyl ether into a terminal alkyne gave **7**, which is primed for addition to AB-ring aldehydes **6** or **8**.

**Scheme 2:**
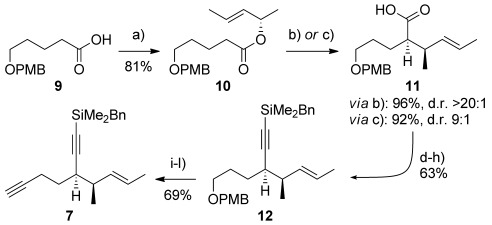
Reagents and conditions: a) (*S*,*E*)-pent-3-en-2-ol, EDC⋅HCl, Et_3_N, DMAP, THF, RT, 16 h, 81 %; b) LiHMDS, Et_3_N/toluene (3:1), −78 °C→RT, 5 h, 95 %, d.r.>20:1; c) LDA, TMSCl/Et_3_N (1:1), THF, −78 °C→0 °C, 3 h, 92 %, d.r. 9:1; d) TMSCHN_2_, toluene/MeOH (5:1), RT, 30 min, 88 %; e) DIBALH, CH_2_Cl_2_, −78 °C→−30 °C, 2 h, 97 %; f) DMP, NaHCO_3_, CH_2_Cl_2_, 0 °C→RT, 1 h, 90 %; g) [Ph_3_PCH_2_I]^+^I^−^, NaHMDS, THF, −78 °C→RT; then NaHMDS, −78 °C→RT, 84 %; h) LiHMDS, THF, −78 °C, 30 min; then BnMe_2_SiCl, −78 °C→RT, 3 h, 98 %; i) DDQ, CH_2_Cl_2_/H_2_O (4:1), RT, 1 h; j) DMP, NaHCO_3_, CH_2_Cl_2_, 0 °C→RT, 30 min, 83 % (2 steps); k) CBr_4_, PPh_3_, CH_2_Cl_2_ −30 °C→0 °C, 1 h, 85 %; l) *n*BuLi, THF, −78 °C→RT, 40 min, 98 %. Bn=benzyl, DIBALH=diisobutylaluminium hydride, DDQ=2,3-dichloro-5,6-dicyano-1,4-benzoquinone, DMAP=4-dimethylaminopyridine, DMP=Dess–Martin periodinane, EDC=1-Ethyl-3-(3-dimethylaminopropyl)carbodiimide, HMDS=1,1,1,3,3,3-hexamethyldisilazane, LDA=lithium diisopropylamide, PMB=*para*-methoxybenzyl, TMS=trimethylsilyl.

The preparation of the alkyne-bearing aldehyde **8** offered an opportunity to avoid the Stille coupling we had used to install the bromoalkene sidechain in aldehyde **6**.^[6a]^ Its synthesis commenced with ester **13** (Scheme 3), which was converted into epoxide **14** through alkyne carbocupration[15] with 3-trimethylsilylpropynylmagnesium bromide,[16] ester reduction, and Sharpless asymmetric epoxidation (83 % over 3 steps, 92 % *ee*).[17] Ring-opening of **14** with allylmagnesium chloride,[18] followed by oxidation and β-lactone formation, afforded **15** in good yield (74 % over 4 steps).[19] Double nucleophilic addition of methylmagnesium bromide to the β-lactone smoothly installed the *gem-*dimethyl group of the B ring (**16**);[20] oxidative cleavage of the terminal alkene in **16**, methyl acetal formation, and oxidation of the remaining primary alcohol afforded aldehyde regioisomers **17** and **18** (1.9:1 ratio, 72 % yield from **16**). The formation of this separable mixture of aldehydes is inconsequential since both were found to be suitable for conversion to the AB-ring aldehyde **8**. In the case of aldehyde **17**, this was achieved through Ando olefination/A-ring lactonization[21] and acetal hydrolysis to give lactol **19**. Pleasingly, exposure of this lactol to methanolic potassium carbonate gave the AB-ring aldehyde **8** in quantitative yield through oxy-Michael addition. From aldehyde **18**, a similar sequence could be applied, which proceeded via enoate **20** (*Z*/*E*=2.5:1); acidic deprotection of the acetal in **20** led to spontaneous lactonization to **19**.

**Scheme 3:**
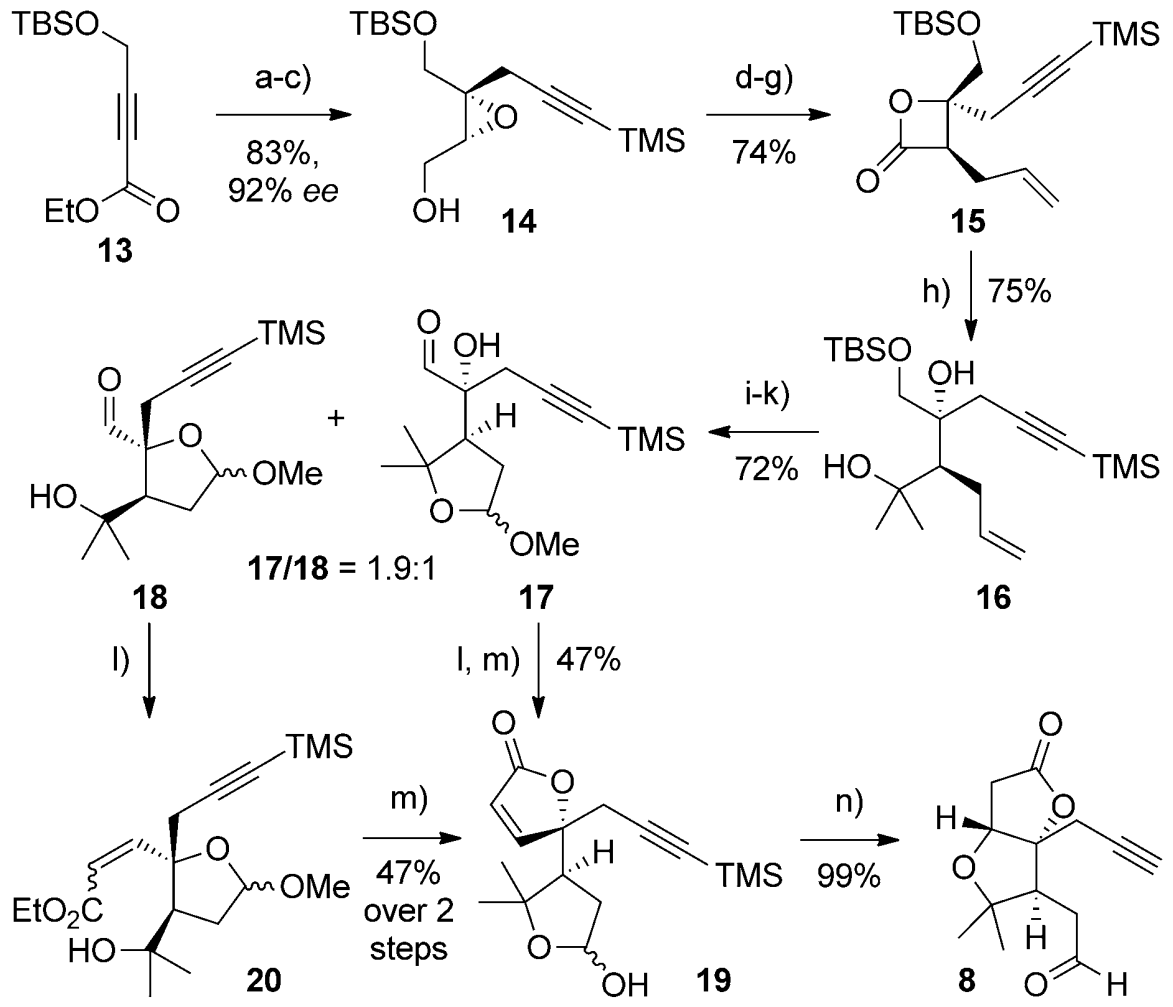
Reagents and conditions: a) TMSCαCCH_2_MgBr, CuBr⋅SMe_2_, THF, −78 °C→−40 °C, 40 min; 13, −78 °C; b) DIBALH, CH_2_Cl_2_, −78 °C→RT, 3 h, 90 % (2 steps); c) Ti(O*i*Pr)_4_, d-(−)-diethyl tartrate, *t*BuOOH, 4 Å MS, CH_2_Cl_2_, −20 °C, 22 h, 92 %, 92 % *ee*; d) AllylMgBr, THF, 0 °C, 10 min, 97 %; e) SO_3_⋅py, DMSO, *i*Pr_2_EtN, CH_2_Cl_2_, 0 °C→RT, 2 h; f) NaOCl, NaH_2_PO_4_, 2-methylbut-2-ene, *t*BuOH/H_2_O (3:1), RT, 18 h, 92 % (2 steps); g) BOPCl, py, MeCN, RT, 3 h, 83 %; h) MeMgBr, THF, −5 °C→RT, 1.5 h, 64 %+31 % ketone, recycled to give 75 % overall; i) OsO_4_, NaIO_4_, 2,6-lutidine, dioxane/H_2_O (4.6:1), RT, 2 h, 88 %; j) (±)-camphorsulfonic acid, MeOH, RT, 18 h, 98 %; k) SO_3_⋅py, DMSO, *i*Pr_2_EtN, CH_2_Cl_2_, 0–10 °C, 1 h, 84 %; l) (PhO)_2_POCH_2_CO_2_Et, KHMDS, THF, 0 °C; m) TFA, CH_2_Cl_2_, 0 °C, 15 min, 47 % (from 17, and 18); n) K_2_CO_3_, MeOH, RT, 2 h, 99 %. BOPCl=bis(2-oxo-3-oxazolidinyl)phosphinic chloride, MS=molecular sieves, py=pyridine, TBS=*tert*-butyldimethylsilyl, TFA=trifluoroacetic acid.

With the key diyne and aldehyde components in hand, we now investigated their union and cyclizations to the ABCDE ring system of rubriflordilactone A (Scheme 4). We first chose to explore the palladium-catalyzed route, which began with addition of diyne **7** to bromoalkenyl aldehyde **6** to give alcohol **21** (67 %). Prior work in the group[6b] had shown that protection of the propargylic alcohol would be required to achieve high yields in the ensuing cyclization, and accordingly a TBS ether was installed at this position. The resultant bromoendiyne was then cyclized by treatment with [Pd(PPh_3_)_4_] (10 mol %) and triethylamine in acetonitrile, which to our delight afforded the ABCDE-ring pentacycle **22** in excellent yield (91 %). Oxidation of the aryl benzyldimethylsilane to the corresponding phenol proceeded smoothly,[22] and after benzylic deoxygenation, the fully functionalized ABCDE framework **23** was revealed.

**Scheme 4:**
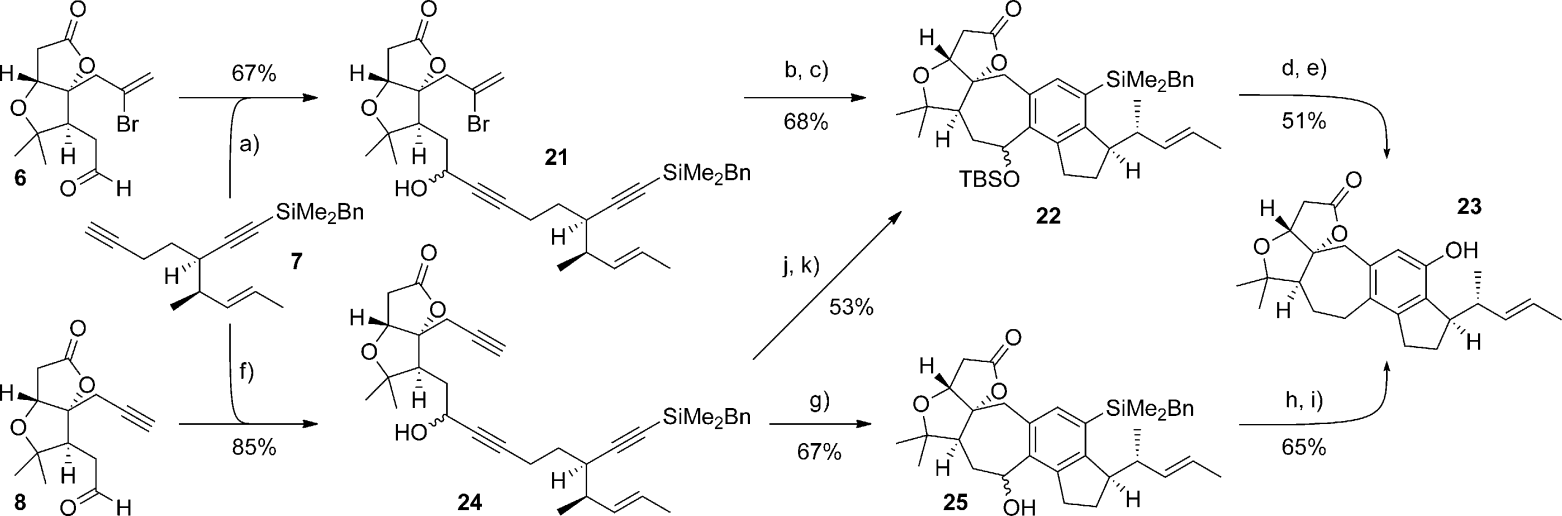
Reagents and conditions: a) *n*BuLi, 7, −78 °C; then add 6, −78 °C→−10 °C, 2 h, 67 %; b) TBSOTf, 2,6-lutidine, CH_2_Cl_2_, 0 °C→RT, 4 h 75 %; c) [Pd(PPh_3_)_4_] (10 mol %), Et_3_N, MeCN, 80 °C, 18 h, 91 %; d) TBAF, THF, RT, 30 min; then H_2_O_2_, KHCO_3_, MeOH, RT, 12 h; e) Et_3_SiH, ZnCl_2_, CH_2_Cl_2_, RT, 3 h; then TBAF, THF, RT, 20 min, 51 % (2 steps); f) *n*BuLi, 7, −78 °C; then add 8, −78 °C→−10 °C, 4 h, 85 %; g) [CpCo(CO)_2_] (20 mol %), PPh_3_ (40 mol %), PhCl, MW (300 W), 150 °C, 25 min, 67 %; h) TBAF, THF, RT, 30 min; then H_2_O_2_, KHCO_3_, MeOH, RT, 12 h, 84 %; i) Et_3_SiH, ZnCl_2_, CH_2_Cl_2_, RT, 3 h, 77 %; j) TBSCl, imid., DMAP, CH_2_Cl_2_, RT, 6 h, 98 %; k) [CpCo(CO)_2_] (20 mol %), PPh_3_ (40 mol %), PhCl, MW (300 W), 150 °C, 25 min, 54 %. Cp=cyclopentadienyl, MW=microwave irradiation, OTf=trifluoromethanesulfonate, TBAF=tetra-*n*-butylammonium fluoride.

At this juncture, we elected to compare the palladium-catalyzed cyclization route with the alternative cobalt-catalyzed cyclotrimerization approach. Diyne **7** was therefore instead combined with alkynyl aldehyde **8** to give triyne **24** (85 %). We were excited to find that cyclotrimerization of **24** under microwave heating[6b, 23] successfully afforded pentacycle **25** (67 %); this product could also be advanced to the same ABCDE-ring structure **23** prepared via the palladium-catalyzed route, through Tamao oxidation followed by benzylic deoxygenation.[22] Notably, silylation of the propargylic alcohol in **24** provides an alternative point of convergence between the two routes, since the product of cyclotrimerization of the resultant silyl ether is pentacycle **22**, albeit obtained with slightly reduced efficiency compared to cyclization of the free alcohol **24**.[24]

Our two strategies had now converged on the late-stage intermediate **23**, and all that remained was elaboration of the FG ring system. This was achieved in four steps (Scheme 5), beginning with a two-step oxidative cleavage of the pendent alkene in **23** to give the lactol **26** (in equilibrium with the open-chain aldehyde). This lactol intercepts with the synthetic route reported by the Li group,[3] who progressed **26** to the natural product through the formation of a fluoropyran, stereoselective coupling of which with a furanyl stannane installed the butenolide G ring. With a view to avoiding the use of a tin-based nucleophile, we explored alternative methods to activate the lactol. This proved challenging, but after some experimentation we found that chloropyran **27** could be prepared by treating lactol **26** with a mixture of thionyl chloride and zinc(II) chloride.[25] This seemingly straightforward transformation in fact proceeds via initial and rapid formation of dimer **28**, which over a period of 3 h is converted into the targeted chloropyran **27** (see graph in Scheme 5). This unstable intermediate was then reacted directly with siloxyfuran **28** in the presence of zinc(II) chloride and, to our delight, this reaction afforded rubriflordilactone A along with its C23-epimer **30** in 71 % yield (d.r.≈1:1). It is notable that the facial selectivity of the addition of the furan to the incipient oxocarbenium ion is excellent, since these two separable diastereomers were the predominant products formed in this addition. The spectroscopic data for the synthetic rubriflordilactone A were found to be identical to those for the natural product, with the exception of the specific rotation, which showed an equal and opposite value, thus indicating that **1** is the unnatural enantiomer (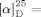 +58.3 (*c*=0.114, MeOH); lit. 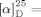
−58.1 (*c*=0.114, MeOH)).[5, 26]

**Scheme 5:**
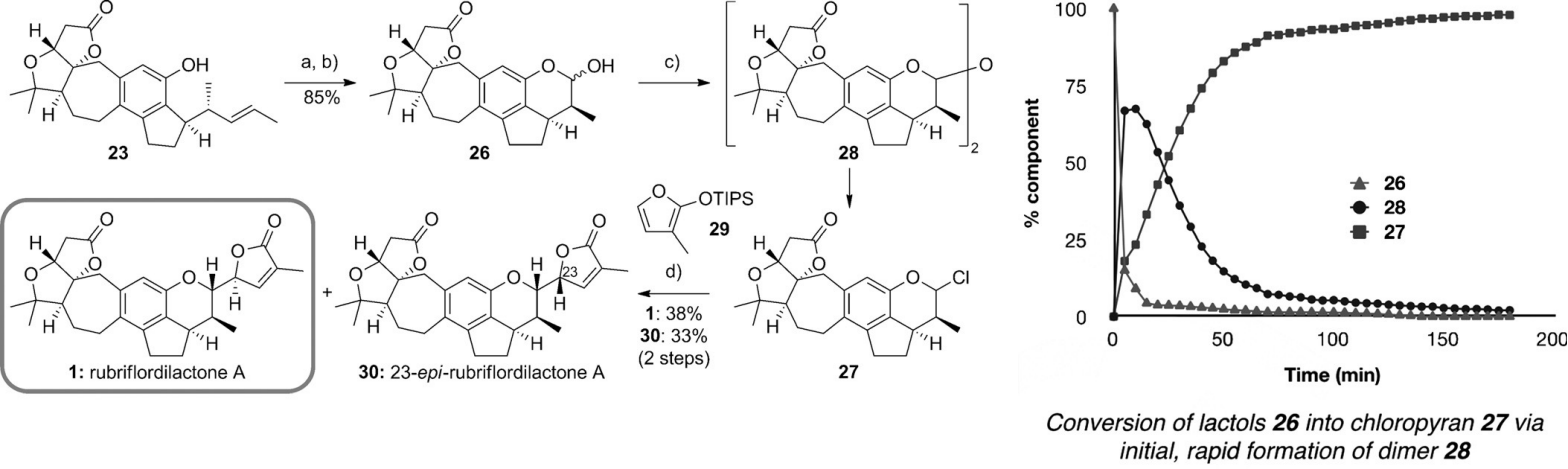
Reagents and conditions: a) OsO_4_ (2 mol %), NMO, acetone/H_2_O (3:1), RT, 3 h; b) NaIO_4_/SiO_2_, CH_2_Cl_2_, RT, 15 min, 85 % (2 steps); c) ZnCl_2_, SOCl_2_, CDCl_3_, RT, 3 h; d) 29, ZnCl_2_, CH_2_Cl_2_, −30 °C→RT, 12 h, 38 % of 1 and 33 % of 30 (2 steps). NMO=*N*-methylmorpholine-*N*-oxide, TIPS=triisopropylsilyl.

In conclusion, we have developed two synthetic strategies that achieve enantioselective syntheses of rubriflordilactone A. These employ palladium or cobalt catalysis to assemble the ABCDE ring system as the key framework-construction step. The routes are strategically highly convergent because their common late-stage intermediate is just four steps from the end of the synthesis. The modular nature of the coupling between a functionalized diyne and AB-ring aldehydes to assemble the cyclization substrates enables a unified approach to other members of this fascinating family of natural products, and offers a high degree of flexibility for the synthesis of rubriflordilactone analogues.

## Supporting Information

As a service to our authors and readers, this journal provides supporting information supplied by the authors. Such materials are peer reviewed and may be re-organized for online delivery, but are not copy-edited or typeset. Technical support issues arising from supporting information (other than missing files) should be addressed to the authors.

miscellaneous_information
