# Supplementary material for: Total Synthesis of (+)-Rubriflordilactone A
Source: Angew Chem Int Ed Engl. 2015 Sep 1;54(43):12618–21. doi: 10.1002/anie.201506366 (PMC4643188; doi:10.1002/anie.201506366)

Angewandte Chemie
Eine Zeitschrift der Gesellschaft Deutscher Chemiker

Supporting Information

Total Synthesis of (+)-Rubriflordilactone A

*Shermin S. Goh, Guilhem Chaubet, Birgit Gockel, Marie-Caroline A. Cordonnier, Hannah Baars, Andrew W. Phillips, and Edward A. Anderson**

anie_201506366_sm_miscellaneous_information.pdf

Contents

1. **EXPERIMENTAL**

1.1 General Experimental Considerations **2**

1.2 Experimental procedures and characterization of compounds **3**

*1.2.1 Synthesis of Diyne **7*** 3

*1.2.2 Synthesis of AB ring alkyne **8*** 12

*1.2.3 Synthesis of ABCDE intermdiate **23** via Pd catalysis route* 26

*1.2.4 Synthesis of ABCDE intermdiate **23** via Co catalysis route* 31

*1.2.5 Synthesis of rubriflordilactone A from intermediate **23*** 36

2. **REFERENCES** **43**

3. **NMR SPECTRA** **44**

*3.1 Intermediates in the synthesis of diyne **7*** 44

*3.2 Intermediates in the synthesis of AB ring alkyne **8*** 56

3.3 Intermediates in the synthesis of the ABCDE rings, and rubriflordilactone A 73

3.4 Spectra for rubriflordilactone A and C23-epi- rubriflordilactone A 91

1. EXPERIMENTAL

1.1 General Experimental Considerations

Nuclear Magnetic Resonance Spectroscopy: ^{1}H NMR spectra were acquired on Bruker DRX500, AVII500 (500 MHz, with cryoprobe) or AVIII400 (400 MHz) spectrometers and were referenced to residual non-deuterated solvent peaks in $CDCl_3$ (δ = 7.26) or C_5D_5N (δ = 8.74, 7.58, 7.22). Chemical shifts (δ_H and δ_C) are reported in parts per million (ppm) with signal splittings recorded as singlet (s), doublet (d), triplet (t), quartet (q), quintet (quin), and multiplet (m); app = apparent. Coupling constants (*J*) are measured to the nearest 0.1 Hz and are presented as observed. ^{13}C NMR spectra were obtained on Bruker AVII500 (126 MHz, with cryoprobe) or AVIII400 (101 MHz) spectrometers and were referenced to solvent peaks in $CDCl_3$ (δ = 77.16) or C_5D_5N (150.35, 135.91, 123.87).

Mass Spectrometry: Low-resolution mass spectra (*m/z*) were recorded on a Waters LCT Premier EX mass spectrometer, using electrospray ionization (ESI). High-resolution mass spectra (HRMS) were recorded by the Departmental Mass Spectrometry Service, University of Oxford on a Bruker MicroTOF (resolution = 5000 FWHM) using electrospray ionisation (ES^+). The parent ion $[M]^+$, $[M+H]^+$ or $[M+Na]^+$ is calculated to 4 decimal places from the molecular formula, and all values are within a tolerance of 5 ppm.

Infrared Spectroscopy: Absorption spectra were obtained in $CHCl_3$ as solvent on a Bruker Tensor 27 FT-IR spectrometer. The sample was prepared as a thin film on a diamond/ZnSe PIKE Miracle ATR module. Wavelengths of maximum absorbance (ν_{max}) are quoted in wavenubers (cm^{-1}). Only selected, characteristic IR absorption data are provided for each compound.

Specific rotations: Optical rotations were recorded on a Perkin Elmer 241 or 341 polarimeter with a path length of 1 dm (using the sodium D line, 589 nm). Specific rotations ($[\alpha]_D$) are reported in units of 10^{-1} deg cm^2 g^{-1}. Concentrations are reported in g/100 mL. Temperatures are reported in °C (typically 25 °C).

Chromatography: Flash chromatography refers to normal phase column chromatography on silica gel using a head pressure of N_2, using either Merck Geduran® Silicagel 60 (40–63 μm) or Macherey-Nagel Silica 60 M (40 - 63 μm). Thin-layer chromatography was performed on Merck Kieselgel 60 F_{254} plates with visualization by ultraviolet light (254 nm) and/or heating the plate after staining with vanillin or $KMnO_4$. High performance liquid chromatography (HPLC) was performed on an Agilent 1200 Series running in normal phase under UV detection using a ZORBAX RX-SIL (150 mm x 4.6 mm ID) as the analytical column. Chiral analysis was carried out using DAICEL CHIRALPAK-IA, IB or IC (250 mm x 4.6 mm ID).

Materials: Unless otherwise stated, all reactions were carried out in oven-dried glassware under an atmosphere of argon, using anhydrous reaction solvents. Et_2O, CH_2Cl_2, THF and toluene were dried over activated alumina before use. All other commercially available reagents and solvents were either used as received, and/or dried and purified before use using standard procedures. Petroleum ether refers to the fraction of light petroleum ether boiling at 40–60 °C unless stated otherwise.

1.2. Experimental procedures and characterization of compounds

1.2.1. Synthesis of Diyne 7

Diyne **7** was prepared according to the following synthetic scheme:

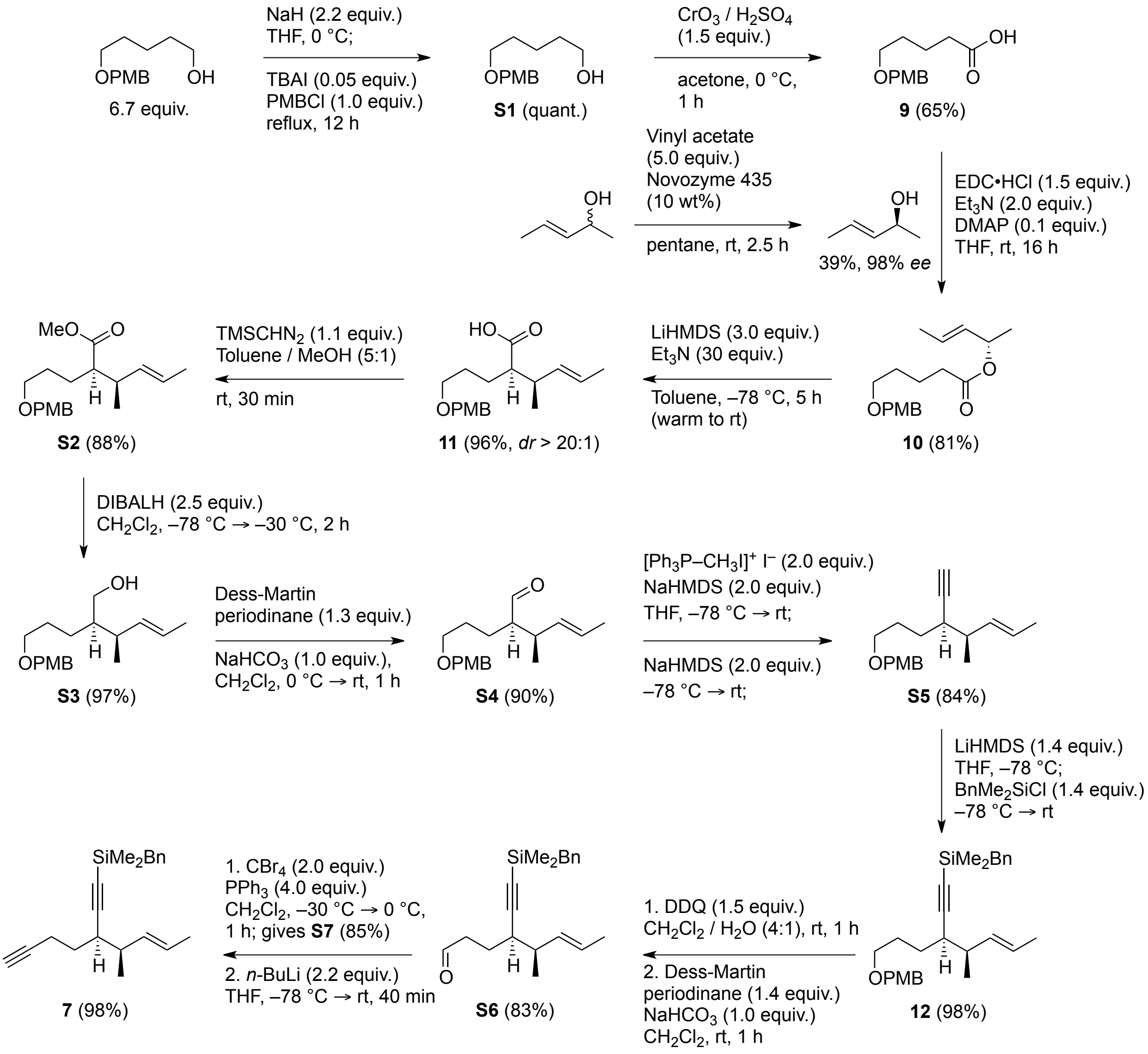

5-((4-methoxybenzyl)oxy)pentan-1-ol, S1

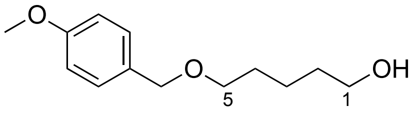

To a stirred suspension of NaH (60% dispersion in mineral oil, 5.20 g, 130 mmol, 2.2 equiv.) in THF (150 mL) under argon at 0 °C was added a solution of 1,5-pentane diol (43.6 mL, 395 mmol, 6.7 equiv.) in THF. The reaction mixture was heated to reflux for 3 h, then allowed to cool to rt before TBAI (1.10 g, 2.00 mmol, 0.05 equiv.) and PMBCl (8.0 mL, 59.0 mmol, 1.0 equiv.) were added. The reaction mixture was again heated to reflux (12 h), then allowed to cool to rt. Water was added, then the mixture was filtered through a short pad of Celite®. The filtrate was concentrated, and the residue was taken up in CH-$_2Cl_2$ and water. The layers were separated and the aqueous phase extracted three times with CH_2Cl_2. The combined organic phases were dried ($MgSO_4$) and concentrated. The product was purified by flash

chromatography on a short plug of silica (2:1→1:1 petroleum ether / EtOAc eluent), to afford alcohol **S1** (13.2 g, 58.8 mmol, quant.) as a colourless oil. **^{1}H NMR** (400 MHz, $CDCl_3$) δ_H 7.26 (2H, d, *J* = 8.7 Hz, ArH), 6.88 (2H, d, *J* = 8.7 Hz, ArH), 4.43 (2H, s, C$\underline{H_2}$Ar), 3.80 (3H, s, OMe), 3.64 (2H, t, *J* = 6.6 Hz, H1), 3.45 (2H, t, *J* = 6.5 Hz, H5), 1.70-1.52 (4H, m, H2 and H4), 1.49-1.37 (3H, m, H3 and OH); **^{13}C NMR** (101 MHz, $CDCl_3$) δ_C 159.3, 130.8, 129.4, 113.6, 72.7, 70.1, 63.0, 55.4, 32.7, 29.6, 22.5. Data in accordance with literature values.[1]

5-((4-methoxybenzyl)oxy)pentanoic acid, 9

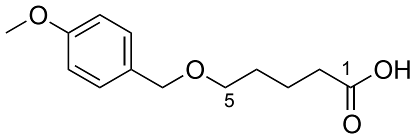

To a stirred solution of alcohol **S1** (14.0 g, 62.1 mmol, 1.0 equiv.) in acetone (120 mL) at 0 °C was added Jones' reagent (2.5 M CrO_3 / H_2SO_4, 37.4 mL, 94 mmol, 1.5 equiv.). The reaction mixture was stirred at 0 °C for 1 h, then the reaction was quenched by addition of propan-2-ol (15 mL), and then carefully neutralised with $NaHCO_3$. The solution was then diluted with EtOAc and washed with 3N NaOH (100 mL). The layers were separated, and the aqueous phase was acidified with conc. HCl, then extracted three times with EtOAc (3 x 200 mL). The combined organic layers were dried ($MgSO_4$) and concentrated to give acid **9** (14.0 g, 56.8 mmol, 91%) as a white solid. **R_f** 0.15 (2:1 petroelum ether / Et_2O); **^{1}H NMR** (500 MHz, $CDCl_3$) δ_H 7.25 (2H, d, *J* = 8.5 Hz, ArH), 6.88 (2H, d, *J* = 8.5 Hz, ArH), 4.43 (2H, s, C$\underline{H_2}$Ar), 3.80 (3H, s, OMe), 3.46 (2H, t, *J* = 6.1 Hz, H5), 2.37 (2H, t, *J* = 7.3 Hz, H2), 1.77-1.69 (2H, m, H4), 1.69-1.62 (2H, m, H3); **^{13}C NMR** (126 MHz, $CDCl_3$) δ_C 179.3, 159.3, 130.6, 129.4, 113.9, 72.7, 69.6, 55.4, 33.7, 29.1, 21.7. Data in accordance with literature values.[2]

(*S*,*E*)-pent-3-en-2-ol

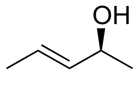

To a stirred solution of crotonaldehyde (2.0 mL, 24.1 mmol, 1.0 equiv.) in Et_2O (10 mL) under argon at 0 °C was added methyllithium (1.5 M in diethyl ether, 19.3 mL, 29.0 mmol, 1.2 equiv.) dropwise. The reaction mixture was warmed to rt and stirred for 1.5 h, then the reaction was quenched by addition of NH_4Cl (sat. aq.). The layers were separated and the aqueous phase was extracted three times with Et_2O. The combined organic layers were dried ($MgSO_4$) and concentrated. The product was purified by flash chromatography on a short plug of silica gel (3:1→1:1 30-40 petroleum ether / Et_2O), to give pent-3-en-2-ol (1.97 g, 22.9 mmol, 95%) as a colourless oil.

Kinetic Resolution: To a stirred solution of racemic pent-3-en-2-ol (5.54 g, 64.3 mmol, 1.0 equiv.) in anhydrous pentane (33 mL) under Ar was added vinyl acetate (29.7 mL, 322 mmol, 5.0 equiv.), activated 4 Å molecular sieves (crushed, 2.72 g, 50 wt%) and Novozyme 435 (554 mg, 10 wt%). The reaction mixture was stirred for 2.5 h at rt, then it was filtered through a pad of Celite®, and the filtrate was concentrated. The product was purified by flash chromatography on a short plug of silica (3:1→1:1 30-40 petroleum ether / Et_2O), to give (*S*,*E*)-pent-3-en-2-ol (2.16 g, 25.1 mmol, 39%) as a colourless oil.

$[\alpha]_D^{25}$ –10.5 (*c* = 1.00, $CHCl_3$); **R_f** 0.45 (1:1, 30-40 petroleum ether / Et_2O); **^{1}H NMR** (400 MHz, $CDCl_3$) δ_H 5.64 (1H, dqd, *J* = 15.2, 6.4 and 0.7 Hz, H4), 5.52 (1H, ddq, *J* = 15.2, 6.4 and 1.2 Hz, H3), 4.24 (1H, quin, *J* = 6.4 Hz, H2), 1.67 (3H, dd, *J* = 6.4 and 1.2 Hz, H5), 1.24 (3H, d, *J* = 6.4 Hz, H1); **^{13}C NMR** (101 MHz, $CDCl_3$) δ_C 135.5, 125.9, 69.1, 23.5, 17.7; Data in accordance with literature values.[3]

The enantiomeric excess was determined as follows: To a solution of the enantioenriched alcohol (20 mg) in CH_2Cl_2 (0.5 mL) was added benzoyl chloride (33 mg), Et_3N (46 µL) and DMAP (1 mg). The reaction was stirred for 3 h at rt, then it was quenched with NH_4Cl (sat., aq.). The layers were separated and the aqueous layer was extracted with CH_2Cl_2. The combined organic layers were dried (Na_2SO_4) and concentrated; the product was purified via flash chromatography (4:1 30-40 petroleum ether / Et_2O) to yield the corresponding benzoate ester, which was used for HPLC analysis. This revealed a **99% *ee*** (CHIRALPAK IA, 0.6 mL/min, 0.5% IPA/hexanes, R_t (*R*) – 8.54 min, R_t (*S*) – 9.86 min).

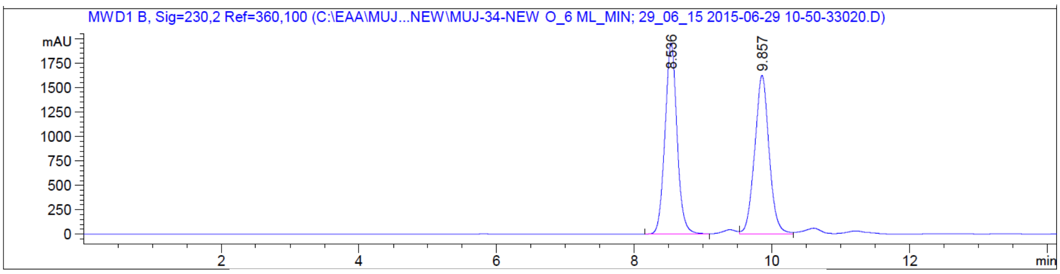

Peak #	RetTime [min]	Type	Width [min]	Area [mAU*s]	Height [mAU]	Area %
1	8.536	VV	0.1845	2.38733e4	1954.12146	50.1369
2	9.857	VV	0.2164	2.37429e4	1625.66553	49.8631
Totals :				4.76162e4	3579.78699	

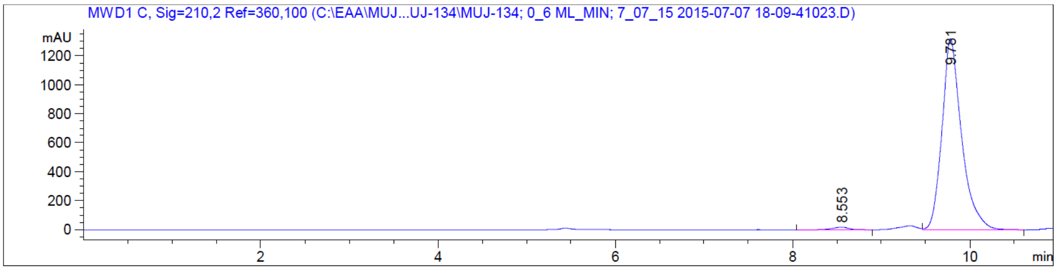

Peak #	RetTime [min]	Type	Width [min]	Area [mAU*s]	Height [mAU]	Area %
1	8.553	VV	0.1735	260.37378	19.04672	1.2658
2	9.781	VV	0.2197	2.03101e4	1318.34253	98.7342
Totals :				2.05704e4	1337.38925	

The absolute configuration was also determined by Mosher ester analysis. A single diastereomer was formed with each enantiomer of the α-methoxy- α-trifluoromethylphenylacetic acid. ^{1}H NMR data are as follows (this allowed confirmation of the assignment of stereochemistry as (*S*)):

S-MTPA-ester derivative: **^{1}H NMR** (400 MHz, $CDCl_3$) δ_H 5.83 (1H, dq, *J* = 6.6 and 13.9 Hz, H4), 5.55 (1H, m, H2), 5.53 (1H, m, H3), 1.71 (3H, d, *J* = 6.6 Hz, H5), 1.32 (3H, d, *J* = 5.9 Hz, H1).

R-MTPA-ester derivative: **^{1}H NMR** (400 MHz, $CDCl_3$) δ_H 5.75 (1H, dq, *J* = 6.7 and 15.2 Hz, H4), 5.53 (1H, app quint, *J* = 6.7 Hz, H2), 5.41 (1H, dd, *J* = 7.1 and 15.2 Hz, H3), 1.66 (3H, d, *J* = 6.6 Hz, H5), 1.38 (3H, d, *J* = 6.5 Hz, H1).

(*S,E*)-pent-3-en-2-yl 5-((4-methoxybenzyl)oxy)pentanoate, 10

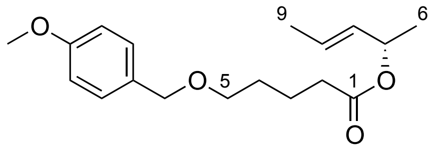

To a stirred solution of (*S,E*)-pent-3-en-2-ol (2.78 g, 32.3 mmol, 1.0 equiv.) and acid **9** (8.46 g, 35.5 mmol, 1.1 equiv.) in THF (28 mL) was added EDC•HCl (9.28 g, 48.4 mmol, 1.5 equiv.), triethylamine (8.97 mL, 64.6 mmol, 2.0 equiv.) and DMAP (394 mg, 3.23 mmol, 0.1 equiv.). The reaction mixture was stirred at rt for 16 h, then it was quenched with water. The layers were separated and the aqueous layer extracted three times with EtOAc. The combined organic phases were washed with 1N HCl, then dried ($MgSO_4$) and concentrated. The product was purified by flash chromatography on a short plug of silica (4:1 petroleum ether / EtOAc), to give ester **10** (7.97 g, 26.0 mmol, 81%) as a colourless oil. $[\alpha]_D^{25}$ +26.8 (*c* = 1.00, $CHCl_3$); R_f 0.27 (2:1 petroleum ether / Et_2O); **^{1}H NMR** (400 MHz, $CDCl_3$) δ_H 7.25 (2H, d, *J* = 8.6 Hz, ArH), 6.87 (2H, d, *J* = 8.6 Hz, ArH), 5.71 (1H, dqd, *J* = 15.4, 6.5 and 0.8 Hz, H8), 5.46 (1H, ddq, *J* = 15.4, 6.8 and 1.5 Hz, H7), 5.30 (1H, quin, *J* = 6.5 Hz, H6), 4.42 (2H, s, C$\underline{H_2}$Ar), 3.80 (3H, s, O$\underline{Me}$), 3.45 (2H, t, *J* = 6.2 Hz, H5), 2.30 (2H, t, *J* = 7.1 Hz, H2), 1.75-1.70 (2H, m, H3), 1.68 (2H, dd, *J* = 6.5 and 0.7 Hz, H9), 1.67-1.60 (2H, m, H4), 1.27 (3H, d, *J* = 6.6 Hz, H10); **^{13}C NMR** (101 MHz, $CDCl_3$) δ_C 173.0, 159.3, 131.0, 130.8, 129.4, 128.2, 113.9, 72.7, 71.1, 69.7, 55.4, 34.5, 29.3, 21.9, 20.5, 17.8. Data in accordance with literature values.[2]

(2*R*,3*R*,*E*)-2-(3-((4-methoxybenzyl)oxy)propyl)-3-methylhex-4-enoic acid, 11[2, 4]

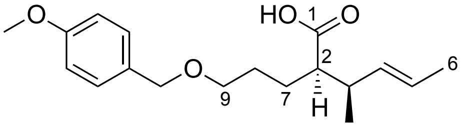

To a stirred solution of LiHMDS (1 M in toluene, 36.9 mL, 36.9 mmol, 3.0 equiv.) and triethylamine (51.3 mL, 369 mmol, 30 equiv.) in toluene (150 mL) under argon at –78 °C was added dropwise a solution of ester **9** (3.77 g, 12.3 mmol, 1.0 equiv.) in toluene (5.2 mL). The reaction was warmed to rt over 5 h, then poured into 5% NaOH (aq.) (370 mL). Et_2O was added and the layers were separated. The aqueous layer was cooled to 0 °C, then acidified with conc. HCl. Et_2O was added, the layers were separated and the aqueous phase extracted three times with Et_2O. The combined organic layers were dried ($MgSO_4$) and concentrated. The product was purified by flash chromatography on a short plug of silica (5:1 petroleum ether / EtOAc +1% AcOH) to afford acid **11** (3.63 g, 11.8 mmol, 96%) as a colourless oil. $[\alpha]_D^{25}$ –29.2 (*c* = 1.00, $CHCl_3$); R_f 0.43 (1:1 petroleum ether / EtOAc); **^{1}H NMR** (500 MHz, $CDCl_3$) δ_H 11.06 (1H, br s, COOH), 7.26 (2H, d, *J* = 8.6 Hz, ArH), 6.88 (2H, d, *J* = 8.6 Hz, ArH), 5.47 (1H, dq, *J* = 15.1 and 6.3 Hz, H5), 5.23 (1H, ddd, *J* = 15.1, 8.6 and 1.3 Hz, H4), 4.43 (2H, s, C$\underline{H_2}$Ar), 3.81 (3H, s, O$\underline{Me}$), 3.53-3.38 (2H, m, H9), 2.41-2.29 (1H, m, H3), 2.22-2.11 (1H, m, H2), 1.67 (3H, d, *J* = 6.6 Hz, H6), 1.66-1.60 (2H, m, H9), 1.60-1.53 (2H, m, H8), 1.03 (3H, d, *J* = 6.6 Hz, Me); **^{13}C NMR** (126 MHz, $CDCl_3$) δ_C 181.8, 159.2, 134.0, 130.6, 129.4, 125.9, 113.9, 72.5, 69.7, 55.3, 51.5, 39.8, 27.8, 27.0, 19.3, 18.0. Data in accordance with literature values.[2]

(2*R*,3*R*,*E*)-Methyl 2-(3-((4-methoxybenzyl)oxy)propyl)-3-methylhex-4-enoate, S2

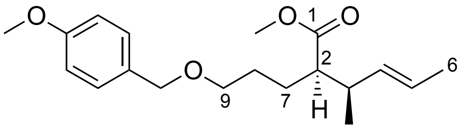

To a stirred solution of acid **11** (593 mg, 1.94 mmol, 1.0 equiv.) in 5:1 toluene / methanol (55 mL) under Ar at 0 °C was slowly added $TMSCHN_2$ (1.16 mL, 2.13 mmol, 1.1 equiv.). The reaction mixture was stirred at rt for 30 min before being quenched with acetic acid (0.5 mL), then it was diluted with water and extracted with Et_2O. The combined organic phases were dried ($MgSO_4$) and concentrated. The residue was purified by flash chromatography (9:1 petroleum ether / Et_2O) to afford methyl ester **S2** (549 mg, 1.71 mmol, 88%) as a colourless oil. **$[\alpha]_D^{25}$** +15.0 (*c* = 0.12, $CHCl_3$); **R_f** 0.38 (7:3 petroleum ether / Et_2O); **IR** (thin film, ν_{max} / cm^{-1}) 2951, 1733, 1513, 1248, 1036, 821; **^{1}H NMR** (500 MHz, $CDCl_3$) δ_H 7.24 (2H, d, *J* = 8.6 Hz, ArH), 6.87 (2H, d, *J* = 8.6 Hz, ArH), 5.43 (1H, dq, *J* = 15.1 and 6.3 Hz, H5), 5.20 (1H, ddd, *J* = 15.1, 8.7 and 1.6 Hz, H4), 4.41 (2H, s, C$\underline{H_2}$Ar), 3.80 (3H, s, O$\underline{Me}$), 3.66 (3H, s, $CO_2\underline{Me}$), 3.45-3.35 (2H, m, H9), 2.35-2.26 (1H, m, H3), 2.15 (1H, td, *J* = 9.5 and 3.5 Hz, H2), 1.65 (3H, dd, *J* = 6.3 and 1.3 Hz, H6), 1.63-1.56 (2H, m, H8), 1.54-1.48 (2H, m, H7), 0.94 (3H, d, *J* = 6.9 Hz, Me); **^{13}C NMR** (126 MHz, $CDCl_3$) δ_C 176.3, 159.3, 134.3, 130.8, 129.4, 125.7, 113.7, 72.6, 69.8, 55.4, 51.7, 51.4, 40.1, 28.0, 27.3, 19.3, 18.0; **HRMS** (ES$^+$) calc. for $C_{19}H_{28}NaO_4$ $[M+Na]^+$ 343.1880; found 343.1873.

(2*R*,3*R*,*E*)-2-(3-((4-Methoxybenzyl)oxy)propyl)-3-methylhex-4-en-1-ol, S3

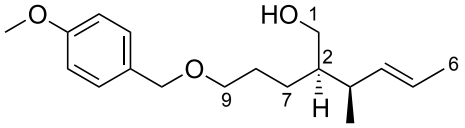

To a stirred solution of methyl ester **S2** (4.80 g, 15.0 mmol, 1.0 equiv.) in CH_2Cl_2 (45 mL) under argon at –78 °C was added a solution of DIBALH (1.0 M in hexane, 37.5 mL, 37.5 mmol, 2.5 equiv.). The solution was warmed to –30 °C over 2 h, then the reaction was quenched by addition of sodium potassium tartrate (sat., aq.), and the mixture was stirred vigorously for a further 1 h. The layers were separated and the aqueous phase was extracted three times with EtOAc. The combined organic layers were dried ($MgSO_4$), then filtered through Celite® and the filtrate was concentrated. The product was purified by flash chromatography (2:1 petroleum ether / Et_2O) to yield the alcohol **S3** (4.23 g, 14.5 mmol, 97%) as a colourless oil. **$[\alpha]_D^{25}$** +17.4 (*c* = 1.00, $CHCl_3$); **R_f** 0.23 (1:1 petroleum ether / Et_2O); **IR** (thin film, ν_{max} / cm^{-1}) 3647, 3385, 2933, 2359, 1513, 1248; **^{1}H NMR** (500 MHz, $CDCl_3$) δ_H 7.26 (2H, d, *J* = 8.8 Hz, ArH), 6.88 (2H, d, *J* = 8.8 Hz, ArH), 5.48-5.40 (1H, dq, *J* = 15.1 and 6.0 Hz, H5), 5.40-5.34 (1H, m, H4), 4.43 (2H, s, C$\underline{H_2}$Ar), 3.80 (3H, s, O$\underline{Me}$), 3.58 (2H, app t, *J* = 5.2 Hz, H1), 3.44 (2H, t, *J* = 6.5 Hz, H9), 2.37-2.25 (1H, m, H3), 1.72-1.66 (1H, m, H8), 1.65 (3H, d, *J* = 6.0 Hz, H6), 1.63-1.53 (1H, m, H8), 1.49 (1H, br s, OH), 1.48-1.44 (1H, m, H7), 1.44-1.39 (1H, m, H2), 1.32-1.23 (1H, m, H7), 0.98 (3H, d, *J* = 6.9 Hz, Me); **^{13}C NMR** (126 MHz, $CDCl_3$) δ_C 159.3, 135.4, 130.8, 129.4, 124.6, 113.9, 72.7, 70.5, 64.1, 55.4, 45.8, 37.8, 27.9, 24.4, 18.2, 17.7; **HRMS** (ES$^+$) calc. for $C_{18}H_{28}NaO_3$ $[M+Na]^+$ 315.1931; found 315.1925.

(2*R*,3*R*,*E*)-2-(3-((4-methoxybenzyl)oxy)propyl)-3-methylhex-4-enal, S4

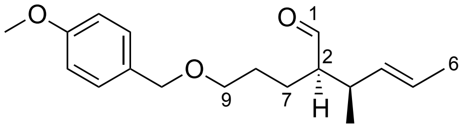

To a stirred solution of alcohol **S3** (4.60 g, 15.7 mmol, 1.0 equiv.) in CH_2Cl_2 (480 mL) under Ar at 0 °C was added Dess-Martin periodinane (8.67 g, 20.5 mmol, 1.3 equiv.) and $NaHCO_3$ (1.33 g, 15.7 mmol, 1.0 equiv.). The reaction mixture was stirred at rt for 1 h before being quenched by addition of $Na_2S_2O_3$ (100 mL, sat., aq.) and $NaHCO_3$ (100 mL, sat., aq.). The mixture was stirred for 30 min, then the layers were separated and the aqueous phase extracted three times with CH_2Cl_2. The combined organic layers were dried ($MgSO_4$) and concentrated. The residue was purified by flash chromatography (2:1 petroleum ether / Et_2O) to yield aldehyde **S4** (4.10 g, 14.1 mmol, 90%) as a colourless oil. **$[\alpha]_D^{25}$** +8.3 (*c* = 1.00, $CHCl_3$); **R_f** 0.45 (7:3 petroleum ether / EtOAc); **IR** (thin film, ν_{max} / cm^{-1}) 2933, 1722, 1513, 1247, 1098, 411; **^{1}H NMR** (500 MHz, $CDCl_3$) δ_H 9.56 (1H, d, *J* = 3.8 Hz, H1), 7.25 (2H, d, *J* = 8.7 Hz, ArH), 6.87 (2H, d, *J* = 8.7 Hz, ArH), 5.46 (1H, dqd, *J* = 15.2, 6.3 and 1.2 Hz, H5), 5.28 (1H, ddq, *J* = 15.2, 8.1 and 1.6 Hz, H4), 4.41 (2H, s, C<u>H</u>$_2$Ar), 3.80 (3H, s, O<u>Me</u>), 3.42 (2H, t, *J* = 5.4 Hz, H9), 2.45 (1H, app sext, *J* = 7.1 Hz, H3), 2.22-2.01 (1H, m, H2), 1.66 (3H, dd, *J* = 6.3 and 1.2 Hz, H6), 1.64-1.51 (4H, m, H7 and H8), 1.00 (3H, d, *J* = 6.9 Hz, Me); **^{13}C NMR** (126 MHz, $CDCl_3$) δ_C 205.7, 159.3, 133.5, 130.7, 129.4, 126.0, 113.9, 72.6, 69.7, 57.3, 55.4, 37.4, 27.8, 23.5, 18.6, 18.0; **HRMS** (ES$^+$) calc. for $C_{18}H_{26}NaO_3$ $[M+Na]^+$ 313.1774; found 313.1774.

1-((((4*R*,5*R*,*E*)-4-Ethynyl-5-methyloct-6-en-1-yl)oxy)methyl)-4-methoxybenzene, S5

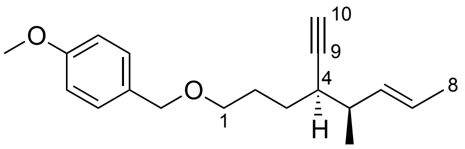

To a stirred suspension of (iodomethyl)triphenylphosphonium iodide (183 mg, 0.344 mmol, 2.0 equiv.) in THF (1.5 mL) under Ar at rt was added NaHMDS (2.0 M in hexane, 172 μL, 0.344 mmol, 2.0 equiv.). After stirring for 20 min, the solution was cooled to –78 °C and a solution of aldehyde **S4** (50.0 mg, 0.172 mmol, 1.0 equiv.) in THF (0.3 mL) was added. After 15 min, the reaction mixture was warmed to rt over 30 min until TLC showed complete formation of the *cis*-vinyl iodide derivative. The reaction mixture was then cooled to –78 °C, and additional NaHMDS (2.0 M in hexane, 172 μL, 0.344 mmol, 2.0 equiv.) was added. The reaction mixture was warmed to rt over 30 min and stirred for a further 10 min before being quenched with NH_4Cl (sat., aq.). Et_2O was added, the layers were separated, and the aqueous phase was extracted three times with Et_2O. The combined organic layers were dried ($MgSO_4$) and concentrated. The product was purified by flash chromatography (9:1 petroleum ether / Et_2O) to yield alkyne **S5** (41.4 mg, 0.144 mmol, 84%) as a colourless oil. **$[\alpha]_D^{25}$** –22.8 (*c* = 1.00, $CHCl_3$); **IR** (thin film, ν_{max} / cm^{-1}) 3260, 2987, 2876, 1613, 1580, 1215, 1090, 694; **R_f** 0.36 (19:1 petroleum ether / Et_2O); **^{1}H NMR** (500 MHz, $CDCl_3$) δ_H 7.26 (3H, d, *J* = 8.6 Hz, ArH), 6.88 (2H, d, *J* = 8.6 Hz, ArH), 5.45 (1H, dq, *J* = 15.1 and 6.0 Hz, H7), 5.30 (1H, dqd, *J* = 15.1, 7.9 and 1.4 Hz, H6), 4.43 (2H, d, *J* = 2.5 Hz, C<u>H</u>$_2$Ar), 3.81 (3H, s, O<u>Me</u>), 3.53-3.36 (2H, m, H1), 2.26-2.20 (1H, m, H4), 2.15 (1H, app sextet, *J* = 6.8 Hz, H5), 2.06 (1H, d, *J* = 2.5 Hz, H10), 1.92-1.79 (1H, m, H2), 1.70-1.64 (1H, m, H2), 1.66 (3H, dd, *J* = 6.1, 0.9 Hz, H8), 1.64-1.55 (1H,

m, H3), 1.46-1.37 (1H, m, H3), 1.07 (3H, d, *J* = 6.6 Hz, Me); **^{13}C NMR** (126 MHz, $CDCl_3$) δ_C 159.3, 135.2, 130.9, 129.4, 124.9, 113.9, 86.4, 72.6, 70.6, 69.9, 55.4, 40.7, 37.7, 29.5, 27.8, 18.1, 17.7; **HRMS** (ES^+) calc. for $C_{19}H_{28}NaO_2$ $[M+Na]^+$ 309.1825; found 309.1823.

Benzyl((3*R*,4*R*,*E*)-3-(3-((4-methoxybenzyl)oxy)propyl)-4-methylhept-5-en-1-yn-1-yl)dimethylsilane 12

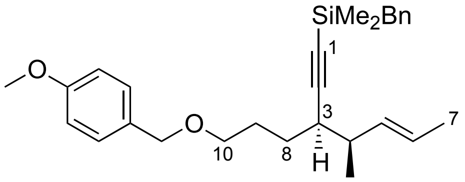

To a stirred solution of alkyne **S5** (500 mg, 1.75 mmol, 1.0 equiv.) in THF (8.0 mL) under Ar at –78 °C was added LiHMDS (1 M in THF, 2.4 mL, 2.4 mmol, 1.4 equiv.). The mixture was stirred for 30 min at –78 °C, followed by addition of a solution of $BnMe_2SiCl$ (451 mg, 2.4 mmol, 1.4 equiv.) in THF (0.7 mL). The mixture was stirred for a further 30 min at –78 °C, then warmed to rt and stirred for 3 h before being quenched with NH_4Cl (sat., aq.). Et_2O was added, the layers were separated, and the aqueous phase was extracted three times with Et_2O. The combined organic phases were dried ($MgSO_4$) and concentrated. The product was purified by flash chromatography (19:1 petroleum ether / Et_2O) to yield the alkynylsilane **12** (741 mg, 1.70 mmol, 98%) as a colourless oil. $[\alpha]_D^{25}$ +17.8 (*c* = 1.00, $CHCl_3$); R_f 0.57 (9:1 petroleum ether / Et_2O); **IR** (thin film, ν_{max} / cm^{-1}) 2929, 2165, 1728, 1612, 1247, 1097, 828; **^{1}H NMR** (500 MHz, $CDCl_3$) δ_H 7.27 (2H, d, *J* = 8.3 Hz, PMB-ArH), 7.20 (2H, d, *J* = 8.5 Hz, Bn-ArH), 7.08 (3H, *app* d, *J* = 6.8 Hz, Bn-ArH), 6.88 (2H, d, *J* = 8.3 Hz, PMB-ArH), 5.41 (1H, dq, *J* = 15.2 and 6.1 Hz, H6), 5.32 (1H, ddd, *J* = 15.2, 8.1 and 1.3 Hz, H5), 4.43 (2H, d, *J* = 1.9 Hz , C$\underline{H_2}$Ar), 3.80 (3H, s, O$\underline{Me}$), 3.49-3.40 (2H, m, H10), 2.25-2.19 (1H, m, H3), 2.17 (2H, s, SiC$\underline{H_2}$Ph), 2.12 (1H, app q, *J* = 7.0 Hz, H4), 1.88-1.79 (1H, m, H9), 1.65 (3H, dd, *J* = 6.1 and 1.3 Hz, H7), 1.65-1.62 (1H, m, H8), 1.61-1.52 (1H, m, H9), 1.37 (1H, dtd, *J* = 13.0, 10.0 and 4.7 Hz, H9), 1.04 (3H, d, *J* = 6.8 Hz, Me), 0.10 (6H, s, Si(C$\underline{H_3}$)$_2$Bn); **^{13}C NMR** (126 MHz, $CDCl_3$) δ_C 159.3, 139.5, 135.3, 130.9, 129.4, 128.5, 128.2, 124.7, 124.3, 113.9, 110.7, 85.1, 72.5, 70.0, 55.4, 40.9, 38.9, 29.4, 27.8, 26.7, 18.1, 17.9, –1.6; **HRMS** (ES^+) calc. for $C_{28}H_{38}NaO_2Si$ $[M+Na]^+$ 457.2533; found 457.2532.

(4*R*,5*R*,*E*)-4-((Benzyldimethylsilyl)ethynyl)-5-methyloct-6-enal, S6

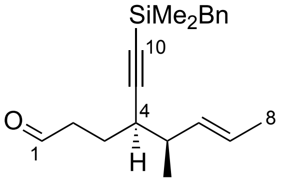

To a stirred solution of PMB ether **12** (209 mg, 0.48 mmol, 1.0 equiv.) in CH_2Cl_2 (3.9 mL) and water (0.9 mL) under Ar was added DDQ (164 mg, 0.72 mmol, 1.5 equiv.). The reaction mixture was stirred for 1 h, then it was quenched by addition of $NaHCO_3$ (sat., aq.). The layers were separated and the aqueous phase extracted three times with CH_2Cl_2. The combined organic layers were dried ($MgSO_4$) and concentrated. The crude alcohol was used in the next step without further purification.

To a stirred solution of this alcohol in CH_2Cl_2 (4.0 mL) under Ar at 0 °C was added Dess-Martin periodinane (286 mg, 0.68 mmol, 1.4 equiv.) and $NaHCO_3$ (41 mg, 0.48 mmol, 1.0 equiv.). The reaction mixture was stirred for 1 h at rt before being quenched by addition of $Na_2S_2O_3$ (2 mL, sat., aq.) and $NaHCO_3$ (2 mL, sat., aq.). The mixture was stirred for 30 min, then the layers were separated and the aqueous phase extracted three times with CH_2Cl_2. The combined organic phases were dried ($MgSO_4$) and concentrated. The residue was purified by flash chromatography (19:1 petroleum ether / Et_2O) to afford aldehyde **S6** (125 mg, 0.40 mmol, 83%) as a colourless oil. $[\alpha]_D^{25}$ +68.6 (*c* = 0.99, $CHCl_3$); R_f 0.43 (9:1 petroleum ether / Et_2O); **IR** (thin film / ν_{max} / cm^{-1}) 2960, 2165, 1726, 1493, 1250, 836, 698; **^{1}H NMR** (400 MHz, $CDCl_3$) δ_H 9.76 (1H, s, H1), 7.23-7.19 (2H, m, ArH), 7.10-7.06 (3H, m, ArH), 5.45 (1H, dq, *J* = 15.3 and 6.2 Hz, H7), 5.29 (1H, ddd, *J* = 15.3, 8.2 and 1.3 Hz, H6), 2.63-2.55 (1H, m, H2), 2.52-2.44 (1H, m, H2), 2.23 (1H, ddd, *J* = 10.7, 7.0 and 4.3 Hz, H4), 2.17 (2H, s, SiC<u>H</u>$_2$Ph), 2.16-2.08 (1H, m, H5), 1.82 (1H, dddd, *J* = 13.4, 9.1, 6.5 and 4.3 Hz, H7), 1.66 (3H, d, *J* = 6.3 Hz, H8), 1.62-1.52 (1H, m, H7), 1.07 (3H, d, *J* = 6.8 Hz, Me), 0.12 (6H, s, Si(C<u>H</u>$_3$)$_2$Bn); **^{13}C NMR** (101 MHz, $CDCl_3$) δ_C 202.5, 139.4, 134.7, 128.5, 128.2, 125.4, 124.4, 109.7, 86.2, 42.2, 41.2, 38.4, 26.6, 25.1, 18.4, 18.1, –1.7; **HRMS** (ES^+) calc. for $C_{20}H_{28}NaOSi$ $[M+Na]^+$ 335.1802; 335.1795.

Benzyl((3*R*,4*R*,*E*)-3-(4,4-dibromobut-3-en-1-yl)-4-methylhept-5-en-1-yn-1-yl)dimethylsilane, S7

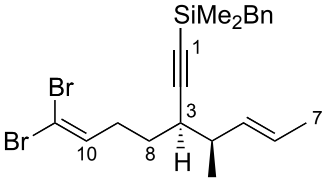

To a stirred solution of CBr_4 (565 mg, 1.70 mmol, 2.0 equiv.) in dry CH_2Cl_2 (4.2 mL) under Ar at 0 °C was added powdered PPh_3 (895 mg, 3.41 mmol, 4.0 equiv.). After stirring for a further 10 min, the reaction mixture was cooled to –30 °C and a solution of aldehyde **S6** (266 mg, 0.852 mmol, 1.0 equiv.) and triethylamine (1.2 mL, 8.52 mmol, 10 equiv.) in CH_2Cl_2 (4.3 mL) was added dropwise. The reaction mixture was stirred for 1 h, warming from –30 °C to 0 °C, before being quenched by addition of water. The layers were separated and the aqueous phase was extracted three times with CH_2Cl_2. The combined organic phases were dried ($MgSO_4$) and concentrated. The residue was purified by flash chromatography (49:1 petroleum ether / Et_2O) to yield vinyl dibromide **S7** (340 mg, 0.726 mmol, 85%) as a colourless oil. $[\alpha]_D^{25}$ +22.3 (*c* = 1.00, $CHCl_3$); R_f 0.83 (19:1 petroleum ether / Et_2O); **IR** (thin film, ν_{max} / cm^{-1}) 3025, 2960, 2167, 1601, 1493, 1250, 837; **^{1}H NMR** (400 MHz, $CDCl_3$) δ_H 7.24-7.22 (2H, m, ArH), 7.12-7.08 (3H, m, ArH), 6.41 (1H, t, *J* = 7.5 Hz, H10), 5.46 (1H, dq, *J* = 15.2 and 6.3 Hz, H6), 5.30 (1H, ddd, *J* = 15.2, 8.1 and 1.3 Hz, H5), 2.34-2.26 (1H, m, H9), 2.25-2.21 (1H, m, H4), 2.19 (2H, s, SiC<u>H</u>$_2$Ph), 2.8-2.10 (2H, m, H9 and H3), 1.68 (3H, dd, *J* = 6.3 and 1.5 Hz, H7), 1.61-1.53 (1H, m, H8), 1.49-1.40 (1H, m, H8), 1.06 (3H, d, *J* = 6.7 Hz, Me), 0.13 (6H, s, Si(C<u>H</u>$_3$)$_2$); **^{13}C NMR** (101 MHz, $CDCl_3$) δ_C 139.4, 138.4, 134.9, 128.5, 128.3, 125.3, 124.4, 109.8, 89.1, 85.9, 41.0, 38.6, 31.3, 30.8, 26.7, 18.3, 18.1, –1.8; **HRMS** (EI/FI) calc. for $C_{21}H_{28}Br_2Si$ 468.0307; found 468.0810.

Benzyl((*3R*,*4R*,*E*)-3-(but-3-ynyl)-4-methylhept-5-en-1-ynyl)dimethylsilane, 7

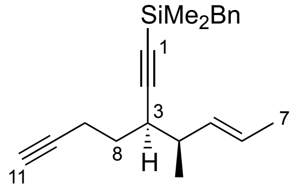

To a solution of vinyl dibromide **S7** (55.0 mg, 0.117 mmol, 1.0 equiv.) in THF (2.0 mL) at –78 °C was added *n*-BuLi (2.5 M in hexane, 105 µL, 0.258 mmol, 2.2 equiv.) dropwise. The mixture was warmed to rt, and stirred for 40 min, then quenched with NH_4Cl (sat., aq.). The layers were separated and the aqueous phase extracted three times with Et_2O. The combined organic layers were dried ($MgSO_4$) and concentrated. The residue was purified by flash chromatography (49:1 petroleum ether / Et_2O) to afford diyne **7** (35.6 mg, 0.115 mmol, 98%) as a colourless oil. $[\alpha]_D^{25}$ +98.0 (*c* = 1.00, $CHCl_3$); R_f 0.70 (19:1 petroleum ether / Et_2O); **IR** (thin film, ν_{max} / cm^{-1}) 2929, 2165, 1728, 1612, 1247, 1097, 828; **^{1}H NMR** (400 MHz, $CDCl_3$) δ_H 7.25-7.21 (2H, m, ArH), 7.11-7.08 (3H, m, ArH), 5.45 (1H, dq, *J* = 15.2 and 6.3 Hz, H6), 5.32 (1H, dd, *J* = 15.2 and 8.1 Hz, H5), 2.42-2.34 (2H, m, H3 and H9), 2.25 (1H, ddd, *J* = 16.7, 8.3 and 2.3 Hz, H9), 2.18 (2H, s, Si$\underline{C}H_2$Ph), 2.14 3 (1H, q, *J* = 7.1 Hz, H4), 1.97 (1H, t, *J* = 2.6 Hz, H11), 1.75-1.69 (1H, m, H8), 1.67 (3H, d, *J* = 6.3 Hz, H7), 1.58-1.49 (1H, m, H8), 1.07 (3H, d, *J* = 6.8 Hz, Me), 0.12 (6H, s, Si(C$\underline{H}_3$)$_2$); **^{13}C NMR** (101 MHz, $CDCl_3$) δ_C 139.4, 134.9, 128.5, 128.2, 125.2, 124.4, 109.6, 85.8, 84.4, 68.5, 40.8, 38.2, 31.8, 26.6, 18.2, 18.1, 16.8, –1.7; **HRMS** (ES^+) calc. for $C_{19}H_{28}NaSi$ $[M+Na]^+$ 331.1858; found 331.1860.

1.2.2 Synthesis of AB ring alkyne 8

AB ring alkyne **8** was prepared according to the following synthetic scheme:

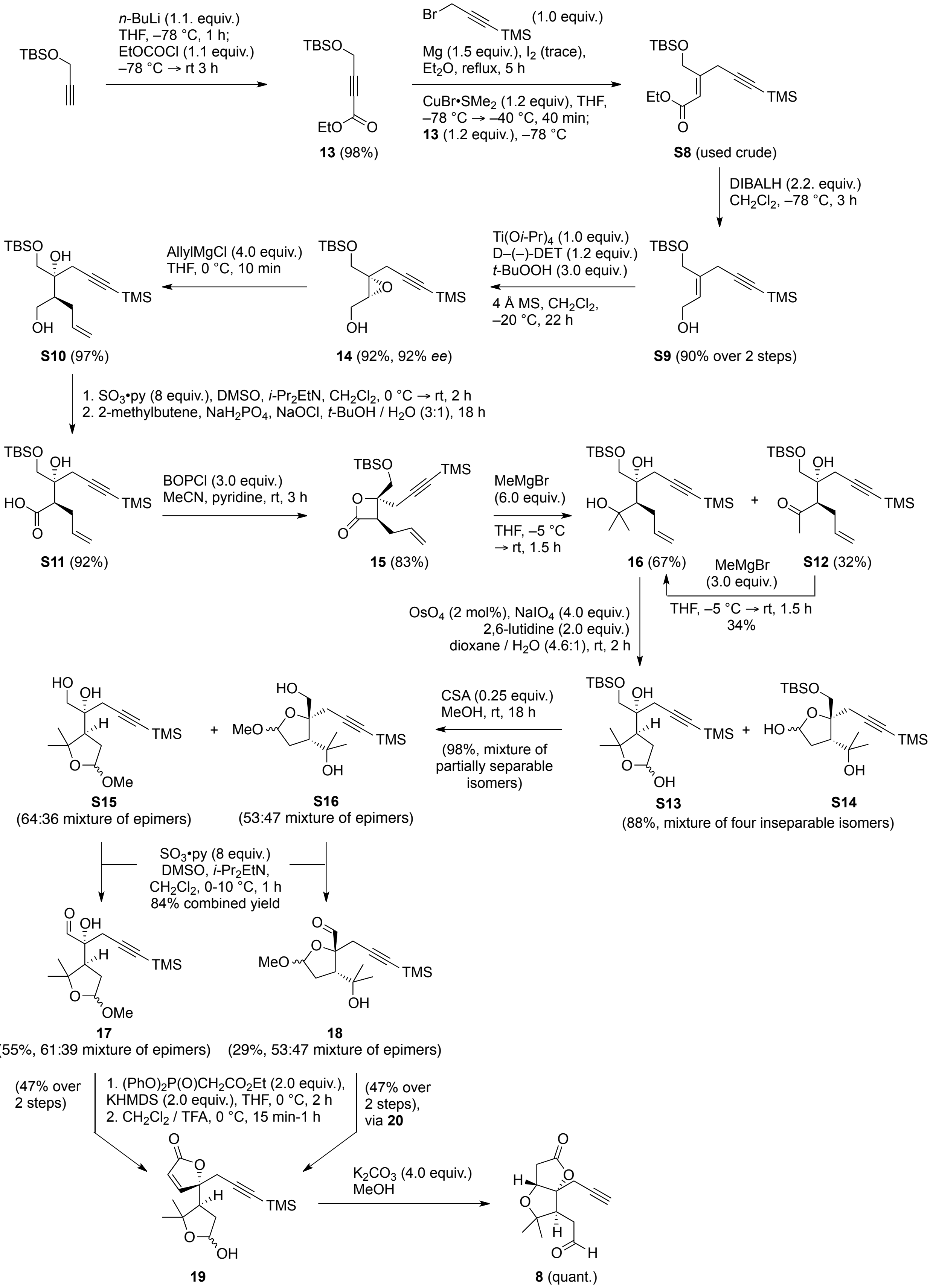

Ethyl 4-((*tert*-butyldimethylsilyl)oxy)but-2-ynoate, 13

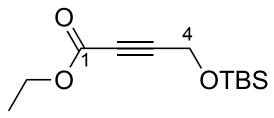

To a stirred solution of propargyl alcohol (5.5 mL, 93.1 mmol, 1.0 equiv.) in dry CH_2Cl_2 (370 mL) under argon at 0 °C was added imidazole (9.53 g, 140 mmol, 1.5 equiv.), DMAP (1.14 g, 3.579.31 mmol, 10 mol%) and TBSCl (15.5 g, 102 mmol, 1.1 equiv.). The reaction mixture was warmed to rt and stirred for 3 h before being quenched with $NaHCO_3$ (sat., aq.). The layers were separated and the aqueous phase extracted three times with Et_2O. The combined organic layers were dried (Na_2SO_4) and concentrated.

The crude *tert*-butyldimethyl(prop-2-yn-1-yloxy)silane (1.0 equiv.) was dissolved in dry THF (400 mL) under argon, and to this stirred solution at –78 °C was added *n*-BuLi (2.5M in hexanes, 37.2 mL, 102 mmol, 1.1 equiv.). After stirring the reaction mixture for 1 h at this temperature, ethyl chloroformate (9.9 mL, 102 mmol, 1.1 equiv.) was added dropwise. The reaction mixture was stirred for 3 h while slowly warming to RT, then the reaction was quenched by addition of NH_4Cl (sat., aq.). The layers were separated and the aqueous phase extracted three times with Et_2O. The combined organic layers were dried (Na_2SO_4) and concentrated. The crude product was purified by flash chromatography (9:1petroleum ether / Et_2O) to yield alkyne **13** (22.0 g, 90.8 mmol, 98%) as a colourless oil.

$\mathbf{R_f}$ 0.20 (20:1 petroleum ether / EtOAc); **^{1}H NMR** (400 MHz, $CDCl_3$) δ_H 4.42 (2H, s, H4), 4.23 (2H, q, *J* = 7.1 Hz, $C\underline{H}_2CH_3$), 1.30 (3H, t, *J* = 7.1, $CH_2C\underline{H}_3$), 0.90 (9H, s, $SiC(C\underline{H}_3)_3$), 0.13 (6H, s, $Si(C\underline{H}_3)_2$); **^{13}C NMR** (101 MHz, $CDCl_3$) δ_C 153.5, 85.8, 76.8, 62.2, 51.5, 25.9, 18.4, 14.1, -5.1; Spectroscopic data are identical to those reported in the literature.[5]

Ethyl (*Z*)-3-(((*tert*-butyldimethylsilyl)oxy)methyl)-6-(trimethylsilyl)hex-2-en-5-ynoate, S8

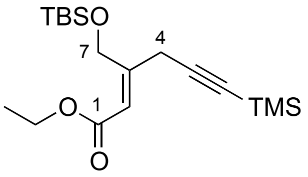

To an oven-dried two-necked flask equipped with a stirrer bar and a reflux condensor under Ar was added acid-washed magnesium turnings (1.79 mg, 73.7 mmol, 1.5 equiv.) and a few crystals of iodine. To the stirred mixture was simultaneously added dropwise diethyl ether (48 mL) and (3-bromoprop-1-yn-1-yl)trimethylsilane (9.39 g, 49.1 mmol, 1.0 equiv.) until reaction initiation was observed as evidenced by a gentle reflux. Dropwise addition continued to maintain this reflux; upon complete addition, the reaction mixture was refluxed for a further 5 h, then allowed to cool to rt. The solution was titrated against salicylaldehyde diphenylhydrazone[8] (29.1 mg) in THF (5 mL) to reveal a concentration of 0.38 M (18.1 mmol, 37%).

To a suspension of copper(I) bromide dimethyl sulfide complex (4.44 g, 21.6 mmol, 1.2 equiv.) in THF (70 mL) under Ar at –78 °C was added the above Grignard solution (0.38 M, 47.1 mL, 18.0 mmol, 1.0 equiv.) dropwise. The reaction mixture was stirred at –40 °C for 40 min before recooling to –78 °C, upon which alkyne **13** (5.24 mg, 21.6 mmol, 1.2 equiv.) was added. The reaction mixture was stirred overnight at –78 °C, before being quenched with NH_4Cl (sat. aq.). The layers were separated and the aqueous phase

extracted three times with Et_2O. The combined organic layers were dried (Na_2SO_4) and concentrated. The residue was passed through a short pad of silica (49:1 petroleum ether / Et_2O eluent) to yield the crude α,β-unsaturated ester **S1** as a colourless oil, which was used directly in the next step but could be purified by further chromatography for the purpose of characterization.

R$_f$ 0.45 (20:1 petroleum ether / EtOAc); **IR**: (thin film, ν_{max} / cm^{-1}) 2957, 2931, 2898, 2858, 2180, 1716, 1651, 1472, 1382, 1363, 1286, 1250, 1208, 1129, 1093, 1040, 1006, 939, 918, 836, 777, 760, 699, 670, 651; **^{1}H NMR** (400 MHz, $CDCl_3$) δ_H 6.07 (1H, app quin, J = 1.8 Hz, H2), 4.80-4.81 (2H, m, H7), 4.16 (2H, q, J = 7.1 Hz, OC$\underline{H}_2$CH$_3$), 3.31 (2H, br s, H4), 1.29 (3H, t, J = 7.1 Hz, OCH$_2$C$\underline{H}_3$), 0.89 (9H, s, OSiC(C$\underline{H}_3$)$_3$), 0.18 (9H, s, Si(C$\underline{H}_3$)), 0.07 (6H, s, Si(C$\underline{H}_3$)$_3$); **^{13}C NMR** (125 MHz, $CDCl_3$) δ_C 166.3, 157.2, 115.9, 102.4, 89.4, 61.8, 60.0, 26.0, 25.1, 18.4, 14.4, 0.2, –5.4; **HRMS** (ES$^+$) calc. for $C_{18}H_{34}NaO_3Si_2$ [M+Na]$^+$ 377.1939, found 377.1932.

(*Z*)-3-(((*tert*-Butyldimethylsilyl)oxy)methyl)-6-(trimethylsilyl)hex-2-en-5-yn-1-ol, S9

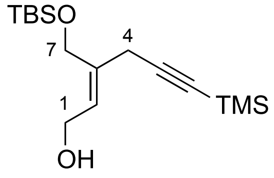

To a stirred solution of the crude α,β-unsaturated ester **S8** in dry CH_2Cl_2 (100 mL) under Ar at –78 °C was added DIBALH (1M in THF, 39.6 mL, 39.6 mmol, 2.2 equiv.) dropwise. The reaction mixture was stirred for 3 h at this temperature before being quenched by dropwise addition of water (1.6 mL), then 2M NaOH (1.6 mL). The reaction flask was placed in a 0 °C bath, then further water (3.9 mL) was added and the mixture was allowed to stir for 30 min. The layers were separated and the aqueous phase was extracted three times with Et_2O. The combined organic layers were dried ($MgSO_4$) and concentrated. The crude product was purified by flash chromatography (4:1 petroleum ether / Et_2O eluent) to yield alcohol **S9** (5.04 g, 16.1 mmol, 90% over 2 steps) as a white crystalline solid.

MP 41-43 °C; **R$_f$** 0.37 (4:1 petroleum ether / Et_2O); **IR** (thin film, ν_{max} / cm^{-1}) 3362, 2957, 2930, 2898, 2857, 2360, 2177, 1727, 1472, 1251, 1084, 1005, 840, 777, 760; **^{1}H NMR** (500 MHz, $CDCl_3$) δ_H 5.84 (1H, t, J = 6.8 Hz, H2), 4.23 (2H, s, H7), 4.21 (2H, app. t, J = 6.1 Hz, H1), 3.08 (2H, s, H4), 1.84 (1H, t, J = 5.8 Hz, OH), 0.90 (9H, s, OSiC(C$\underline{H}_3$)$_3$), 0.16 (9H, s, Si(C$\underline{H}_3$)$_3$), 0.08 (6H, s , OSi(C$\underline{H}_3$)$_2$); **^{13}C NMR** (125 MHz, $CDCl_3$) δ_C 137.1, 126.8, 103.6, 87.9, 60.8, 58.8, 26.0, 25.7, 18.4, 0.2, -5.3; **HRMS** (ES$^+$) calc. for $C_{16}H_{32}NaO_2Si_2$ [M+Na]$^+$ 335.1833; found 335.1827.

((2*R*,3*S*)-3-(((*tert*-Butyldimethylsilyl)oxy)methyl)-3-(3-(trimethylsilyl)prop-2-yn-1-yl)oxiran-2-yl)methanol, 14

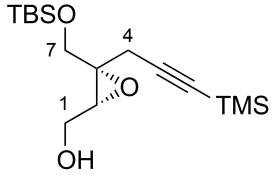

Activated 4 Å molecular sieves (1.66 g, 30 w/w%) were heated (heat gun) under vacuum for 5 min in a Schlenk tube and then cooled under argon. CH_2Cl_2 (53 mL) and Ti(O*i*-Pr)$_4$ (5.30 mL, 17.7 mmol, 1.0

equiv.) were added and the solution was cooled to –30 °C, then D-(–)-diethyl tartrate (3.85 mL, 21.2 mmol, 1.2 equiv.) was added dropwise. After stirring the reaction mixture for 30 min at the –30 °C, a solution of alkene **S9** (5.52 g, 17.7 mmol, 1.0 equiv.) in CH_2Cl_2 (19 mL) was added dropwise, followed again by stirring for 30 min at –30 °C. *Tert*-butyl hydroperoxide (5.5 M in hexanes, 9.65 mL, 53.1 mmol, 3.0 equiv.) was added dropwise and the reaction flask was placed in a freezer at –20 °C. After 22 h, the reaction was cooled to –30 °C and a solution of tartaric acid (7.95 g, 53.1 mmol, 3.0 equiv.) and iron(**II**) sulfate heptahydrate (44.2 g, 48.7 mmol, 9.0 equiv.) in water (54 mL) was poured in with vigorous stirring of the reaction mixture. After warming slowly to rt (~1 h), additional water was added to aid separation, then the layers were separated and the aqueous phase was extracted three times with CH_2Cl_2. The combined organic layers were dried (Na_2SO_4) and concentrated. Et_2O (35 mL) was added to the crude product, which was then cooled to 0 °C. A solution of sodium hydroxide in saturated sodium chloride solution (0.75 M, 42.4 mL) was added dropwise. After stirring for 1.5 h at 0 °C, water was added, the layers were separated and the aqueous phase was extracted three times with Et_2O (3 x 35 mL). The combined organic layers were dried (Na_2SO_4) and concentrated. The crude product was purified by flash chromatography (2:1 petroleum ether / EtOAc eluent) to afford **14** (5.36 g, 16.3 mmol, 92%) as a yellow oil. $[\alpha]_D^{25}$ +14.0 (*c* 1.0, $CHCl_3$); R_f 0.11 (9:1 Petroleum ether / EtOAc); **IR** (thin film, ν_{max} / cm^{-1}) 3433, 2957, 2931, 2360, 2179, 1472, 1251, 1099, 971, 841, 761, 669, 648; **^{1}H NMR** (500 MHz, $CDCl_3$) δ_H 3.98 (1H, d, *J* = 11.1 Hz, H7), 3.88-3.86 (1H, m, H1), 3.78-3.74 (1H, m, H1), 3.68 (1H, d, *J* = 11.1 Hz, H7), 3.28 (1H, t, *J* = 6.3 Hz, H2), 2.77 (1H, d, *J* = 17.5 Hz, H4), 2.59 (1H, d, *J* = 17.5 Hz, H4), 2.14-2.07 (1H, br s, OH), 0.91 (9H, s, $OSiC(CH_3)_3$), 0.15 (9H, s, $Si(CH_3)_3$), 0.11 (3H, $OSi(CH_3)_2$), 0.10 (3H, $OSi(CH_3)_2$); **^{13}C NMR** (125 MHz, $CDCl_3$) δ_C 101.0, 87.9, 63.4, 61.7, 61.2, 61.0, 26.0, 25.3, 18.4, 0.1, –5.3, –5.4; **HRMS** (ES^+) calc. for $C_{16}H_{32}NaO_3Si_2$ $[M+Na]^+$ 351.1782; found 351.1777.

Note: The assignment of absolute stereochemistry of this epoxide is made by analogy to the entiomeric epoxidation of the following epoxides, using (L)-(+)-DET; and by eventual conversion of this epoxide to the natural product.

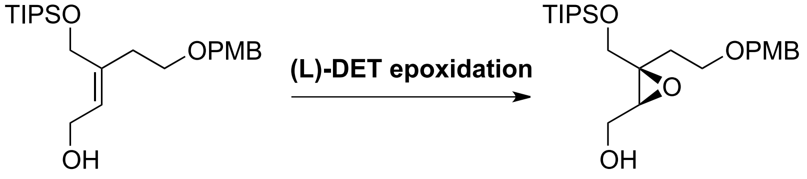

Reference: [6]

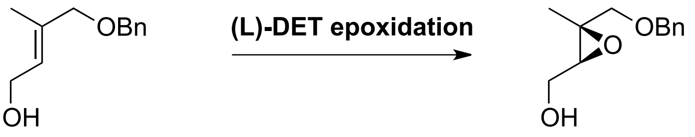

References: [7]

The *ee* of epoxide **14** was determined by conversion to the corresponding benzoate ester (see next page):

((2R,3S)-3-(((Tert-butyldimethylsilyl)oxy)methyl)-3-(3-(trimethylsilyl)prop-2-yn-1-yl)oxiran-2-yl)methyl benzoate

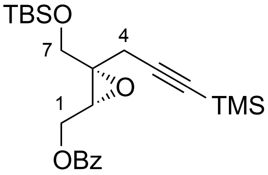

To a stirred solution of epoxide **14** (42.2 mg, 0.13 mmol, 1.0 equiv.) in CH_2Cl_2 (1.5 mL) under N_2 were added pyridine (32 μL, 0.39 mmol, 3.0 equiv.), DMAP (1.6 mg, 13 μmol, 0.1 equiv.) and benzoyl chloride (23 μL, 0.20 mmol, 1.5 equiv.). The reaction mixture was stirred at rt for 3 h, then MeOH (1 mL) was added. The reaction mixture was concentrated, and the resulting crude material was purified by flash chromatography (97:3 petroleum ether / EtOAc) to afford the title ester (49.8 mg, 89%) as a colourless oil; $[\alpha]_D^{25}$ +12.1 (*c* 1.0, MeOH); $\mathbf{R_f}$ 0.36 (97:3 petroleum ether / EtOAc); **IR** (thin film, ν_{max} / cm^{-1}) 2957, 2929, 2361, 2180, 1726, 1272, 1250, 1108, 780, 760, 711; ^{1}**H NMR** (400 MHz, $CDCl_3$) δ_H 8.08 (2H, m, HAr), 7.58 (1H, app. tt, *J* = 7.3 Hz and 1.2 Hz, ArH), 7.45 (2H, t, *J* = 7.8 Hz, ArH), 4.63 (1H, dd, *J* = 12.4 Hz and 4.0 Hz, H1), 4.38 (1H, dd, *J* = 12.4 Hz and 7.0 Hz, H1), 3.88 (1H, d, *J* = 11.2 Hz, H7), 3.85 (1H, d, *J* = 11.2 Hz, H7), 3.44 (1H, dd, *J* = 4.0 Hz and 7.0 Hz, H2), 2.79 (1H, d, *J* = 17.4 Hz, H4), 2.63 (1H, d, *J* = 17.4 Hz, H4), 0.91 (9H, s, $OSiC(CH_3)_3$), 0.15 (9H, s, $Si(CH_3)_3$), 0.10 (3H, s, $OSi(CH_3)_2$), 0.09 (3H, s, $OSi(CH_3)_2$); ^{13}**C NMR** (101 MHz, $CDCl_3$) δ_C 166.4, 133.4, 129.9 (2 C), 128.6, 101.1, 87.9, 63.1, 62.8, 61.9, 59.0, 26.0 (3 C), 24.8, 18.4, 0.1 (3 C), -5.3, -5.4; **HRMS** (ES^+) calc. for $C_{23}H_{36}NaO_4Si_2$ $[M+Na]^+$ 455.20443; found 455.20456.

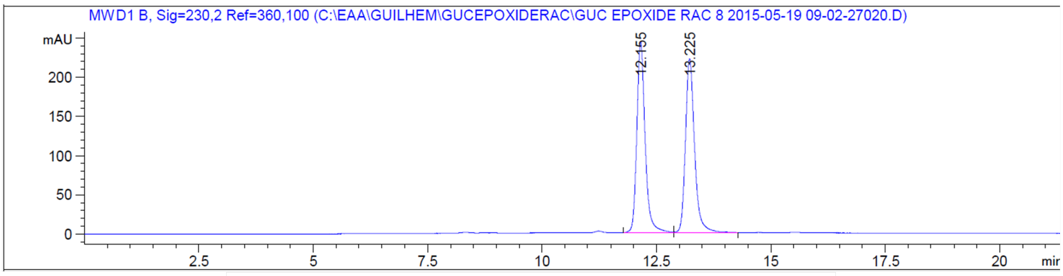

Peak #	RetTime [min]	Type	Width [min]	Area [mAU*s]	Height [mAU]	Area %
1	12.155	BV	0.1917	3090.04810	244.01199	49.9847
2	13.225	VB	0.2104	3091.93677	222.10698	50.0153
Totals :				6181.98486	466.11897	

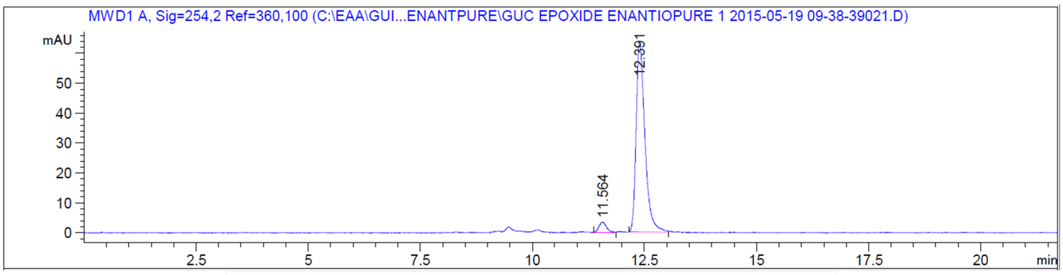

Peak #	RetTime [min]	Type	Width [min]	Area [mAU*s]	Height [mAU]	Area %
1	11.564	BB	0.1532	37.99422	3.40511	4.2005
2	12.391	BB	0.2061	866.52704	63.53046	95.7995
Totals :				904.52126	66.93558	

(2*S*,3*R*)-2-Allyl-3-(((*tert*-butyldimethylsilyl)oxy)methyl)-6-(trimethylsilyl)hex-5-yne-1,3-diol, S10

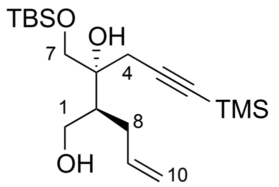

To a stirred solution of epoxide **14** (5.36 g, 16.3 mmol, 1.0 equiv.) in dry THF (150 mL) under argon at 0 °C was added allylmagnesium chloride (1.7 M in THF, 38.4 mL, 65.3 mmol, 4 equiv.) dropwise. The reaction mixture was stirred for 10 min, then it was quenched by addition of NH_4Cl (10 mL, sat., aq.). The suspension was filtered through a pad of celite, and the solvent was removed *in vacuo*. The crude product was purified by flash chromatography (4:1 Petroleum ether / EtOAc eluent) to give **S10** (5.85 g, 97%) as white solid. $[\alpha]_D^{25}$ –2.4 (*c* 0.79, $CHCl_3$); **MP** 56-58 °C; **R*f*** 0.35 (4:1 petroleum ether / EtOAc); **IR** (thin film, ν_{max} / cm^{-1}) 3735, 3386, 2956, 2930, 2858, 2360, 2175, 1944, 1640, 1418, 1251, 1100, 841; **^{1}H NMR** (500 MHz, $CDCl_3$) δ_H 5.81 (1H, dddd, *J* = 17.1, 10.1, 8.1 and 5.9 Hz, H9), 5.09 (1H, dq, *J* = 17.1 and 1.5 Hz, H10), 5.04 (1H, d, *J* = 10.1 Hz, H10), 3.85 (dd, *J* = 11.7 and 1.7 Hz, H1), 3.71 (1H, m, H1), 3.65 (2H, s, H7), 2.98 (1H, t, *J* = 5.6 Hz, OH), 2.91 (1H, s, OH), 2.63-2.55 (2H, AB q, H4), 2.29 (1H, ddddd, *J* = 10.1, 5.9, 4.0, 2.2 and 1.8 Hz, H8), 2.15-2.08 (1H, m, H8), 1.94 (1H, ddt, *J* = 10.2, 6.2 and 3.1 Hz, H2), 0.91 (9H, s, OSiMe$_2$*t*-Bu), 0.15 (9H, s, SiMe$_3$), 0.10 (6H, s, OSiMe$_2$*t*-Bu); **^{13}C NMR** (101 MHz, $CDCl_3$) δ_C 137.3, 116.4, 102.8, 88.2, 76.4, 65.4, 61.7, 44.6, 30.5, 28.4, 25.8, 18.2, 0.0, –5.4, –5.5; **HRMS** (ES$^+$) calc. for $C_{19}H_{38}NaO_3Si_2$ $[M+Na]^+$ 393.2252; found 393.2249.

(2*R*,3*R*)-2-Allyl-3-(((*tert*-butyldimethylsilyl)oxy)methyl)-3-hydroxy-6-(trimethylsilyl)hex-5-ynoic acid, S11

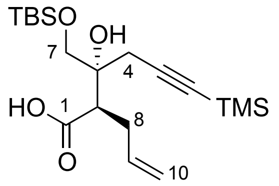

Dry DMSO (9.8 mL, 138 mmol, 42 equiv) was added to SO_3•py (4.27 g, 26.7 mmol, 8.1 equiv) under Ar, and the suspension was stirred at rt for 15 min. CH_2Cl_2 (70 mL) was added, then the mixture was cooled to 0 °C and stirred for a further 10 min. A solution of **S10** (1.22 g, 3.29 mmol, 1.0 equiv.) and *N*,*N*-diisopropylethylamine (11.3 mL, 67.5 mmol, 20.5 equiv.) in dry CH_2Cl_2 (70 mL) was added, and the resulting mixture was warmed to rt and stirred for 2 h, before being quenched by addition of NH_4Cl (sat., aq.). CH_2Cl_2 was added, the layers were separated, and the organic layer was washed with brine. The organic layer was dried ($MgSO_4$) and concentrated to the corresponding aldehyde, which was directly used in the subsequent Pinnick oxidation.

To a stirred solution of this crude aldehyde (3.29 mmol, 1.0 equiv.) in wet *t*-BuOH (120 mL) was added 2-methyl-2-butene (12.1 mL, 98.7 mmol, 30 equiv.). To the reaction mixture was added a solution of sodium chlorite (80%, 3.00 g, 32.9 mmol, 10 equiv.) and $NaH_2PO_4.2H_2O$ (4.16 g, 26.3 mmol, 8.0 equiv.) in water (43 mL). After stirring overnight, brine was added, the layers were separated, and the aqueous phase was

extracted four times with EtOAc. The combined organic layers were dried (Na_2SO_4) and concentrated. The crude product was purified by flash chromatography (1:1 petroleum ether / EtOAc eluent) to give carboxylic acid **S11** (1.17 g, 3.04 mmol, 92%) as a colourless highly viscous oil. **$[\alpha]_D^{25}$** –5.7 (*c* 1.00, $CHCl_3$); **MP** 89 °C; **R_f** 0.22 (2:1 petroleum ether / EtOAc); **IR** (thin film, ν_{max} / cm^{-1}) 3310, 2956, 2177, 1699, 1423, 125, 1190, 1130, 1074, 920, 841, 779, 760; **^{1}H NMR** (400 MHz, $CDCl_3$) δ_H 10.30 (1H, br s, COOH), 5.79 (1H, ddt, *J* = 17.0, 10.1 and 7.0 Hz, H9), 5.11 (1H, dd, *J* = 17.0 and 1.6 Hz, H10), 5.05 (1H, dd, *J* = 10.1 and 1.6 Hz, H10), 3.73 (1H, d, *J* = 10.0 Hz, H7), 3.64 (1H, d, *J* = 10.0 Hz, H7), 2.88 (1H, dd, *J* = 8.2 and 6.6 Hz, H2), 2.68 (1H, d, *J* = 17.1 Hz, H8), 2.55 (1H, d, *J* = 17.1 Hz, H8), 2.45 (2H, br t, *J* = 7.5 Hz, H4), 2.10 (1H, s, OH), 0.90 (9H, s, $OSiC(CH_3)_3$), 0.16 (9H, s, $Si(CH_3)_3$), 0.09 (3H, s, $OSi(CH_3)_2$), 0.09 (3H, s, $OSi(CH_3)_2$); **^{13}C NMR** (126 MHz, $CDCl_3$) δ_C 176.5, 135.4, 117.3, 101.8, 88.9, 74.2, 65.2, 50.7, 31.3, 28.4, 25.9, 18.4, 0.1, –5.4; **HRMS** (ES^+) calc. for $C_{19}H_{36}NaO_4Si_2$ $[M+Na]^+$ 407.2044; found 407.2040.

(3*R*,4*R*)-3-Allyl-4-(((*tert*-butyldimethylsilyl)oxy)methyl)-4-(3-(trimethylsilyl)prop-2-yn-1-yl)oxetan-2-one, 15

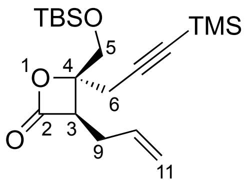

To a stirred solution of carboxylic acid **S11** (500 mg, 1.30 mmol, 1.0 equiv.) in dry MeCN (11 mL) under Ar was added dry pyridine (8.0 mL) and bis(2-oxo-3-oxazolidinyl)phosphonic chloride (BOPCl) (992 mg, 3.90 mmol, 3.0 equiv.). The reaction mixture was stirred for 3 h before being quenched with water. The layers were separated and the aqueous phase extracted four times with ethyl acetate. The combined organic layers were dried (Na_2SO_4) and concentrated. The crude product was purified by flash chromatography (19:1 petroleum ether / Et_2O eluent) to yield β-lactone **15** (394 mg, 1.07 mmol, 83%) as a colourless oil. **$[\alpha]_D^{25}$** +5.8 (*c* 1.00, $CHCl_3$); **R_f** 0.58 (9:1 petroleum ether / Et_2O); **IR** (thin film, ν_{max} / cm^{-1}) 2958, 2929, 2857, 2360, 2180, 1833, 1462, 1251, 1111, 1009, 841, 779, 760; **^{1}H NMR** (400 MHz, $CDCl_3$) δ_H 5.84 (1H, ddt, *J* = 17.0, 10.3 and 6.5 Hz, H10), 5.15 (1H, dq, *J* = 17.0 and 1.5 Hz, H11), 5.10 (1H, dq, *J* = 10.3 and 1.5 Hz, H11), 3.95 (2 H, app d, *J* = 0.7 Hz, H5), 3.66 (1H, app t, *J* = 8.2 Hz, H3), 2.85 (1H, d, *J* = 17.4 Hz, H6), 2.73 (1H, d, *J* = 17.4 Hz, H6), 2.64-2.57 (2H, m, H9), 0.90 (9H, s, $OSiC(CH_3)_3$), 0.16 (9H, s, $Si(CH_3)_3$), 0.09 (6H, s, $OSi(CH_3)_2$); **^{13}C NMR** (101 MHz, $CDCl_3$) δ_C 169.8, 134.5, 117.2, 99.6, 89.1, 80.4, 63.4, 55.5, 28.4, 27.3, 25.9, 18.4, 0.0, –5.4, –5.5; **HRMS** (ES^+) calc. for $C_{19}H_{34}NaO_3Si_2$ $[M+Na]^+$ 389.1939; found 389.1924.

(3*S*,4*R*)-3-Allyl-4-(((*tert*-butyldimethylsilyl)oxy)methyl)-2-methyl-7-(trimethylsilyl)hept-6-yne-2,4-diol, 16 and (3*R*,4*R*)-3-Allyl-4-(((*tert*-butyldimethylsilyl)oxy)methyl)-4-hydroxy-7-(trimethylsilyl) hept-6-yn-2-one, S12

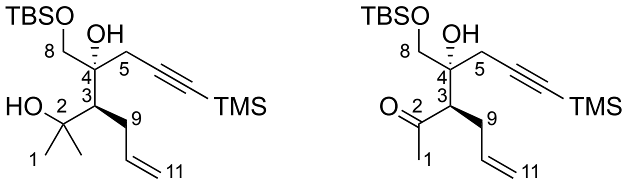

16 **S12**

To a stirred solution of β-lactone **15** (555 mg, 1.51 mmol, 1.0 equiv.) in THF (15 mL) under Ar at –5 °C was added methylmagnesium bromide (3 M in Et_2O, 3.0 mL, 9.00 mmol, 6.0 equiv.) dropwise. The reaction mixture was warmed slowly to rt over 1.5 h before being quenched with NH_4Cl (sat., aq.). The layers were separated and the aqueous phase was extracted four times with ethyl acetate. The combined organic layers were dried (Na_2SO_4) and concentrated. The crude product was purified by flash chromatography (95:5 petroleum ether / EtOAc eluent) to afford ketone **S12** (177 mg, 0.48 mmol, 31%) and tertiary alcohol **16** (383.5 mg, 0.96 mmol, 64%) as colourless oils. **S12** could be partly converted to **16** using the following representative procedure, which increased the overall yield for the formation of **16** to 75%.

Conversion of S12 to 16: To a stirred solution of **S12** (177 mg, 0.48 mmol, 1.0 equiv.) in THF (5 mL) under Ar at –5 °C was added methylmagnesium bromide (3 M in Et_2O, 0.46 mL, 1.38 mmol, 3.0 equiv.) dropwise. The reaction mixture was warmed slowly to rt over 1.5 h before being quenched with NH_4Cl (15 mL, sat., aq.,). The layers were separated and the aqueous phase was extracted three times with ethyl acetate. The combined organic layers were dried (Na_2SO_4) and concentrated. The crude product was purified by flash chromatography (95:5 petroleum ether / EtOAc) to afford alcohol **16** (65 mg, 0.16 mmol, 34%) as a colourless oil.

Data for 16: $[\alpha]_D^{25}$ +10.0 (*c* 1.01, $CHCl_3$); R_f 0.26 (4:1 Petroleum ether / Et_2O); **IR** (thin film, ν_{max} / cm^{-1}) 3390, 2956, 2930, 2857, 2176, 1464, 1251, 1096, 841, 779; ^{1}**H NMR** (400 MHz, $CDCl_3$) δ_H 5.84 (1H, ddt, *J* = 17.1, 10.3 and 6.6 Hz, H10), 5.01 (1H, dq, *J* = 17.1 and 1.7 Hz, H11), 4.97 (1H, dq, *J* = 10.3 and 1.7 Hz, H11), 4.22 (1H, br s, OH), 3.77 (1H, d, *J* = 10.0 Hz, H8), 3.73 (1H, d, *J* = 10.0 Hz, H8), 3.65 (1H, br s, OH), 2.66 (1H, d, *J* = 16.9 Hz, H5), 2.53 (1H, d, *J* = 16.9 Hz, H5), 2.29 (1H, dddt, *J* = 15.4, 6.6, 5.4, 1.7 Hz and H9), 2.20 (1H, t, *J* = 5.4 Hz, H3), 2.10 (1H, dddt, *J* = 15.4, 6.6, 5.4 and 1.7 Hz, H9), 1.29 (3H, s, H1), 1.28 (3H, s, H1'), 0.92 (9H, s, OSiC(C$\underline{H}_3$)$_3$), 0.16 (9H, s, Si(C$\underline{H}_3$)$_3$), 0.10 (6H, s, OSi(C$\underline{H}_3$)$_2$); ^{13}**C NMR** (126 MHz, $CDCl_3$) δ_C 140.2, 114.9, 103.7, 88.3, 77.5, 74.9, 66.0, 53.3, 32.6, 32.4, 31.3, 27.0, 26.0, 18.4, 0.2, –5.3. **HRMS** (ES^+) calc. for $C_{21}H_{42}NaO_3Si_2$ $[M+Na]^+$ 421.2565; found 421.2553.

Data for S12: $[\alpha]_D^{25}$ +23.4 (*c* 1.00, $CHCl_3$); R_f 0.38 (9:1 petroleum ether / Et_2O); **IR** (thin film, ν_{max} / cm^{-1}) 2957, 2857, 2173, 1716, 1638, 1472, 1361, 1251, 1106, 842, 778, 669; ^{1}**H NMR** (400 MHz, $CDCl_3$) δ_H 5.70 (1H, ddt, *J* = 17.0, 10.1 and 7.1Hz, H10), 5.05 (1H, dq, *J* = 17.0, 1.5 Hz and H11), 5.01 (1H, ddt, *J* = 10.1, 1.5 and 0.7 Hz), 3.65 (1H, d, *J* = 10.0 Hz, H8), 3.57 (1H, d, *J* = 10.0 Hz, H8), 3.12 (1H, dd, *J* = 8.6 and 6.6 Hz, H3), 3.10 (1H, s, OH), 2.57 (1H, d, *J* = 17.1 Hz, H5), 2.51 (1H, d, *J* = 17.1 Hz, H5), 2.39-2.34 (2H, m, H9), 2.23 (3H, s, H1), 0.90 (9H, s, OSiC(C$\underline{H}_3$)$_3$), 0.16 (9H, s, Si(C$\underline{H}_3$)$_3$), 0.08 (6H, s, OSi(C$\underline{H}_3$)$_2$);

^{13}**C NMR** (101 MHz, $CDCl_3$) δ_C 213.9, 135.7, 117.2, 102.9, 88.5, 74.7, 65.6, 55.5, 34.2, 31.7, 28.8, 26.0, 18.4, 0.2, –5.3, –5.4; **HRMS** (ES^+) calc. for $C_{20}H_{38}NaO_3Si_2$ $[M+Na]^+$ 405.2252, found 405.2238.

(4*R*)-4-((*R*)-1-((*tert*-Butyldimethylsilyl)oxy)-2-hydroxy-5-(trimethylsilyl)pent-4-yn-2-yl)-5,5-dimethyltetrahydrofuran-2-ol S13 and (4*S*,5*R*)-5-((((*tert*-butyldimethylsilyl) oxy)methyl)-4-(2-hydroxypropan-2-yl)-5-(3-(trimethylsilyl)prop-2-yn-1-yl)tetrahydrofuran -2-ol S14

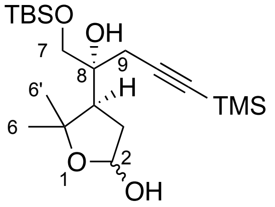

S13

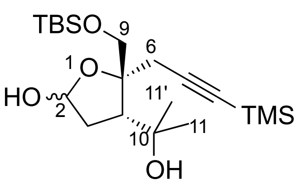

S14

To a stirred solution of alkene **16** (88.4 mg, 0.222 mol, 1.0 equiv.) in 1,4-dioxane (2.3 mL) and water (0.5 mL) was sequentially added 2,6-lutidine (51 μL, 0.443 mmol, 2.0 equiv.), OsO_4 (2.5 wt% in *t*-BuOH, 58 μL, 0.0044 mmol, 0.02 equiv.) and $NaIO_4$ (189 mg, 0.887 mmol, 4.0 equiv.). The reaction mixture was stirred for 2 h at rt, then it was diluted with water and Et_2O. The layers were separated and the aqueous phase was extract three times with Et_2O. The combined organic phases were dried ($MgSO_4$) and concentrated. The crude product was filtered through a short plug of silica (4:1 petroleum ether / Et_2O eluent), then concentrated to give lactols **S13** and **S14** as a 40:27:22:11 inseparable mixture of isomers, as a colourless oil (78.1 mg, 0.194 mmol, 88%). $\mathbf{R_f}$ 0.15 (7:3 petroleum ether / Et_2O); **IR** (thin film, ν_{max} / cm^{-1}) 3420, 2956, 2930, 2857, 2176, 1464, 1251, 1096, 841, 779; ^{1}**H NMR** (500 MHz, $CDCl_3$) δ_H 5.41 (1H, d, *J* = 5.1 Hz, H2), 3.73 (1H, d, *J* = 9.3 Hz, H7), 3.45 (1H, d, *J* = 9.3 Hz, H7), 2.65 (1H, d, *J* = 16.8 Hz, H9), 2.55 (1H, dd, *J* = 13.0 and 6.7 Hz, H4), 2.50 (1H, d, *J* = 16.8 Hz, H9), 2.31 (1H, app td, *J* = 12.8 and 5.1 Hz, H3), 1.88 (1H, dd, *J* = 12.6, 6.7 Hz, H3), 1.53 (3H, s, H6), 1.26 (3H, s, H6), 0.92 (9H, s, $OSiC(CH_3)_3$), 0.15 (3H, s, $OSi(CH_3)_2$), 0.14 (12H, app br s, $OSi(CH_3)_2$ and $Si(CH_3)_3$); ^{13}**C NMR** (126 MHz, $CDCl_3$) δ_C 104.1, 103.4, 96.6, 96.1, 95.6, 88.1, 88.1, 86.5, 84.7, 83.4, 73.8, 73.7, 70.6, 69.2, 67.1, 66.7, 50.5, 50.1, 47.2, 37.6, 35.9, 35.1, 32.8, 32.5, 31.0, 28.3, 28.2, 27.9, 26.2, 26.0, 26.0, 25.9, 25.8, 25.5, 18.4, 18.4, 0.1, –5.3, –5.4; **HRMS** (ES^+) calc. for $C_{20}H_{40}NaO_4Si_2$ $[M+Na]^+$ 423.2357; found 423.2347.

(2*R*)-2-((3*R*)-5-methoxy-2,2-dimethyltetrahydrofuran-3-yl)-5-(trimethylsilyl)pent-4-yne-1,2-diol S15 and 2-((2*R*,3*S*)-2-(hydroxymethyl)-5-methoxy-2-(3-(trimethylsilyl)prop-2-yn-1-yl)tetrahydrofuran-3-yl)propan-2-ol S16

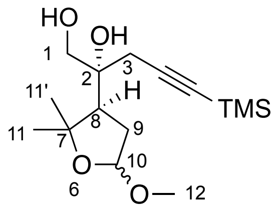

S15

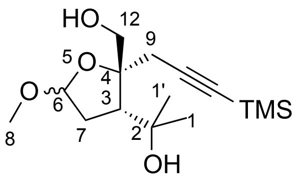

S16

To a stirred solution of lactols **S13** and **S14** (73.4 mg, 0.18 mmol, 1.0 equiv) in methanol (2.6 mL) was

added CSA (10.5 mg, 0.045 mmol, 0.25 equiv). The reaction mixture was stirred overnight, then it was quenched by addition of $NaHCO_3$ (10 mL, sat., aq.,). Et_2O (20 mL) was added to the solution, the layers were separated and the aqueous phase was extracted with Et_2O (3 x 20 mL). The combined organic phases were dried ($MgSO_4$) and concentrated. The crude product was purified by flash chromatography (7:3 petroleum ether / EtOAc eluent) to give a mixture of diols **S15** and **S16** (53 mg, 0.175 mmol, 98%) as a white solid. These diols were generally not readily separated by chromatography, and they were generally carried forward to the (separable) aldehydes **17** and **18**; the data presented below was obtained for the purpose of characterization by careful chromatography.

Data for S15 (64:36 mixture of epimers): **IR** (thin film, ν_{max} / cm^{-1}) 3436, 2958, 2928, 2175, 1467, 1250, 1105, 1051, 1033; **HRMS** (ES^+) calc. for $C_{15}H_{28}NaO_4Si$ $[M+Na]^+$ 323.1649; found 323.1641

Major epimer: $\mathbf{R_f}$ 0.23 (7:3 petroleum ether / EtOAc); ^{1}**H NMR** (500 MHz, $CDCl_3$) δ_H 4.88 (1H, d, *J* = 5.0 Hz, H10), 3.68 (1H, dd, *J* = 10.9 and 5.5 Hz, H1), 3.58 (1H, dd, *J* = 10.9 and 6.5 Hz, H1), 3.33 (3H, s, H12), 2.64 (2H, app d, *J* = 2.8 Hz, H3), 2.48 (1H, dd, *J* = 12.6 and 6.6 Hz, H8), 2.43 (1H, s, OH), 2.21 (1H, td, *J* = 12.6 and 5.0 Hz, H9), 2.07 (1H, t, *J* = 6.3 Hz, OH), 1.90 (1H, dd, *J* = 12.6 and 6.6 Hz, H9), 1.47 (3H, s, H11), 1.30 (3H, s, H11'), 0.16 (9H, s, $Si(CH_3)_3$); ^{13}**C NMR** (126 MHz, $CDCl_3$) δ_C 102.8, 102.0, 89.3, 83.9, 73.4, 67.4, 54.2, 48.8, 34.5, 32.5, 28.6, 25.7, 0.1.

Minor epimer: $\mathbf{R_f}$ 0.33 (7:3 petroleum ether / EtOAc); ^{1}**H NMR** (500 MHz, $CDCl_3$) δ_H 4.95 (1H, dd, *J* = 6.0 and 3.8 Hz, H10), 3.61 (2H, app t, *J* = 5.8 Hz, H1), 3.36 (3H, s, H12), 2.78 (1H, s, OH), 2.64 (2H, app d, *J* = 0.9 Hz, H3), 2.32 (1H, td, *J* = 8.8 and 6.0 Hz, H9), 2.29 (1H, dd, *J* = 11.0 and 8.8 Hz, H8), 2.14-2.09 (1H, m, H9), 1.43 (3H, s, H11), 1.41 (3H, s, H11'), 0.15 (9H, s, $Si(CH_3)_3$); ^{13}**C NMR** (126 MHz, $CDCl_3$) δ_C 103.3, 103.0, 89.2, 83.4, 73.6, 67.4, 55.2, 50.9, 34.7, 31.0, 28.8, 25.6, 0.1.

Data for S16 (53:47 mixture of epimers): $\mathbf{R_f}$ 0.18 (7:3 petroleum ether / EtOAc); **IR** (thin film, ν_{max} / cm^{-1}) 3414, 2957, 2923, 2179, 1463, 1250, 1104, 1046, 843; **HRMS** (ES^+) calc. for $C_{15}H_{28}NaO_4Si$ $[M+Na]^+$ 323.1649; found 323.1637.

Major epimer: ^{1}**H NMR** (500 MHz, $CDCl_3$) δ_H 4.97 (1H, d, *J* = 5.5 Hz, H6), 3.90 (1H, d, *J* = 11.7 Hz, H12), 3.78 (1H, d, *J* = 12.4 Hz, H12), 3.38 (3H, s, H8), 2.71 (1H, d, *J* = 17.4 Hz, H9), 2.64 (1H, d, *J* = 17.4 Hz, H9), 2.63 (1H, app dd, *J* = 13.8 and 7.2 Hz, H3), 2.42-2.37 (1H, m, H7), 2.33 (1H, br s, OH), 2.03 (1H, ddd, *J* = 17.0, 15.4 and 4.5 Hz, H7), 1.38 (3H, s, H1), 1.26 (3H, s, H1), 0.15 (9H, s, $Si(CH_3)_3$); ^{13}**C NMR** (126 MHz, $CDCl_3$) δ_C 104.3, 103.6, 88.6, 85.3, 70.6, 66.6, 55.1, 49.3, 35.3, 31.0, 29.6, 25.9, 0.1.

Minor epimer: ^{1}**H NMR** (500 MHz, $CDCl_3$) δ_H 4.98 (1H, d, *J* = 5.1 Hz, H6), 3.73 (1H, d, *J* = 11.4 Hz, H12), 3.65 (1H, d, *J* = 11.5 Hz, H12), 3.40 (3H, s, H8), 2.83 (1H, d, *J* = 17.2 Hz, H9), 2.75 (1H, d, *J* = 17.2 Hz, H9), 2.40-2.35 (1H, m, H3), 2.33 (1H, br s, OH), 2.27 (1H, ddd, *J* = 13.7, 12.5 and 5.1 Hz, H7), 1.97 (1H, dd, *J* = 12.5 and 6.7 Hz, H7), 1.39 (3H, s, H1), 1.32 (3H, s, H1), 0.14 (9H, s, $Si(CH_3)_3$); ^{13}**C NMR** (126 MHz, $CDCl_3$) δ_C 104.5, 103.0, 88.0, 87.6, 70.8, 68.7, 55.9, 51.8, 35.7, 31.3, 29.9, 25.6, 0.0.

(2*R*)-2-hydroxy-2-((3*R*)-5-methoxy-2,2-dimethyltetrahydrofuran-3-yl)-5-(trimethylsilyl)pent-4-ynal, 17 and (2*R*,3*S*)-3-(2-hydroxypropan-2-yl)-5-methoxy-2-(3-(trimethylsilyl)prop-2-yn-1-yl)tetrahydrofuran-2-carbaldehyde 18

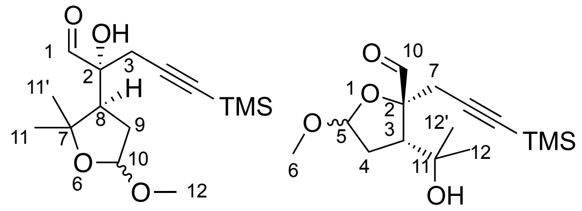

Dry DMSO (1.02 mL, 14.3 mmol) was added to SO_3•py (438 mg, 2.75 mmol, 8.1 equiv.) under Ar, and the suspension was stirred for 15 min at rt. CH_2Cl_2 (5.5 mL) was added, then the mixture was cooled to 0 °C and stirred for a further 10 min. A solution of diols **S15** and **S16** (101 mg, 0.34 mmol, 1.0 equiv.) and *N*,*N*-diisopropylethylamine (1.00 mL, 1.85 mmol) in CH_2Cl_2 (5.5 mL) was added, and the resulting mixture stirred between 0 °C and 10 °C for 1 h before being quenched by addition of NH_4Cl (10 mL, sat., aq.). Et_2O was added (~30 mL), the layers were separated, and the organic layer was washed sequentially with $NaHCO_3$ (10 mL, sat., aq.), followed by brine (10 mL). The organic layer was dried ($MgSO_4$) and concentrated. The product was purified by flash chromatography on a short plug of silica (8:2 petroleum ether / Et_2O eluent), to yield the separable aldehydes **17** (56.2 mg, 0.19 mmol, 55%, 61:39 mixture of epimers) and **18** (29.0 mg, 0.097 mmol, 29%, 53:47 mixture of epimers), both as a colourless oils;

Data for 17 (61:39 mixture of epimers): $\mathbf{R_f}$ 0.44 (4:1 petroleum ether / Et_2O); **IR** (thin film, ν_{max} / cm^{-1}) 3420, 2958, 2920, 2179, 1732, 1370, 1250, 1105, 1045), 977, 844, 760; **HRMS** (ES^+) calc. for $C_{15}H_{26}NaO_4Si$ $[M+Na]^+$ 321.1493; found 321.1490.

Major epimer: ^{1}H NMR (500 MHz, $CDCl_3$) δ_H 9.69 (1H, d, J = 0.9 Hz, H1), 4.87 (1H, d, J = 4.8 Hz, H10), 3.48 (1H, d, J = 0.9 Hz, OH), 3.32 (3H, s, H12), 2.78 (1H, d, J = 17.0 Hz, H3), 2.77 (1H, d, J = 13.0, 6.6 Hz, H8), 2.74 (1H, d, J = 17.0 Hz, H3), 2.09 (1H, td, J = 12.8 and 4.8 Hz, H9), 1.73 (1H, dd, J = 12.6 and 6.6 Hz, H9), 1.50 (3H, s, H11), 1.43 (3H, s, H11'); **^{13}C NMR** (126 MHz, $CDCl_3$) δ_C 202.2, 102.2, 99.5, 90.5, 83.8, 78.4, 54.2, 49.0, 34.2, 32.6, 30.8, 28.6, 0.0.

Minor epimer: ^{1}H NMR (500 MHz, $CDCl_3$) δ_H 9.73 (1H, d, J = 0.7 Hz, H1), 4.97 (1H, dd, J = 6.1 and 3.8 Hz, H10), 3.71 (1H, d, J = 0.7 Hz, OH), 3.34 (3H, s, H12), 2.61 (1H, d, J = 12.0 Hz, H3), 2.57 (1H, d, J = 12.0 Hz, H3), 2.51 (1H, dd, J = 10.8 and 9.4 Hz, H8), 2.24 (1H, ddd, J = 13.6, 9.4 and 6.1 Hz, H9), 2.02 (1H, ddd, J = 13.6, 10.8 and 3.8 Hz, H9), 1.31 (3H, s, H11), 1.22 (3H, s, H11'); **^{13}C NMR** (126 MHz, $CDCl_3$) δ_C 202.2, 103.4, 99.6, 90.4, 83.2, 78.4, 55.3, 51.0, 34.5, 28.7, 25.8, 25.5, 0.0.

Data for 18 (53:47 mixture of epimers): **IR** (thin film, ν_{max} / cm^{-1}) 3458, 2959, 2179, 1740, 1211, 1043, 842; **HRMS** (ES^+) calc. for $C_{15}H_{26}NaO_4Si$ $[M+Na]^+$ 321.1493; found 321.1496.

Major epimer: $\mathbf{R_f}$ 0.26 (4:1 petroleum ether / EtOAc); **^{1}H NMR** (500 MHz, $CDCl_3$) δ_H 9.53 (1H, s, H10), 5.16 (1H, dd, J = 6.0 and 4.2 Hz, H5), 3.47 (3H, s, H6), 3.05 (1H, d, J = 17.0 Hz, H7), 2.97 (1H, d, J = 17.0 Hz, H7), 2.39 (1H, ddd, J = 12.5, 9.1 and 6.0 Hz, H4), 2.34 (1H, dd, J = 9.9 and 9.1 Hz, H3), 2.12 (1H, ddd, J = 12.5, 9.9 and 4.2 Hz, H4), 1.95 (1H, s, OH), 1.25 (3H, s, H12), 1.25 (3H, s, H12), 0.12 (9H, s, $Si(CH_3)_3$); **^{13}C NMR** (126 MHz, $CDCl_3$) δ_C 200.3, 105.1, 102.9, 87.7, 87.4, 70.3, 56.2, 54.2, 34.5, 30.7, 30.6, 24.0, 0.1.

Minor epimer: **R_f** 0.34 (4:1 petroleum ether / EtOAc); **^{1}H NMR** (500 MHz, $CDCl_3$) δ_H 9.39 (1H, s, H10), 5.18 (1H, d, J = 5.0 Hz, H5), 3.42 (3H, s, H6), 2.86 (1H, d, J = 17.3 Hz, H7), 2.82 (1H, d, J = 17.3 Hz, H7), 2.64 (1H, dd, J = 13.1, 6.8 Hz, H3), 2.37 (1H, ddd, J = 13.1, 12.1 and 5.0 Hz, H4), 2.02 (1H, dd, J = 12.1 and 6.8 Hz, H4), 1.98 (1H, s, OH), 1.30 (3H, s, H12), 1.28 (3H, s, H12), 0.15 (9H, s, $Si(CH_3)_3$); **^{13}C NMR** (126 MHz, $CDCl_3$) δ_C 200.6, 104.5, 103.4, 88.5, 88.1, 70.9, 55.1, 50.5, 35.2, 30.6, 30.4, 23.6, 0.0.

(5*S*)-5-((3*S*)-5-hydroxy-2,2-dimethyltetrahydrofuran-3-yl)-5-(3-(trimethylsilyl)prop-2-yn-1-yl)furan-2(5*H*)-one, 19

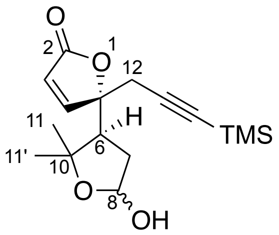

From **17**: To a stirred solution of ethyl 2-(diphenoxyphosphoryl)acetate (34.7 mg, 0.108 mmol, 2.0 equiv.) in THF (1 mL) under Ar at 0 °C was added KHMDS (0.5 M in toluene, 0.2 mL, 0.102 mmol, 1.9 equiv.) and the resulting mixture was stirred at 0 °C for 20 min before being added dropwise to a solution of aldehyde **17** (16.1 mg, 0.054 mmol, 1.0 equiv.) in THF (0.64 mL). The reaction mixture was stirred between 0 °C and 10 °C for 2 h and was then quenched by addition of NH_4Cl (sat., aq., 5 mL). Et_2O was added (10 mL), the layers were separated, and the aqueous phase was extracted two times with Et_2O (2x10 mL). The combined organic layers were dried ($MgSO_4$) and concentrated. The crude product was purified by flash chromatography on a short plug of silica (8:2 petroleum ether / EtOAc), to yield the intermediate α,β-unsaturated lactone as a colourless oil, which co-eluted with residual phosphonate, and was therefore used in the next step without further purification.

To a stirred solution of this lactone in CH_2Cl_2 (2.8 mL) and water (13 μL) at 0 °C was added TFA (0.26 mL) dropwise. The reaction mixture was stirred for 15 min at 0 °C before being neutralized by addition of $NaHCO_3$ (sat., aq., 5 mL). The layers were separated and the aqueous phase was extracted three times with CH_2Cl_2 (3x10 mL). The combined organic layers were dried ($MgSO_4$) and concentrated. The product was purified by flash chromatography on a short plug of silica (1:1 petroleum ether / Et_2O eluent), to give lactol **19** (7.7 mg, 0.025 mmol, 47% over 2 steps, inseparable 67:33 mixture of lactol epimers) as a colourless oil.

R_f 0.18 (Petroleum Ether/ EtOAc (1:1)); **IR** (thin film, ν_{max} / cm^{-1}) 3420, 2959, 2138, 1752, 1252, 1005, 841; **HRMS** (ES^+) calc. for $C_{16}H_{24}O_4NaSi$ $[M+Na]^+$ 331.1336; found 331.1328.

Major diastereomer: **^{1}H NMR** (500 MHz, $CDCl_3$) δ_H 7.50 (1H, d, J = 5.6 Hz, H4), 6.13 (1H, d, J = 5.6 Hz, H3), 5.38 (1H, dd, J = 4.6 and 1.7 Hz, H8), 2.95 (1H, dd, J = 13.0 and 6.5 Hz, H6), 2.94 (1H, d, J = 16.8 Hz, H12), 2.66 (1H, d, J = 16.8 Hz, H12), 2.56 (1H, br s, OH), 1.87 (1H, tdd, J = 12.8, 4.6 and 1.3 Hz, H7), 1.73 (1H, dd, J = 12.5 and 6.5 Hz, H7), 1.55 (3H, s, H11), 1.25 (3 H, s, H11'), 0.15 (9H, s, $Si(CH_3)_3$); **^{13}C NMR** (126 MHz, $CDCl_3$) δ_C 172.1 (C2), 159.4 (C4), 121.2 (C3), 99.4 (C13), 95.3 (C8), 90.7 (C14), 87.9 (C5), 83.8 (C10), 48.2 (C6), 35.3 (C7), 32.4 (C11), 29.3 (C12), 25.4 (C11'), –0.1 ($Si(CH_3)_3$).

Minor diastereomer: **^{1}H NMR** (500 MHz, $CDCl_3$) δ_H 7.49 (1H, d, J = 5.7 Hz, H4), 6.15 (1H, d, J = 5.7 Hz, H3), 5.48 (1H, ddd, J = 6.2, 5.0 and 3.8 Hz, H8), 2.86 (1H, d, J = 16.9 Hz, H12), 2.76 (1H, d, J = 3.8 Hz,

OH), 2.66 (1H, d, J = 16.9 Hz, H12), 2.59 (1H, dd, J = 12.5 and 8.3 Hz, H6), 2.23 (1H, ddd, J = 13.6, 7.9 and 6.2 Hz, H7), 1.63 (1H, td, J = 12.9 and 5.0 Hz, H7), 1.43 (3H, m, H11), 1.35 (3H, s, H11'), 0.15 (9H, s, $Si(CH_3)_3$); **^{13}C NMR** (126 MHz, $CDCl_3$) δ_C 171.8 (C2), 158.5 (C4), 121.7 (C3), 99.3 (C13), 96.1 (C8), 90.8 (C14), 87.9 (C5), 82.7 (C10), 51.9 (C6), 36.2 (C7), 30.3 (C11), 30.1 (C12), 25.8 (C11'), –0.1 ($Si(CH_3)_3$).

Ethyl 3-((2*S*,3*S*)-3-(2-hydroxypropan-2-yl)-5-methoxy-2-(3-(trimethylsilyl)prop-2-yn-1-yl)tetrahydro furan-2-yl)acrylate, 20; and conversion of 20 to 19

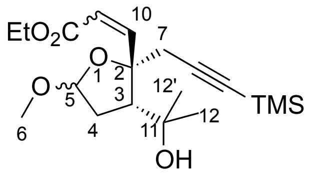

To a stirred solution of ethyl 2-(diphenoxyphosphoryl)acetate (62.1 mg, 0.19 mmol, 2.0 equiv.) in THF (1.9 mL) under Ar at 0 °C was added KHMDS (0.50 M in toluene, 0.37 mL, 0.18 mmol, 1.9 equiv.). After stirring at 0 °C for 20 min., the resulting mixture was tranferred to a syringe and added dropwise to a solution of aldehyde **18** (29.0 mg, 97.0 μmol, 1.0 equiv.) in THF (1.0 mL) at -20 °C, and the reaction mixture was stirred between –20 °C and 0 °C for 2 h. The reaction was then quenched by addition of NH_4Cl (sat., aq., 10 mL). Et_2O was added (20 mL), the layers were separated, and the aqueous phase was extracted with Et_2O (2 x 20 mL). The combined organic layers were dried (Na_2SO_4) and concentrated. The crude product was purified by flash chromatography on a short plug of silica (8:2 Petroleum ether / EtOAc), to yield the α,β-unsaturated ester **20** (*Z*:*E* = 1:0.4, both as a 1:1 mixure of epimers at the C5) as a colourless oil, which co-eluted with residual phosphonate, and was therefore used in the next step without further purification. To a stirred solution of this ester in CH_2Cl_2 (5.0 mL) and water (23.0 μL) at 0 °C was added TFA (0.46 mL) dropwise. The reaction mixture was stirred for 1 h at 0 °C before being neturalized by addition of $NaHCO_3$ (10 mL, sat., aq.). The layers were separated and the aqueous phase was extracted with CH_2Cl_2 (2 x 20 mL). The combined organic layers were dried (Na_2SO_4) and concentrated. The product was purified by flash chromatography on a short plug of silica (1:1 petroleum ether / EtOAc), to give lactol **19** (14.0 mg, 45.3 μmol, inseparable 1:0.35 mixture of lactol epimers, 47% over 2 steps) as a colourless oil. Data identical to this compound prepared from **17** as described above.

2-((3*S*,3a*R*,6a*R*)-2,2-dimethyl-5-oxo-3a-(prop-2-yn-1-yl)hexahydrofuro[3,2-*b*]furan-3-yl)acetaldehyde, 8

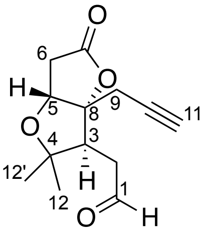

To a stirred solution of lactol **19** (134 mg, 0.436 mmol, 1.0 equiv.) in MeOH (18 mL) under Ar was added K_2CO_3 (241 mg, 1.74 mmol, 4.0 equiv.). The reaction mixture was stirred at rt for 2 h, then NH_4Cl (10 mL, sat., aq.) was added. The mixture was extracted three times with EtOAc (3 x 10 mL), then the combined

organic layers were dried (Na_2SO_4) and concentrated. The product was purified by flash chromatography on a short plug of silica (2:1 petroleum ether / EtOAc) to give the aldehyde **8** (103 mg, 0.436 mmol, quant.) as a white solid. $[\alpha]_D^{25}$ +38.5 (*c* = 1.05, $CHCl_3$); **mp** 93-96 °C; R_f 0.26 (3:2 petroleum ether / EtOAc); **IR** (thin film, ν_{max} / cm^{-1}) 2977, 1775, 1722, 1475, 1246, 1199, 1058; **^{1}H NMR** (400 MHz, $CDCl_3$) δ_H 9.81 (1H, t, *J* = 2.0 Hz, H1), 4.54 (1H, d, *J* = 6.4 Hz, H5), 3.02 (1H, dd, *J* = 18.7 and 6.7 Hz, H6), 2.80 (1H, dd, *J* = 9.0 and 6.2 Hz, H3), 2.73 (1H, dd, *J* = 18.7 and 0.5 Hz, H6), 2.70 (1H, dd, *J* = 17.0 and 2.6 Hz, H9), 2.61 (1H, ddd, *J* = 16.7, 9.0 and 2.1 Hz, H2), 2.51 (1H, dd, *J* = 17.0 and 2.6 Hz, H9), 2.43 (1H, ddd, *J* = 16.7, 6.2 and 1.9 Hz, H2), 2.13 (1H, t, *J* = 2.6 Hz, H11), 1.37 (3H, s, H12), 1.14 (3 H, s, H12); **^{13}C NMR** (101 MHz, $CDCl_3$) δ_C 199.3, 175.0, 94.1, 83.2, 78.7, 77.4, 72.9, 52.8, 40.7, 37.3, 28.0, 25.5, 21.2; **HRMS** (ES^+) calc. for $C_{13}H_{16}NaO_2$ $[M+Na]^+$ 259.0941; found 259.0938.

1.2.3. Synthesis of ABCDE intermediate 23 *via* Pd catalysis route

Intermediate **23** was prepared by the following Pd- or Co-catalyzed routes:

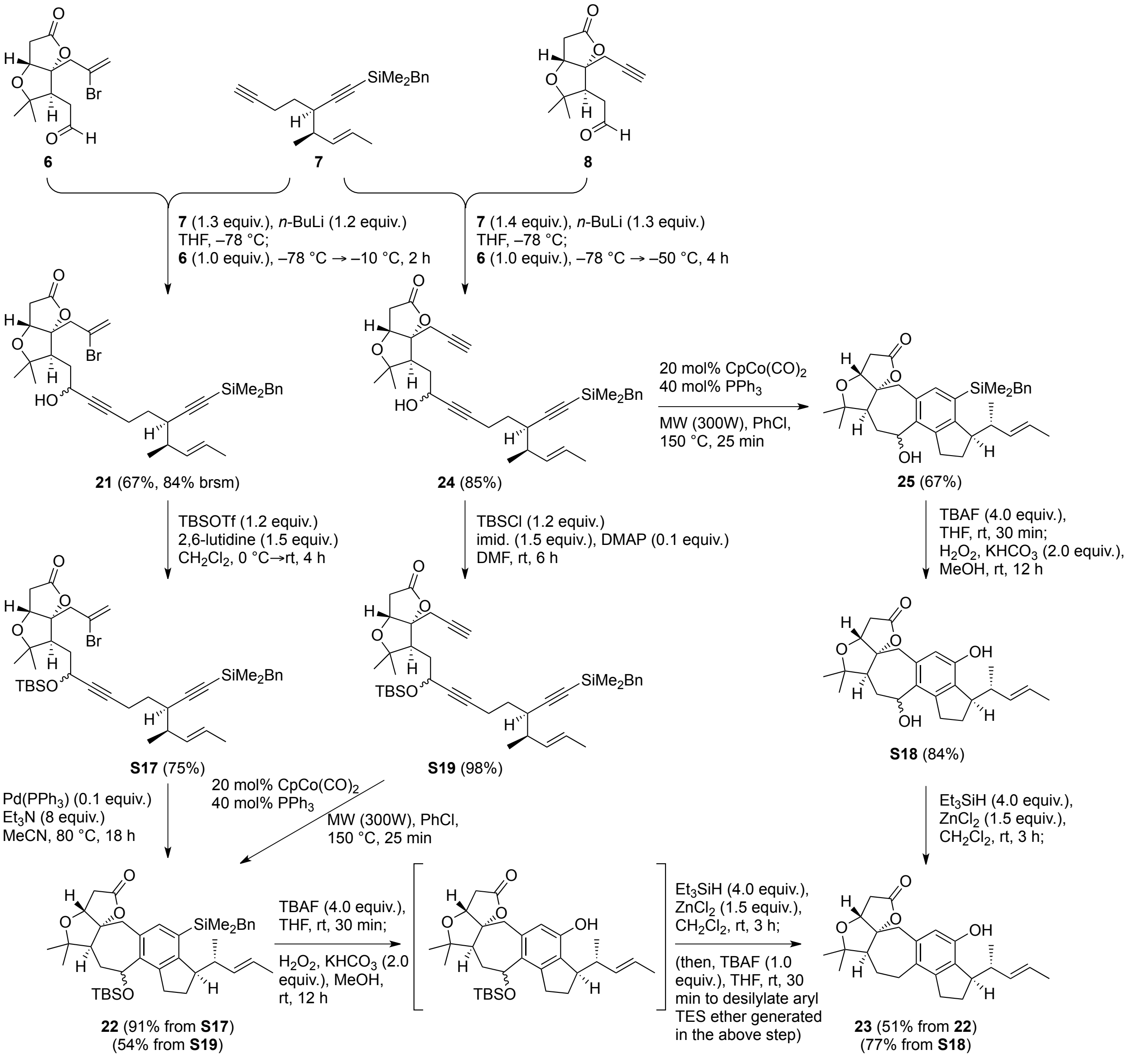

NB: All pentacyclic compounds are characterized with 'rubriflordilactone A' numbering.

(3a*R*,6*S*,6a*R*)-6-((7*R*,8*R*,*E*)-7-((Benzyldimethylsilyl)ethynyl)-2-hydroxy-8-methylundeC9-en-3-yn-1-yl)-6a-(2-bromoallyl)-5,5-dimethyltetrahydrofuro[3,2-b]furan-2(3*H*)-one, 21

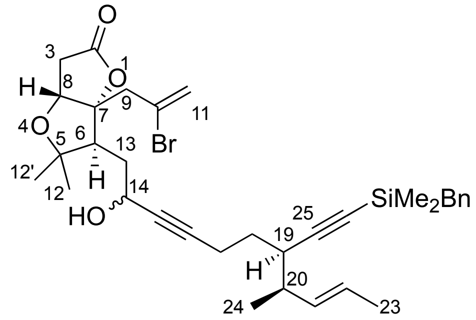

(*Diyne **7** and aldehyde **6** were both azeotroped 3 times using 3 mL of toluene each time and further dried on high-vacuum prior to use*). To a stirred solution of diyne **7** (38.2 mg, 0.16 mmol, 1.3 equiv.) in THF (0.60 mL) under N_2 at –78 °C was added *n*-BuLi (2.5 M in hexanes, 56.0 μL, 0.14 mmol, 1.2 equiv.) dropwise. The reaction mixture was stirred for 45 min, then a solution of aldehyde **6** (38.2 mg, 0.12 mmol, 1.0 equiv.) in THF (0.92 mL) was added. The reaction mixture was stirred for 2 h, with warming from –78 °C to –10 °C, before being diluted with Et_2O (10 mL) and quenched with NH_4Cl (5 mL, sat., aq.,), then warmed to rt. The layers were separated and the aqueous phase was extracted with Et_2O (2 x 20 mL). The combined organic layers were dried ($MgSO_4$) and concentrated. The product was purified by flash chromatography on a short plug of silica (7:3 Petroleum ether / EtOAc), to give secondary alcohol **21** (50.3 mg, 80.4 μmol, 67%; 84% brsm; inseparable 1:0.50 mixture of diastereomers) as a colourless oil. **R_f** 0.48 (7:3 petroleum ether / EtOAc); **IR** (thin film, ν_{max} / cm^{-1}) 3435, 2969, 2166, 1785, 1209, 1019, 837; **HRMS** (ES^+) calc. for $C_{34}H_{45}O_4{}^{79}Br^{23}Na^{28}Si$ $[M+Na]^+$ 647.21627; found 647.21582; **^{1}H NMR** (500 MHz, $CDCl_3$) δ_H 7.21 (2H, t, *J* = 7.4 Hz, ArH), 7.08 (3H, app t, *J* = 8.1 Hz, ArH), 5.77 (1H, s, H11), 5.71 (1H, s, H11), 5.44 (1H, dq, *J* = 15.2, 6.3 Hz, H22), 5.30 (1H, app ddd, *J* = 15.2, 8.2, 1.3 Hz, H21), 4.86 (1H, d, *J* = 6.1 Hz, $H7^{maj}$), 4.85 (1H, d, *J* = 6.1 Hz, $H7^{min}$), 4.55-4.52 (1H, m, $H14^{maj}$), 4.45-4.40 (1H, m, $H14^{min}$), 3.42 (1H, d, *J* = 14.9 Hz, $H9^{maj}$), 3.41 (1H, d, *J* = 14.9 Hz, $H9^{min}$), 2.89 (1H, dd, *J* = 18.8, 7.0 Hz, $H3^{maj}$), 2.88 (1H, dd, *J* = 18.8, 7.0 Hz, $H3^{min}$), 2.67 (1H, d, *J* = 14.9 Hz, $H9^{min}$), 2.66 (1H, dd, *J* = 18.8 and 0.7 Hz, $H3^{maj}$), 2.64 (1H, dd, *J* = 18.8 and 0.7 Hz, $H3^{min}$), 2.64 (1H, d, *J* = 14.7 Hz, $H9^{maj}$), 2.50 (1H, dd, *J* = 9.6, 3.5 Hz, $H6^{maj}$), 2.41 (1H, dddd, *J* = 16.6, 9.0, 5.1, and 2.0 Hz, H17), 2.41 (1H, obscured, $H6^{min}$), 2.33-2.21 (2H, m, H19 and H17), 2.17 (2H, s, Si$\underline{CH_2}$Ph), 2.12 (1H, app sextet, *J* = 7.1 Hz, H20), 2.04 (1H, d, *J* = 4.7 Hz, OH^{min}), 2.02 (1H, d, *J* = 6.2 Hz, OH^{maj}), 1.84 (1H, ddd, *J* = 14.5, 9.6 and 5.0 Hz, $H13^{maj}$), 1.81 (1H, obscured, $H13^{min}$) 1.67 (3H, d, *J* = 6.3 Hz, H23), 1.70-1.47 (3H, m, H13, H19 and H17), 1.35 (3H, s, $H12^{min}$), 1.33 (3H, s, $H12^{maj}$), 1.09 (3H, s, H12), 1.05 (3H, d, *J* = 6.8 Hz, H24), 0.11 (6H, s, $Si(CH_3)_2Bn$).; **^{13}C NMR** (125 MHz, $CDCl_3$) δ_C 175.7, 175.6, 139.4 (2C), 134.9, 134.8, 128.5 (4C), 128.2 (4C), 125.2, 125.2, 124.6, 124.5, 124.4, 124.4, 124.3 (2C) 109.5, 109.5, 95.2, 95.0, 86.8, 86.4, 85.9 (2C), 83.1 (2C), 80.5, 80.3, 76.8, 76.8, 62.0, 61.2, 57.0, 55.7, 46.0, 45.9, 40.9 (2C), 38.4 (2C), 37.5, 37.4, 34.3, 33.6, 31.8 (2C), 27.5, 27.5, 26.6 (2C), 20.5, 18.1, 18.1, 18.1 (2C), 17.0 (2C), –1.6 (4C); Note: '2C' refers to overlapping peaks from a single carbon atom in both the major and minor diastereomers. '4C' refers to overlapped equivalent carbon atoms in both diastereomers on the benzyldimethylsilane group.

(3a*R*,6*S*,6a*R*)-6-((7*R*,8*R*,*E*)-7-((Benzyldimethylsilyl)ethynyl)-2-((*tert*-butyldimethylsilyl)oxy)-8-methylundeC9-en-3-yn-1-yl)-6a-(2-bromoallyl)-5,5-dimethyltetrahydrofuro[3,2-b]furan-2(3*H*)-one, S17

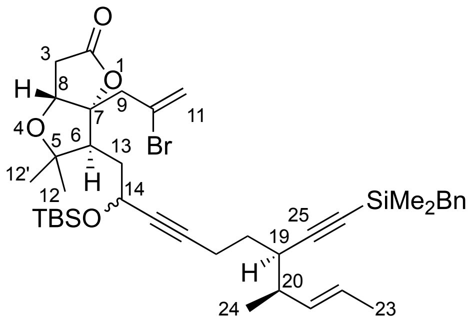

To a stirred solution of alcohols **21** (24.3 mg, 0.039 mmol, 1.0 equiv.) in CH_2Cl_2 (0.5 mL) under N_2 at 0 °C was added sequentially 2,6-lutidine (6.9 μL, 0.059 mmol, 1.5 equiv.), and TBSOTf dropwise (11 μL, 0.047 mmol, 1.2 equiv.). The reaction mixture was stirred and slowly warmed to rt over 4 h, then diluted with CH_2Cl_2 (10 mL) before being quenched by addition of NH_4Cl (10 mL, sat., aq.). The layers were separated and the aqueous phase was extracted with CH_2Cl_2 (2 x 20 mL). The combined organic layers were dried (Na_2SO_4) and concentrated. The product was purified by flash chromatography (9:1 petroleum ether / Et_2O eluent, then 7:3 petroleum ether / EtOAc to collect the remaining starting material) to afford the bromoendiyne **S17** (22.3 mg, as a 1:1 mixture of diastereomers, 75%) as a colourless oil, along with 2.8 mg of the remaining alcohols **21** (85% brsm). **R_f** 0.32 (9:1 petroleum ether / Et_2O); **IR** (thin film, ν_{max} / cm^{-1}) 3025, 2957, 2857, 2167, 1789, 1250, 1208, 1083, 837; **HRMS** (ES^+) calc. for $C_{40}H_{59}O_4{}^{79}Br^{23}Na^{28}Si_2$ $[M+Na]^+$ 761.30275; found 761.30231; **^{1}H NMR** (500 MHz, $CDCl_3$) δ_H 7.21 (2H, t, *J* = 7.7 Hz, SiCH$_2$<u>Ph</u>), 7.11-7.04 (3H, m, SiCH$_2$<u>Ph</u>), 5.77 (1H, s, H11), 5.71 (1H, s, H11), 5.52-5.36 (1H, m, H22), 5.36-5.24 (1H, m, H21), 4.82 (1H, d, *J* = 6.8 Hz, H8), 4.40-4.36 (1H, m, H14), 3.16 (1H, d, *J* = 14.9 Hz, $H9^{min}$), 3.14 (1H, d, *J* = 14.9 Hz, $H9^{maj}$), 2.86 (1H, dd, *J* = 18.7 and 6.5 Hz, $H3^{min}$), 2.85 (1H, dd, *J* = 18.8 and 6.8 Hz, $H3^{maj}$), 2.67 (1H, d, *J* = 15.0 Hz, H9), 2.63 (1H, dd, *J* = 18.8 and 0.7 Hz, H3), 2.45 (1H, dd, *J* = 9.8 and 3.3 Hz, H6), 2.42-2.20 (3H, m, 2 x H17 and H19), 2.17 (2H, s, SiC<u>H$_2$</u>Ph), 2.15-2.07 (1H, m, H20), 1.80-1.70 (1H, m, H13), 1.70-1.63 (1H, m, H18), 1.66 (3H, d, *J* = 6.2 Hz, H23), 1.34 (3H, s, $H12^{min}$), 1.31 (3H, s, $H12^{maj}$), 1.08 (3H, s, $H12^{min}$), 1.07 (3H, s, $H12^{maj}$), 1.05 (3H, d, *J* = 6.8 Hz, $H24^{maj}$), 1.05 (3H, d, *J* = 6.8 Hz, $H24^{min}$), 0.93 (9H, s, $OSiC(C\underline{H}_3)_3{}^{min}$), 0.91 (9H, s, $OSiC(C\underline{H}_3)_3{}^{maj}$), 0.18-0.14 (6H, m, $Si(C\underline{H}_3)_2Bn$), 0.11 (6H, s, $OSi(C\underline{H}_3)_2{}^tBu^{maj}$), 0.11 (3H, s, $OSi(C\underline{H}_3)_2{}^tBu^{min}$); **^{13}C NMR** (126 MHz, $CDCl_3$) δ_C 175.7 ($C2^{maj}$), 175.5 ($C2^{min}$), 139.4 (2C, SiCH$_2$<u>Ph</u>), 134.9 (2C, C21), 128.5 (2C, SiCH$_2$<u>Ph</u>), 128.2 (2C, SiCH$_2$<u>Ph</u>), 125.2 (2C, C22), 124.5 (2C, C11), 124.4 (2C, SiCH$_2$<u>Ph</u>), 124.4 (2C, C10), 109.6 (2C, C≡C), 95.2 ($C10^{min}$), 95.1 ($C10^{maj}$), 85.8 ($C≡C^{maj}$), 85.8 ($C≡C^{min}$), 85.6 (2C, C≡C), 83.3 ($C4^{min}$), 83.1 ($C4^{maj}$), 81.4 ($C≡C^{maj}$), 81.2 ($C≡C^{min}$), 77.3 ($C8^{min}$), 76.8 ($C8^{maj}$), 62.4 ($C14^{min}$), 61.2 ($C14^{maj}$), 56.2 ($C6^{min}$), 55.3 ($C6^{maj}$), 46.0 ($C9^{maj}$), 46.0 ($C9^{min}$), 40.9 ($C20^{min}$), 40.9 ($C20^{maj}$), 38.3 (2C, C19), 37.5 ($C3^{maj}$), 37.4 ($C3^{min}$), 35.5 ($C13^{min}$), 35.4 ($C13^{maj}$), 31.9 (2C, C18), 27.6 (2C, C12), 26.7 (2C, Si<u>C</u>H$_2$Ph), 26.0 ($OSiC(\underline{C}H_3)_3{}^{min}$), 26.0 ($OSiC(\underline{C}H_3)_3{}^{maj}$), 20.7 ($C12^{min}$), 20.6 ($C12^{maj}$), 18.4 ($C24^{min}$), 18.3 ($C24^{maj}$), 18.2 ($C23^{min}$), 18.1 ($C23^{maj}$), 18.1 (2C, $OSi\underline{C}(CH_3)_3$), 17.0 (2C, C17), –1.6 (2C, $Si(\underline{C}H_3)_2Bn$), –4.1 ($OSi(\underline{C}H_3)_2{}^tBu^{maj}$), –4.2 ($OSi(\underline{C}H_3)_2{}^tBu^{min}$), –4.6 ($OSi(\underline{C}H_3)_2{}^tBu^{min}$), –4.6 ($OSi(\underline{C}H_3)_2{}^tBu^{maj}$).

(3a*R*,5a*S*,10*R*,13a*R*)-11-(Benzyldimethylsilyl)-7-((*tert*-butyldimethylsilyl)oxy)-5,5-dimethyl-10-((*R,E*)-pent-3-en-2-yl)-3,3a,5,5a,6,7,8,9,10,13-decahydro-2*H*furo[3,2-b]indeno[4',5':5,6]cyclohepta[1,2-c]furan-2-one, 22

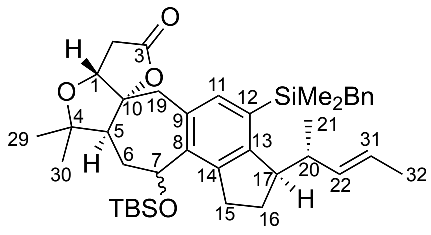

Dry MeCN and NEt_3 were separately degassed for 1 h prior to the reaction, with a thorough N_2 bubbling. Bromoenediyne **S17** (70.9 mg, 0.093 mmol, 1.0 equiv.) was dissolved in degassed MeCN (3.9 mL), and the resulting solution was again degassed with N_2 bubbling for 15 min. To a vial equipped with a stirrer bar containing $Pd(PPh_3)_4$ (10.7 mg, 0.0093 mmol, 10 mol%) was added the degassed solution of starting material by syringe (0.5 mL of degassed MeCN were used to rinse the flask two times), followed by degassed triethylamine (0.10 mL, 0.744 mmol, 8.0 equiv.). The vial was capped and the resulting mixture stirred at 80 °C overnight, then cooled to rt and concentrated. The product was purified by flash chromatography (4:1 petroleum ether / Et_2O eluent) to give pentacycle **22** (57.7 mg, as an inseparable 67:33 mixture of diastereomers, 0.087 mmol, 91%) as a yellow oil. **IR** (thin film, ν_{max} / cm^{-1}) 2927, 2855, 1780, 1458, 1386, 1251, 1193, 1067, 935, 835; **HRMS** (ES^+) calc. for $C_{40}H_{58}NaO_4Si_2$ $[M+Na]^+$ 681.3766; found 681.3761.

Major diastereomer: R_f 0.20 (3:1 petroleum ether / Et_2O); **^{1}H NMR** (500 MHz, $CDCl_3$) δ_H 7.17 (2H, t, *J* = 7.6 Hz, $SiCH_2\underline{Ph}$), 7.04 (1H, t, *J* = 7.4 Hz, $SiCH_2\underline{Ph}$), 6.94 (2H, d, *J* = 7.3 Hz, $SiCH_2\underline{Ph}$), 6.86 (1H, s, H11), 5.25 (1H, dqd, *J* = 15.8, 6.2 and 0.9 Hz, H23), 5.15 (1H, ddd, *J* = 15.8, 5.7 and 0.9 Hz, H22), 5.03 (1H, dd, *J* = 9.2 and 6.1 Hz, H7), 4.40 (1H, d, *J* = 5.1 Hz, H1), 3.67 (1H, d, *J* = 14.4 Hz, H19), 3.17 (1H, m, H17), 2.91-2.83 (1H, m, H15), 2.84 (1H, dd, *J* = 18.2 and 5.1 Hz, H2), 2.74-2.68 (1H, m, H15), 2.69 (1H, d, *J* = 18.2 Hz, H2), 2.52-2.46 (1H, m, H20), 2.49 (1H, d, *J* = 14.4 Hz, H19), 2.33 (1H, app d, *J* = 4.0 Hz, $Si\underline{CH_2}Ph$), 2.21-2.12 (1H, m, H6), 2.08 (1H, dd, *J* = 12.6 and 2.8 Hz, H5), 2.01 (1H, dd, *J* = 13.4 and 8.5 Hz, H6), 1.98-1.87 (2H, m, H16), 1.51 (3H, d, *J* = 6.2 Hz, H24), 1.29 (3H, s, H25), 1.21 (3H, s, H25), 1.11 (3H, d, *J* = 6.8 Hz, H21), 0.89 (9H, s, $OSiC(C\underline{H_3})_3$), 0.28 (3H, s, $Si(C\underline{H_3})_2Bn$), 0.23 (3H, s, $Si(C\underline{H_3})_2Bn$), 0.09 (3H, s, $OSi(C\underline{H_3})_2{}^tBu$), 0.04 (3H, s, $OSi(C\underline{H_3})_2{}^tBu$); **^{13}C NMR** (126 MHz, $CDCl_3$) δ_C 174.8 (C3), 152.7 (C13), 144.4 (C14), 140.0 ($SiCH_2\underline{Ph}$), 137.5 (C11), 135.9 (C8), 133.2 (C12), 131.9 (C23), 130.6 (C9), 128.6 ($SiCH_2\underline{Ph}$), 128.2 ($SiCH_2\underline{Ph}$), 124.5 ($SiCH_2\underline{Ph}$), 124.2 (C22), 98.9 (C10), 85.0 (C4), 79.6 (C1), 71.8 (C7), 51.6 (C5), 50.7 (C17), 41.7 (C20), 39.3 (C19), 36.9 (C2), 35.6 (C6), 31.3 (C15), 29.9 (C25), 27.3 ($Si\underline{C}H_2Ph$), 26.3 ($OSiC(\underline{C}H_3)_3$), 25.1 (C16), 23.8 (C25), 19.2 (C21), 18.6 (C24), 18.5 ($OSi\underline{C}(CH_3)_3$), –1.4 ($Si(\underline{C}H_3)_2Bn$), –1.5 ($Si(\underline{C}H_3)_2Bn$), –3.5 ($OSi(\underline{C}H_3)_2{}^tBu$), –3.6 ($OSi(\underline{C}H_3)_2{}^tBu$).

Minor diastereomer: R_f 0.24 (3:1 petroleum ether / Et_2O); **^{1}H NMR** (500 MHz, $CDCl_3$) δ_H 7.15 (2H, t, *J* = 7.6 Hz, $SiCH_2\underline{Ph}$), 7.05 (1H, t, *J* = 7.3 Hz, $SiCH_2\underline{Ph}$), 6.88 (2H, d, *J* = 7.0 Hz, $SiCH_2\underline{Ph}$), 6.73 (1H, s, H11), 5.12-4.97 (3H, m, H22, H31 and H7), 4.13 (1H, d, *J* = 5.3 Hz, H1), 3.70 (1H, d, *J* = 14.9 Hz, H19), 3.17-3.08 (1H, m, H17), 2.90-2.80 (2H, m, H15 and H5), 2.77 (1H, dd, *J* = 18.4 and 5.3 Hz, H2), 2.75 (1H, d, *J* = 18.4 Hz, H19), 2.67 (1H, d, *J* = 18.4 Hz, H19), 2.57 (1H, dt, *J* = 15.9 and 9.4 Hz, H15), 2.41-2.33 (1H, m, H20), 2.30 (2H, s, $Si\underline{CH_2}Ph$), 2.14 (1H, ddd, *J* = 14.7, 6.8 and 5.5 Hz, H6), 1.97-1.88 (2H, m, 2×H16),

1.69 (1 H, ddd, J = 14.7, 11.7 and 1.0 Hz, H6), 1.46 (3H, d, J = 4.8 Hz, H32), 1.36 (3H, s, H29), 1.11 (3H, d, J = 6.9 Hz, H21), 1.05 (3H, s, H30), 0.89 (9H, s, OSiC(C<u>H</u>$_3$)$_3$), 0.33 (3H, s, Si(C<u>H</u>$_3$)$_2$Bn), 0.26 (3H, s, Si(C<u>H</u>$_3$)$_2$Bn), 0.12 (3H, s, OSi(C<u>H</u>$_3$)$_2$^{t}Bu), 0.02 (3H, s, OSi(C<u>H</u>$_3$)$_2$^{t}Bu); **^{13}C NMR** (126 MHz, CDCl$_3$) δ_C 175.5 (C3), 152.9 (C13), 142.2 (C14), 139.9 (SiCH$_2$<u>C</u>$_6$H$_5$), 138.6 (C8), 137.3 (C11), 132.4 (C12), 131.8 (C31), 129.7 (C9), 128.5 (SiCH$_2$<u>C</u>$_6$H$_5$), 128.2 (SiCH$_2$<u>C</u>$_6$H$_5$), 124.3 (SiCH$_2$<u>C</u>$_6$H$_5$), 124.3 (C22), 97.5 (C10), 85.0 (C4), 78.7 (C1), 69.3 (C7), 51.0 (C17), 50.9 (C5), 42.3 (C20), 40.9 (C19), 37.1 (C2), 33.1 (C6), 30.8 (C15), 29.8 (C29), 27.5 (Si<u>C</u>H$_2$C$_6$H$_5$), 26.0 (OSiC(<u>C</u>H$_3$)$_3$), 25.0 (C16), 23.9 (C30), 19.2 (C32), 18.4 (C21), 18.3 (OSi<u>C</u>(CH$_3$)$_3$), –1.2 (Si(<u>C</u>H$_3$)$_2$Bn), –1.5 (Si(<u>C</u>H$_3$)$_2$Bn), –3.5 (OSi(<u>C</u>H$_3$)$_2$^{t}Bu), –4.8 (OSi(<u>C</u>H$_3$)$_2$^{t}Bu).

(3a*R*,5a*S*,10*R*,13a*R*)-11-hydroxy-5,5-dimethyl-10-((*R*,*E*)-pent-3-en-2-yl)-3,3a,5,5a,6,7,8,9,10,13-decahydro-2*H*-furo[3,2-b]indeno[4',5':5,6]cyclohepta[1,2-c]furan-2-one, 23

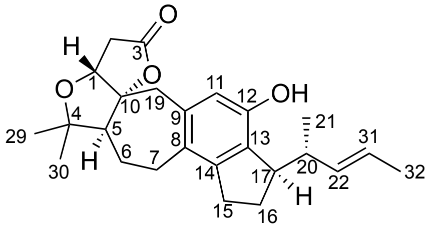

To a stirred solution of benzyldimethylarylsilane **22** (30.9 mg, 47.0 μmol, 1.0 equiv.) in THF (1.1 mL) was added TBAF (1 M in THF, 0.19 mL, 0.19 mmol, 4.0 equiv.). The reaction mixture was stirred for 30 min, then H_2O_2 (30% w/v in water, 0.11 mL, 0.94 mmol, 20.0 equiv.) in MeOH (1.1 mL) and $KHCO_3$ (9.4 mg, 94 μmol, 2.0 equiv.) were added. The reaction mixture was stirred overnight at rt, then diluted with Et_2O (8.0 mL), quenched by addition of $Na_2S_2O_3$ (4.0 mL, sat., aq.) and NH_4Cl (4.0 mL, sat., aq.). The layers separated and the aqueous phase was extracted with Et_2O (2 x 10 mL). The combined organic phases were dried ($MgSO_4$), concentrated, and quickly filtered through a short pad of silica gel to remove the remaining TBAF (1:1 petroleum ether / EtOAc eluent). The resulting mixture of phenols was used without further purification in the next step.

To a stirred solution of these phenols in CH_2Cl_2 (0.90 mL) was added anhydrous $ZnCl_2$ (9.7 mg, 71.0 μmol, 1.5 equiv.) and Et_3SiH (30.3 μL, 0.19 mmol, 4.0 equiv.). The reaction mixture was stirred for 3 h at rt before being quenched by addition of NH_4Cl (sat., aq., 2.0 mL) and diluted with Et_2O (5.0 mL). The layers were separated and the aqueous phase was extracted with Et_2O (2 x 5.0 mL). The combined organic layers were dried ($MgSO_4$) and concentrated. The resulting crude was dissolved in THF (2.0 mL) and TBAF (47.0 μL, 47.0 μmol, 1.0 equiv.) was added. The reaction mixture was stirred 30 min. at rt before being quenched by addition of NH_4Cl (sat., aq., 2.0 mL) and diluted with Et_2O (5.0 mL). The layers were separated and the aqueous phase was extracted with Et_2O (2 x 5.0 mL). The combined organic phases were dried ($MgSO_4$), concentrated, and the resulting crude was was purified by flash chromatography on a short plug of silica (6:4 petroleum ether / EtOAc) to yield phenol **23** (9.5 mg, 24.0 μmol, 51% over three steps) as a colourless.

$[\alpha]_D^{25}$ +20.0 (c = 0.20, $CHCl_3$); **R_f** 0.21 (3:2 petroleum ether / EtOAc); **IR** (thin film, ν_{max} / cm^{-1}) 3397, 2925, 1774, 1601, 1458, 1318, 1202, 1172, 935, 848; **^{1}H NMR** (500 MHz, CDCl$_3$) δ_H 6.25 (1H, s, H11), 5.56-5.47 (2H, m, H22 and H31), 4.83 (1H, br s, ArO<u>H</u>), 4.26 (1H, d, J = 6.0 Hz, H1), 3.53 (1H, d, J = 15.4 Hz, H19), 3.28 (1H, dt, J = 9.0 and 3.8 Hz, H17), 2.97 (1H, br dd, J = 17.2 and 4.4 Hz, H7), 2.85 (1H, dd, J =

18.6 and 6.0 Hz, H2), 2.77-2.66 (2H, m, H7 and H15), 2.70 (1H, d, J = 18.6 Hz, H2), 2.62 (1H, d, J = 15.4 Hz, H19), 2.62-2.53 (2H, m, H15 and H20), 2.28 (1H, dd, J = 12.7 and 3.4 Hz, H5), 2.15 (1H, ddd, J = 17.3, 13.0 and 9.0 Hz, H16), 2.05-1.96 (1H, m, H6), 1.93 (1H, m, H16), 1.76 (1H, ddt, J = 14.3, 5.4 and 3.4 Hz, H6), 1.66 (3 H, br d, J = 3.2 Hz, H32), 1.36 (3H, s, H29), 1.15 (3H, s, H30), 0.97 (3H, d, J = 6.9 Hz, H21); ^{13}**C NMR** (101 MHz, $CDCl_3$) δ_C 175.4 (C3), 150.4 (C12), 147.1 (C14), 135.9 (C22), 132.5 (C9), 129.0 (C13), 126.0 (C8), 125.8 (C31), 117.5 (C11), 99.2 (C10), 84.5 (C4), 79.7 (C1), 59.5 (C5), 48.9 (C17), 40.8 (C19), 39.9 (C20), 36.0 (C2), 31.5 (C15), 31.0 (C7), 29.1 (C16), 28.5 (C32), 24.1 (C6), 21.3 (C29), 18.2 (C30), 16.6 (C21); **HRMS** (ES^+) calc. for $C_{25}H_{31}NaO_4$ $[M+Na]^+$ 395.2228; found 395.2224.

1.2.4. Synthesis of ABCDE intermediate 23 *via* Co catalysis route

(3a*R*,6*S*,6a*R*)-6-((7*R*,8*R*,*E*)-7-((benzyldimethylsilyl)ethynyl)-2-hydroxy-8-methylundec-9-en-3-yn-1-yl)-5,5-dimethyl-6a-(prop-2-yn-1-yl)tetrahydrofuro[3,2-*b*]furan-2(5*H*)-one, 24

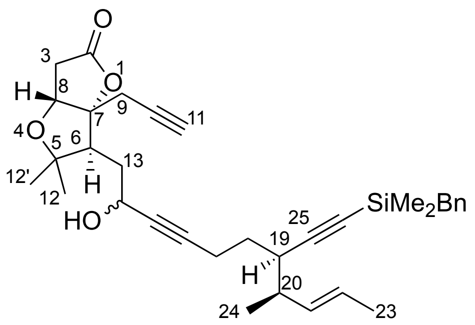

To a stirred solution of diyne **7** (137 mg, 0.444 mmol, 1.4 equiv.) in THF (3.7 mL) under Ar at –78 °C was added *n*-BuLi (2.5 M in hexanes, 0.165 mL, 0.412 mmol, 1.3 equiv.) dropwise. The reaction mixture was stirred for 30 min, then a solution of aldehyde **8** (75 mg, 0.317 mmol, 1.0 equiv.) in THF (2 mL) was added. The reaction mixture was stirred for 4 h, with warming from –78 °C to –50 °C, then quenched by addition of NH_4Cl (0.2 mL, sat., aq.) and warmed to rt. The mixture was filtered through a plug of Celite®, eluted with EtOAc, and concentrated. The product was purified by flash chromatography on silica gel (7:3 petroleum ether / EtOAc) to afford alcohol **24** (147 mg, inseparable 64:36 mixture of C14 diastereomers, 0.270 mmol, 85%) as a colourless oil. $\mathbf{R_f}$ 0.34 (7:3 petroleum ether / EtOAc); **IR** (thin film, ν_{max} / cm^{-1}) 3291, 2969, 2924, 2165, 1787, 1600, 1495, 1624, 1375, 1319, 1250, 1197, 1021, 837; ^{1}**H NMR** (500 MHz, $CDCl_3$) δ_H 7.21 (2H, t, J = 7.6 Hz, ArH), 7.14-6.99 (3H, m, ArH), 5.44 (1H, dq, J = 15.3 and 6.2 Hz, H22), 5.30 (1H, ddd, J = 15.3, 8.1 and 1.2 Hz, H21), 4.55-4.49 (1.64H, m, H8 and $H14^{maj}$), 4.45-4.39 (0.36H, m, $H14^{min}$), 3.10-2.97 (2H, m, H9 and H3), 2.68 (1H, ddd, J = 18.7, 8.0 and 0.9 Hz, H3), 2.54-2.45 (2H, m, H6 and H9), 2.45-2.36 (1H, m, H17), 2.34-2.20 (2H, m, H19 and H17), 2.16 (1H, s, $SiC\underline{H_2}Ph$), 2.12 (1H, app quin, J = 7.1 Hz, H20), 2.09-2.05 (1H, m, H11), 1.98 (0.64H, d, J = 4.3 Hz, OH^{maj}), 1.96 (0.36H, J = 6.3 Hz, OH^{min}) ,1.86-1.75 (1H, m, H13), 1.71-1.62 (1H, m, H18), 1.66 (3H, dd, J = 6.3 and 1.4 Hz, H23), 1.58 (0.36H, dd, J = 7.2 and 3.2 Hz, $H13^{min}$), 1.55-1.47 (1.64H, m, $H13^{maj}$ and H18), 1.34 (1.08H, s, $H12^{min}$), 1.33 (1.92H, s, $H12^{maj}$), 1.07 (3H, s, H12), 1.05 (3H, app d, J = 6.8 Hz, H24), 0.11 (6H, s, $Si(C\underline{H_3})_2Bn$); ^{13}**C NMR** (126 MHz, $CDCl_3$) δ_C 175.6 (C2), 139.4 (C_{Ar}), 134.9 ($C21^{min}$), 134.8 ($C21^{maj}$), 128.5 (C_{Ar}), 128.3 (C_{Ar}), 125.2 (C22), 124.4 (C_{Ar}), 109.5 (C25), 94.9 ($C7^{maj}$), 94.7 ($C7^{min}$), 86.8 ($C16^{maj}$), 86.4 ($C16^{min}$), 85.9 (C26), 83.5 (C5), 80.5 ($C15^{min}$), 80.3 ($C15^{maj}$), 78.9 ($C8^{min}$), 78.8 ($C8^{maj}$), 78.1 ($C10^{maj}$),

78.0 (C10^{min}), 72.4 (C11), 62.0 (C14^{min}), 61.3 (C14^{maj}), 56.1 (C6^{min}), 54.7 (C6^{maj}), 40.9 (C20), 38.4 (C19), 37.7 (C3^{maj}), 37.6 (C3^{min}), 34.3 (C13^{min}), 33.6 (C13^{maj}), 31.8 (C18), 27.7 (C12), 26.7 (Si$\underline{C}H_2$Ph), 26.1 (C9^{maj}), 25.9 (C9^{min}), 20.6 (C12), 18.2 (C24), 18.1 (C23), 17.1 (C17^{min}), 17.0 (C17^{maj}), –1.6 (Si(CH_3)$_2$Bn); **HRMS** (ES$^+$) calc. for $C_{34}H_{44}NaO_4Si$ [M+Na]$^+$ 567.2901; found 567.2895.

(3a*R*,5a*S*,10*R*,13a*R*)-11-(Benzyldimethylsilyl)-7-hydroxy-5,5-dimethyl-10-((*R*,*E*)-pent-3-en-2-yl)-3,3a,5,5a,6,7,8,9,10,13-decahydro-2*H*-furo[3,2-b]indeno[4',5':5,6]cyclohepta[1,2-c]furan-2-one, (7*S*)-25 and (7*R*)-25

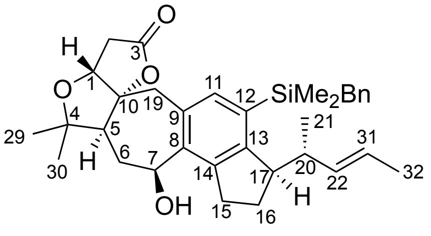

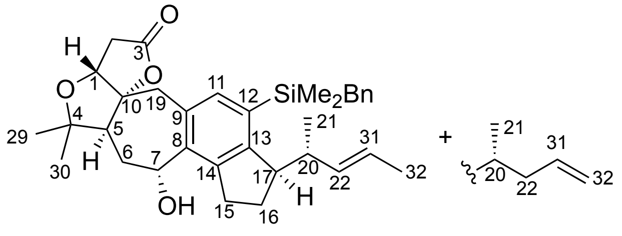

(7*S*)-25 **(7*R*)-25 + 25-ter (**85:15)

To a solution of triyne **24** (63.0 mg, 0.116 mmol, 1.0 equiv) in chlorobenzene (2.9 mL, 0.04 M) in a microwave tube was added PPh_3 (12.1 mg, 0.046 mmol, 40 mol%), and the mixture was degassed with Ar bubbling for 30 min. $CpCo(CO)_2$ (4.2 mg, 0.023 mmol, 20 mol%) was added and the reaction mixture was heated in a microwave (300 W) at 150 °C for 25 min. Upon cooling, the reaction was concentrated. The product was purified by flash chromatography (4:1 petroleum ether / EtOAc eluent) to afford **(7*R*)-25** (16.1 mg, 0.0296 mmol, 22%), which co-eluted as a 85:15 inseparable mixture with a C31–C32 terminal alkene isomer (4%), as a colourless oil, and **(7*S*)-25** (28.3 mg, 0.0519 mmol, 45%) as a white foam. **IR** (thin film, ν_{max} / cm^{-1}) 3476, 2957, 2930, 1776, 1600, 1493, 1373, 1248, 1201, 1059, 1107, 937, 817; **HRMS** (ES$^+$) calc. for $C_{34}H_{44}NaO_4Si$ [M+Na]$^+$ 567.2901; found 567.2882.

Data for (7*S*)-25: R$_f$ 0.23 (3:2 petroleum ether / EtOAc); **^{1}H NMR** (400 MHz, $CDCl_3$) δ_H 7.18 (2H, t, *J* = 7.5 Hz, $SiCH_2\underline{Ph}$), 7.06 (1H, t, *J* = 7.4 Hz, $SiCH_2\underline{Ph}$), 6.95 (2H, d, *J* = 7.4 Hz, $Si\underline{Ph}$), 6.84 (1H, s, H11), 5.16 (1H, ddd, *J* = 15.4, 6.7 and 0.8 Hz, H22), 5.02 (1H, dq, *J* = 15.4 and 6.1 Hz, H31), 4.92 (1H, dt, *J* = 9.9 and 6.1 Hz, H7), 4.32 (1H, d, *J* = 5.5 Hz, H1), 3.62 (1H, d, *J* = 15.0 Hz, H19), 3.12 (1H, dd, *J* = 8.1 and 2.1 Hz, H17), 2.93-2.83 (1H, m, H15), 2.84 (1H, dd, *J* = 18.2 and 5.5 Hz, H2), 2.80-2.72 (1H, m, H5), 2.69 (1H, d, *J* = 18.2 Hz, H2), 2.56 (1 H, d, *J* = 15.0 Hz, H19), 2.41-2.34 (1H, m, H20), 2.33 (2H, s, $SiC\underline{H_2}Ph$), 2.24 (1H, dd, *J* = 12.2, 3.5 Hz, H5), 2.21-2.04 (2H, m, H6), 2.03-1.83 (2H, m, H16), 1.65 (1H, d, *J* = 5.8 Hz, OH), 1.48 (3H, d, *J* = 6.1 Hz, H32), 1.38 (3H, s, H29), 1.22 (3H, s, H30), 1.12 (3H, d, *J* = 6.9 Hz, H21), 0.29 (3H, s, $Si(C\underline{H_3})_2Bn$), 0.25 (3H, s, $Si(C\underline{H_3})_2Bn$); **^{13}C NMR** (101 MHz, $CDCl_3$) δ_C 174.7 (C3), 153.6 (C13), 145.3 (C14), 139.9 ($SiCH_2\underline{Ph}$), 137.8 (C8), 136.0 (C11), 133.7 (C12), 132.1 (C22), 129.5 (C9), 128.6 ($SiCH_2\underline{Ph}$), 128.3 ($SiCH_2\underline{Ph}$), 124.3 (C31), 124.2 ($SiCH_2\underline{Ph}$), 98.7 (C10), 84.7 (C4), 79.5 (C1), 72.0 (C7), 52.8 (C5), 51.0 (C17), 42.2 (C20), 40.0 (C19), 36.1 (C2), 34.3 (C6), 31.7 (C15), 29.0 (C29), 27.2 ($Si\underline{C}H_2Ph$), 25.4 (C16), 22.2 (C30), 19.3 (C21), 18.3 (C32), –1.3 ($Si(\underline{C}H_3)_2Bn$), –1.4 ($Si(\underline{C}H_3)_2Bn$). The stereochemistry at C7 was assigned using a NOESY experiment, in which an enhancement was seen between H7 and H5. This enhancement was not observed in the equivalent NOESY experiment for (7*R*)-25.

Data for (7*R*)-25: This product contains a small amount (15%) of the C31–C32 terminal alkene regioisomer; signals from this compound are indicated as H^{ter} in the ^1H NMR data. **R_f** 0.51 (3:2 petroleum ether / EtOAc); **^{1}H NMR** (500 MHz, $CDCl_3$) δ_H 7.19 (2H, t, *J* = 7.6 Hz, $SiCH_2C_6\underline{H_5}$), 7.06 (1H, t, *J* = 7.4 Hz, $SiCH_2\underline{Ph}$), 6.94 (2H, d, *J* = 7.2 Hz, $SiCH_2\underline{Ph}$), 6.82 (0.85H, s, H11), 6.79 (0.15H, s, $H11^{ter}$), 5.58-5.49 (0.15H, m, $H31^{ter}$), 5.10-5.06 (1.7H, m, H22 and H31), 5.04 (1H, dd, *J* = 8.8, 6.5 Hz, H7), 4.91-4.86 (0.3H, m, 2 × $H32^{ter}$), 4.30 (1H, d, *J* = 6.6 Hz, H1), 3.40 (1H, d, *J* = 15.8 Hz, H19), 3.19 (1H, dt, *J* = 8.0 and 2.6, H17), 3.11 (1H, dd, *J* = 16.0, 8.6 and 1.9 Hz, H15), 2.93 (1H, dd, *J* = 19.0 and 6.6, H2), 2.83-2.75 (1H, m, H5), 2.80 (1H, d, *J* = 15.8 Hz, H19), 2.75 (1H, d, *J* = 19.0 Hz, H2), 2.74-2.67 (1H, m, H15), 2.43-2.35 (1 H, m, H20), 2.32 (2H, s, $SiC\underline{H_2}Ph$), 2.13 (1H, ddd, *J* = 14.2, 6.5 and 3.8 Hz, H6), 2.06-1.93 (3H, m, H6 and 2 × H16), 1.80 (1H, d, *J* = 9.0 Hz, OH), 1.73-1.64 (0.3H, m, 2 × $H22^{ter}$), 1.49 (2.55H, d, *J* = 3.6 Hz, H32), 1.40 (3H, s, H29), 1.20 (3H, s, H30), 1.12 (2.55H, d, *J* = 6.9 Hz, H21), 1.04 (0.45H, d, *J* = 6.8 Hz, $H21^{ter}$), 0.29 (3H, s, $Si(C\underline{H_3})_2Bn$), 0.25 (3H, s, $Si(C\underline{H_3})_2Bn$); **^{13}C NMR** (126 MHz, $CDCl_3$) δ_C 175.0 (C3), 153.0 (C13), 145.8 (C14), 139.9 ($SiCH_2\underline{Ph}$), 137.8 (C11), 136.3 (C8), 133.7 (C12), 131.8 (C22), 129.3 (C9), 128.6 ($SiCH_2\underline{Ph}$), 128.3 (+, $SiCH_2\underline{Ph}$), 124.3 (+, $SiCH_2\underline{Ph}$), 124.3 (C31), 98.4 (C10), 84.1 (C4), 79.8 (C1), 68.8 (C7), 52.5 (C5), 50.9 (C17), 42.1 (C20), 42.0 (C19), 36.1 (C2), 30.7 (C15), 30.6 (C6), 28.2 (C29), 27.2 ($Si\underline{C}H_2Ph$), 25.0 (C16), 21.4 (C30), 19.1 (C21), 18.3 (C32), –1.3 ($Si(\underline{C}H_3)_2Bn$), –1.6 ($Si(\underline{C}H_3)_2Bn$).

(3a*R*,5a*S*,10*R*,13a*R*)-7,11-Dihydroxy-5,5-dimethyl-10-((*R*,*E*)-pent-3-en-2-yl)-3,3a,5,5a,6,7,8,9,10,13-decahydro-2*H*-furo[3,2-b]indeno[4',5':5,6]cyclohepta[1,2-c]furan-2-one, (7*S*)-S18 and (7*R*)-S18

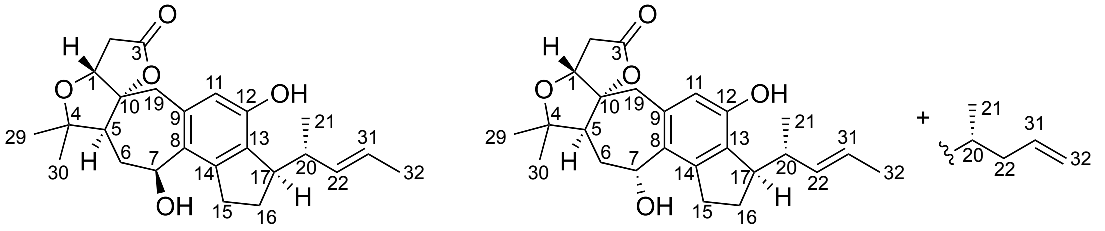

(7*S*)-S18 **(7*R*)-S18 + S18-ter** (85:15)

Representative procedure for the oxidation of **(7*S*)-25**, separated in the previous step: To a stirred solution of **(7*S*)-25** (39.5 mg, 0.0725 mmol, 1.0 equiv.) in THF (1.3 mL) was added TBAF (1 M in THF, 290 *μ*L, 0.290 mmol, 4.0 equiv.). The reaction mixture was stirred for 30 min, then H_2O_2 (30% w/v in water, 184 *μ*L, 1.45 mmol, 20 equiv.) in methanol (1.3 mL), and $KHCO_3$ (14.5 mg, 0.145 mmol, 2.0 equiv.) were added. The reaction mixture was stirred at RT overnight, then quenched with $Na_2S_2O_3$ (1 mL, sat., aq.) and NH_4Cl (1 mL, sat. aq.). The layers were separated and the aqueous phase was extracted with EtOAc (3 x 2 mL). The combined organic phases were dried (Na_2SO_4) and concentrated. The product was purified by flash chromatography (99:1 CH_2Cl_2 / MeOH eluent) to afford the phenol **(7*S*)-18** (25.2 mg, 84%) as a white foam.

IR (thin film, ν_{max} / cm^{-1}) 3376 (br s), 2961 (s), 2925 (s), 1774 (s), 1597 (m), 1461 (m), 1389 (m), 1319 (m), 1437 (m), 1246 (m), 1195 (m), 1171 (s), 1071 (m), 970 (m); **HRMS** (ESI^+) calc. for $C_{25}H_{32}NaO_5$ $[M+Na]^+$ 435.2142, found 435.2142.

Data for (7*S*)-S18: **R_f** 0.35 (19:1 CH_2Cl_2 / MeOH); **^{1}H NMR** (500 MHz, $CDCl_3$) δ_H 6.23 (1H, s, H11), 5.53-5.46 (2H, m, H22 and H31), 5.16 (1H, br s, OH), 4.94-4.84 (1H, m, H7), 4.31 (1H, d, *J* = 5.3 Hz, H1), 3.58

(1H, d, J = 14.9 Hz, H19), 3.22 (1H, ddd, J = 8.8, 4.7 and 2.5 Hz, H17), 3.08 (1H, dt, J = 16.6 and 8.5 Hz, H15), 2.81 (1H, dd, J = 18.4 and 5.3 Hz, H2), 2.77 (1H, ddd, J = 16.6, 9.1 and 2.7 Hz, H15), 2.69 (1H, d, J = 18.4 Hz, H2), 2.59-2.53 (1H, m, H20), 2.50 (1H, d, J = 14.9 Hz, H19), 2.26-2.20 (1H, m, H5), 2.16-2.07 (3H, m, H16 and H6), 2.00 (1H, ddt, J = 12.9, 8.5 and 2.5 Hz, H16), 1.66 (3H, d, J = 4.5 Hz, H32), 1.36 (3H, s, H29), 1.21 (3H, s, H30), 0.99 (3H, d, J = 6.9 Hz, H21); **^{13}C NMR** (126 MHz, $CDCl_3$) δ_C 174.9 (C3), 152.1 (C12), 149.4 (C14), 135.8 (C22), 132.7 (C9), 130.9 (C13), 127.6 (C8), 126.0 (C23), 117.5 (C11), 98.7 (C10), 84.8 (C4), 79.4 (C1), 71.7 (C7), 52.7 (C5), 48.6 (C17), 40.3 (C20), 39.7 (C19), 36.1 (C2), 34.6 (C6), 32.1 (C15), 29.5 (C16), 29.0 (C29), 22.3 (C30), 18.3 (C32), 17.0 (C21).

An equivalent procedure was carried out for **(7*R*)-25**, to give **(7*R*)-S18:** This product contains a small amount (15%) of the C31–C32 terminal alkene regioisomer (contained in the starting material, see above); signals from this impurity are indicated as H^{ter} in the ^{1}H NMR data. **R_f** 0.31 (19:1 CH_2Cl_2 / MeOH); **^{1}H NMR** (400 MHz, $CDCl_3$) δ_H 6.27 (0.85H, s, H11), 6.23 (0.15H, s, $H11^{ter}$), 5.76-5.64 (0.15H, m, $H31^{ter}$), 5.58-5.41 (1.7H, m H22 and H31), 5.22 (1H, br s, ArOH), 5.04 (1H, d, J = 5.9 Hz, H7), 4.99-4.90 (0.3H, m, 2 × $H32^{ter}$), 4.29 (1H, d, J = 6.4 Hz, H1), 3.37 (1H, d, J = 15.7 Hz, H19), 3.30 (1H, dt, J = 8.2 and 4.0 Hz, H17), 3.10 (1H, ddd, J = 16.1, 9.3 and 4.4 Hz, H15), 2.96-2.84 (1H, m, H15), 2.90 (1H, dd, J = 19.2 and 6.4 Hz, H2), 2.80 (1H, dd, J = 13.1 and 3.6 Hz, H5), 2.77-2.68 (2H, m, H2 and H19), 2.68-2.58 (1H, m, H20), 2.23-2.14 (1H, m, H16), 2.10 (1H, ddd, J = 14.2, 6.1 and 4.0 Hz, H6), 2.06-1.88 (3H, m, H6, H16 and OH), 1.84-1.68 (0.3H, m, 2 × $H22^{ter}$), 1.66 (2.55H, d, J = 4.5 Hz, H32), 1.39 (3H, s, H29), 1.18 (3H, s, H30), 0.98 (2.55H, d, J = 6.9 Hz, H21), 0.95 (0.45H, d, J = 6.9 Hz, $H21^{ter}$); **^{13}C NMR** (101 MHz, $CDCl_3$) δ_C 175.1 (C3), 152.1 (C12), 149.6 (C14), 135.6 (C22), 132.5 (C9), 130.0 (C13), 128.3 (C8), 126.0 (C23), 117.4 (C11), 98.5 (C10), 84.3 (C4), 79.7 (C1), 68.5 (C7), 52.3 (C5), 49.0 (C17), 41.8 (C19), 39.3 (C20), 36.0 (C2), 31.2 (C15), 30.9 (C6), 29.1 (C16), 28.2 (C29), 21.4 (C30), 18.3 (C21), 16.5 (C32).

(3a*R*,5a*S*,10*R*,13a*R*)-11-hydroxy-5,5-dimethyl-10-((*R*,*E*)-pent-3-en-2-yl)-3,3a,5,5a,6,7,8,9,10,13-decahydro-2*H*-furo[3,2-b]indeno[4',5':5,6]cyclohepta[1,2-c]furan-2-one, 23

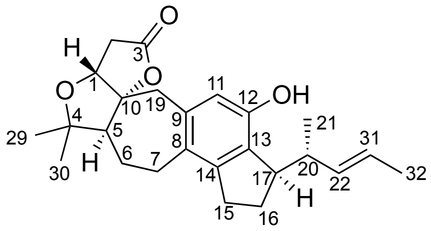

23

To a stirred solution of the benzylic alcohols **S18** (16.9 mg, 41.0 μmol, 1.0 equiv.) in CH_2Cl_2 (0.8 mL) was added $ZnCl_2$ (8.4 mg, 0.061 mmol, 1.5 equiv.) and triethylsilane (26 μL, 0.164 mmol, 4.0 equiv.). The reaction mixture was stirred at RT for 3 h, then NH_4Cl (1.5 mL, sat., aq.) was added, and the mixture was diluted with EtOAc (3 mL). The layers were separated and the aqueous phase was extracted with ethyl acetate (3 x 3 mL). The combined organic phases were dried ($MgSO_4$) and concentrated. The product was purified by flash chromatography (3:2 petroleum ether / EtOAc eluent) to give phenol **23** (12.5 mg, 31.5 μmol, 77%) as a colourless oil. The data for this compound was identical to that recorded for **23** derived from the Pd-catalyzed route (see above).

(3a*R*,6*S*,6a*R*)-6-((7*R*,8*R*,*E*)-7-((benzyldimethylsilyl)ethynyl)-2-((*tert*-butyldimethylsilyl)oxy)-8-methylundec-9-en-3-yn-1-yl)-5,5-dimethyl-6a-(prop-2-yn-1-yl)tetrahydrofuro[3,2-*b*]furan-2(5*H*)-one, S19

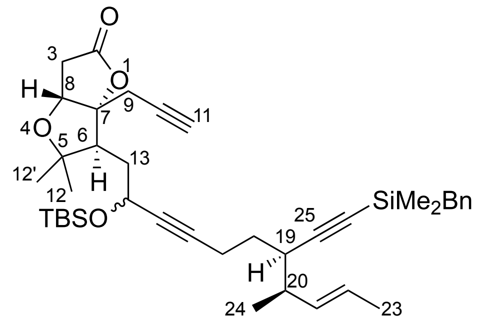

To a stirred solution of alcohol **24** (20.0 mg, 0.0367 mmol, 1.0 equiv.) in CH_2Cl_2 (0.4 mL) under Ar was added sequentially imidazole (3.7 mg, 0.0551 mmol, 1.5 equiv.), DMAP (0.4 mg, 0.004 mmol, 0.1 equiv.) and TBSCl (6.6 mg, 0.0441 mmol, 1.2 equiv.). The reaction mixture was stirred at rt for 6 h before being quenched with $NaHCO_3$ (0.4 mL, sat., aq.). The layers were separated and the aqueous phase was extracted with dichloromethane (3 x 0.4 mL). The combined organic phases were dried (Na_2SO_4) and concentrated. The product was purified by flash chromatography (9:1 petroleum ether / EtOAc) to afford **S19** (23.7 mg, 2:1 mixture of diastereomers, 0.0360 mmol, 98%) as a colourless oil. **R_f** 0.19 (9:1 petroleum ether / EtOAc); **IR** (thin film, ν_{max} / cm^{-1}) 2957, 2929, 2166, 1790, 1494, 1250, 1195, 1083, 930, 836, 779; **^{1}H NMR** (400 MHz, $CDCl_3$) δ_H 7.21 (2H, t, *J* = 7.6 Hz, ArH), 7.10-7.05 (3H, t, *J* = 6.4 Hz, ArH), 5.44 (1H, dq, *J* = 15.0 and 6.1 Hz, H22), 5.30 (1H, ddd, *J* = 15.0, 8.3 and 1.2 Hz, H21), 4.48 (1H, d, *J* = 6.7 Hz, H8), 4.43-4.35 (1H, m, H14), 2.99 (1 H, dd, *J* = 18.6 and 7.0 Hz, H3), 2.90 (1H, dd, *J* = 16.9 and 2.6 Hz, H9), 2.66 (1H, d, *J* = 18.6, H3),), 2.48 (1H, d, *J* = 16.9, H9), 2.45 (1H, dd, *J* = 7.5 and 3.2 Hz, H6), 2.43-2.29 (2H, m, H19 and H17), 2.24 (1H, ddd, *J* = 16.6, 7.9 and 1.2 Hz, H17), 2.17 (1.33H, s, $SiC\underline{H}_2Ph^{maj}$), 2.12 (1H, qd, *J* = 7.2 and 2.8 Hz, H20), 2.07 (0.67H, s, $SiC\underline{H}_2Ph^{min}$), 1.73 (1H, ddd, *J* = 14.1, 9.8 and 3.7 Hz, H13), 1.69-1.62 (1H, m, H18), 1.66 (3 H, d, *J* = 6.1 Hz, H23), 1.64-1.45 (2H, m, H13 and H18), 1.34 (1H, s, $H12^{min}$), 1.31 (2 H, s, $H12^{maj}$), 1.07-1.04 (6H, m, H12 and H24), 0.92 (3H, s, $SiC(CH_3)_3{}^{min}$), 0.92 (6H, s, $SiC(CH_3)_3{}^{maj}$), 0.21-0.13 (6H, m, $OSi(C\underline{H}_3)_2{}^tBu$), 0.11 (6H, app d, *J* = 1.8 Hz, $Si(CH_3)_2Bn$); **^{13}C NMR** (101MHz, $CDCl_3$) δ_C 175.6 ($C2^{min}$), 175.4 ($C2^{maj}$), 139.4 (C_{Ar}), 135.0 (C21), 128.5 (C_{Ar}), 128.2 (C_{Ar}), 125.1 (C22), 124.4 (C_{Ar}), 109.7 (C25), 94.7 ($C7^{min}$), 94.6 ($C7^{maj}$), 85.8 (C26), 85.5 (C16), 83.7 ($C5^{min}$), 83.5 ($C5^{maj}$), 81.4 ($C15^{maj}$), 81.2 ($C15^{min}$), 78.9 (C8), 78.1 (C10), 72.4 (C11), 62.6 ($C14^{min}$), 61.2 ($C14^{maj}$), 55.5 ($C6^{min}$), 54.5 ($C6^{maj}$), 40.9 (C20), 38.3 (C19), 37.7 (C3), 35.6 ($C13^{min}$), 35.4 ($C13^{maj}$), 31.9 (C18), 27.8 ($C12^{min}$), 27.7 ($C12^{maj}$) 26.7 ($Si\underline{C}H_2Ph$), 26.1 (C9), 26.0 ($SiC(\underline{C}H_3)_3$, 20.7 (C12), 18.3 ($OSi\underline{C}(CH_3)_3$), 18.1 (C24), 18.1 (C23), 17.0 (C17), –1.6 ($Si(CH_3)_2Bn$), –4.1 ($OSi(\underline{C}H_3)_2{}^tBu^{maj}$), –4.2 ($OSi(\underline{C}H_3)_2{}^tBu^{min}$), –4.6 ($OSi(\underline{C}H_3)_2{}^tBu^{min}$), –4.9 ($OSi(\underline{C}H_3)_2{}^tBu^{maj}$); **HRMS** ($ES^+$) calc. for $C_{40}H_{58}NaO_4Si_2$ $[M+Na]^+$ 681.3766; found 681.3764.

(3a*R*,5a*S*,10*R*,13a*R*)-11-(Benzyldimethylsilyl)-7-((*tert*-butyldimethylsilyl)oxy)-5,5-dimethyl-10-((*R,E*)-pent-3-en-2-yl)-3,3a,5,5a,6,7,8,9,10,13-decahydro-2*H*furo[3,2-b]indeno[4',5':5,6]cyclohepta[1,2-c]furan-2-one, 22

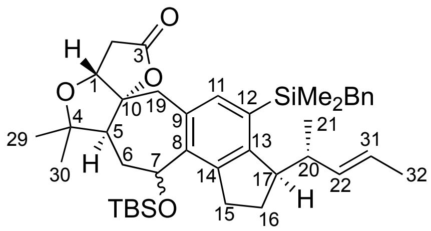

22

To a solution of triyne **S19** (23.7 mg, 0.036 mmol, 1.0 equiv) in chlorobenzene (0.9 mL, 0.04 M) in a microwave tube was added triphenylphosphine (9.4 mg, 0.036 mmol, 1 equiv.) and the mixture was degassed with Ar bubbling for 30 min. $CpCo(CO)_2$ (3.2 mg, 0.018 mmol, 0.5 equiv.) was added and the reaction mixture was heated in a microwave (300 W) at 150 °C for 25 min. Upon cooling, the reaction mixture was concentrated, and the product was purified by flash chromatography (9:1 petroleum ether / EtOAc) to give pentacycle **22** (17.9 mg, as a 31:40:29 inseparable mixture of diastereomers along with terminal alkene side product, 54% of desired product by ^{1}H NMR) as a colourless oil. The data for **22** were identical to that recorded from the Pd-catalyzed route.

1.2.5 Synthesis of rubriflordilactone A from intermediate 23

(3a*R*,5a*S*,10*R*,13a*R*)-10-((2*S*)-3,4-Dihydroxypentan-2-yl)-11-hydroxy-5,5-dimethyl-3,3a,5,5a,6,7,8,9,10,13-decahydro-2*H*-furo[3,2-b]indeno[4',5':5,6]cyclohepta[1,2-c]furan-2-one, S20

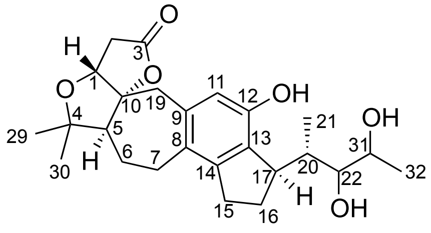

To a stirred solution of alkene **23** (8.8 mg, 0.022 mmol, 1.0 equiv.) in acetone (0.17 mL) and water (55 µL) was sequentially added OsO_4 (4% wt in water, 5.6 μL, 0.009 mmol, 0.04 equiv.) and NMO (50% wt in water, 7.8 μL, 0.033 mmol, 1.5 equiv.). The reaction mixture was stirred at RT for 2 h before being quenched with sat. *aq*. $Na_2S_2O_3$ solution (0.3 mL). The layers were separated and the aqueous layer extracted three times with ethyl acetate (0.3 mL ×3). The combined organic layers were dried with $MgSO_4$ and the solvent removed carefully *in vacuo*. The crude product was purified by flash column chromatography on a short plug of silica (49:1 CH_2Cl_2/ MeOH, then concentrated to yield triols **S20** (8.1 mg, as a 1:1 mixture of diastereomers, 85%) as a white foam. These compounds were generally carried forward as a mixture to the next step, but could be separated by careful chromatography for the purposes of characterization.

IR (thin film, ν_{max} / cm^{-1}) 3341 (br), 2923 (s), 1773 (s), 1586 (m), 1458 (m), 1373 (m), 1227 (m), 1202 (s), 1107 (m), 1032 (m), 934 (m); **HRMS** (ESI$^+$) calc. for $C_{25}H_{34}NaO_6$ $[M+Na]^+$ 453.2248, found 453.2248.

Diastereomer 1: R$_f$ 0.34 (19:1 CH_2Cl_2 / MeOH); **^{1}H NMR** (500 MHz, $CDCl_3$) δ_H 8.33 (1H, br s, ArO$\underline{H}$), 6.31 (1H, s, H11), 4.26 (1H, d, *J* = 6.1 Hz, H1), 3.98 (1H, qd, *J* = 5.9 and 2.5 Hz, H31), 3.64 (1H, dt, *J* = 9.8 and 2.5 Hz, H17), 3.51 (1H, d, *J* = 15.5 Hz, H19), 3.13 (1H, dd, *J* = 8.2 and 2.5 Hz, H22), 2.95 (1H, ddd, J = 17.0, 5.4 and 1.9 Hz, H7), 2.86 (1H, dd, *J* = 18.6 and 6.3 Hz, H2), 2.79 (1H, dd, *J* = 16.0 and 8.3 Hz, H15), 2.75-2.71 (1H, m, H7), 2.70 (1H, d, *J* = 18.6 Hz, H2), 2.66 (1H, d, *J* = 15.5 Hz, H19), 2.60 (1H, ddd, *J* = 16.0, 9.7 and 1.8 Hz, H15), 2.37 (1H, app dq, *J* = 13.0 and 9.7 Hz, H16), 2.28 (1H, dd, *J* = 12.7 and 3.5 Hz, H5), 2.11-2.04 (1H, m, H20), 1.99 (1H, app qd, *J* = 13.1 and 2.4 Hz, H6), 1.84 (1H, ddt, *J* = 13.0, 8.2 and 2.2 Hz, H16), 1.75 (1H, ddt, *J* = 14.3, 5.8 and 2.8 Hz, H6), 1.60 (1H, br s, OH), 1.55 (1H, s, OH), 1.36 (3H, s, H29), 1.29 (3H, d, *J* = 6.3 Hz, H32), 1.15 (3H, s, H30), 0.76 (3H, d, *J* = 6.9 Hz, H21); **^{13}C NMR** (126 MHz, $CDCl_3$) δ_C 175.4 (C3), 151.9 (C12), 147.5 (C14), 132.9 (C8), 126.4 (C13), 124.9 (C9), 117.8 (C11), 99.1 (C10), 84.4 (C4), 79.7 (C1), 78.7 (C22), 67.5 (C31), 59.6 (C5), 42.1 (C20), 41.3 (C17), 40.8 (C19), 36.0 (C2), 32.7 (C16), 32.2 (C15), 31.1 (C7), 28.4 (C29), 24.1 (C6), 21.2 (C30), 21.0 (C32), 12.6 (C21).

Diastereomer 2: R$_f$ 0.32 (19:1 CH_2Cl_2 / MeOH); **^{1}H NMR** (500 MHz, $CDCl_3$) δ_H 8.37 (1H, br s, ArOH), 6.31 (1H, s, H11), 4.26 (1H, d, *J* = 6.1 Hz, H1), 3.73 (1H, dq, *J* = 8.2 and 6.2 Hz, H31), 3.57 (1H, dd, *J* = 8.2 and 2.2 Hz, H22), 3.52 (1H, d, *J* = 15.4 Hz, H19), 3.34-3.29 (1H, m, H17), 2.98 (1H, ddd, *J* = 17.0, 5.7 and 2.5 Hz, H7), 2.86 (1H, dd, *J* = 18.6 and 6.2 Hz, H2), 2.81-2.73 (1H, m, H15), 2.73-2.70 (1H, m, H15), 2.69 (1H, d, *J* = 18.6 Hz, H2), 2.64 (1H, d, *J* = 15.4 Hz, H19), 2.57 (1H, ddd, *J* = 13.4, 9.0 and 3.8 Hz, H15), 2.38 (1H, dq, *J* = 12.5 and 8.8 Hz, H16), 2.27 (1H, dd, *J* = 12.7 and 3.3 Hz, H5), 2.06-1.92 (2H, m, H6 and H20), 1.86 (1H, ddd, *J* = 12.5, 8.0 and 3.8 Hz, H16), 1.75 (1 H, ddt, *J* = 11.4, 5.2 and 2.6 Hz, H6), 1.36 (3H, s, H29), 1.22 (3H, d, *J* = 6.2 Hz, H32), 1.15 (3H, s, H30), 0.72 (3H, d, *J* = 7.3 Hz, H21); **^{13}C NMR** (126 MHz, $CDCl_3$) δ_C 175.5 (C3), 151.6 (C12), 147.1 (C14), 132.8 (C8), 128.4 (C13), 125.3 (C9), 118.4 (C11), 99.2 (C10), 84.4 (C4), 81.3 (C22), 79.7 (C1), 69.5 (C31), 59.5 (C5), 47.1 (C17), 40.7 (C19), 40.3 (C20), 36.0 (C2), 35.6 (C16), 31.3 (C7), 31.1 (C15), 28.5 (C29), 24.1 (C6), 21.2 (C30), 19.7 (C32), 9.6 (C21).

Equilibrium mixture of aldehyde (*S*)-2-((3a*R*,5a*S*,10*R*,13a*R*)-11-hydroxy-5,5-dimethyl-2-oxo-3,3a,5,5a,6,7,8,9,10,13-decahydro-2*H*-furo[3,2-b]indeno[4',5':5,6]cyclohepta[1,2-c]furan-10-yl)propanal, and lactols (3a*R*,5a*S*,9a*R*,10*S*,14a*R*)-11-Hydroxy-5,5,10-trimethyl-3,3a,5,5a,6,7,8,9,9a,10,11,14-dodecahydro-2*H*-cyclopenta[de]furo[3'',2'':2',3']furo[3',4':4,5] cyclohepta[1,2-g]chromen-2-one, 26

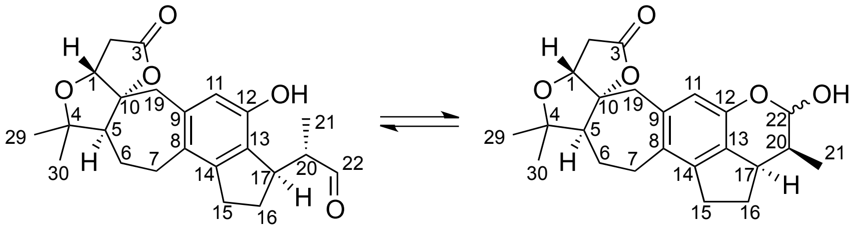

To a stirred solution of diol **S20** (22.9 mg, 0.053 mmol, 1.0 equiv.) in CH_2Cl_2 (0.6 mL) was added $NaIO_4$ supported on silica gel (10 wt% , 137 mg, 0.0638 mmol, 1.2 equiv.).[8] The suspension was stirred for 15 min before being loaded onto a short plug of silica and purified by flash column chromatography (99:1 CH_2Cl_2/MeOH), then concentrated to yield lactol **26** (20.5 mg, 0.053 mmol, quant., as a 56:33:11

inseparable equilibrium mixture of the major lactol epimer, the open-chain aldehyde form, and the minor lactol epimer, respectively) as a colourless oil. **R*f*** 0.44 (19:1 CH_2Cl_2 / MeOH); **IR** (thin film, ν_{max} / cm^{-1}) 3380, 2925, 1774, 1611, 1460, 1374, 1320, 1229, 1201, 1059, 1023, 934; **^{1}H NMR** (500 MHz, $CDCl_3$) δ_H 9.64 (0.33H, s, H22^{ald}), 6.24 (0.56H, s, H11^{maj}), 6.24 (0.56H, s, H11^{ald}), 6.22 (0.11H, s, H11^{min}), 5.42 (0.11 H, d, *J* = 4.6 Hz, H22^{min}), 5.37 (0.56H, s, H22^{maj}), 6.06 (0.33H, s, ArOH^{ald}), 4.26 (0.67H, d, *J* = 6.1 Hz, H1$^{maj\&min}$), 4.25 (0.33H, d, *J* = 6.1 Hz, H1^{ald}), 3.73 (0.33H, dt, *J* = 7.1, 3.3 Hz, H17^{ald}), 3.54 (0.56H, d, *J* = 15.7 Hz, H19^{maj}), 3.52 (0.11H, d, *J* = 15.8 Hz, H19^{min}),3.50 (0.33H, d, *J* = 15.8 Hz, H19^{ald}), 3.42 (0.33H, dt, *J* = 11.7 and 6.1 Hz, H17^{ald}), 3.35 (0.11H, dt, *J* = 11.3 and 5.9 Hz, H17^{min}), 3.11 (0.56H, ddd, *J* = 17.2, 5.6 and 2.3 Hz, H7^{maj}), 3.11-3.05 (0.11H, m, H7^{min}), 3.05 (0.56H, br s, OH^{maj}), 2.96 (0.33H, ddd, *J* = 17.1, 5.4 and 1.9 Hz, H7^{ald}), 2.89 (0.33H, qd, *J* = 7.3 and 3.3 Hz, H20^{ald}), 2.85 (1H, dd, *J* = 18.6 and 6.1 Hz, H2), 2.80-2.68 (3H, m, H7 and 2 × H6), 2.69 (1H, d, *J* = 18.6 Hz, H2), 2.67 (0.67H, *J* = 15.7 Hz, H19 $^{maj\&min}$), 2.61 (1H, *J* = 15.8 Hz, H19^{ald}), 2.35 (0.33H, dt, *J* = 13.0 and 9.1 Hz, H15^{ald}), 2.32-2.23 (1.67H, m, H20 and H5), 2.17 (0.56H, ddd, *J* = 14.4, 7.1 and 3.8 Hz, H15 ^{maj}), 2.12 (0.11H, ddd, *J* = 7.0, 4.4 and 2.8 Hz, H15^{min}), 2.04-1.93 (1H, m, H16), 1.84 (0.33H, ddd, *J* = 13.0, 8.4 and 3.9 Hz, H15^{ald}), 1.80-1.70 (1.67H, m, H15$^{maj\&min}$ and H16), 1.36 (3H, s, H29), 1.15 (3H, s, H30), 1.04 (0.99H, d, *J* = 7.3 Hz, H21^{ald}), 0.76 (0.33H, d, *J* = 7.0 Hz, H21^{min}), 0.71 (1.68H, d, *J* = 7.2 Hz, H21^{maj}); **^{13}C NMR** (126 MHz, $CDCl_3$; note that ^{13}C shifts for the minor lactol diastereomer are not reported) δ_C 207.2 (C22^{ald}), 175.4 (C3), 150.5 (C12^{ald}), 146.9 (C12^{maj}), 144.6 (C14), 133.3 (C9^{ald}), 132.8 (C9^{maj}), 127.0 (C8^{ald}), 126.5 (C8^{maj}), 126.2 (C13^{maj}), 125.9 (C13^{ald}), 117.6 (C11 ^{ald}), 115.4 (C11^{maj}), 99.1 (C10), 97.5 (C22^{maj}), 84.5 (C4), 79.6 (C1), 59.4 (C5^{ald}), 59.2 (C5^{maj}), 51.4 (C20^{ald}), 42.2 (C17^{ald}), 41.0 (C19^{maj}), 40.6 (C19^{ald}), 36.0 (C2), 35.1 (C17^{maj}), 32.2 (C20^{maj}), 31.5 (C6^{ald}), 31.3 (C6^{maj}), 31.0 (C7^{ald}), 30.4 (C15^{ald}), 30.4 (C7^{maj}), 29.7 (C15^{maj}), 28.5 (C29^{maj}), 28.4 (C29^{ald}), 24.4 (C16^{maj}), 24.0 (C16^{ald}), 21.3 (C30^{maj}), 21.2 (C30^{ald}), 10.9 (C21^{maj}), 10.4 (C21^{ald}); **HRMS** (ES^-) calc. for $C_{23}H_{27}O_5$ $[M-H^+]^-$ 383.1853; found 383.1879.

(3a*R*,5a*S*,9a*R*,10*S*,14a*R*)-11-Chloro-5,5,10-trimethyl-3,3a,5,5a,6,7,8,9,9a,10,11,14-dodecahydro-2*H*-cyclopenta[de]furo[3",2":2',3']furo[3',4':4,5]cyclohepta[1,2-g]chromen-2-one, 27

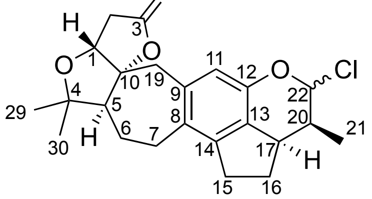

27

To an NMR tube charged with anhydrous $ZnCl_2$ (36.3 mg, 0.266 mmol, 5.0 equiv.) under Ar was added a solution of lactol-aldehyde mixture **26** (20.5 mg, 0.0532 mmol, 1.0 equiv.) in dry $CDCl_3$ (0.5 mL), followed by a solution of thionyl chloride in dry $CDCl_3$ (4.2 mM, 0.05 mL, 0.210 mmol, 4.0 equiv.). The reaction was monitored by ^{1}H NMR spectroscopy until complete conversion to the chloropyran (3 h). The mixture was then filtered through a short pad of oven-dried Celite®, and washed with dry toluene. The filtrate was concentrated to give chloropyran **27** as a colourless foam, which was used in the next step without further purification; N.B. This compound is unstable to water (in the presence of $ZnCl_2$) and silica. **^{1}H NMR** (500 MHz, $CDCl_3$) δ_H 6.32 (1H, s, H11), 6.15 (1H, d, *J* = 1.8 Hz, H22), 4.26 (1H, d, *J* = 6.1 Hz, H1), 3.72 (1H,

dt, J = 11.7, 6.1 Hz, H17), 3.53 (1H, d, J = 15.5 Hz, H19), 3.12 (1H, m, J = 17.4, 5.8, 2.7 Hz, H7), 2.85 (1H, dd, J = 18.6, 6.3 Hz, H2), 2.79-2.71 (3H, m, H7 and 2×H15), 2.72-2.68 (2H, m, H2 and H19), 2.50 (1H, qdd, J = 7.2, 5.4, 1.8 Hz, H20), 2.29 (1H, dd, J = 12.6, 3.6 Hz, H5), 2.26- 2.20 (1H, m, H16), 1.98 (1H, dtd, J = 14.8, 12.6, 2.5 Hz, H6), 1.82-1.71 (2H, m, H6 and H16), 1.36 (3H, s, H29), 1.15 (3H, s, H30), 0.82 (3H, d, J = 7.2 Hz, H21); **^{13}C NMR** (126 MHz, $CDCl_3$) δ_C 175.4 (C3), 145.5 (C12), 144.9 (C14), 133.3 (C9), 128.2 (C8), 125.4 (C13), 116.0 (C11), 98.9 (C10), 94.0 (C22), 84.5 (C4), 79.6 (C1), 59.4 (C5), 41.0 (C19), 36.9 (C20), 36.0 (C2), 35.5 (C17), 31.3 (C15), 30.5 (C7), 30.3 (C16), 28.5 (C29), 24.0 (C6), 21.2 (C30), 12.3 (C21).

(3a*R*,3a'*R*,5a*S*,5a'*S*,9a*R*,9a'*R*,10*S*,10'*S*,14a*R*,14a'*R*)-11,11'-oxybis(5,5,10-trimethyl-3,3a,5,5a,6,7,8,9,9a,10,11,14-dodecahydro-2*H*-cyclopenta[*de*]furo[3'',2'':2',3']furo[3',4':4,5]cyclohepta[1,2-*g*]chromen-2-one), 28

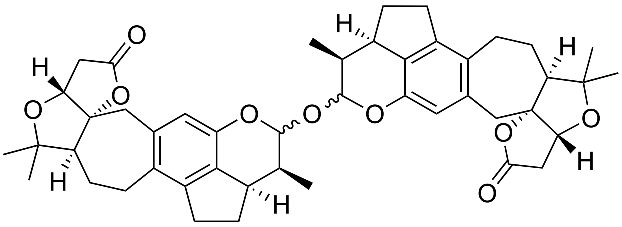

This dimer was observed spectroscopically as indicated in the ^{1}H NMR spectra below. HRMS on an aliquot showed a peak at the required mass. Model studies in which similar dimers had also been observed and isolated will be reported in due course.

HRMS (ES$^+$) calc. for $C_{46}H_{54}O_9{}^{23}Na$ [M+Na]$^+$ 773.3660; found 773.3656.

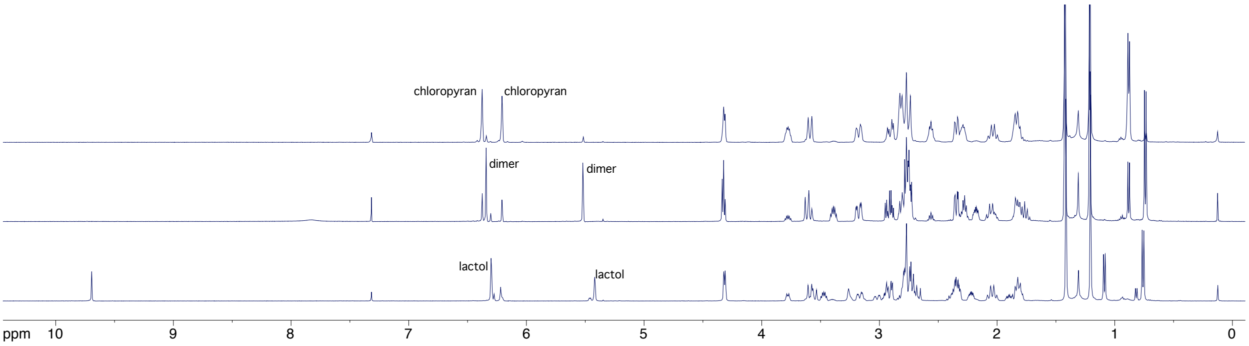

Rubriflordilactone A, 1, and C23-*epi*-rubriflordilactone A, 30

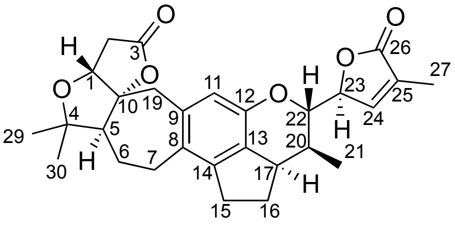

1

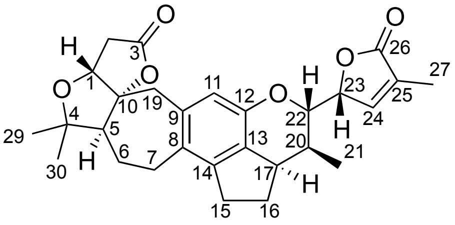

30

An oven-dried flask equipped with a stirrer bar and charged with anhydrous $ZnCl_2$ (2.9 mg, 0.021 mmol, 0.4 equiv.) was heated to 150 °C under vacuum overnight to remove any traces of water, cooled to rt and refilled with argon, then cooled to –30 °C. In a separate flask, to a solution of crude chloropyran **27** in CH_2Cl_2 (0.8 mL) under argon at –30 °C was added triisopropyl((3-methylfuran-2-yl)oxy)silane **30** (27.1

mg, 0.107 mmol, 2.0 equiv.). The resulting solution was transferred to the flask containing $ZnCl_2$, and the reaction mixture was stirred, warming to rt, overnight. The reaction mixture was then filtered through a short pad of Celite®, and washed with CH_2Cl_2. The solvent was removed *in vacuo* and the crude product was purified by flash chromatography (9:1 → 4:1 → 1:1 petroleum ether / EtOAc) to yield rubriflordilactone A **1** (9.3 mg, 0.020 mmol, 38%) as a white solid and its C-23 epimer **30** (8.2 mg, 0.018 mmol, 33%) as a white solid.

Data for rubriflordilactone A (1): $[\alpha]_D^{25}$ +58.3 (*c* = 0.114, MeOH); R_f 0.28 (1:1 petroleum ether / EtOAc); **IR** (thin film, ν_{max} / cm^{-1}) 2970, 2341, 2327, 1760, 1610, 1479, 1199, 1025, 934, 654; **^{1}H NMR** (500 MHz, Pyr) δ_H 7.30 (1H, s, H24), 6.52 (1H, s, H11), 5.07 (1H, d, *J* = 7.9 Hz, H23), 4.33 (1H, d, *J* = 6.1 Hz, H1), 4.08 (1H, dd, *J* = 7.8, 1.3 Hz, H22), 3.56 (1H, d, *J* = 15.6 Hz, H19), 3.26-3.17 (1H, m, H17), 3.20 (1H, dd, *J* = 18.3, 6.1 Hz, H2), 3.04 (1H, dd, *J* = 17.3 and 3.9 Hz, H7), 2.87 (1H, d, *J* = 15.6 Hz, H19), 2.85 (1H, d, *J* = 18.3 Hz, H2), 2.71 (1H, dd, *J* = 15.3, 8.0 Hz, H15), 2.67-2.56 (2H, m, H7 and H15), 2.38-2.31 (2H, m, H5 and H20), 2.09 (1H, dt, *J* = 11.5, 6.6 Hz, H16), 1.95-1.83 (1H, m, H6), 1.92 (3H, s, H27), 1.81-1.68 (1H, m, H16), 1.66-1.57 (1H, m, H6), 1.35 (3H, s, H30), 1.13 (3H, s, H29), 0.85 (3H, d, *J* = 7.0 Hz, H21); **^{13}C NMR** (126 MHz, Pyr) δ_c 176.0 (C3), 174.2 (C26), 148.6 (C12), 145.5 (C14), 145.5 (C24), 134.5 (C8), 132.4 (C25), 127.1 (C9), 125.2 (C13), 116.5 (C11), 99.6 (C10), 84.5 (C4), 83.9 C22), 82.6 (C23), 80.3 (C1), 60.6 (C5), 41.0 (C19), 38.2 (C17), 36.5 (C2), 31.7 (C15), 31.2 (C7), 31.2 (C16), 30.5 (C20), 28.8 (C29), 24.5 (C6), 21.4 (C30), 13.6 (C21), 11.3 (C27); **HRMS** (ES^+) calc. for $C_{28}H_{32}NaO_6$ $[M+Na]^+$ 487.2091; found 487.2092. Spectroscopic data are identical to those reported in the literature.[9],[10]

Data for C23-*epi*-rubriflordilactone A (30): $[\alpha]_D^{25}$ +64.3 (*c* = 0.114, MeOH); R_f 0.45 (1:1 petroleum ether/ EtOAc); **IR** (thin film, ν_{max} / cm^{-1}) 2954, 2361, 1756, 1613, 1485, 1198, 1062, 931, 813, 670; **^{1}H NMR** (500 MHz, $CDCl_3$) δ_H 7.32 (1H, app. quint, *J* = 1.6 Hz, H24), 6.59 (1H, s, H11), 4.90 (1H, app dquint, *J* = 9.5 and 1.7 Hz, H23), 4.36 (1H, d, *J* = 5.9 Hz, H1), 3.92 (1H, dd, *J* = 9.5 and 1.6 Hz, H22), 3.62 (1H, d, *J* = 15.6 Hz, H19), 3.34 (1H, dt, *J* = 11.5 and 6.0 Hz, H17), 3.18 (1H, dd, *J* = 18.4 and 6.1 Hz, H2), 3.03 (1H, m, H7), 2.93 (1H, d, *J* = 15.6 Hz, H19), 2.87 (1H, d, *J* = 18.4 Hz, H2), 2.68 (1H, dd, *J* = 15.4 and 8.3 Hz, H15), 2.65-2.57 (2H, m, H15 and H7), 2.55 (1H, qdd, *J* = 7.1, 5.6 and 1.5 Hz, H20), 2.37 (1H, dd, *J* = 12.6 and 3.3 Hz, H5), 2.05 (1H, dt, *J* = 6.4 and 11.9 Hz, H16), 1.93 (1H, m, H6), 1.87 (3H, app t, *J* = 1.7 Hz, H27), 1.70 (1H, ddd, *J* = 19.6, 11.3 and 8.5 Hz, H16), 1.62 (1H, ddt, *J* = 14.1, 6.1 and 3.3 Hz, H6), 1.36 (3H, s, H30), 1.14 (3H, s, H29), 0.80 (3H, d, *J* = 7.1 Hz, H21); **^{13}C NMR** (126 MHz, $CDCl_3$) δ_C 176.1 (C3), 174.3 (C26), 149.7 (C24), 149.0 (C12), 145.8(C14), 134.9 (C8), 130.2 (C25), 127.2 (C8), 125.4 (C13), 115.9 (C11), 99.8 (C10), 84.7 (C4), 82.7 (C22), 80.4 (C1), 79.5 (C23), 60.3 (C5), 41.0 (C19), 37.4 (C17), 36.5 (C2), 31.8 (C15), 31.3 (C7), 31.2 (C16), 29.5 (C20), 28.9 (C30), 24.5 (C6), 21.5 (C29), 13.4 (C21), 11.1 (C27); **HRMS** (ES^+) calc. for $C_{28}H_{32}NaO_6$ $[M+Na]^+$ 487.2091; found 487.2092.

Comparison Table of ^{1}H NMR data for rubriflordilactone A

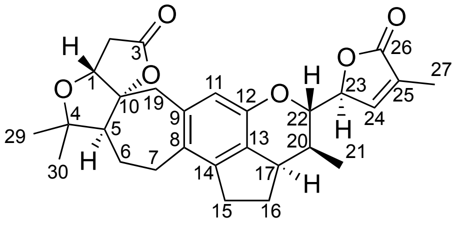

1

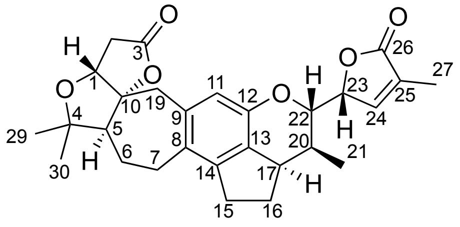

30

atom	Natural (Sun)[9] δ$_H$, [ppm, mult., *J* (Hz)] 400 MHz		Synthetic (Li)[10] δ$_H$, [ppm, mult., *J* (Hz)] 500 MHz		Synthetic (Anderson) δ$_H$, [ppm, mult., *J* (Hz)] 500 MHz	
1	4.30	1H, d, 6.1	4.32	1H, d, 6.1	4.33	1H, d, 6.1
2α	2.83	1H, d, 18.3	2.85	1H, d, 18.3	2.85	1H, d, 18.3
2β	3.19	1H, dd, 18.3, 6.1	3.21	1H, dd, 18.3, 6.1	3.21	1H, dd, 18.3, 6.2
5	2.32	1H, overlapped	2.35	1H, dd, 12.6, 2.0	2.36	1H, dd, 12.5, 3.2
6α	1.83	1H, m	1.96-1.83	1H, m	1.95-1.83	1H, m
6β	1.58	1H, m	1.65-1.58	1H, m	1.66-1.57	1H, m
7α	2.99	1H, dd, 16.2, 2.6	3.02	1H, dd, 17.3, 4.0	3.03	1H, dd, 17.3, 3.9
7β	2.71	1H, overlapped	2.70	1H, dd, 15.4, 8.2	2.70	1H, dd, 15.4, 8.5
11	6.50	1H, s	6.52	1H, s	6.52	1H, s
15α	2.59	1H, overlapped	2.67-2.57	1H, m	2.67-2.56	1H, m
15β	1.69	1H, m	1.78-1.67	1H, m	2.71	1H, dd, 15.3, 8.0
16α	2.67	1H, m	2.67-2.57	1H, m	1.81-1.68	1H, m
16β	2.06	1H, m	2.11-2.05	1H, m	2.09	1H, dt, 11.8, 6.3
17	3.21	1H, m	3.26-3.20	1H, m	3.26-3.20	1H, m
19α	2.84	1H, d, 15.6	2.87	1H, d, 15.6	2.87	1H, d, 15.6
19β	3.54	1H, d, 15.6	3.56	1H, d, 15.6	3.56	1H, d, 15.6
20	2.30	1H, m	2.34-2.29	1H, m	2.33-2.30	1H, m
21	0.82	3H, d, 7.1	0.84	3H, d, 6.8	0.85	3H, d, 7.0
22	4.05	1H, dd, 7.8, 1.3	4.08	1H, d, 8.0	4.08	1H, d, 8.0
23	4.96	1H, overlapped	5.08-5.04	1H, m	5.07	1H, d, 7.9
24	7.29	1H, br s	7.31	1H, s	7.30	1H, s
27	1.89	3H, s	1.92	3H, s	1.92	3 H, s
29	1.10	3H, s	1.13	3H, s	1.13	3H, s
30	1.32	3H, s	1.35	3H, s	1.35	3H, s

Blue highlighted rows indicate a reassignment of data based on our HSQC and COSY experiments; see the spectral data below for copies of these spectra.

Comparison Table of ^{13}C NMR data for rubriflordilactone A

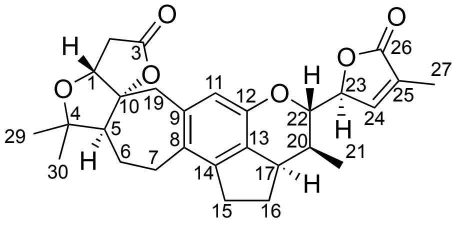

1

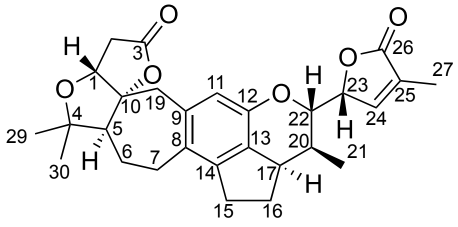

30

atom	Natural (Sun)[9] δC, [ppm] 101 MHz	Synthetic (Li)[10] δC, [ppm] 125 MHz	Synthetic (Anderson) δC, [ppm] 125 MHz
1	80.0	80.4	80.4
2	36.1	36.6	36.5
3	175.5	176.0	175.9
4	84.1	84.6	84.5
5	60.2	60.7	60.6
6	24.1	24.5	24.5
7	30.8	31.3	31.2
8	134.5	135.0	134.9
9	126.7	127.2	127.1
10	99.1	99.6	99.5
11	116.1	116.6	116.5
12	148.3	148.7	148.7
13	124.8	125.2	125.2
14	145.1	145.6	145.5
15	31.3	31.8	31.7
16	30.0	30.6	31.2
17	37.8	38.2	38.2
19	40.7	41.1	41.1
20	30.1	31.3	30.5
21	13.2	13.7	13.6
22	83.5	84.0	83.9
23	82.2	82.7	82.6
24	145.1	145.6	145.5
25	132.0	132.5	132.4
26	171.9	174.2	174.2
27	10.8	11.4	11.3
29	28.4	28.8	28.8
30	21.0	21.5	21.4

Blue highlighted rows indicate a reassignment of data based on our HSQC and COSY experiments; see the spectral data below for copies of these spectra.

2. REFERENCES

[1] A. B. Smith, R. J. Fox, J. A. Vanecko, *Org. Lett.* **2005**, *7*, 3099.

[2] M. S. Wilson, J. C. S. Woo, G. R. Dake, *J. Org. Chem.* **2006**, *71*, 4237.

[3] a) A. P. Pulis, V. K. Aggarwal, *J. Am. Chem. Soc.* **2012**, *134*, 7570; b) Z. Li, B. T. Parr, H. M. L. Davies, *J. Am. Chem. Soc.* **2012**, *134*, 10942.

[4] P. F. Godenschwager, D. B. Collum, *J. Am. Chem. Soc.* **2008**, *130*, 8726.

[5] J. A. Calderone, W. L. Santos, *Angew. Chem. Int. Ed.* **2014**, *53*, 4154.

[6] K. Morokuma, Y. Taira, Y. Uehara, S. Shibahara, K. Takahashi, J. Ishihara, S. Hatakeyama, *Tetrahedron Lett.* **2008**, *49*, 6043.

[7] a) A. Fontana, R. Messina, A. Spinella, G. Cimino, *Tetrahedron Lett.* **2000**, *41*, 7559; b) L. A. Paquette, K. W. Lai, *Org. Lett.* **2008**, *10*, 2111.

[8] Y.-L. Zhong, T. K. M. Shing, *J. Org. Chem.* **1997**, *62*, 2622.

[9] W. L. Xiao, L. M. Yang, N. B. Gong, L. Wu, R. R. Wang, J. X. Pu, X. L. Li, S. X. Huang, Y. T. Zheng, R. T. Li, Y. Lu, Q. T. Zheng, H. D. Sun, *Org. Lett.* **2006**, *8*, 991.

[10] J. Li, P. Yang, M. Yao, J. Deng, A. Li, *J. Am. Chem. Soc.* **2014**, *136*, 16477.

3. NMR SPECTRA

*3.1 Intermediates in the synthesis of diyne **7***

5-((4-methoxybenzyl)oxy)pentan-1-ol, S1

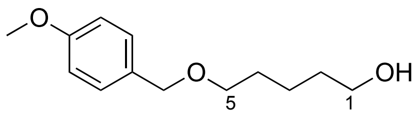

S1

^{1}H NMR (400 MHz, $CDCl_3$)

^{13}C NMR (100 MHz, $CDCl_3$)

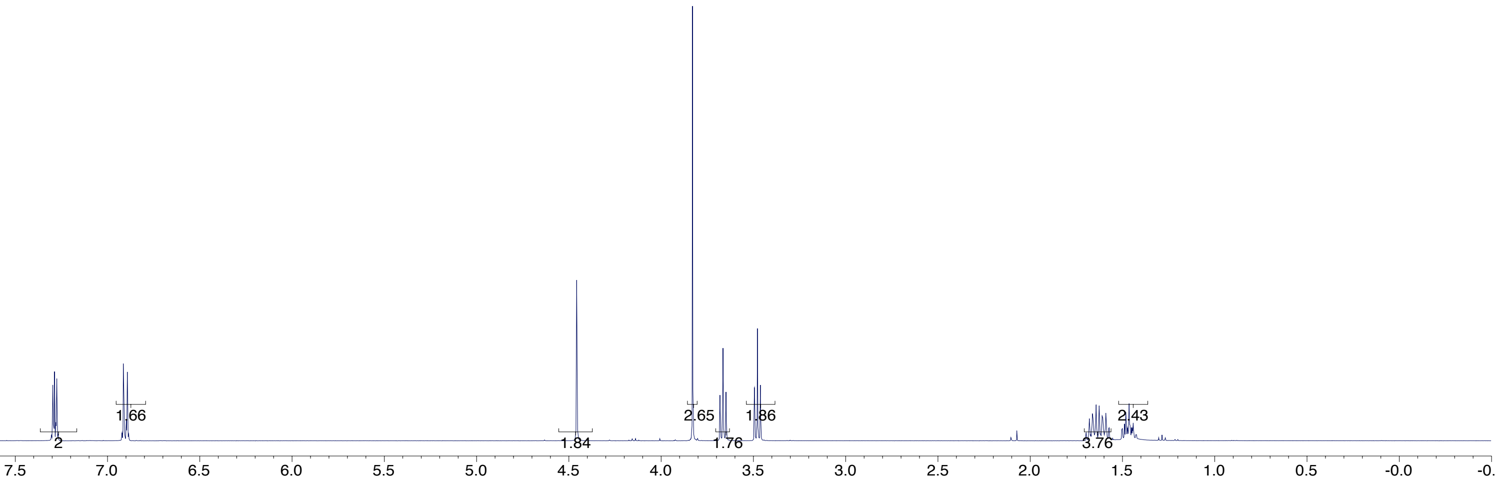

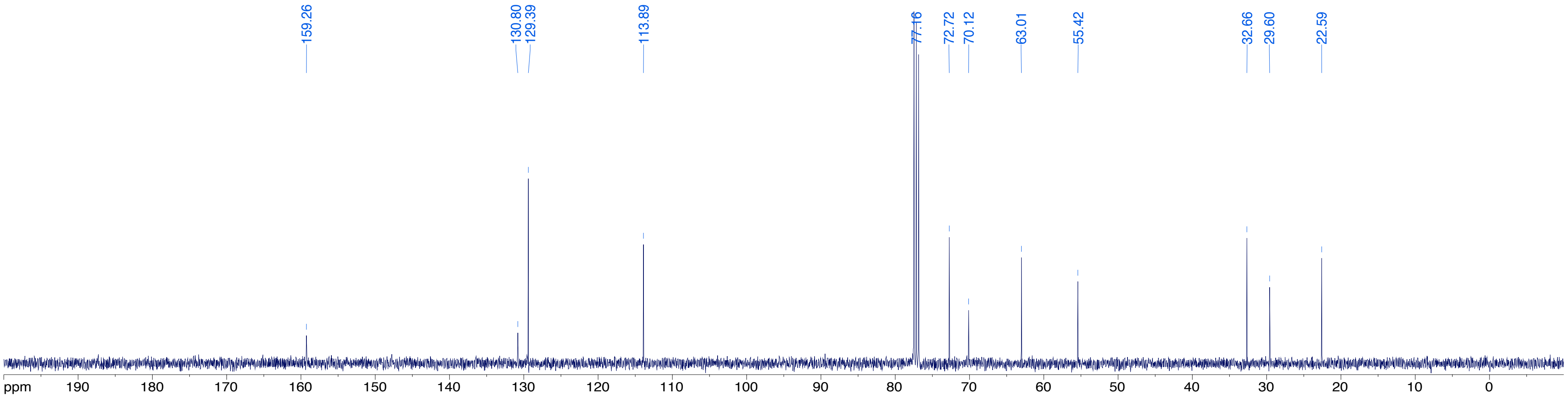

5-((4-methoxybenzyl)oxy)pentanoic acid, 9

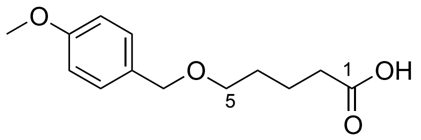

9

^{1}H NMR (500 MHz, $CDCl_3$)

^{13}C NMR (125 MHz, $CDCl_3$)

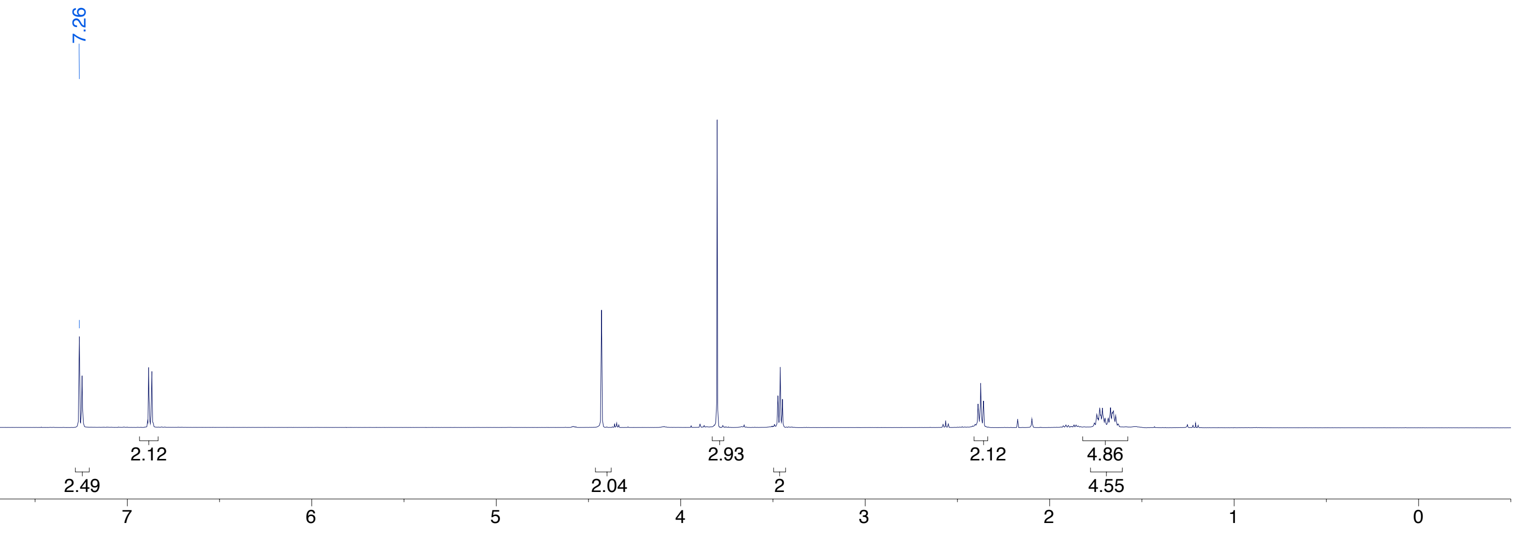

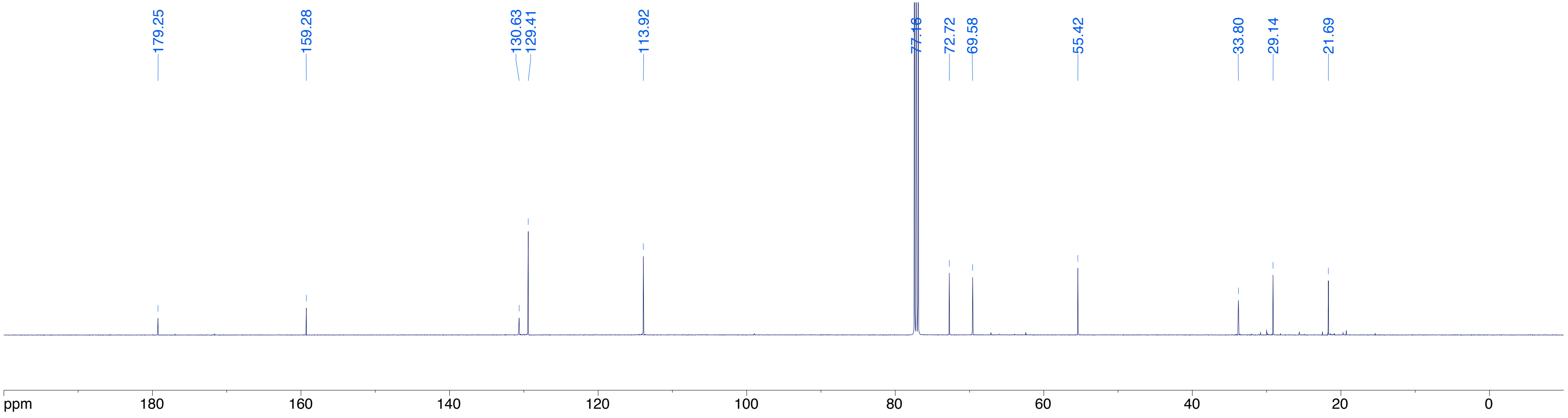

(*S*,*E*)-pent-3-en-2-yl 5-((4-methoxybenzyl)oxy)pentanoate, 10

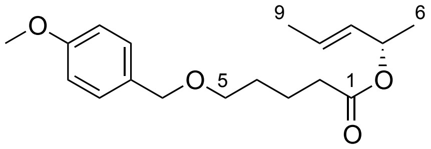

10

^{1}H NMR (400 MHz, $CDCl_3$)

^{13}C NMR (100 MHz, $CDCl_3$)

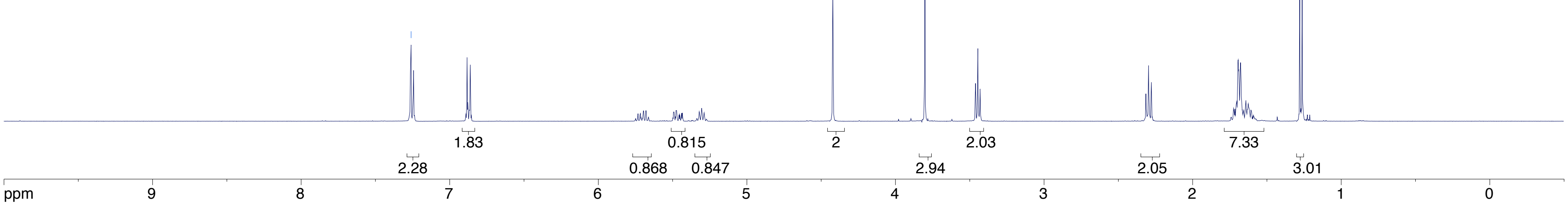

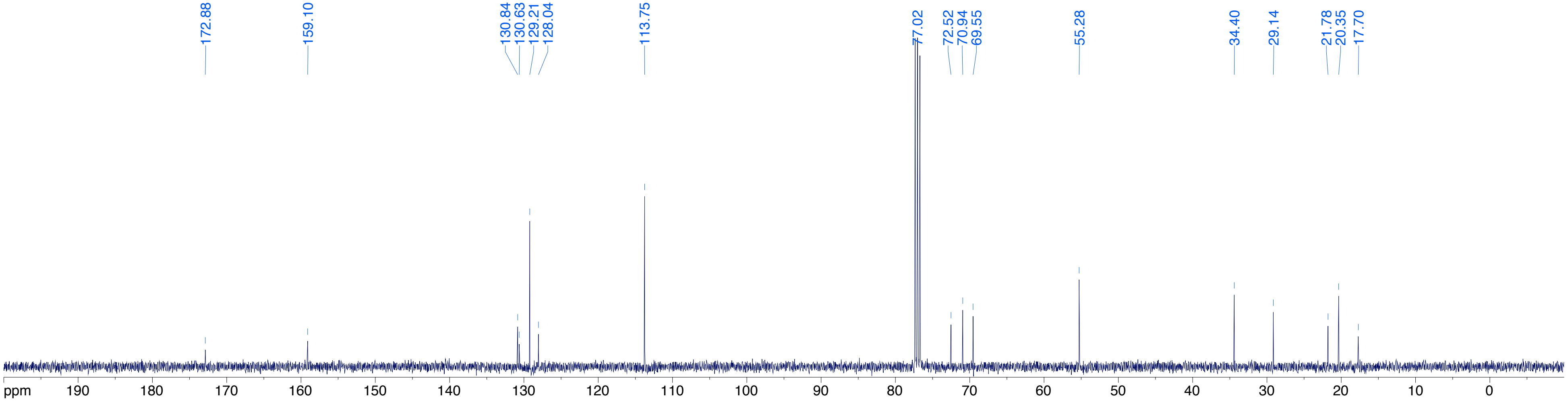

(2*R*,3*R*,*E*)-2-(3-((4-methoxybenzyl)oxy)propyl)-3-methylhex-4-enoic acid, 11

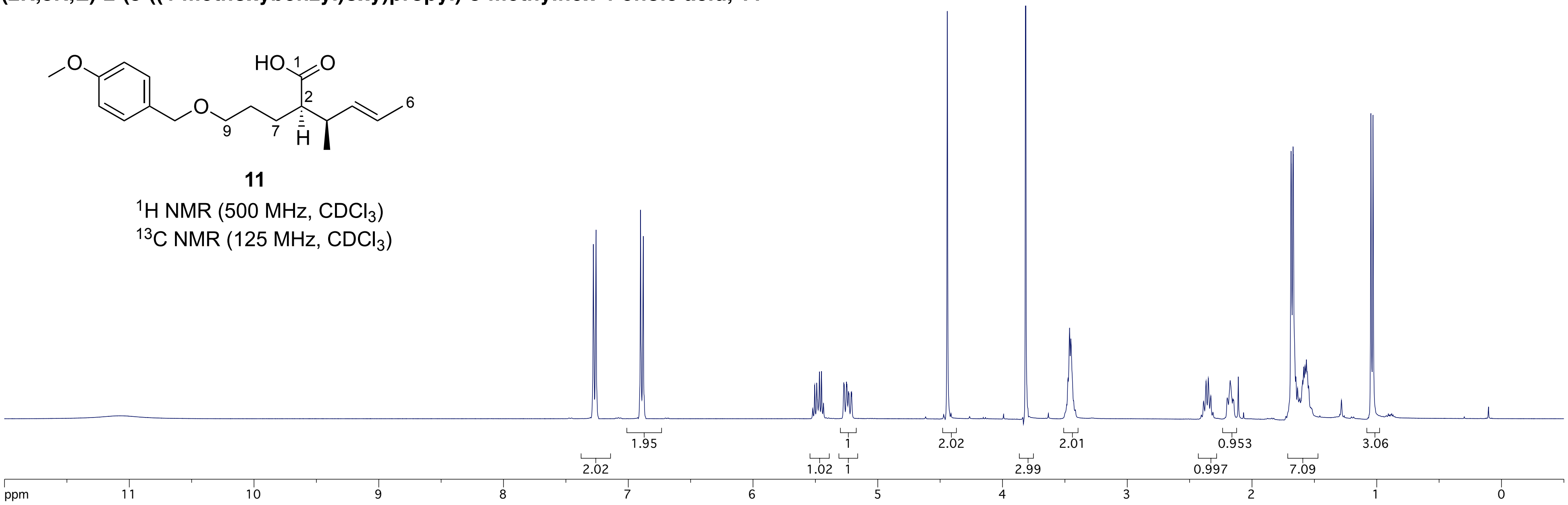

11

^{1}H NMR (500 MHz, $CDCl_3$)

^{13}C NMR (125 MHz, $CDCl_3$)

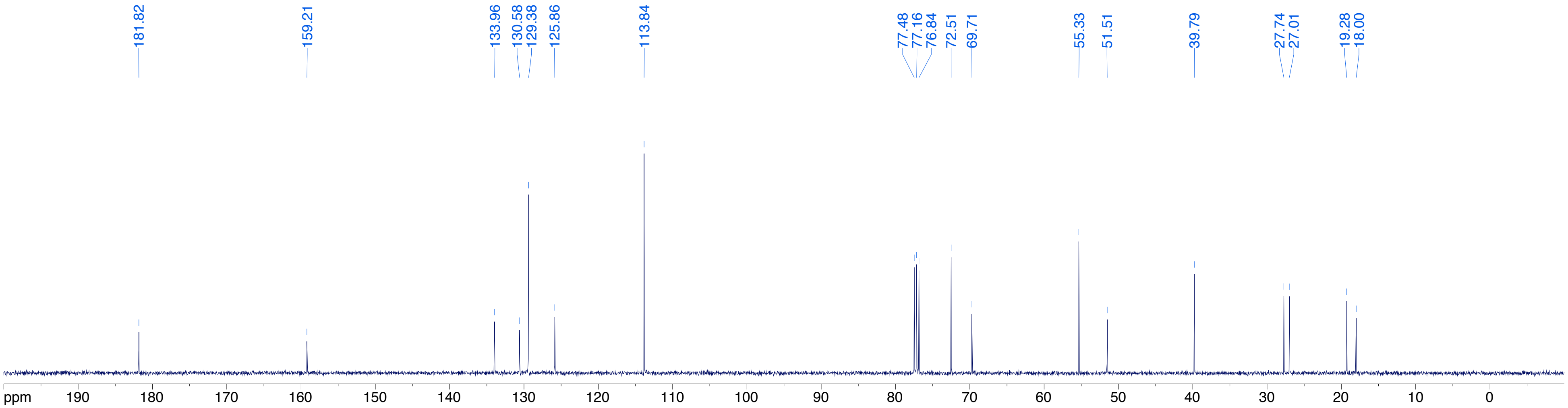

(2*R*,3*R*,*E*)-Methyl 2-(3-((4-methoxybenzyl)oxy)propyl)-3-methylhex-4-enoate, S2

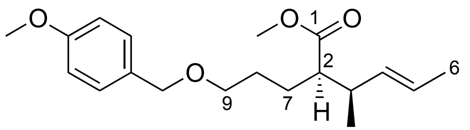

S2

^{1}H NMR (500 MHz, $CDCl_3$)

^{13}C NMR (125 MHz, $CDCl_3$)

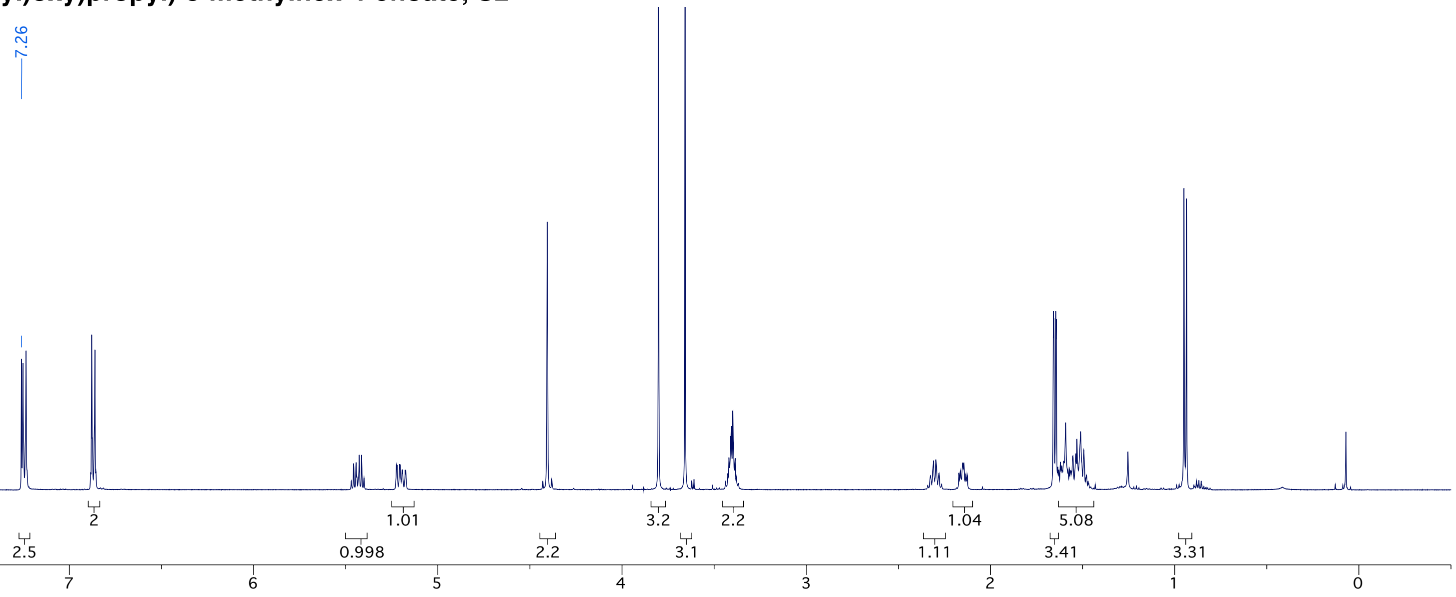

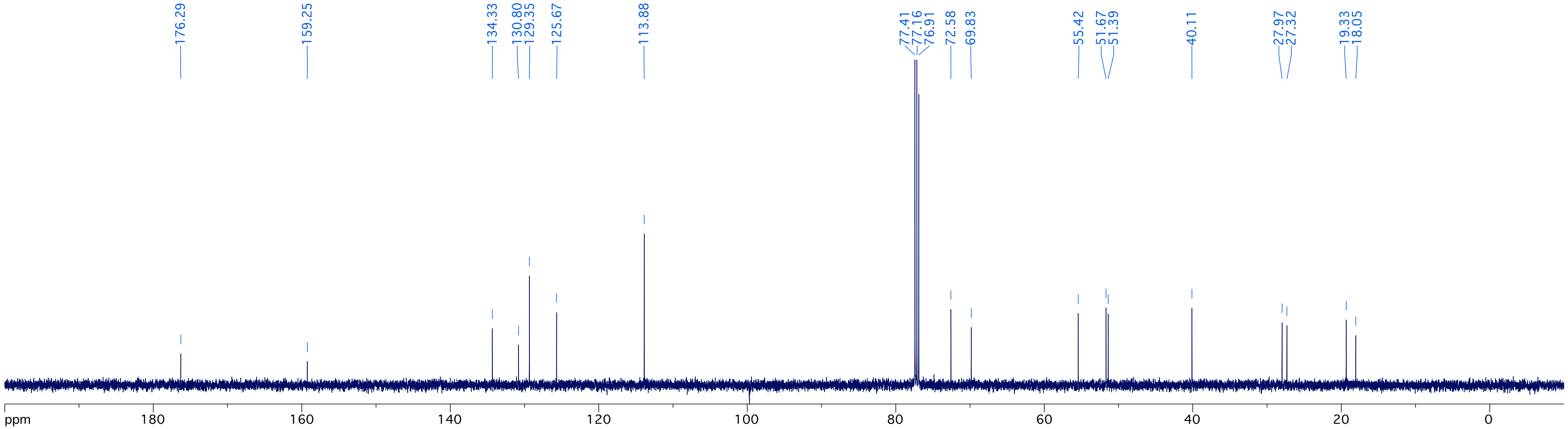

(2*R*,3*R*,*E*)-2-(3-((4-Methoxybenzyl)oxy)propyl)-3-methylhex-4-en-1-ol, S3

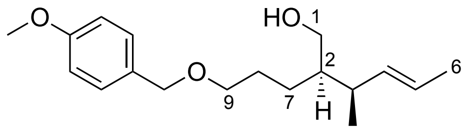

S3

^{1}H NMR (500 MHz, $CDCl_3$)
^{13}C NMR (125 MHz, $CDCl_3$)

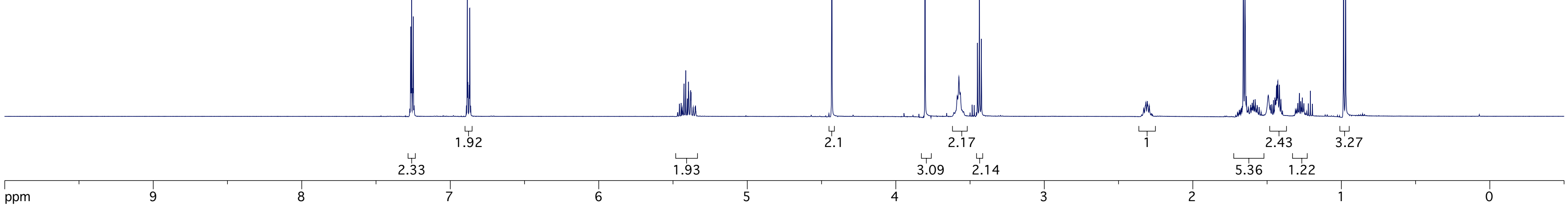

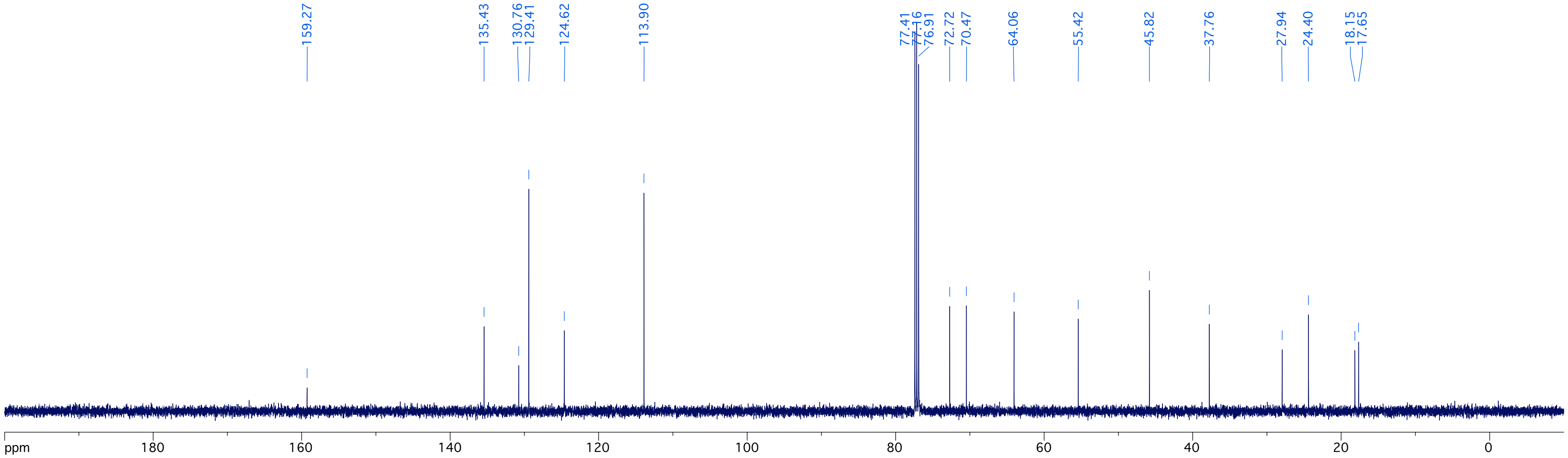

(2*R*,3*R*,*E*)-2-(3-((4-methoxybenzyl)oxy)propyl)-3-methylhex-4-enal, S4

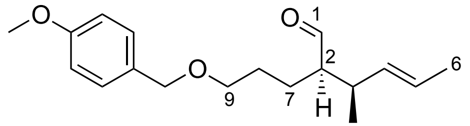

S4

^{1}H NMR (500 MHz, $CDCl_3$)

^{13}C NMR (125 MHz, $CDCl_3$)

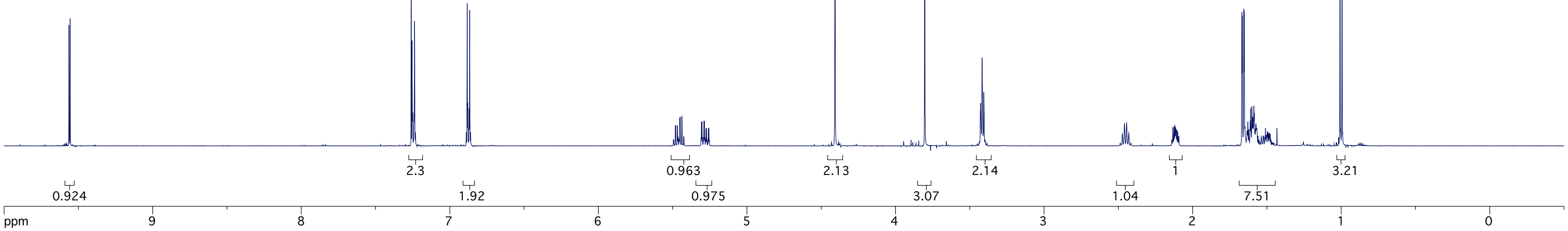

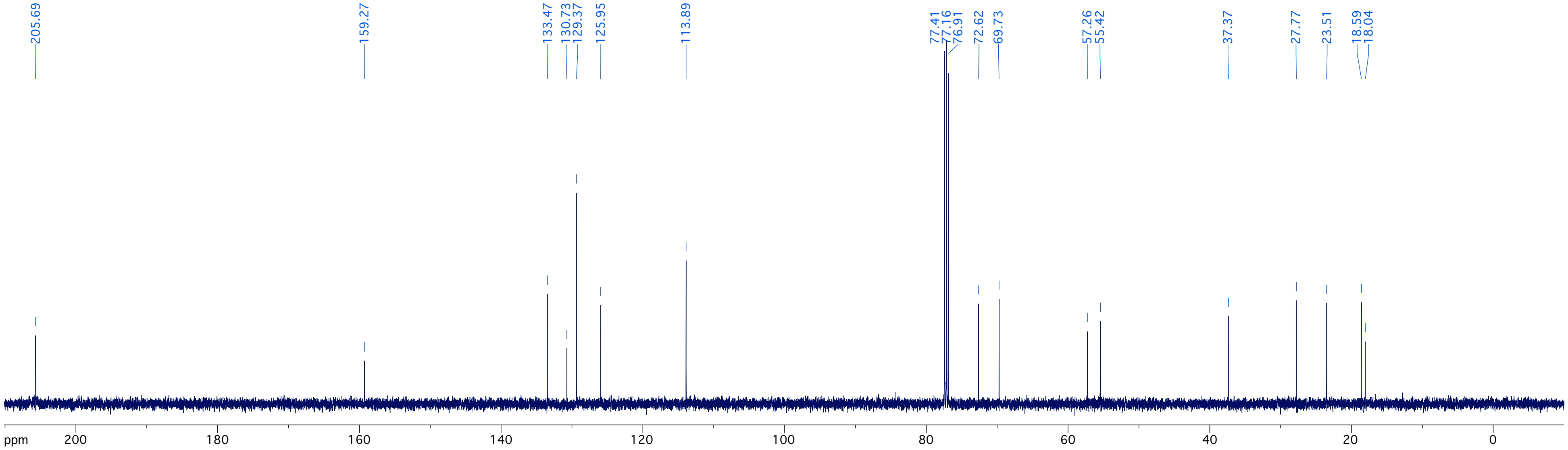

1-((((4*R*,5*R*,*E*)-4-Ethynyl-5-methyloct-6-en-1-yl)oxy)methyl)-4-methoxybenzene, S5

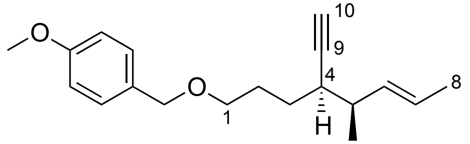

S5

^{1}H NMR (500 MHz, $CDCl_3$)

^{13}C NMR (125 MHz, $CDCl_3$)

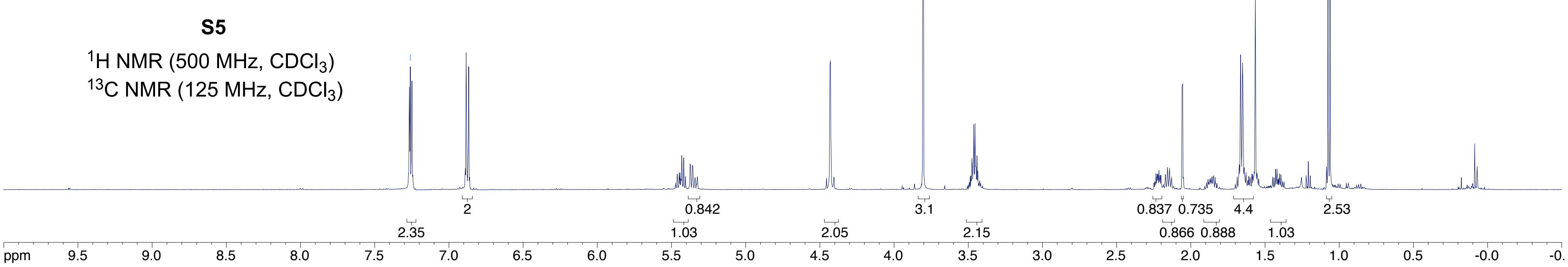

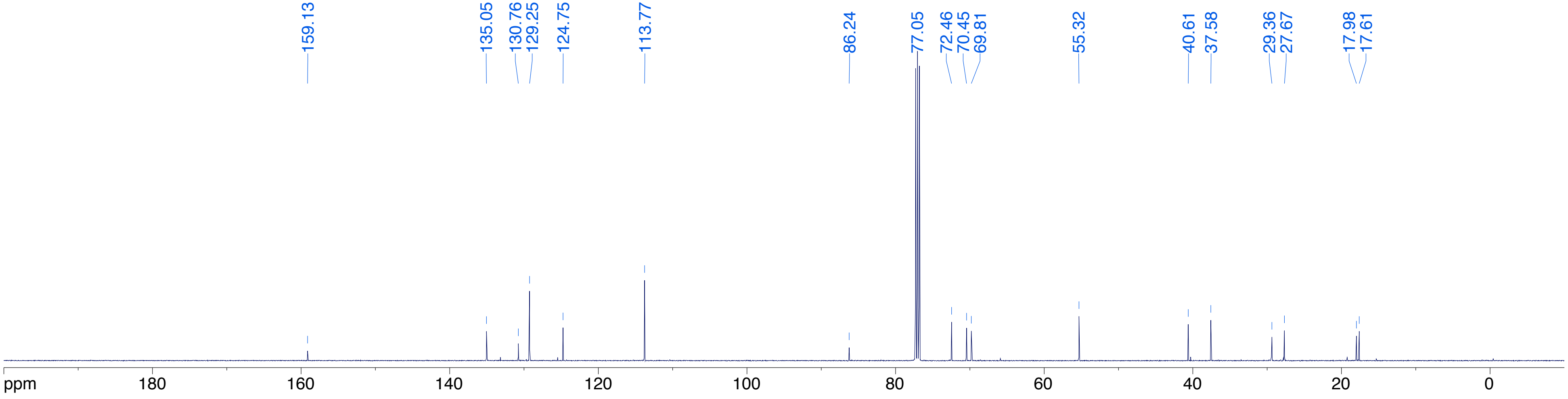

Benzyl((3*R*,4*R*,*E*)-3-(3-((4-methoxybenzyl)oxy)propyl)-4-methylhept-5-en-1-yn-1-yl)dimethylsilane 12

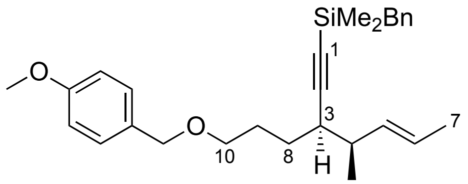

12

^{1}H NMR (500 MHz, $CDCl_3$)

^{13}C NMR (125 MHz, $CDCl_3$)

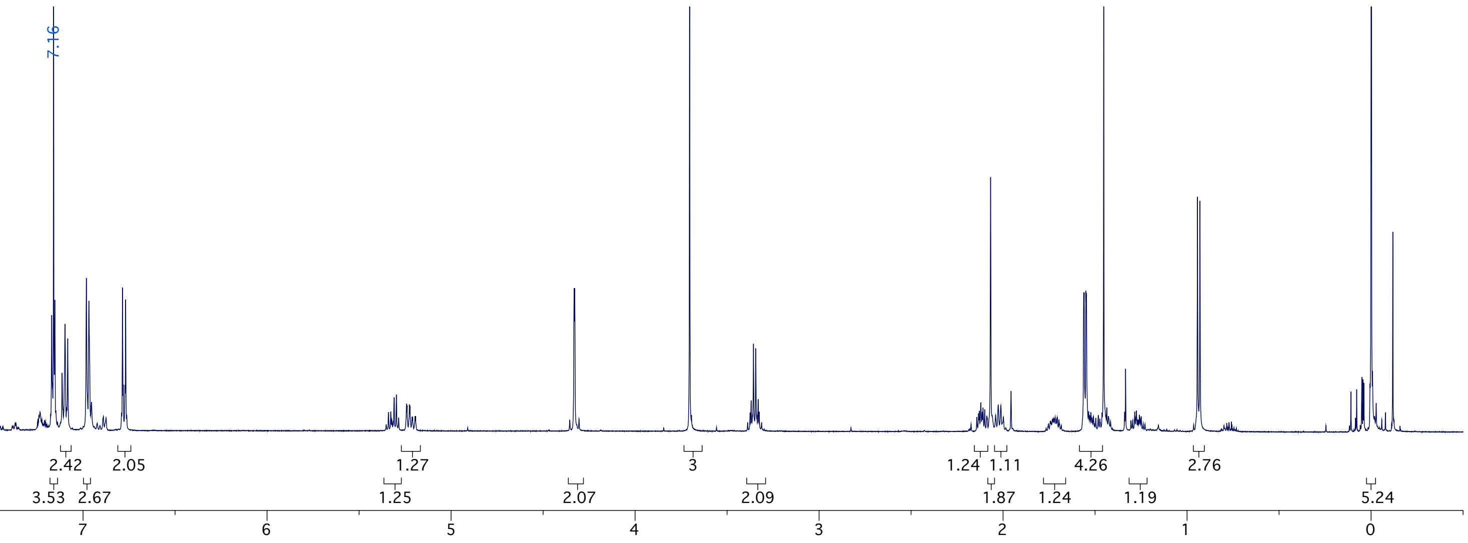

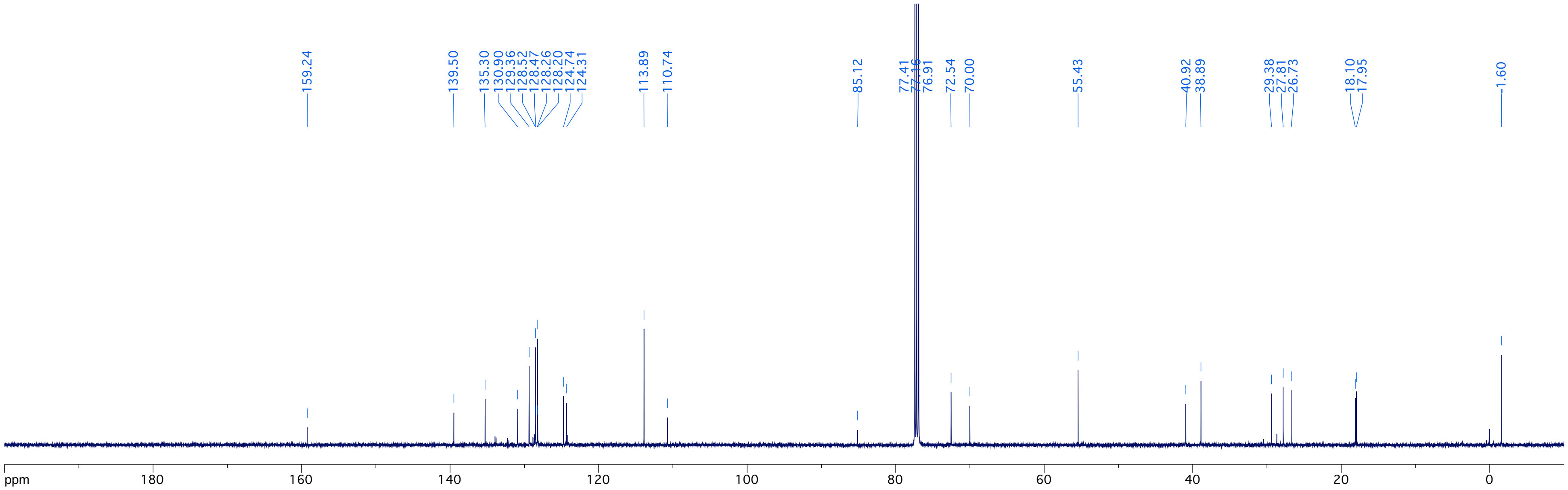

(4*R*,5*R*,*E*)-4-((Benzyldimethylsilyl)ethynyl)-5-methyloct-6-enal, S6

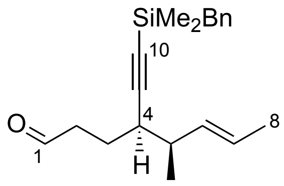

S6

^{1}H NMR (400 MHz, $CDCl_3$)

^{13}C NMR (100 MHz, $CDCl_3$)

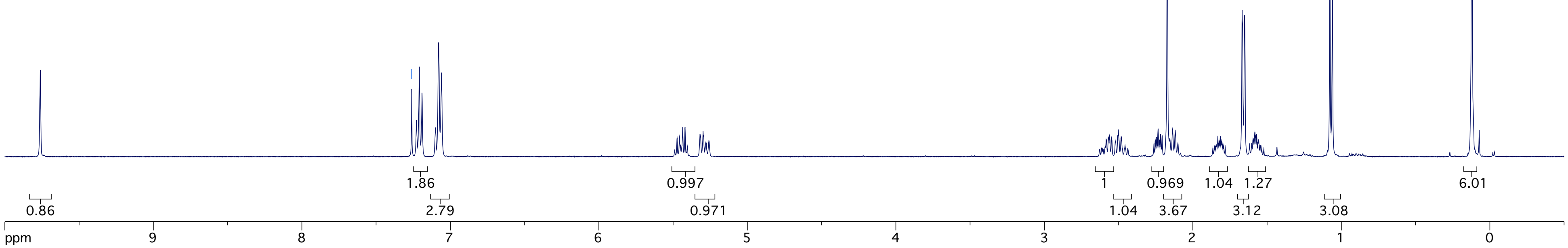

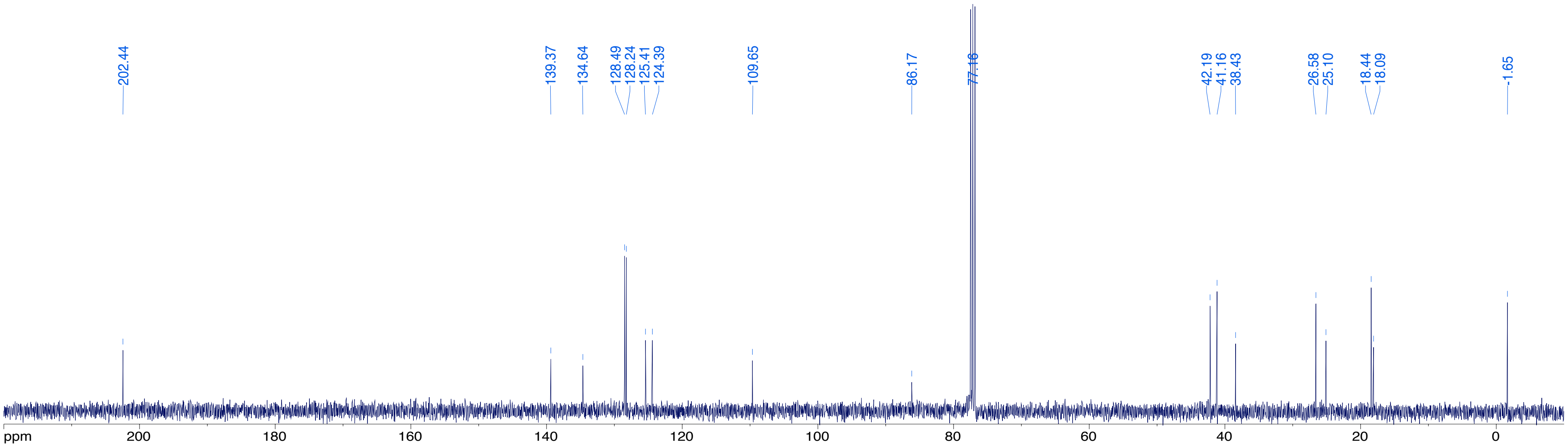

Benzyl((3*R*,4*R*,*E*)-3-(4,4-dibromobut-3-en-1-yl)-4-methylhept-5-en-1-yn-1-yl)dimethylsilane, S7

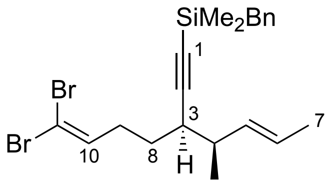

S7

^{1}H NMR (400 MHz, $CDCl_3$)
^{13}C NMR (100 MHz, $CDCl_3$)

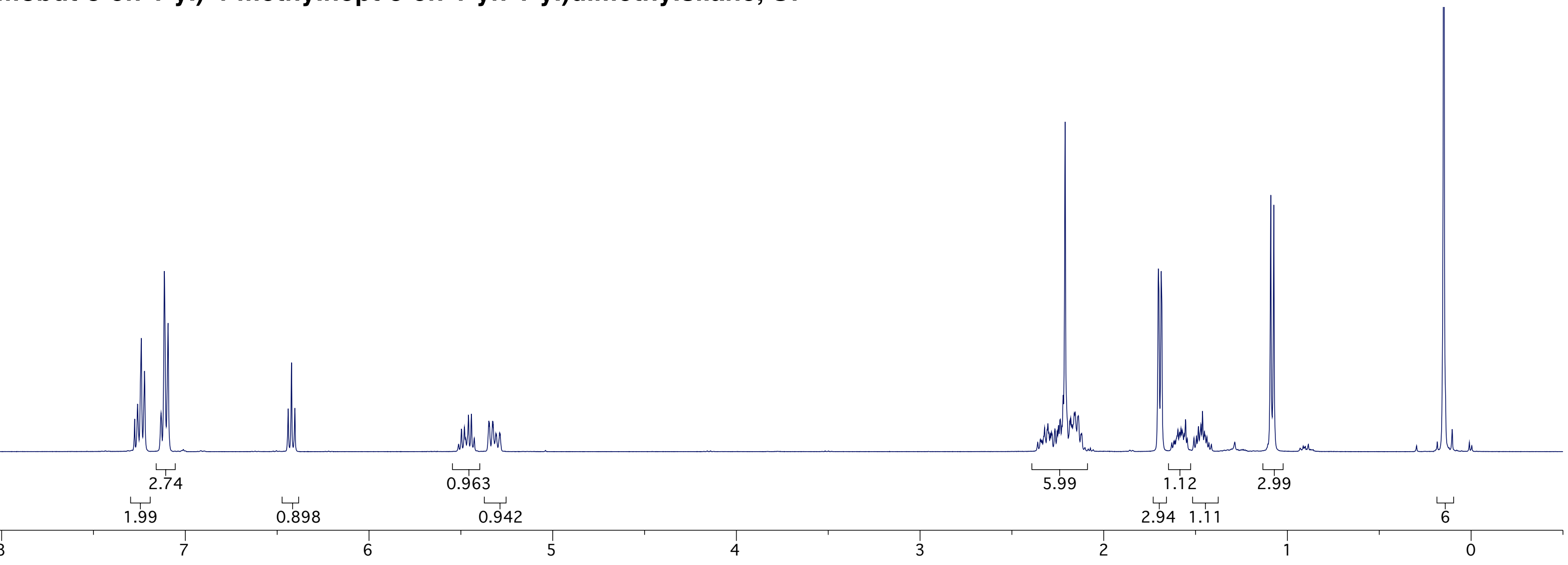

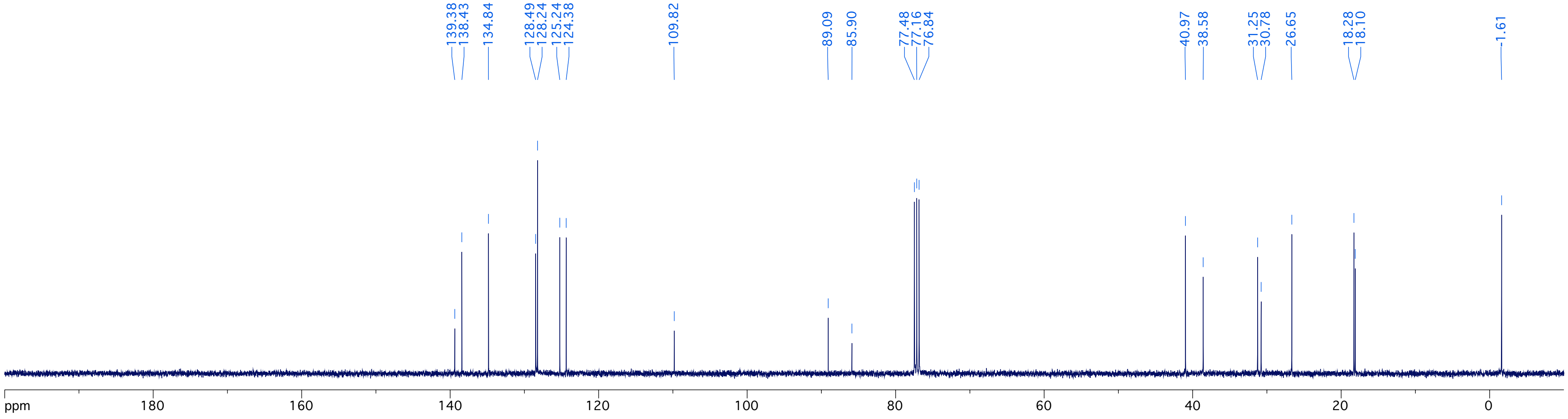

Benzyl((*3R*,*4R*,*E*)-3-(but-3-ynyl)-4-methylhept-5-en-1-ynyl)dimethylsilane, 7

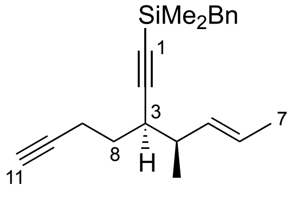

7

^{1}H NMR (400 MHz, $CDCl_3$)

^{13}C NMR (100 MHz, $CDCl_3$)

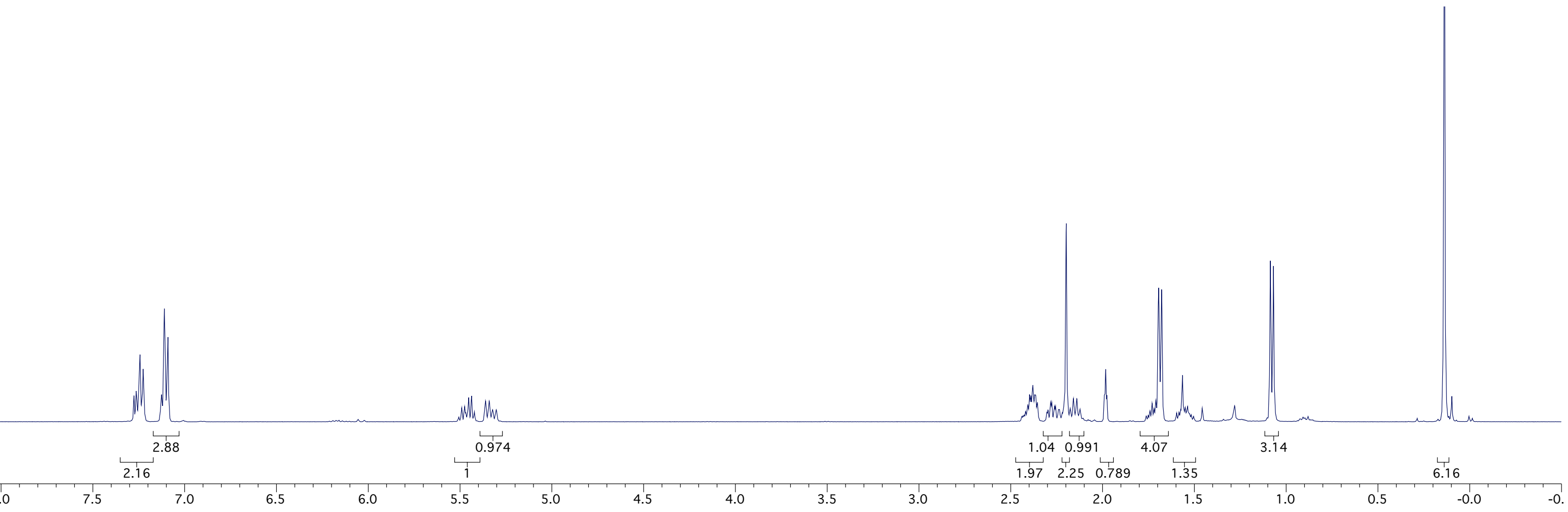

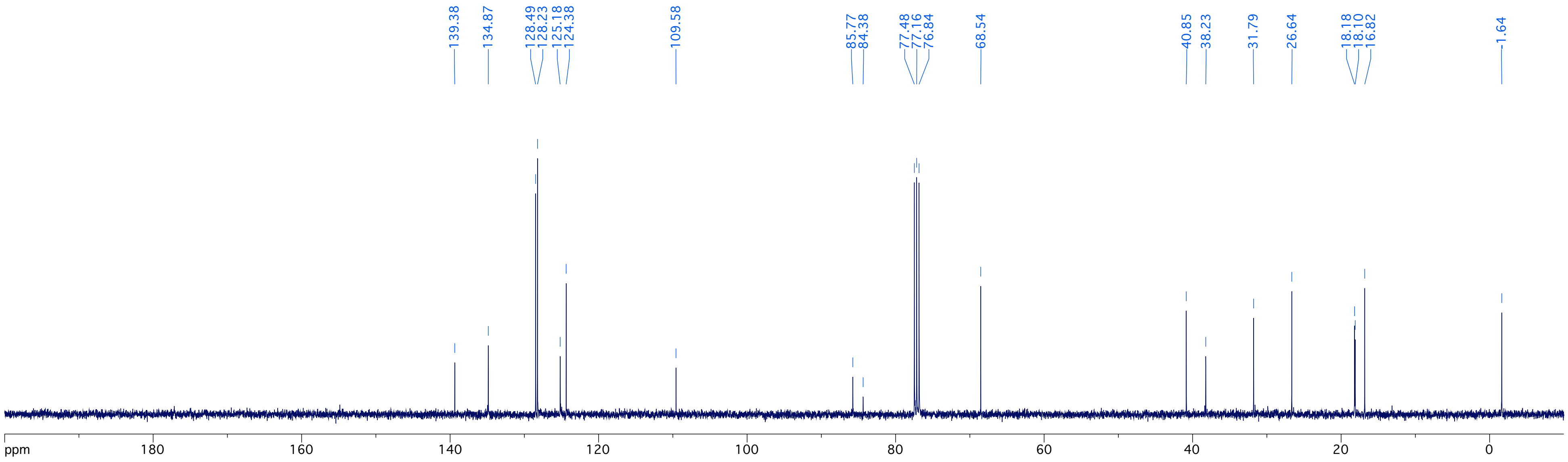

3.2 *Intermediates in the synthesis of AB ring alkyne **8***

Ethyl 4-((*tert*-butyldimethylsilyl)oxy)but-2-ynoate, 13

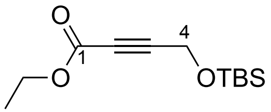

13

^{1}H NMR (400 MHz, $CDCl_3$)

^{13}C NMR (100 MHz, $CDCl_3$)

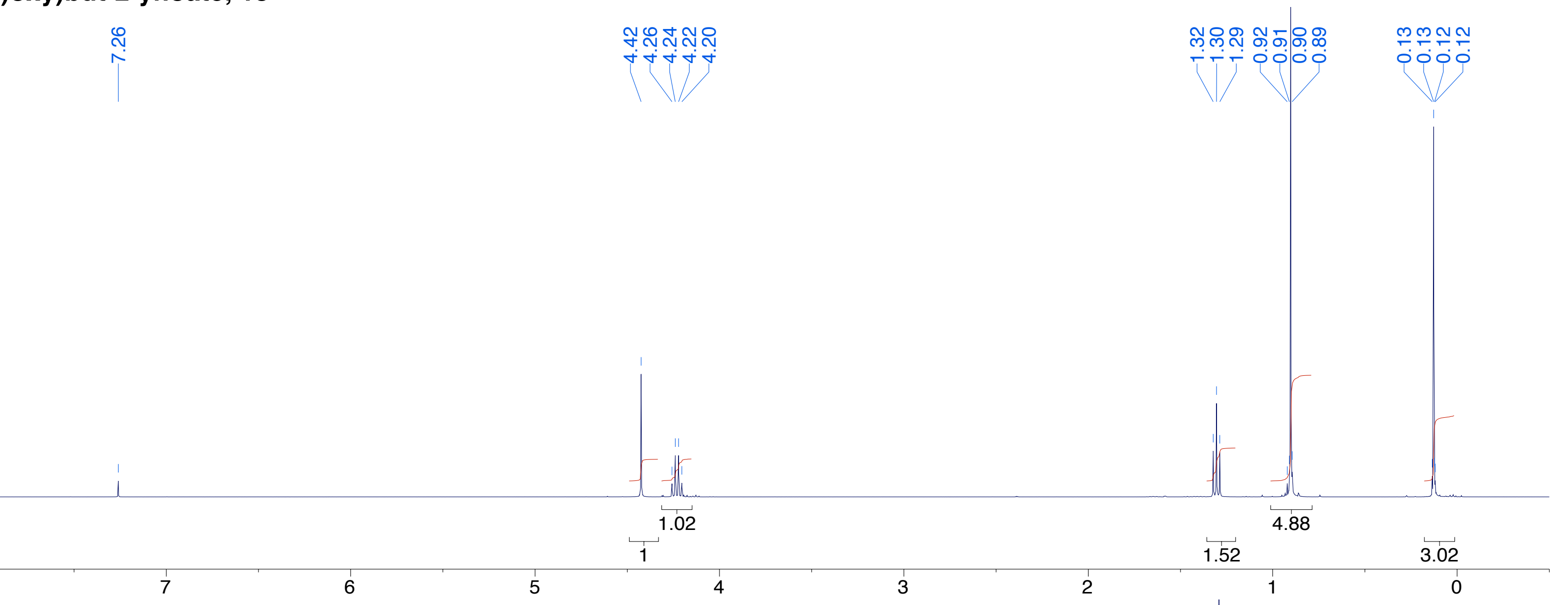

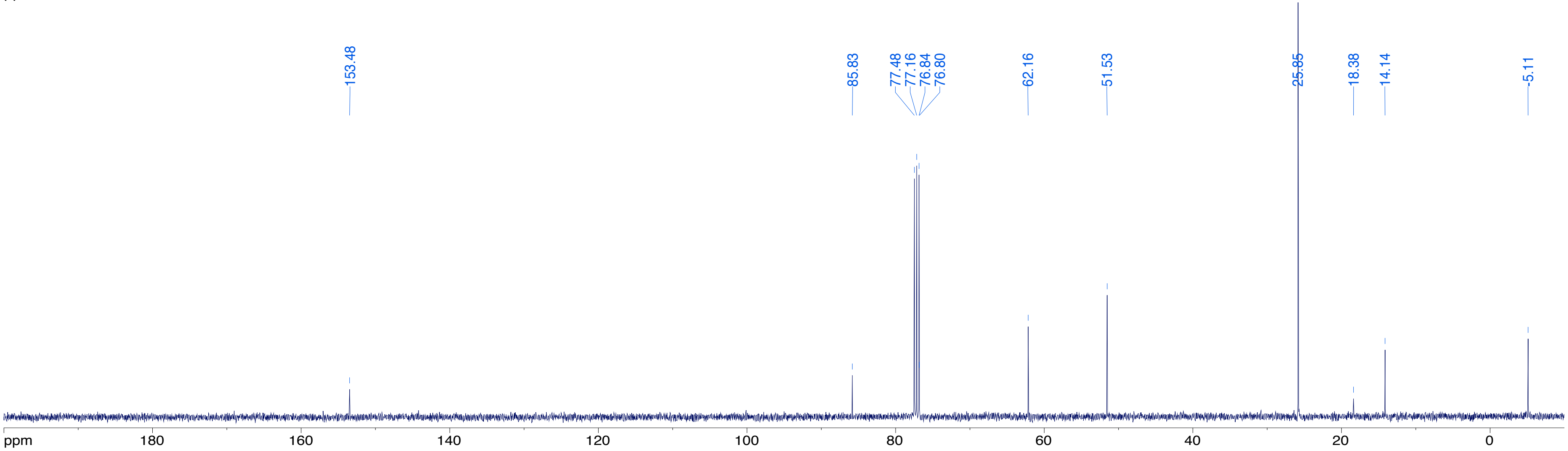

Ethyl (*Z*)-3-(((*tert*-butyldimethylsilyl)oxy)methyl)-6-(trimethylsilyl)hex-2-en-5-ynoate, S8

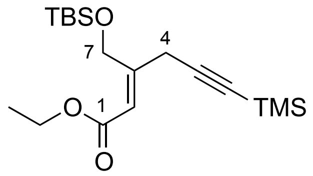

S8

^{1}H NMR (500 MHz, $CDCl_3$)

^{13}C NMR (125 MHz, $CDCl_3$)

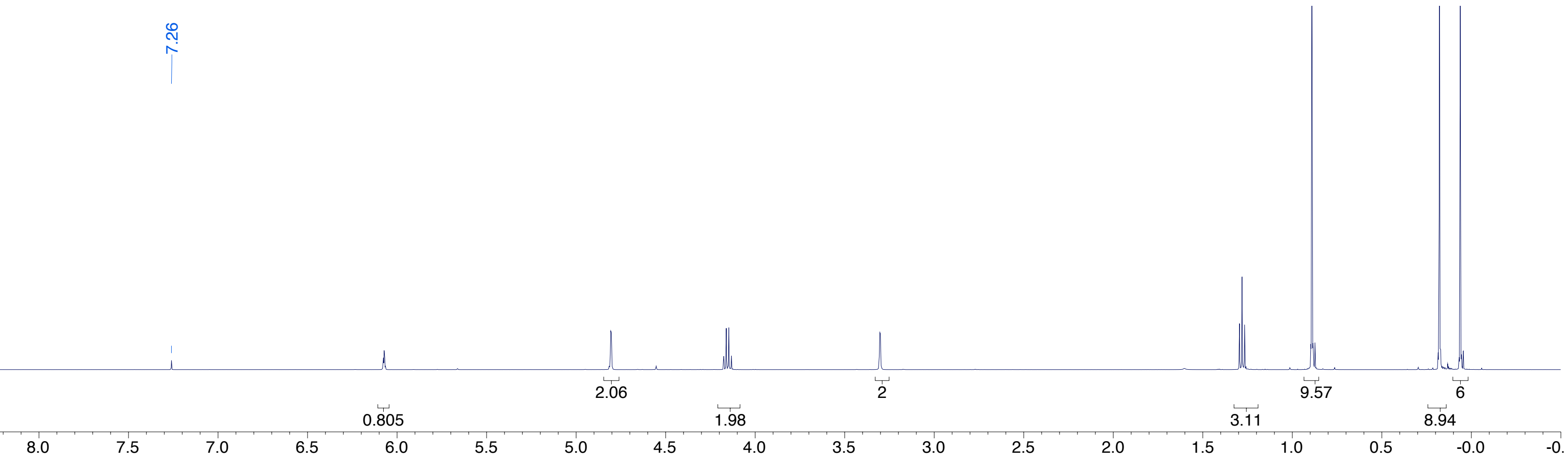

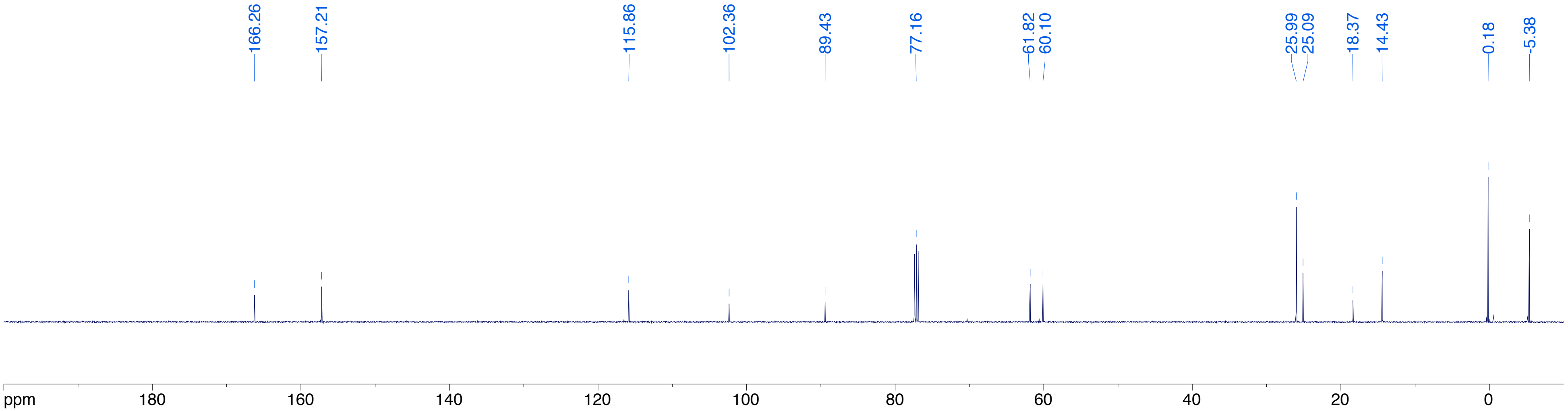

(*Z*)-3-(((*tert*-Butyldimethylsilyl)oxy)methyl)-6-(trimethylsilyl)hex-2-en-5-yn-1-ol, S9

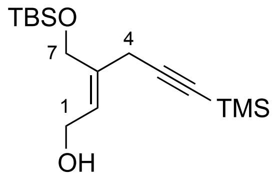

S9

^{1}H NMR (500 MHz, $CDCl_3$)

^{13}C NMR (125 MHz, $CDCl_3$)

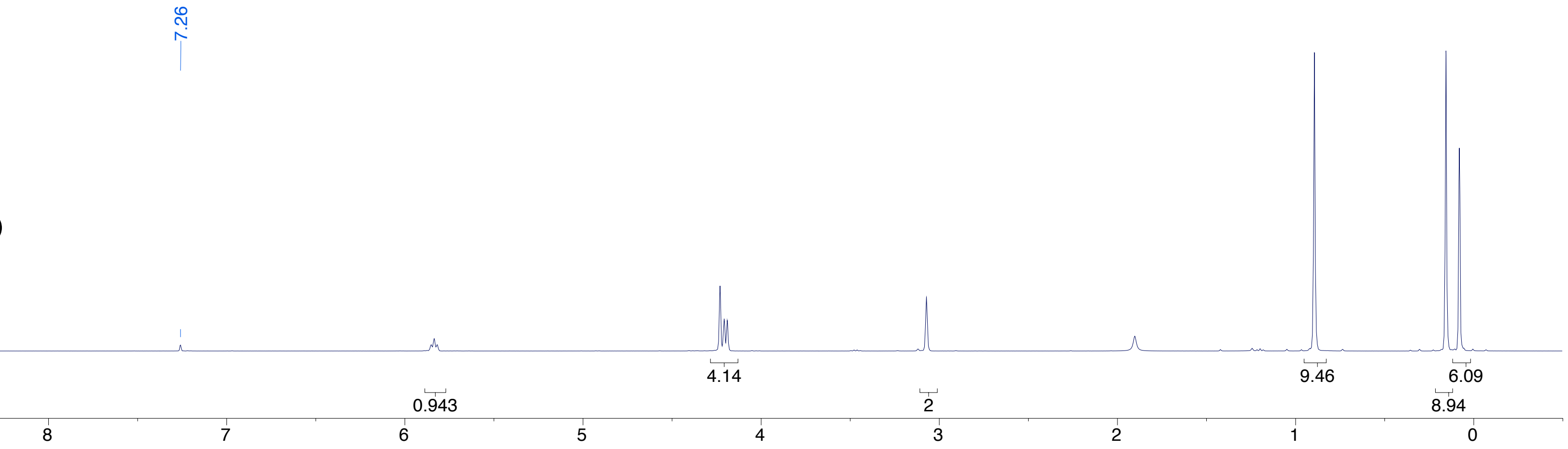

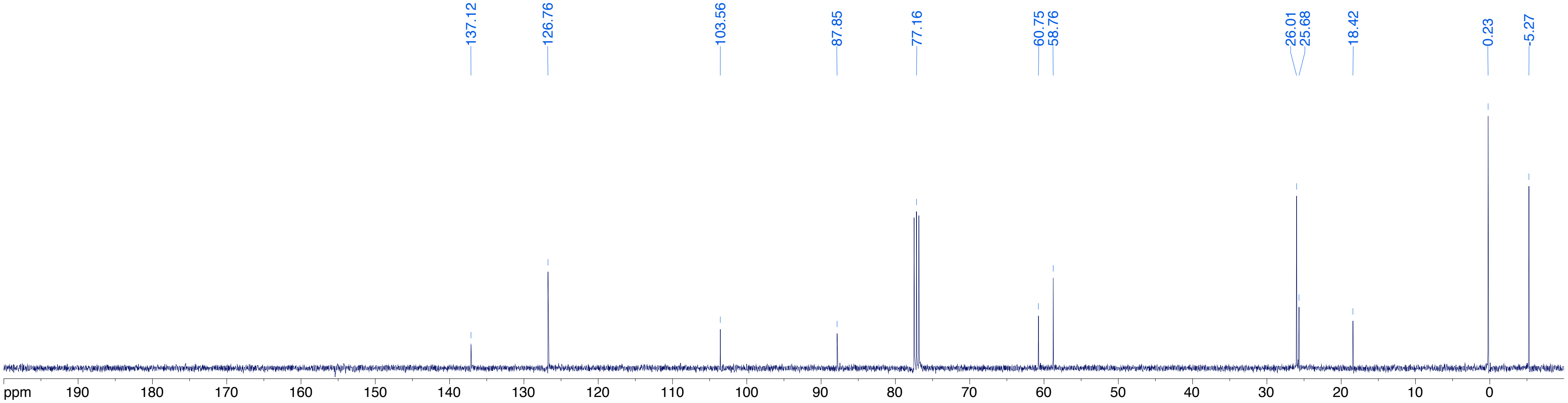

((2*R*,3*S*)-3-(((*tert*-Butyldimethylsilyl)oxy)methyl)-3-(3-(trimethylsilyl)prop-2-yn-1-yl)oxiran-2-yl)methanol, 14

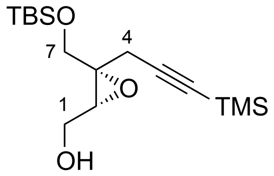

14

^{1}H NMR (500 MHz, $CDCl_3$)

^{13}C NMR (125 MHz, $CDCl_3$)

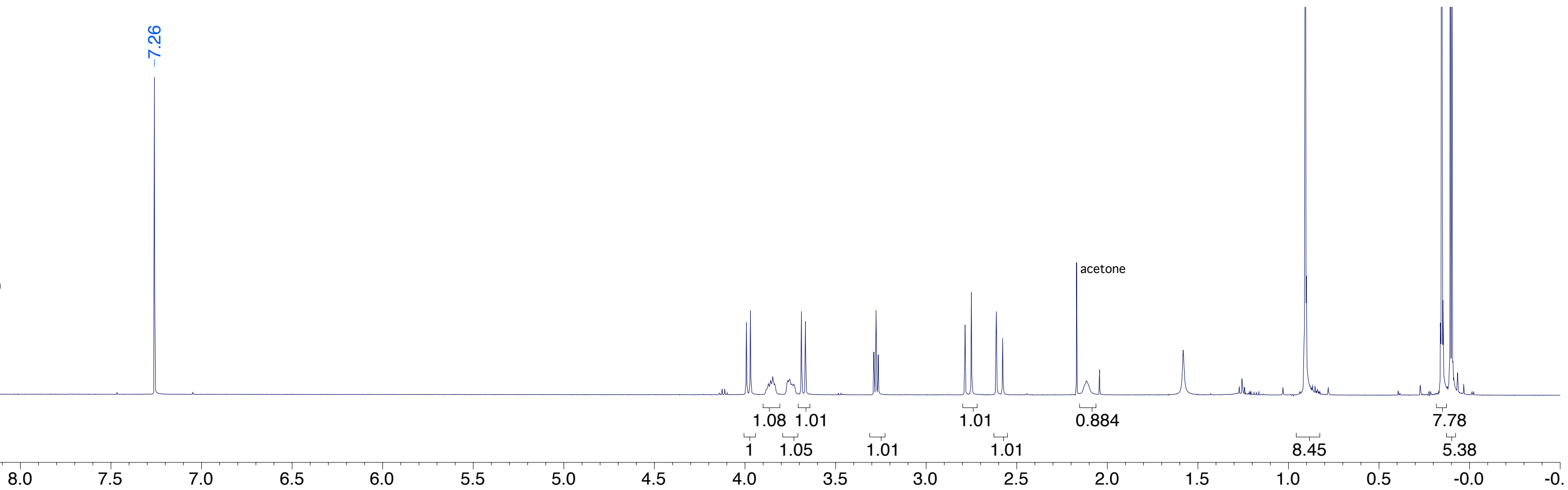

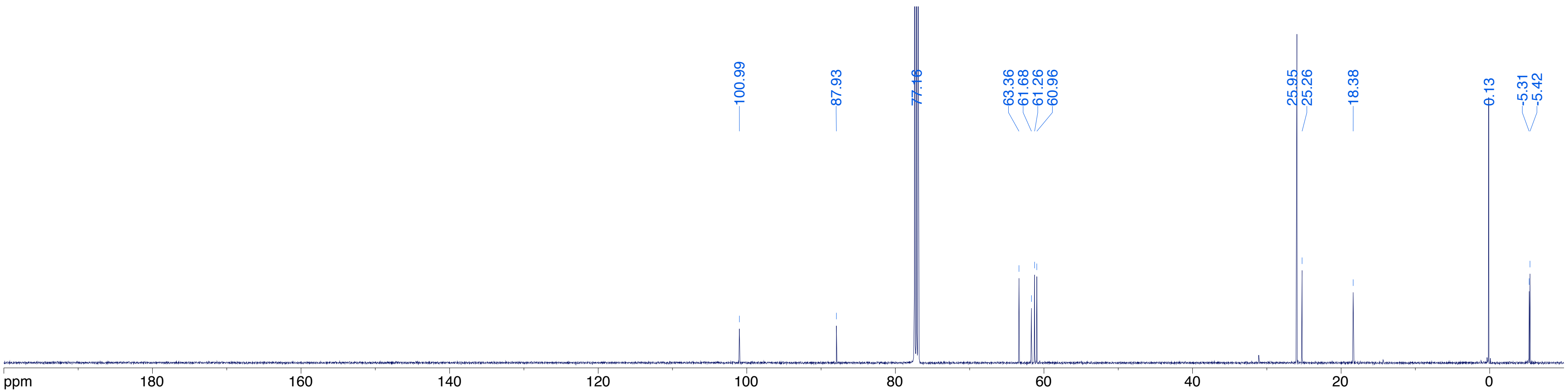

((2*R*,3*S*)-3-(((*tert*-butyldimethylsilyl)oxy)methyl)-3-(3-(trimethylsilyl)prop-2-yn-1-yl)oxiran-2-yl)methyl benzoate (prepared for purposes of determining the *ee* of **14**).

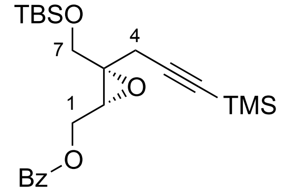

^{1}H NMR (400 MHz, $CDCl_3$)

^{13}C NMR (100 MHz, $CDCl_3$)

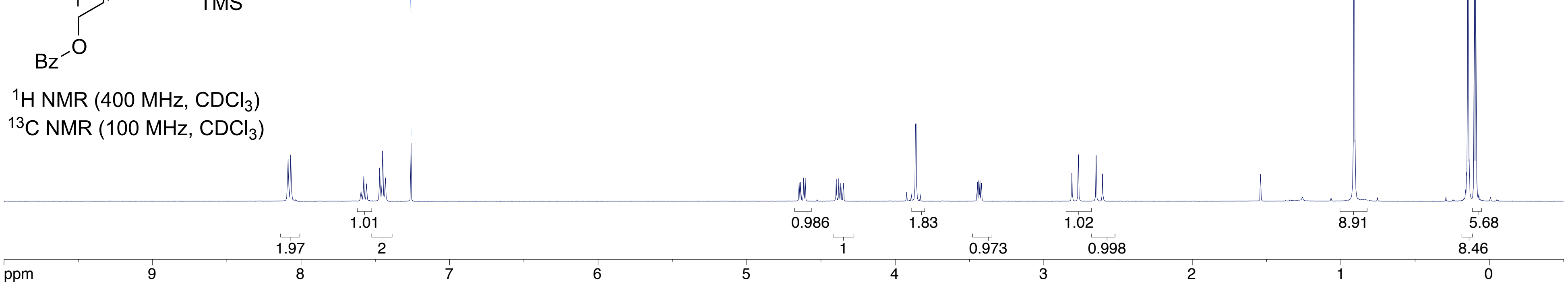

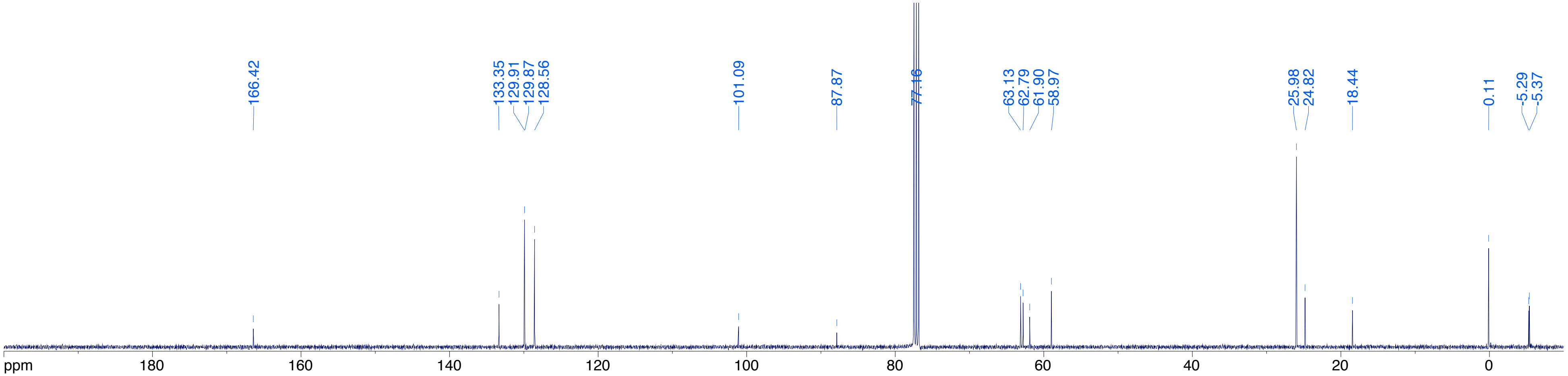

(2*S*,3*R*)-2-Allyl-3-(((*tert*-butyldimethylsilyl)oxy)methyl)-6-(trimethylsilyl)hex-5-yne-1,3-diol, S10

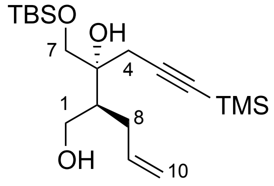

S10

^{1}H NMR (500 MHz, $CDCl_3$)

^{13}C NMR (125 MHz, $CDCl_3$)

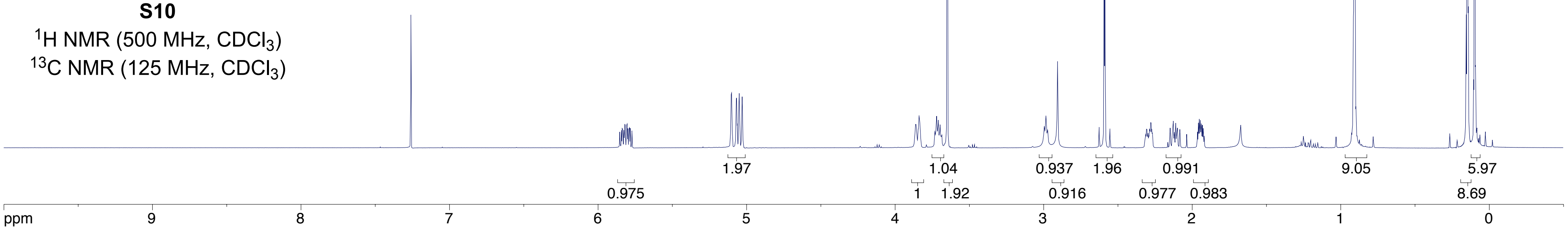

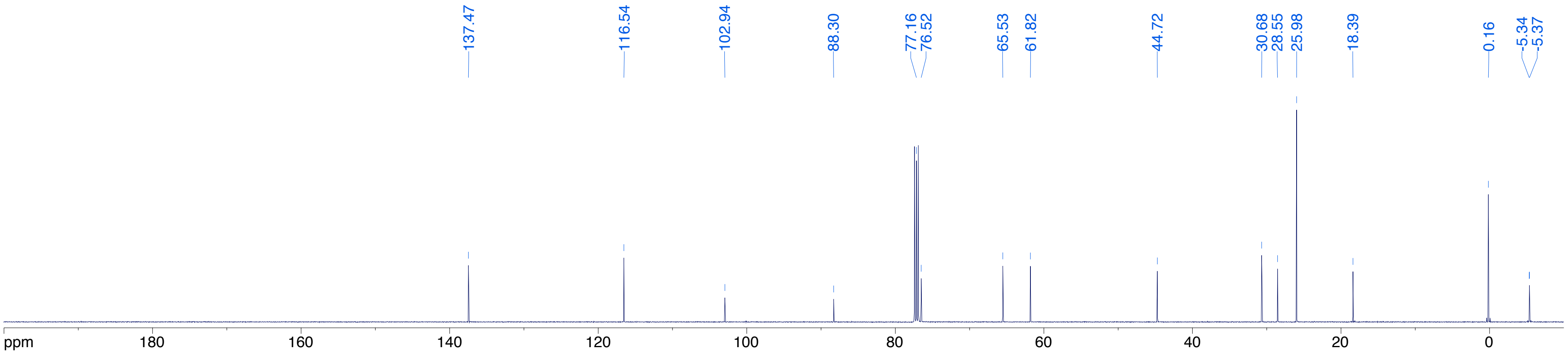

(2*R*,3*R*)-2-Allyl-3-(((*tert*-butyldimethylsilyl)oxy)methyl)-3-hydroxy-6-(trimethylsilyl)hex-5-ynoic acid, S11

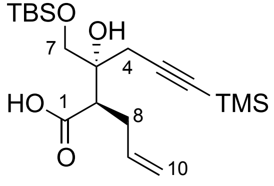

S11

^{1}H NMR (500 MHz, $CDCl_3$)

^{13}C NMR (125 MHz, $CDCl_3$)

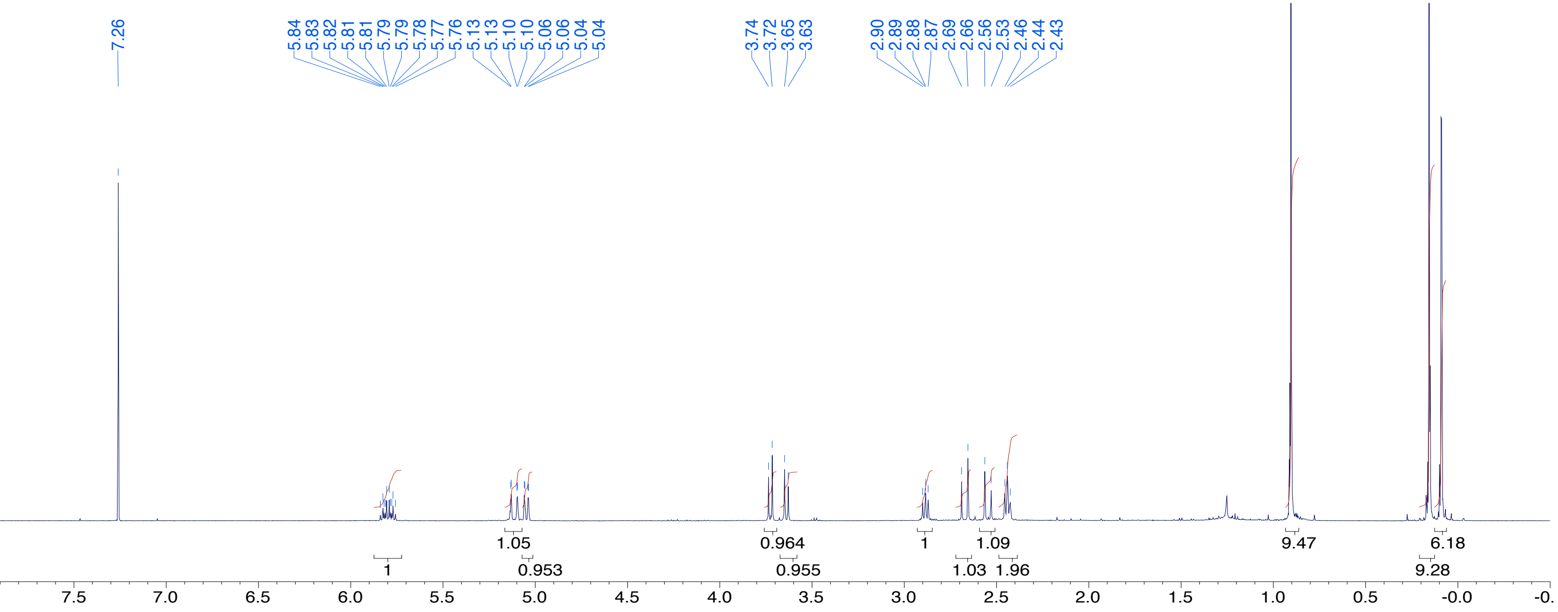

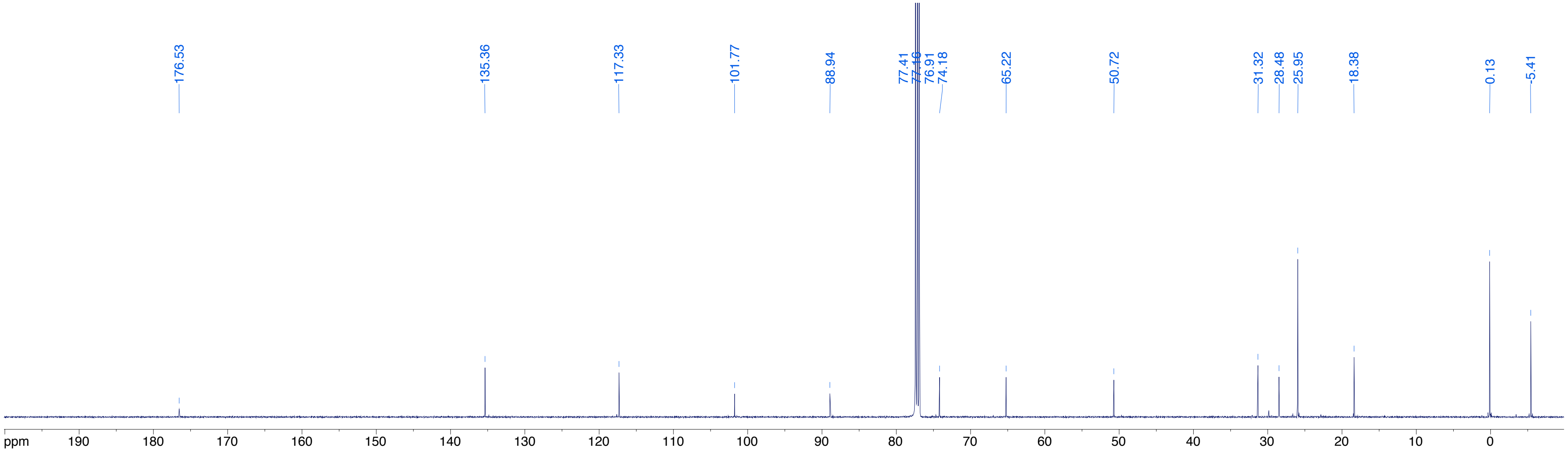

(3*R*,4*R*)-3-Allyl-4-(((*tert*-butyldimethylsilyl)oxy)methyl)-4-(3-(trimethylsilyl)prop-2-yn-1-yl)oxetan-2-one, 15

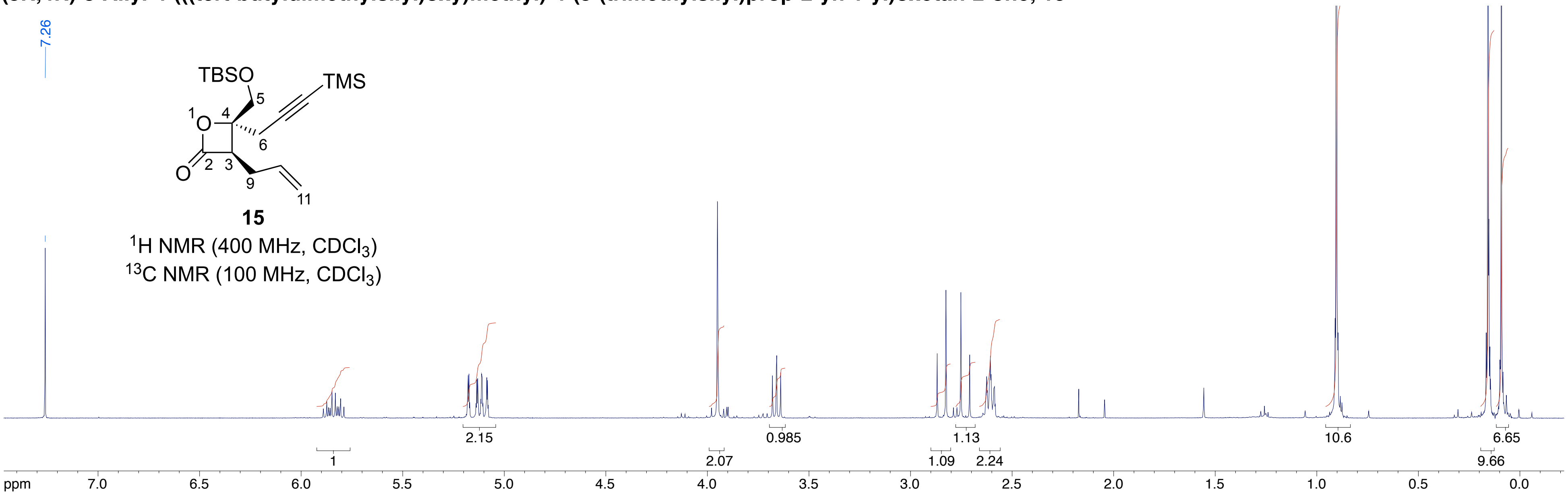

15

^{1}H NMR (400 MHz, $CDCl_3$)

^{13}C NMR (100 MHz, $CDCl_3$)

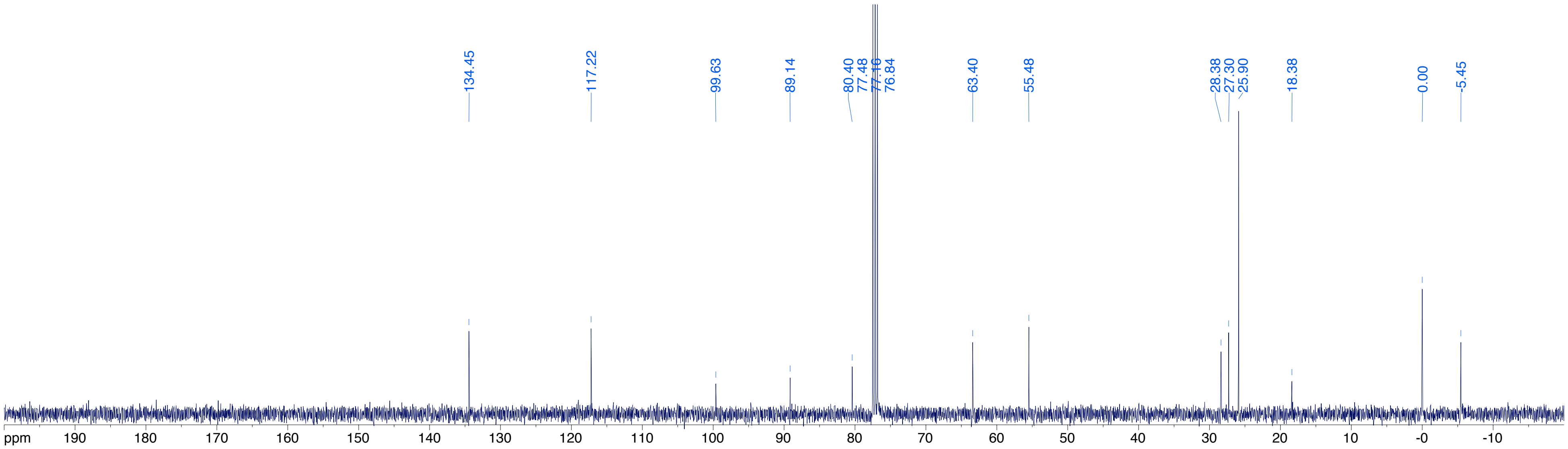

(3*S*,4*R*)-3-Allyl-4-(((*tert*-butyldimethylsilyl)oxy)methyl)-2-methyl-7-(trimethylsilyl)hept-6-yne-2,4-diol, 16

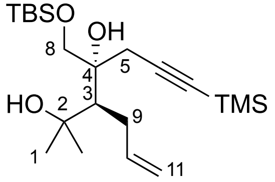

16

^{1}H NMR (400 MHz, $CDCl_3$)

^{13}C NMR (125 MHz, $CDCl_3$)

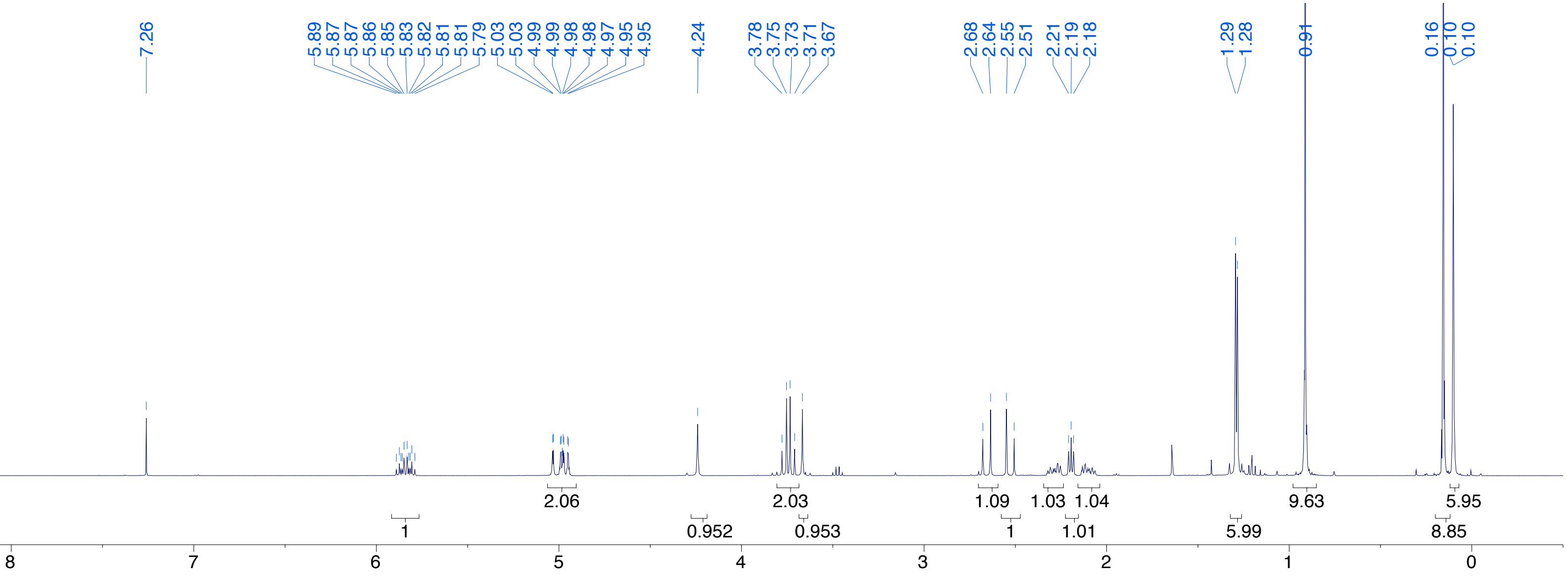

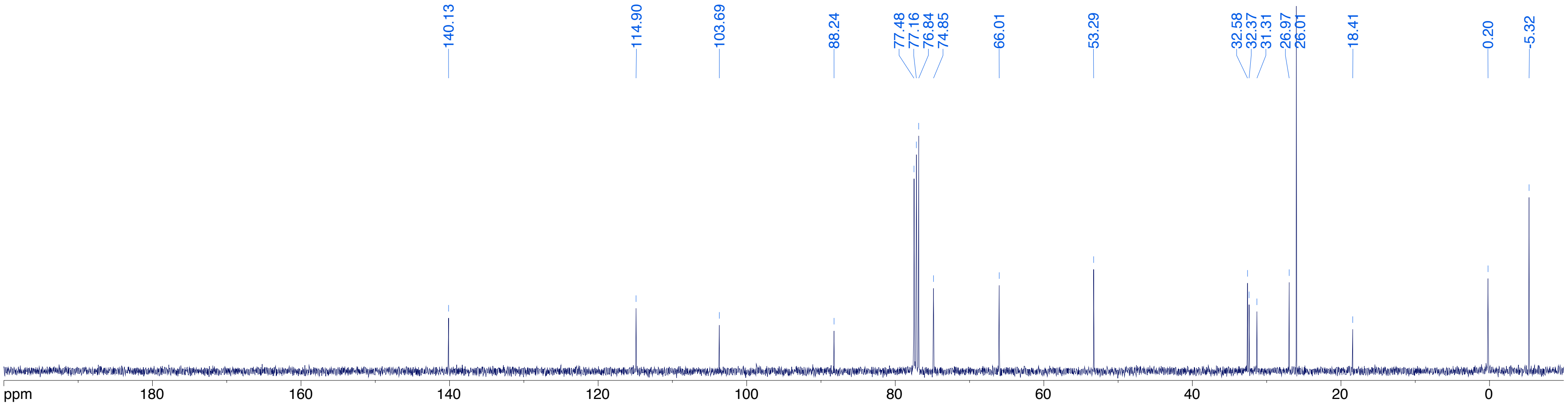

(3*R*,4*R*)-3-allyl-4-(((*tert*-butyldimethylsilyl)oxy)methyl)-4-hydroxy-7-(trimethylsilyl)hept-6-yn-2-one, S12

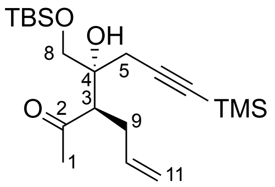

S12

^{1}H NMR (400 MHz, $CDCl_3$)

^{13}C NMR (101 MHz, $CDCl_3$)

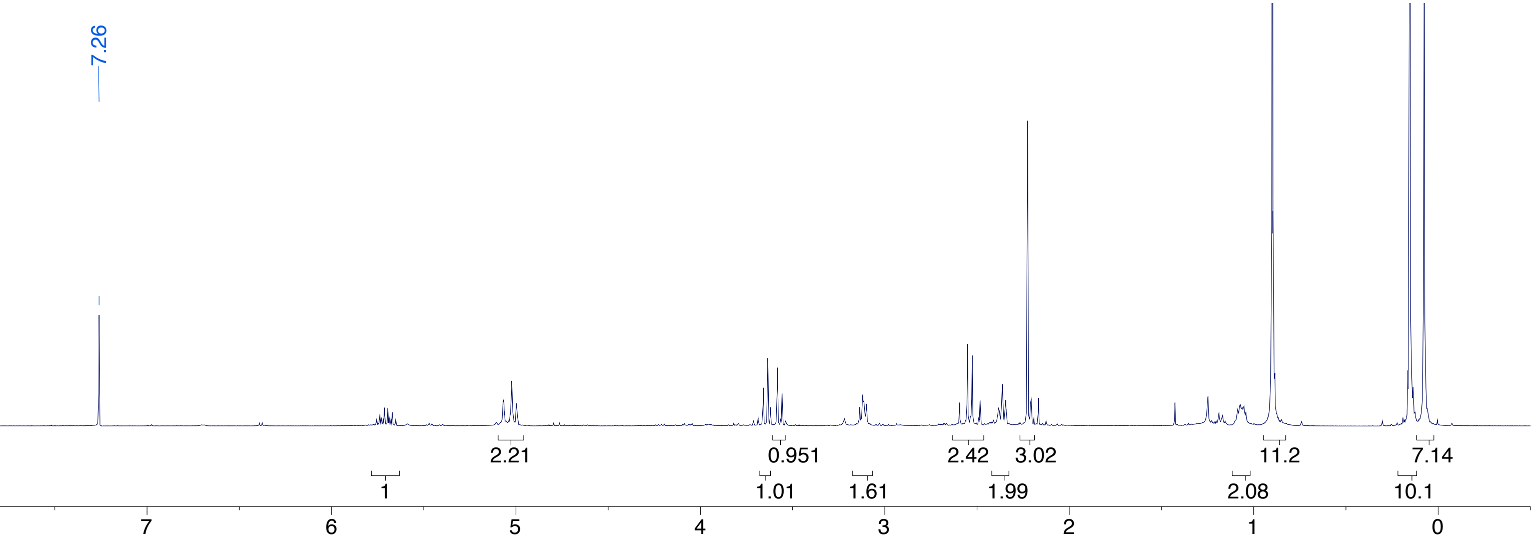

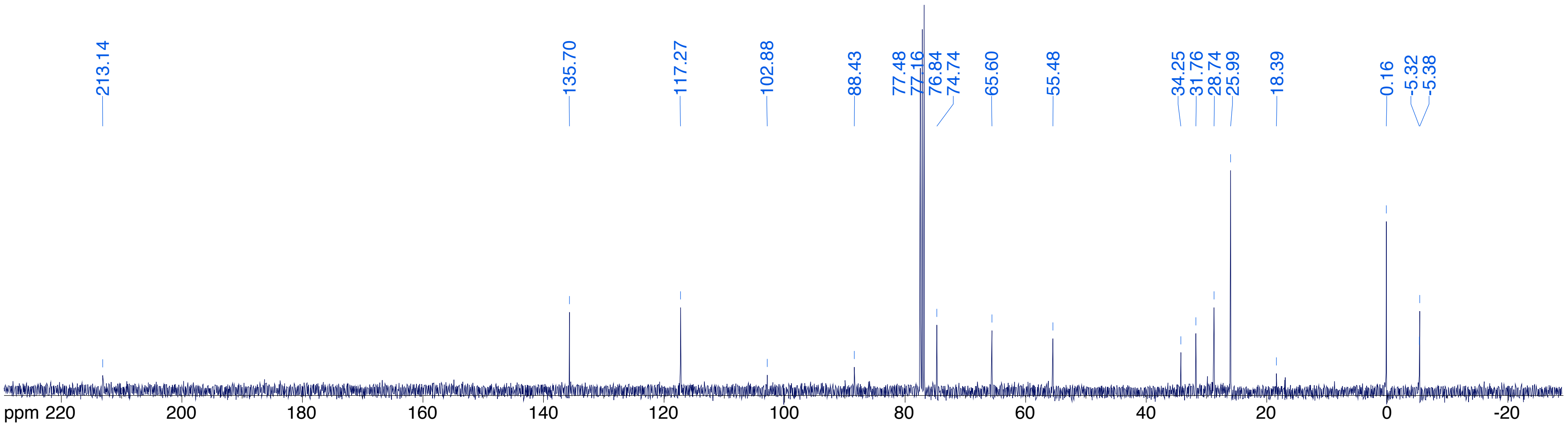

(4*R*)-4-((*R*)-1-((*tert*-Butyldimethylsilyl)oxy)-2-hydroxy-5-(trimethylsilyl)pent-4-yn-2-yl)-5,5-dimethyltetrahydrofuran-2-ol, S13 and (4*S*,5*R*)-5-(((*tert*-butyldimethylsilyl)oxy)methyl)-4-(2-hydroxypropan-2-yl)-5-(3-(trimethylsilyl)prop-2-yn-1-yl)tetrahydrofuran-2-ol, S14

These compounds were isolated as a 40:27:22:11 inseparable mixture of regio and stereoisomers

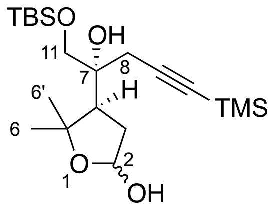

S13

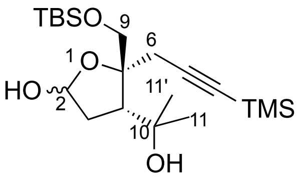

S14

^{1}H NMR (500 MHz, $CDCl_3$)

^{13}C NMR (125 MHz, $CDCl_3$)

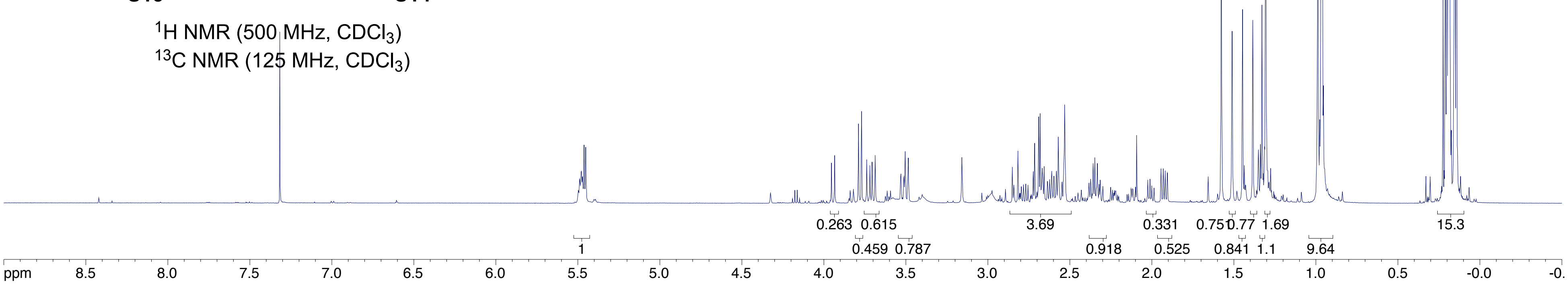

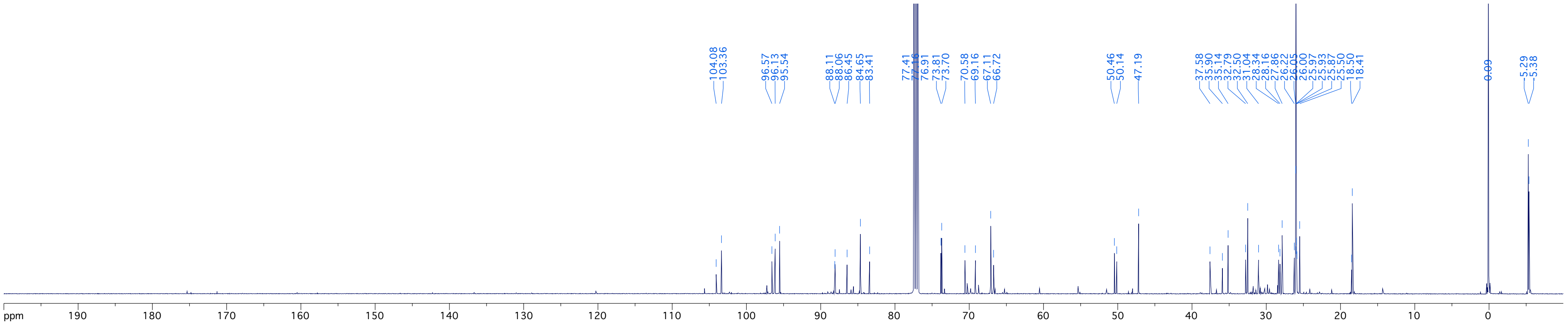

(2*R*)-2-((3*R*)-5-methoxy-2,2-dimethyltetrahydrofuran-3-yl)-5-(trimethylsilyl)pent-4-yne-1,2-diol S15

These compounds were typically isolated as mixtures of regio and stereoisomers, and are readily separable only when oxidized to aldehydes (see below)

*Major epimer of **S15**, contains small amount of the minor epimer:*

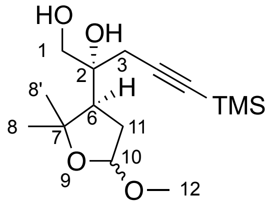

S15

^{1}H NMR (500 MHz, $CDCl_3$)

^{13}C NMR (125 MHz, $CDCl_3$)

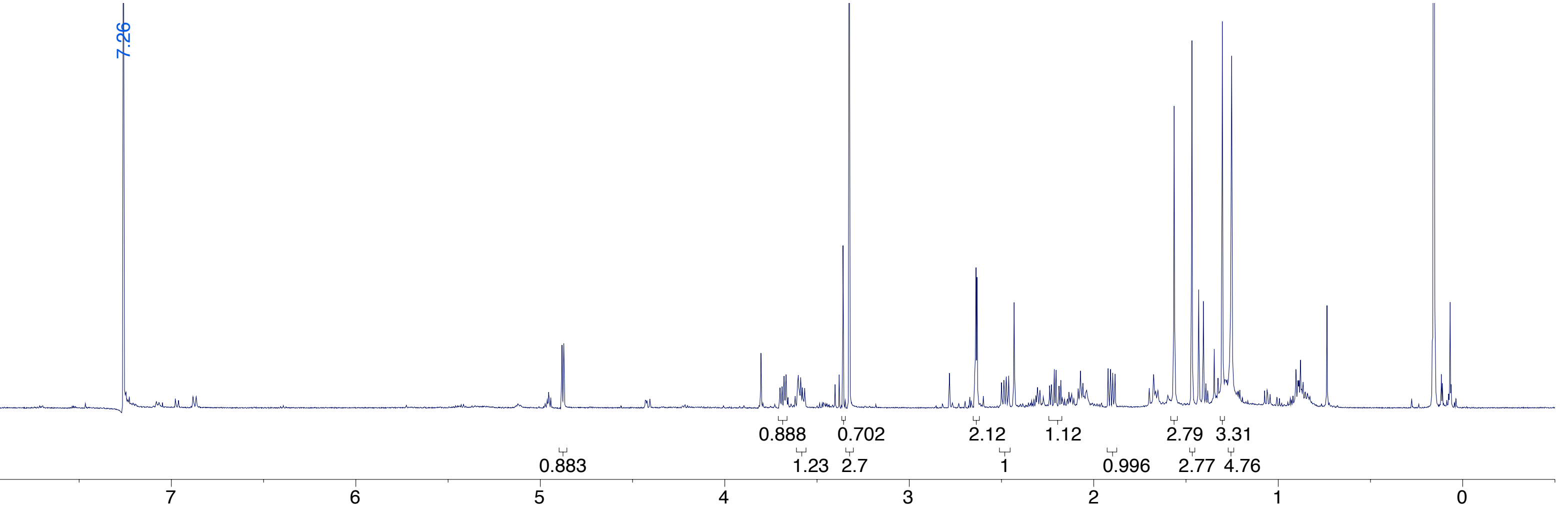

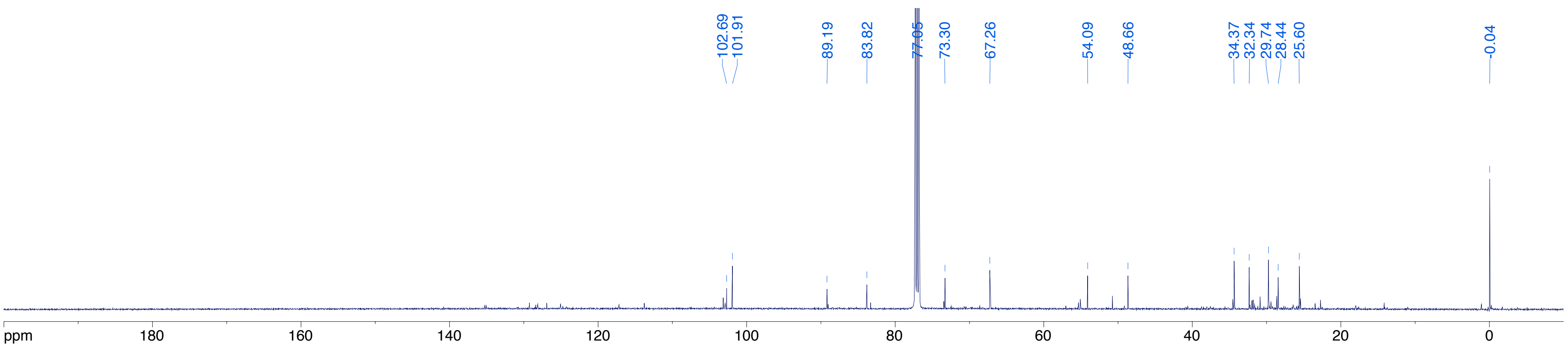

*Spectra of minor epimer of **S15**, in a 3:1 ratio with the major epimer:*

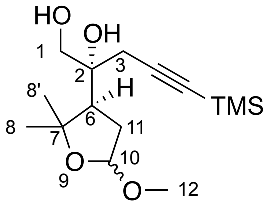

S15

^{1}H NMR (500 MHz, $CDCl_3$)

^{13}C NMR (125 MHz, $CDCl_3$)

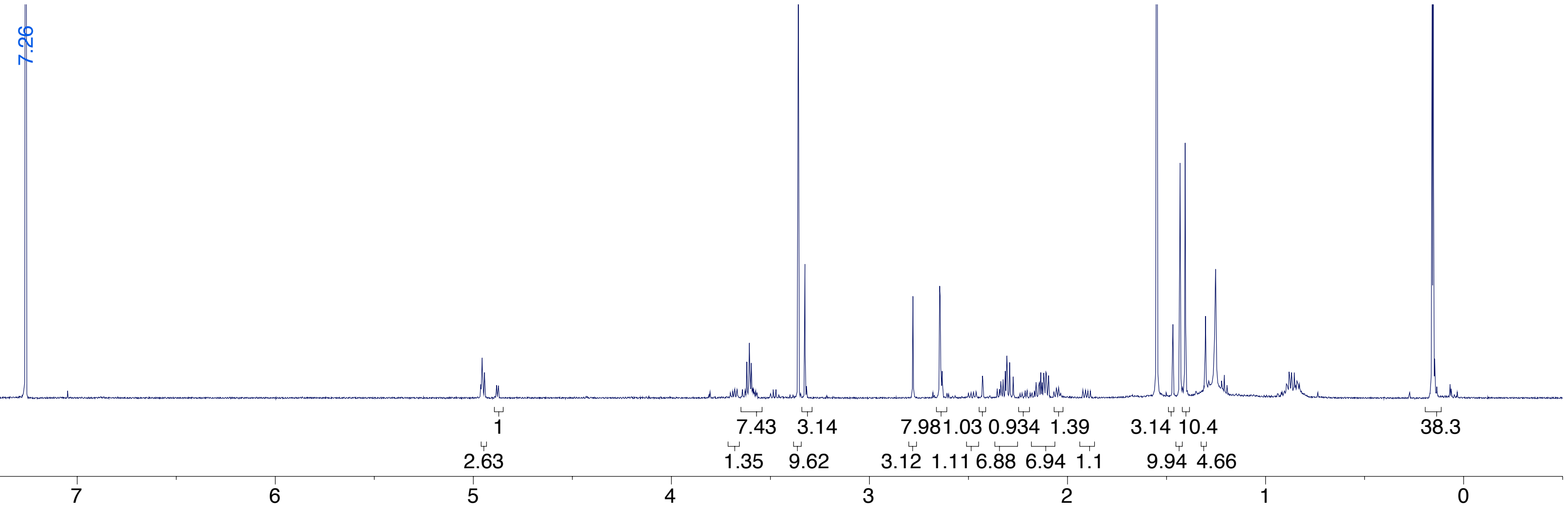

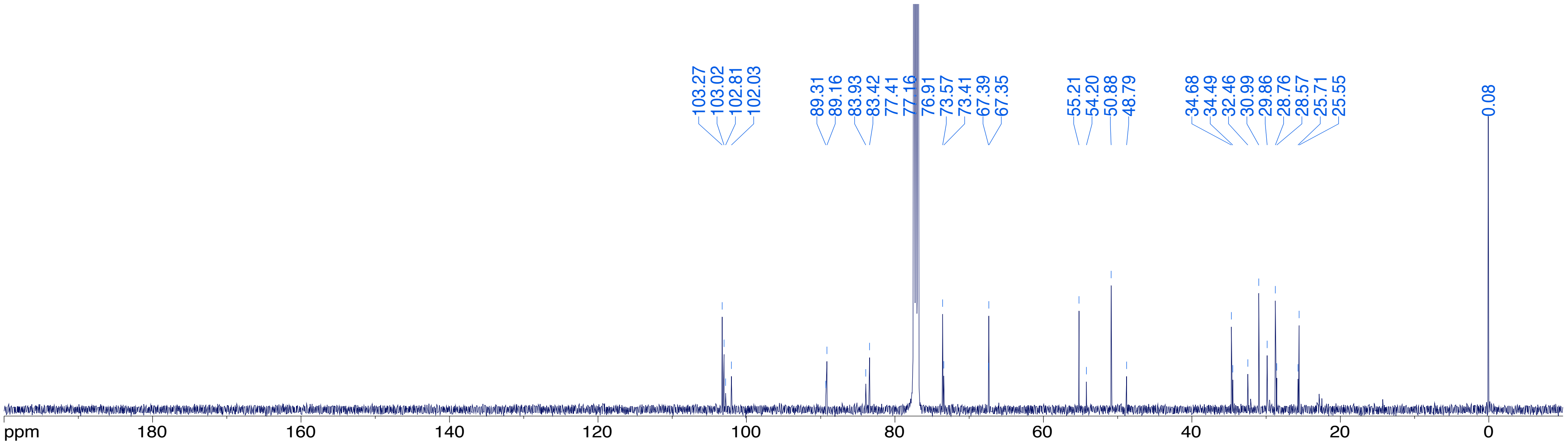

2-((2*R*,3*S*)-2-(hydroxymethyl)-5-methoxy-2-(3-(trimethylsilyl)prop-2-yn-1-yl)tetrahydrofuran-3-yl)propan-2-ol, S16

This spectrum is a ca. 1:1 mixture of the two C6 epimers

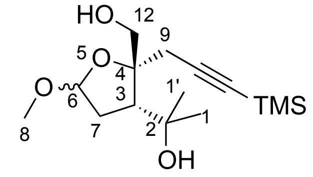

S16

^{1}H NMR (500 MHz, $CDCl_3$)

^{13}C NMR (125 MHz, $CDCl_3$)

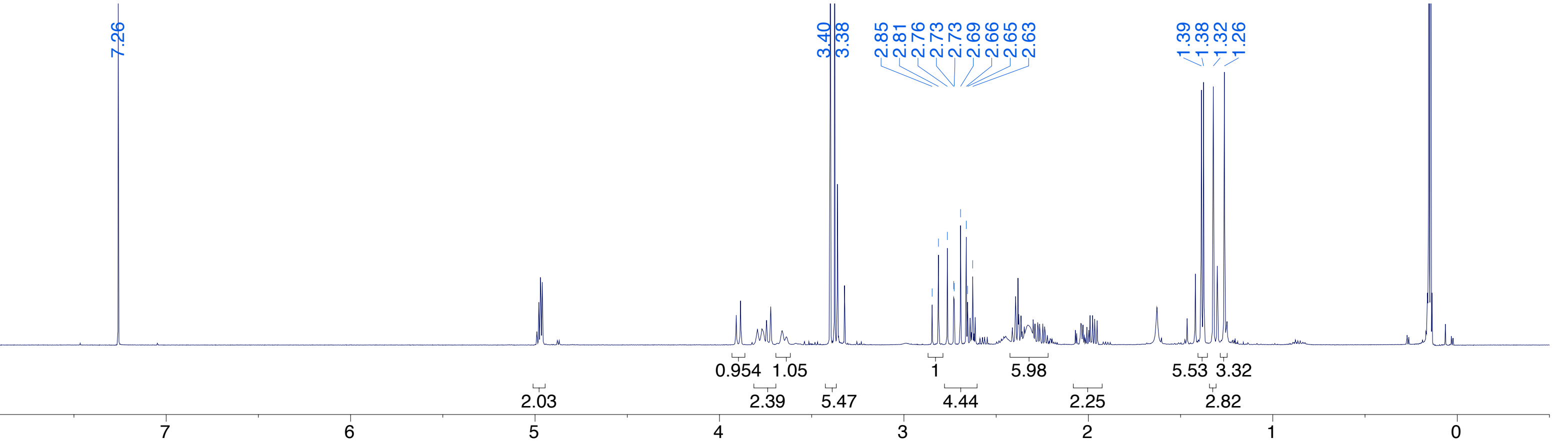

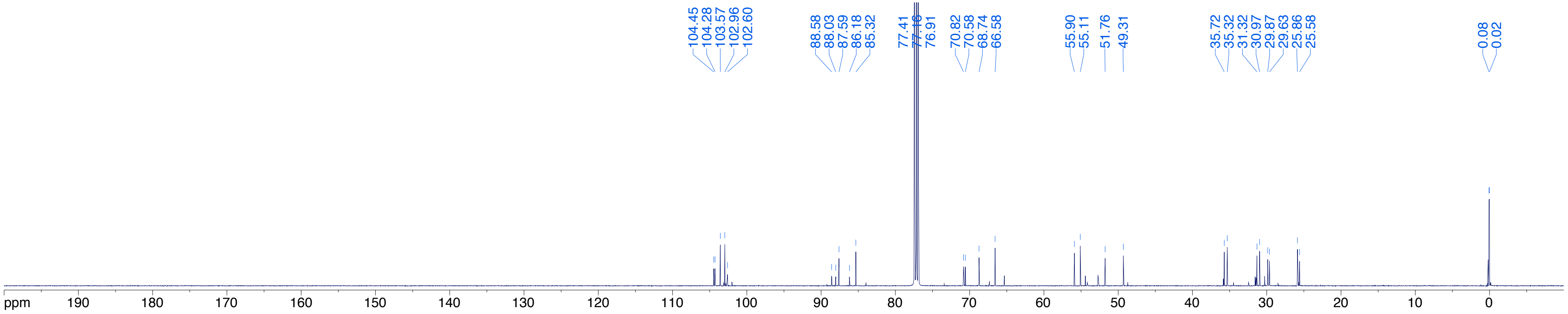

(2*R*)-2-hydroxy-2-((3*R*)-5-methoxy-2,2-dimethyltetrahydrofuran-3-yl)-5-(trimethylsilyl)pent-4-ynal, 17

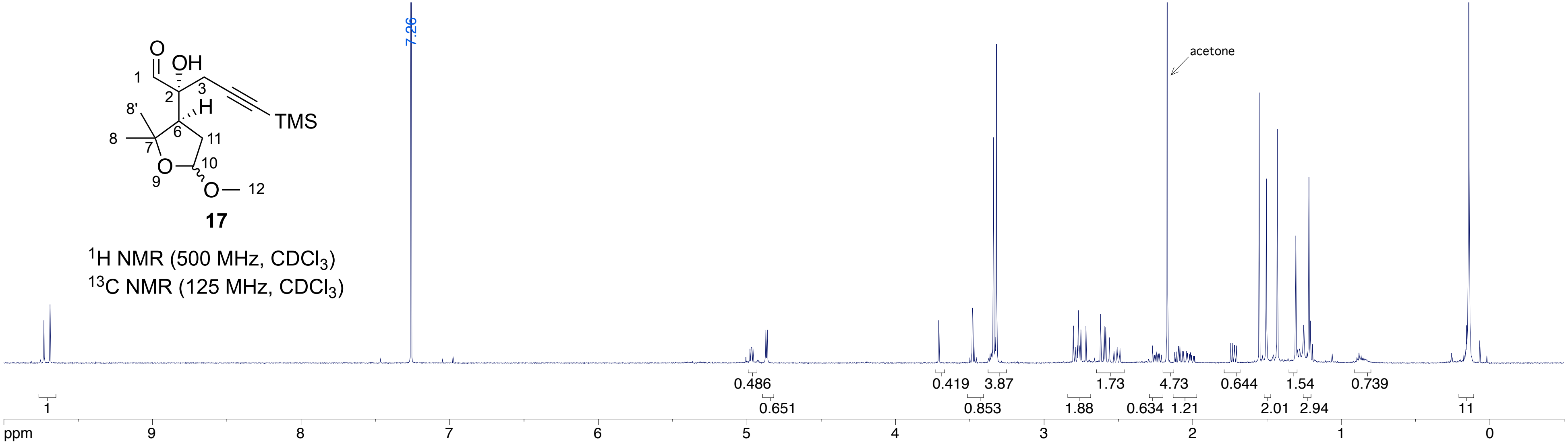

17

^{1}H NMR (500 MHz, $CDCl_3$)

^{13}C NMR (125 MHz, $CDCl_3$)

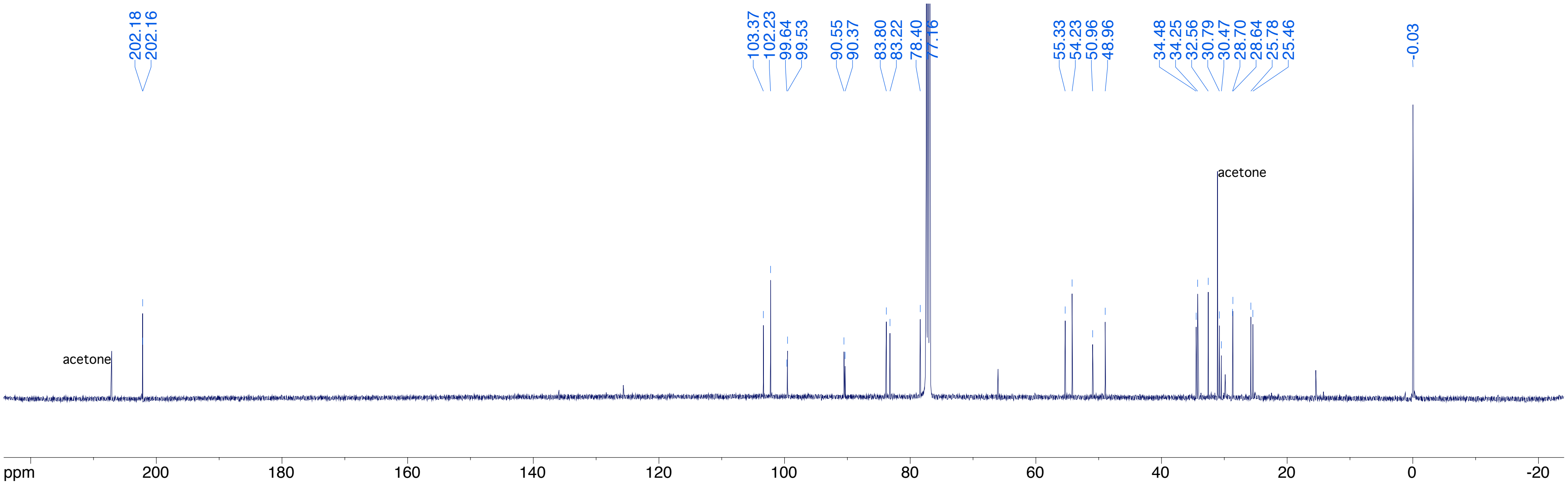

(2*R*,3*S*)-3-(2-hydroxypropan-2-yl)-5-methoxy-2-(3-(trimethylsilyl)prop-2-yn-1-yl)tetrahydrofuran-2-carbaldehyde 18

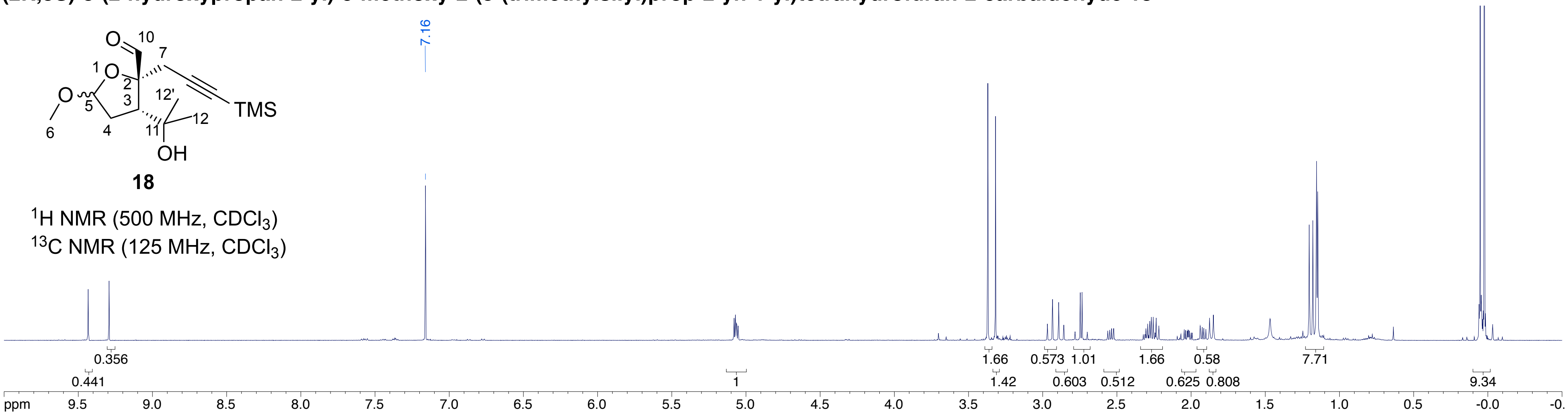

18

^{1}H NMR (500 MHz, $CDCl_3$)

^{13}C NMR (125 MHz, $CDCl_3$)

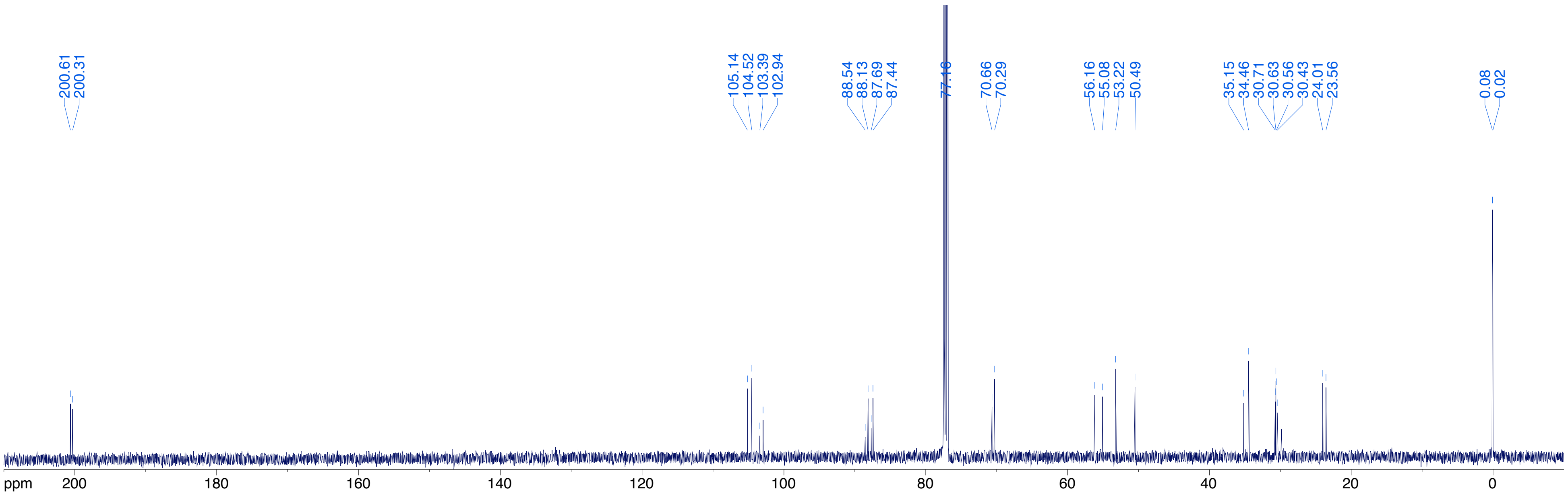

(5*S*)-5-((3*S*)-5-hydroxy-2,2-dimethyltetrahydrofuran-3-yl)-5-(3-(trimethylsilyl)prop-2-yn-1-yl)furan-2(5*H*)-one, 19

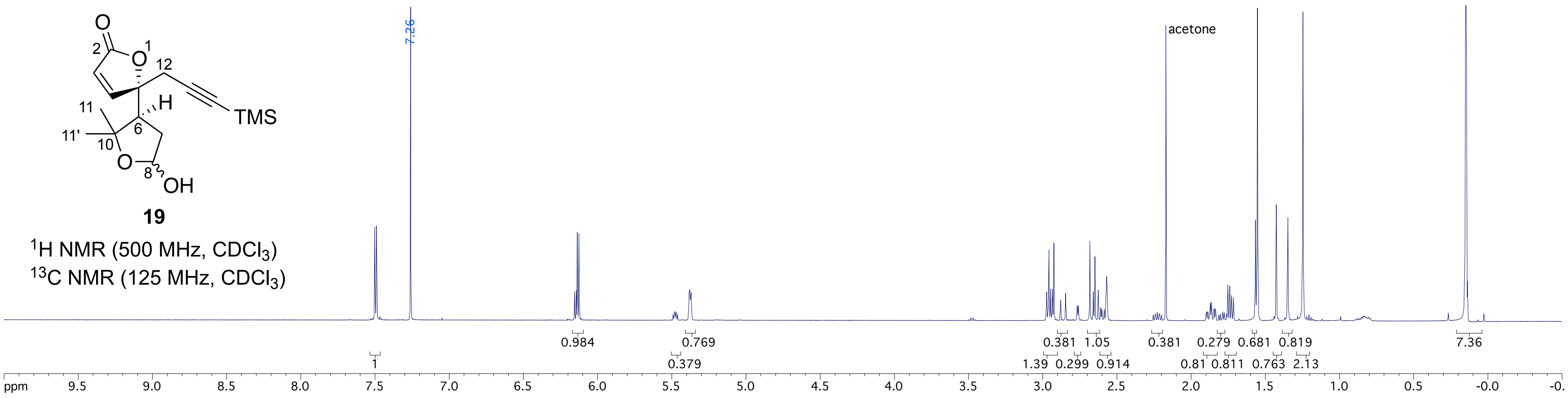

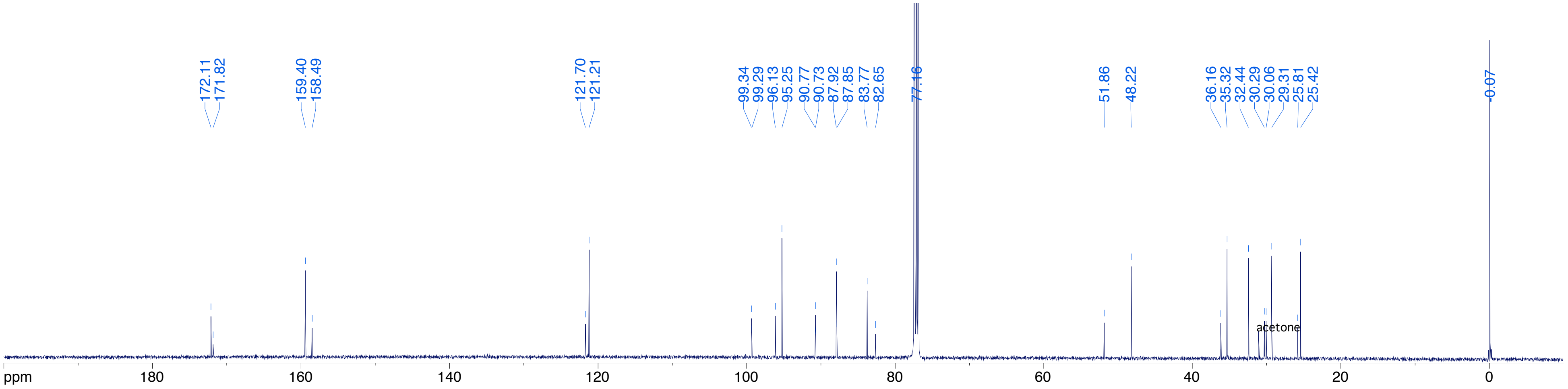

2-((3*S*,3a*R*,6a*R*)-2,2-dimethyl-5-oxo-3a-(prop-2-yn-1-yl)hexahydrofuro[3,2-*b*]furan-3-yl)acetaldehyde, 8

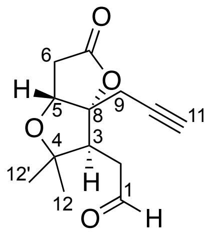

8

^{1}H NMR (500 MHz, $CDCl_3$)
^{13}C NMR (125 MHz, $CDCl_3$)

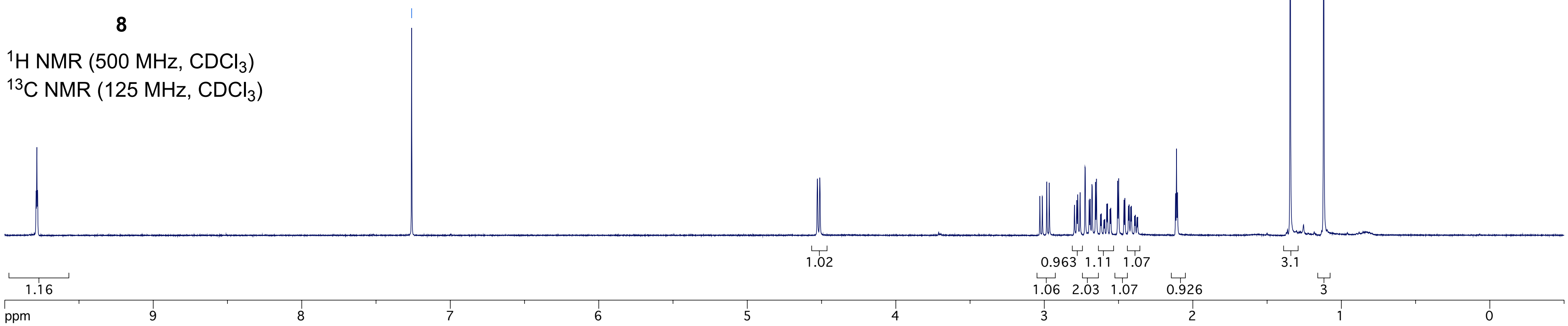

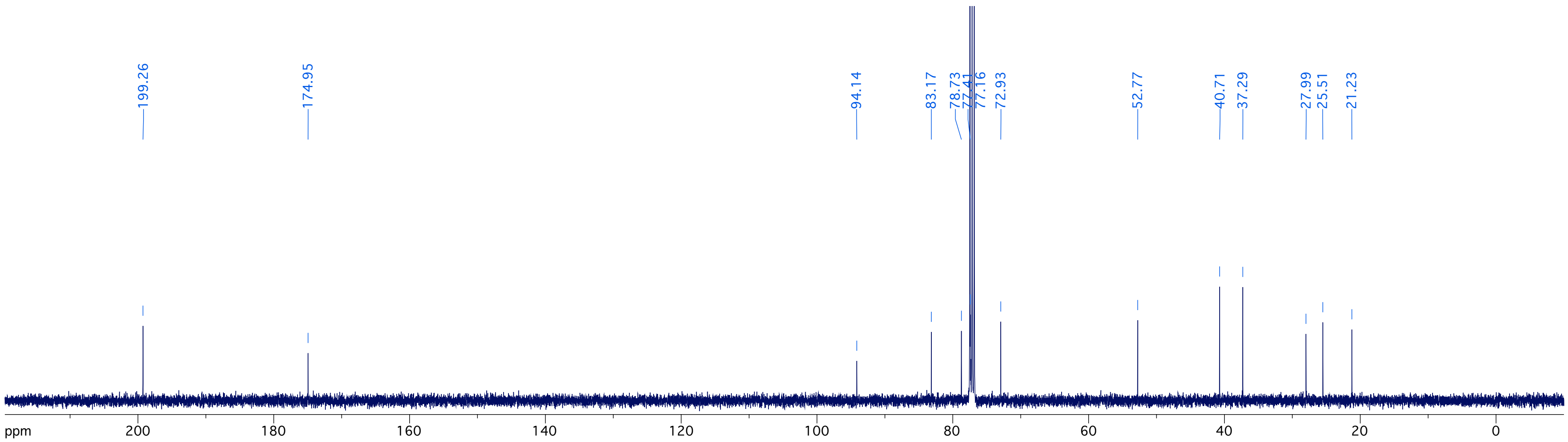

(3a*R*,6*S*,6a*R*)-6-((7*R*,8*R*,*E*)-7-((Benzyldimethylsilyl)ethynyl)-2-hydroxy-8-methylundeC9-en-3-yn-1-yl)-6a-(2-bromoallyl)-5,5-dimethyltetrahydrofuro[3,2-b]furan-2(3*H*)-one, 21

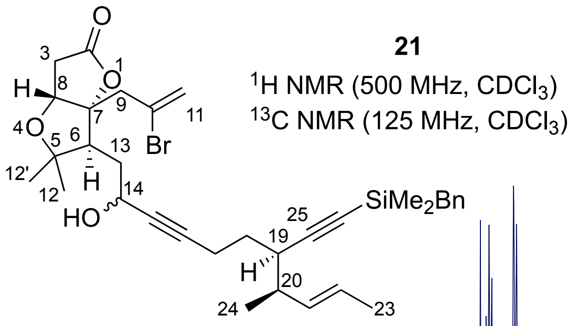

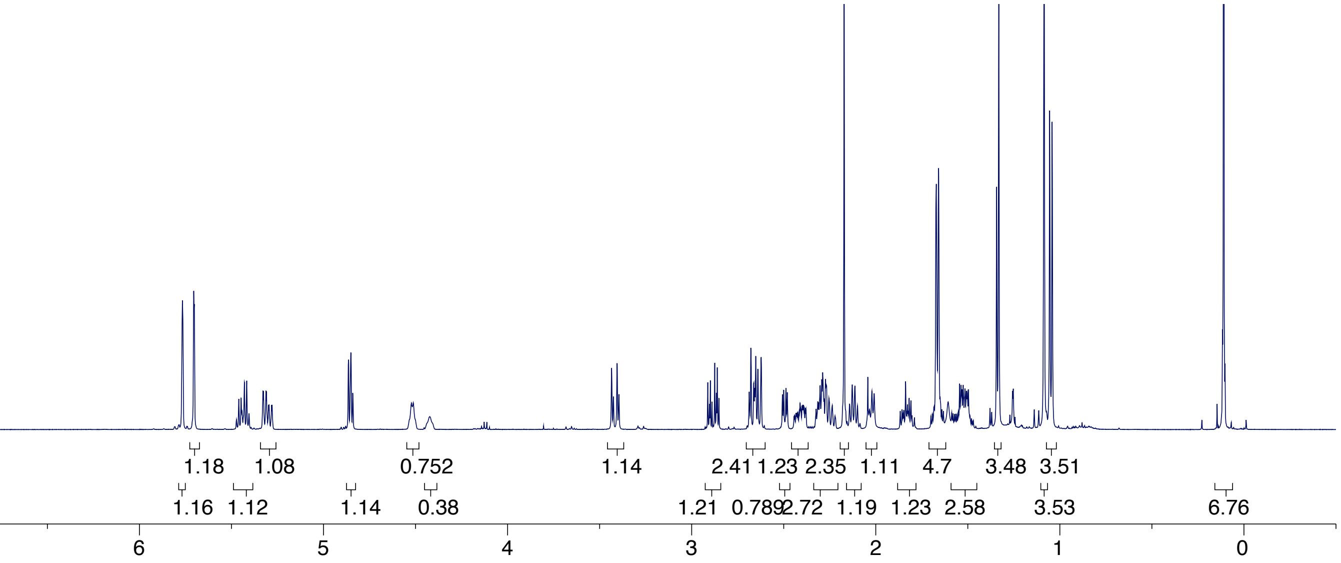

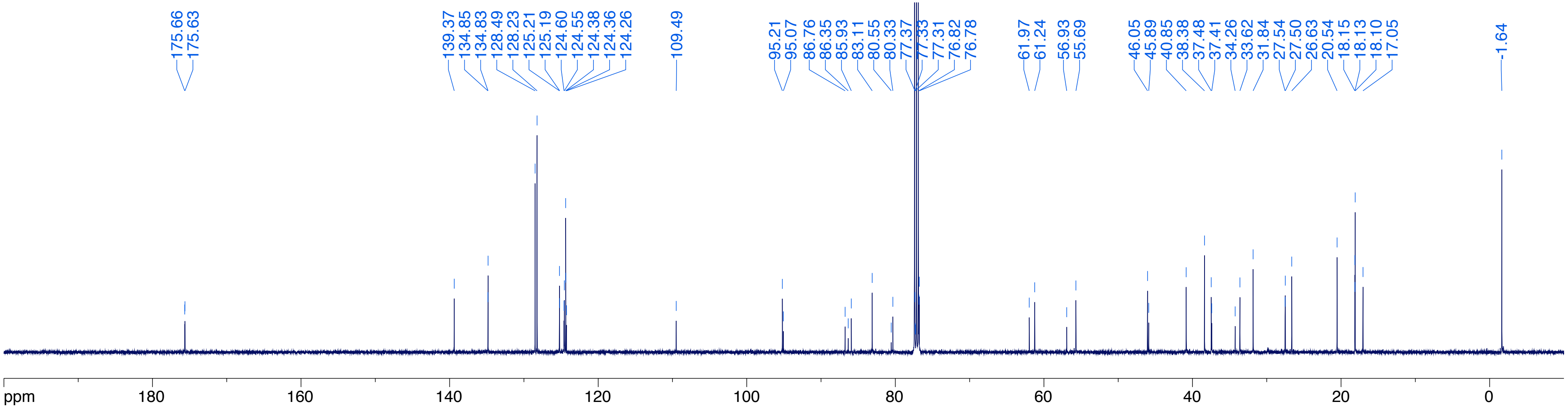

(3a*R*,6*S*,6a*R*)-6-((7*R*,8*R*,*E*)-7-((Benzyldimethylsilyl)ethynyl)-2-((*tert*-butyldimethylsilyl)oxy)-8-methylundeC9-en-3-yn-1-yl)-6a-(2-bromoallyl)-5,5-dimethyltetrahydrofuro[3,2-b]furan-2(3*H*)-one, S17

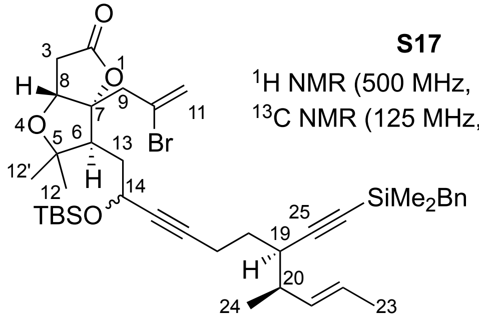

S17

^{1}H NMR (500 MHz, $CDCl_3$)

^{13}C NMR (125 MHz, $CDCl_3$)

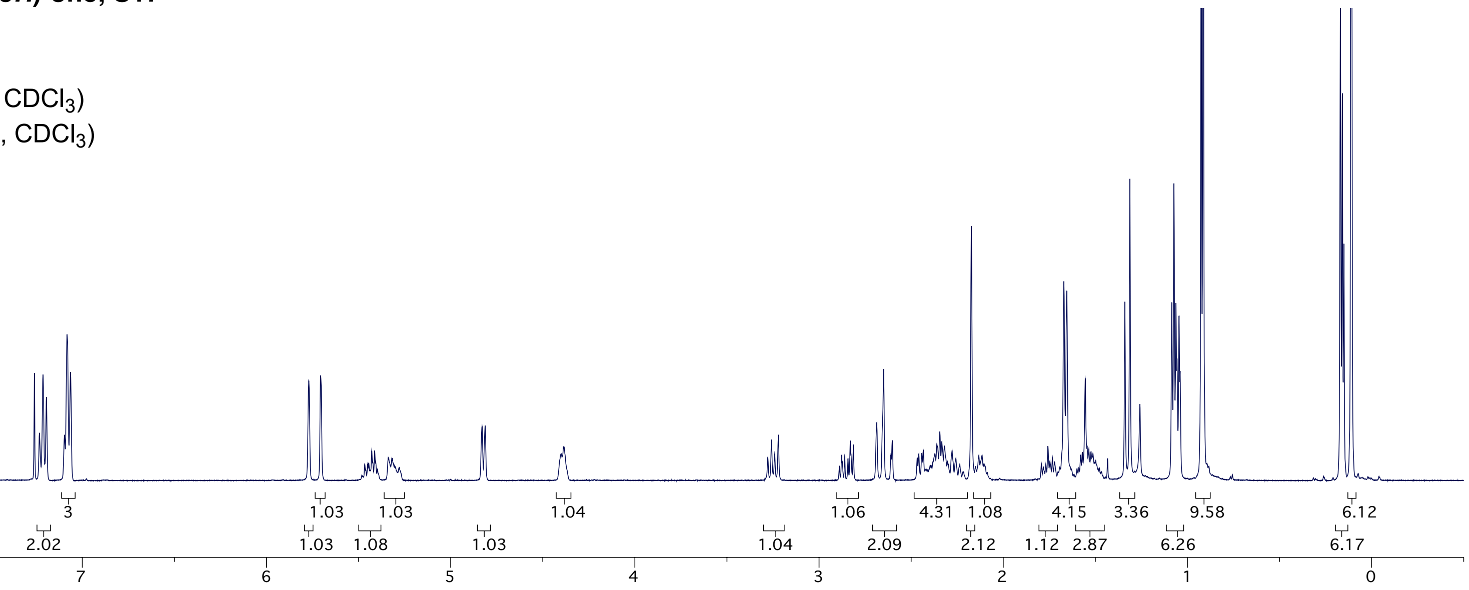

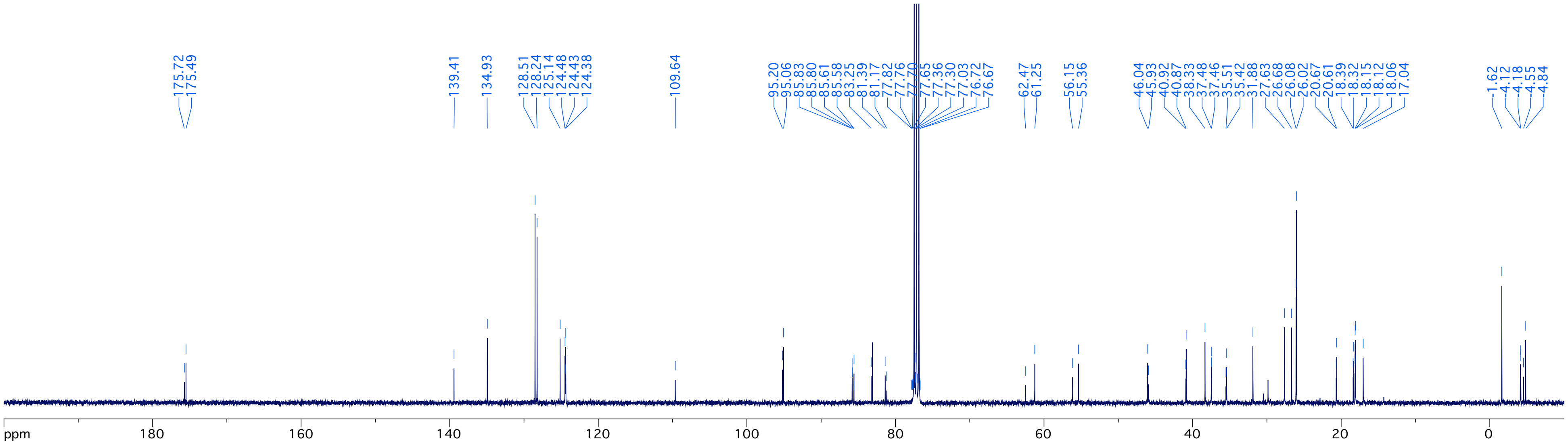

(3a*R*,5a*S*,10*R*,13a*R*)-11-(Benzyldimethylsilyl)-7-((*tert*-butyldimethylsilyl)oxy)-5,5-dimethyl-10-((*R*,*E*)-pent-3-en-2-yl)-3,3a,5,5a,6,7,8,9,10,13-decahydro-2*H*-furo[3,2-b]indeno[4',5':5,6]cyclohepta[1,2-c]furan-2-one, 22 – <u>*Spectra of diastereomer mixture which could be partially separated on the following 2 pages.*</u>

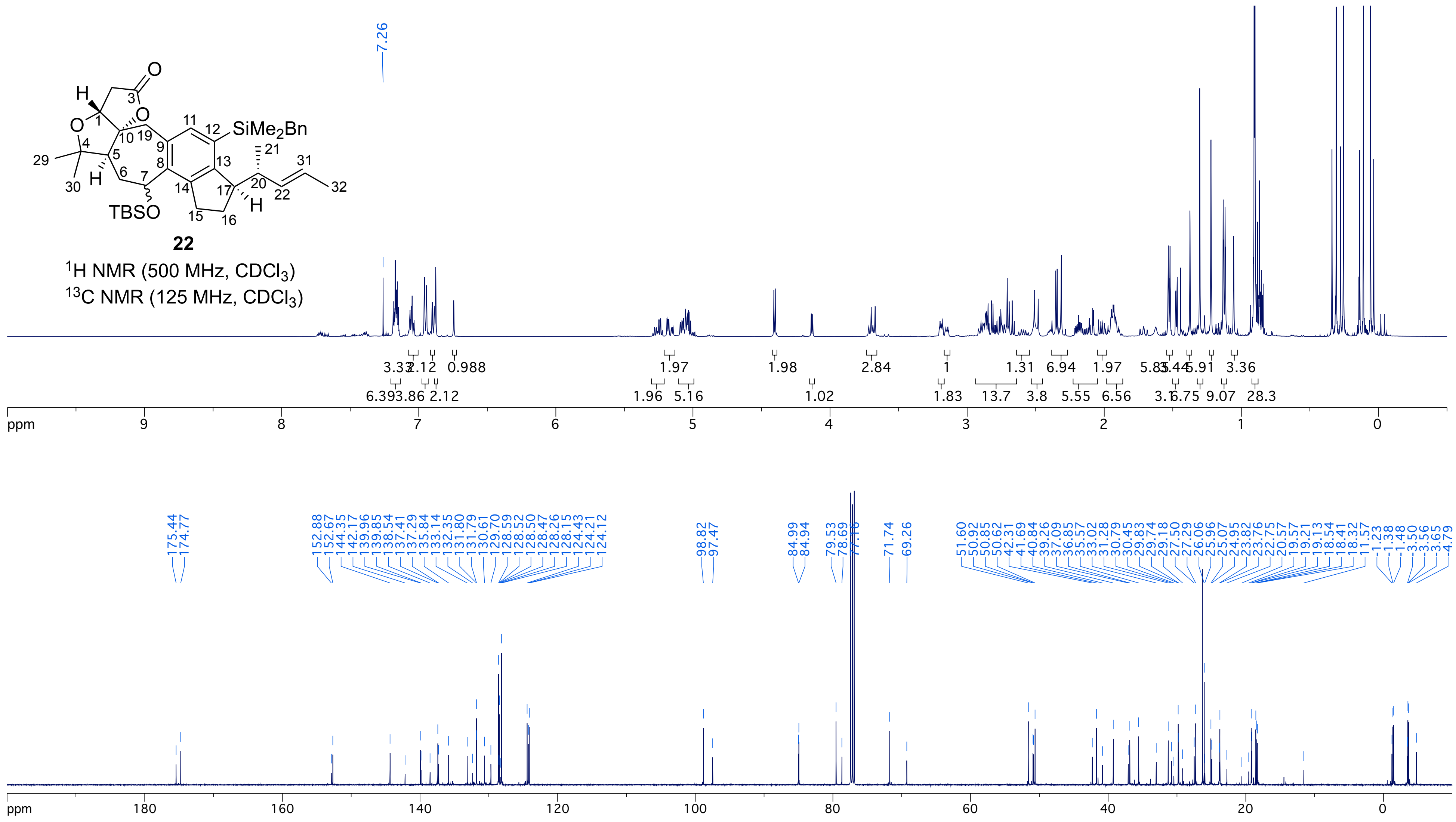

(3a*R*,5a*S*,10*R*,13a*R*)-11-(Benzyldimethylsilyl)-7-((*tert*-butyldimethylsilyl)oxy)-5,5-dimethyl-10-((*R*,*E*)-pent-3-en-2-yl)-3,3a,5,5a,6,7,8,9,10,13-decahydro-2*H*furo[3,2-b]indeno[4',5':5,6]cyclohepta[1,2-c]furan-2-one, 22

Spectra of the major diastereomer, contains some of the minor (C7) epimer:

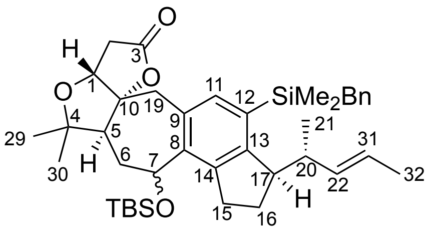

22

^{1}H NMR (500 MHz, $CDCl_3$)

^{13}C NMR (125 MHz, $CDCl_3$)

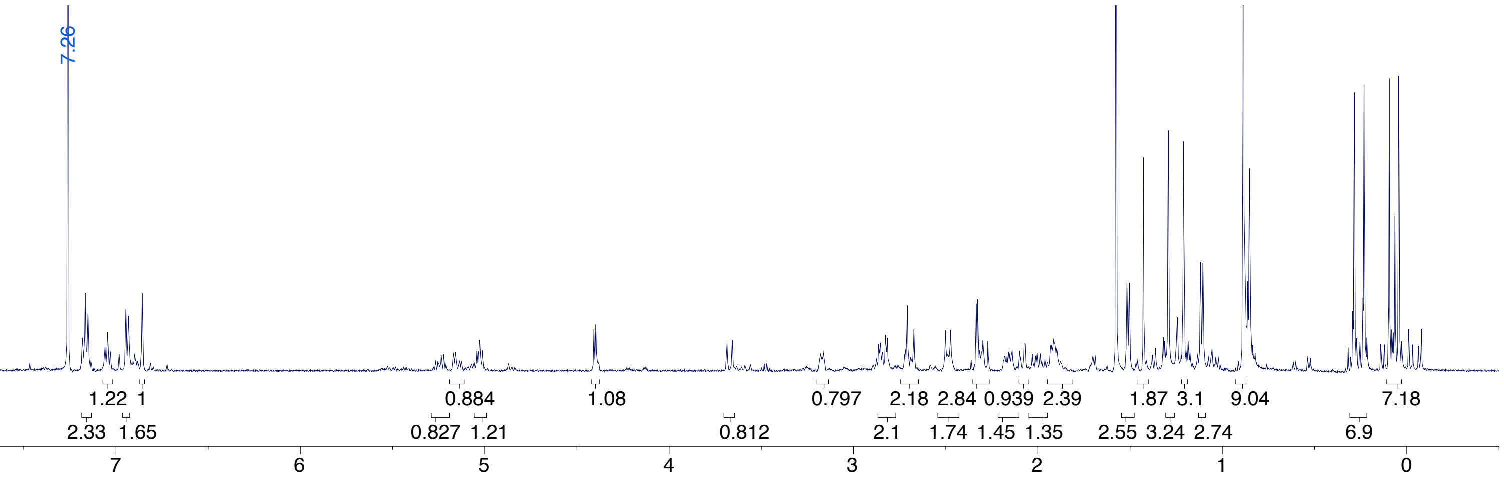

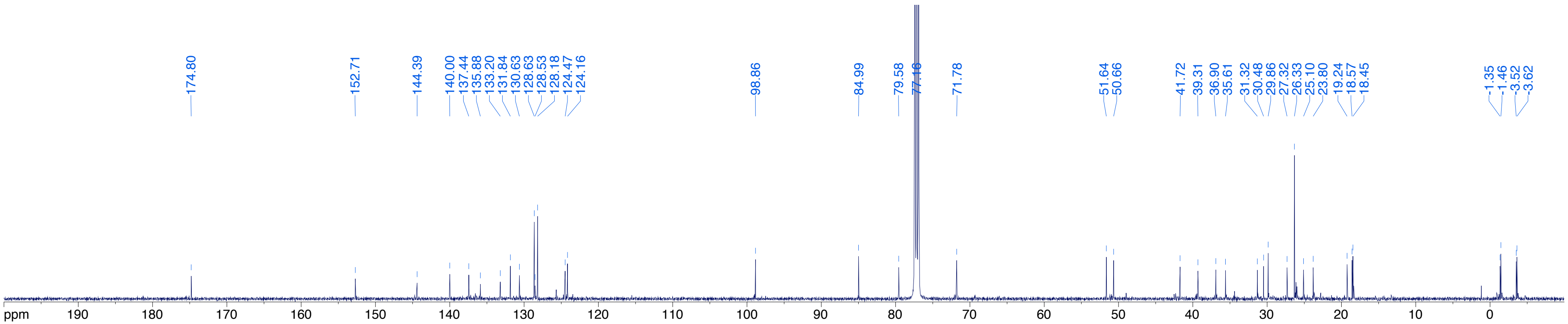

Spectra of the minor diastereomer:

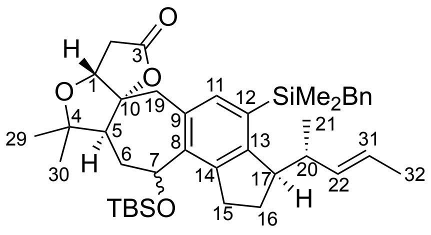

22

^{1}H NMR (500 MHz, $CDCl_3$)

^{13}C NMR (125 MHz, $CDCl_3$)

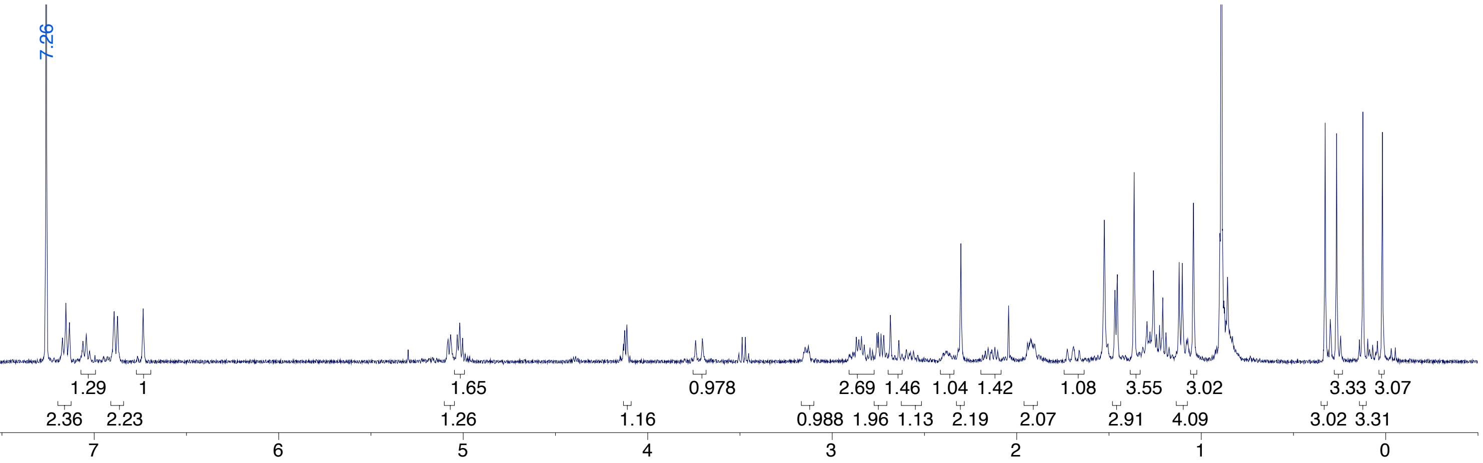

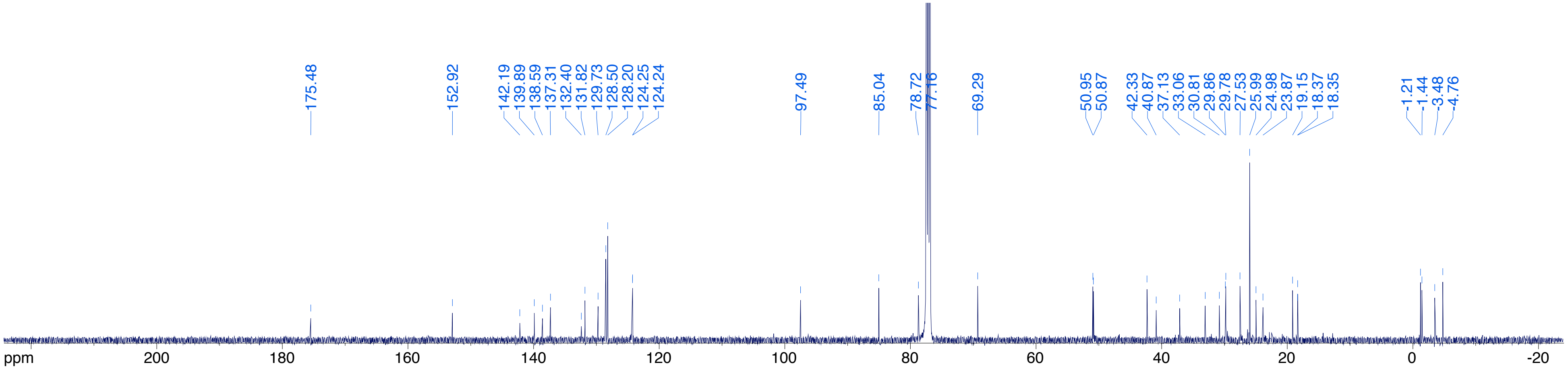

(3a*R*,5a*S*,10*R*,13a*R*)-11-hydroxy-5,5-dimethyl-10-((*R*,*E*)-pent-3-en-2-yl)-3,3a,5,5a,6,7,8,9,10,13-decahydro-2*H*-furo[3,2-b]indeno[4',5':5,6]cyclohepta[1,2-c]furan-2-one, 23

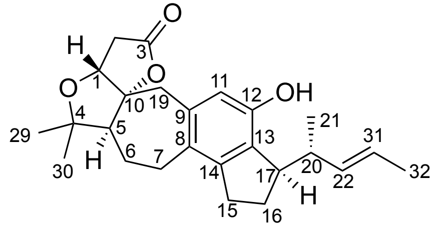

23

^{1}H NMR (500 MHz, $CDCl_3$)
^{13}C NMR (125 MHz, $CDCl_3$)

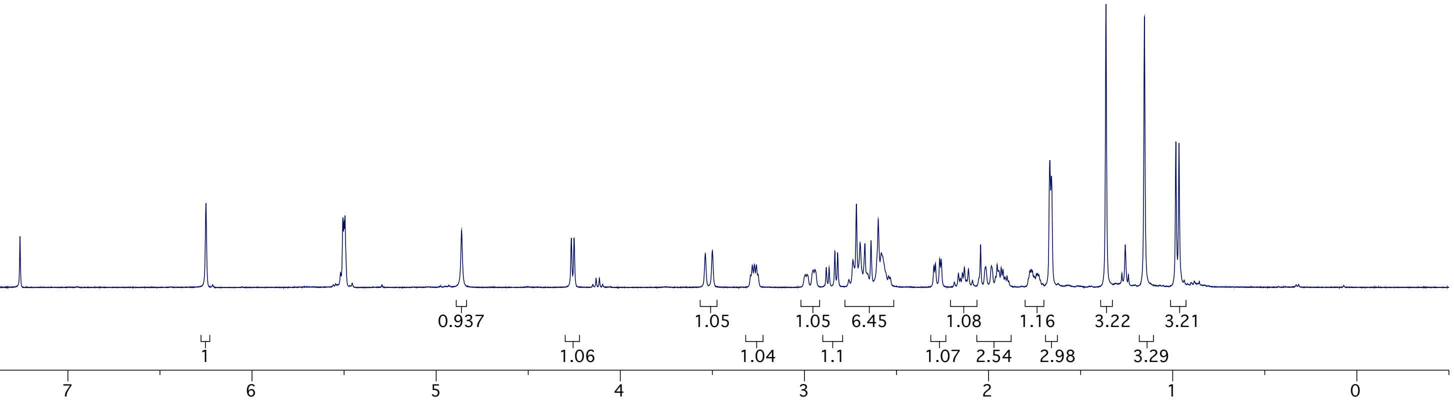

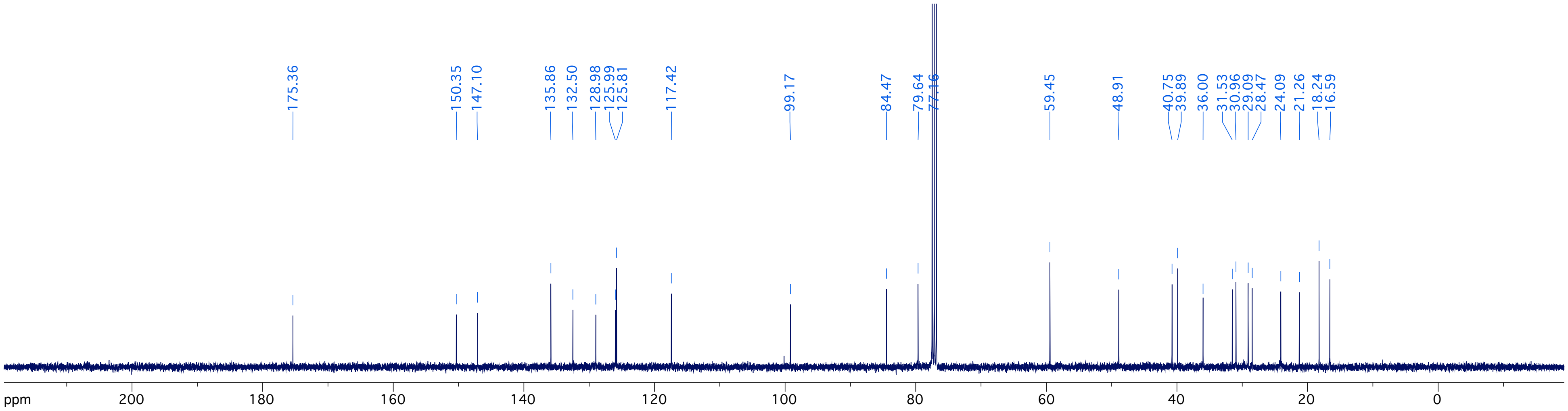

(3a*R*,6*S*,6a*R*)-6-((7*R*,8*R*,*E*)-7-((benzyldimethylsilyl)ethynyl)-2-hydroxy-8-methylundec-9-en-3-yn-1-yl)-5,5-dimethyl-6a-(prop-2-yn-1-yl)tetrahydrofuro[3,2-*b*]furan-2(5*H*)-one, 24

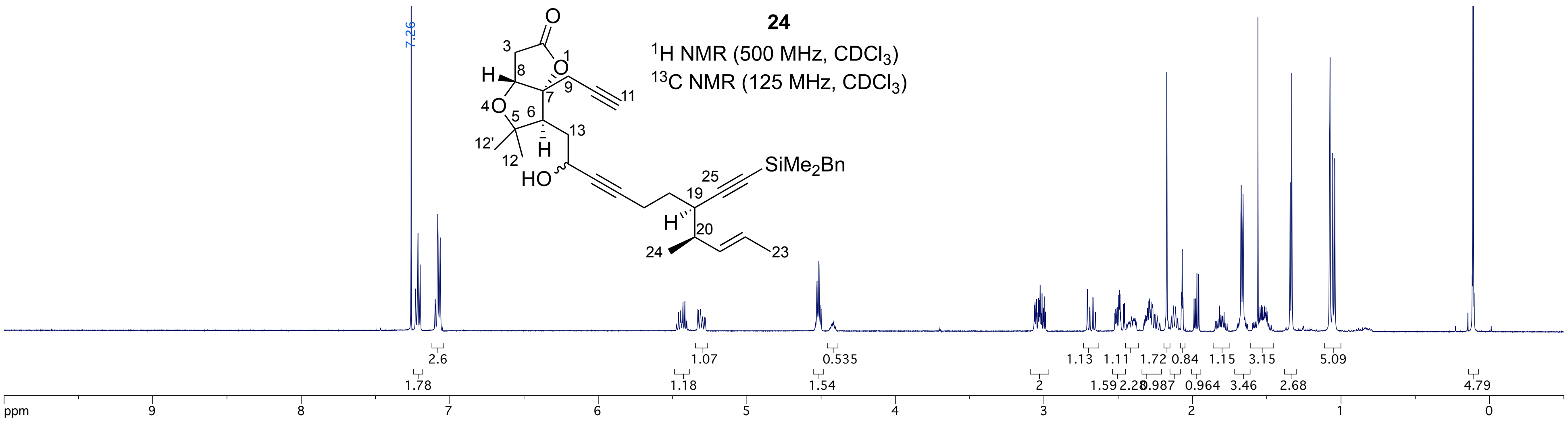

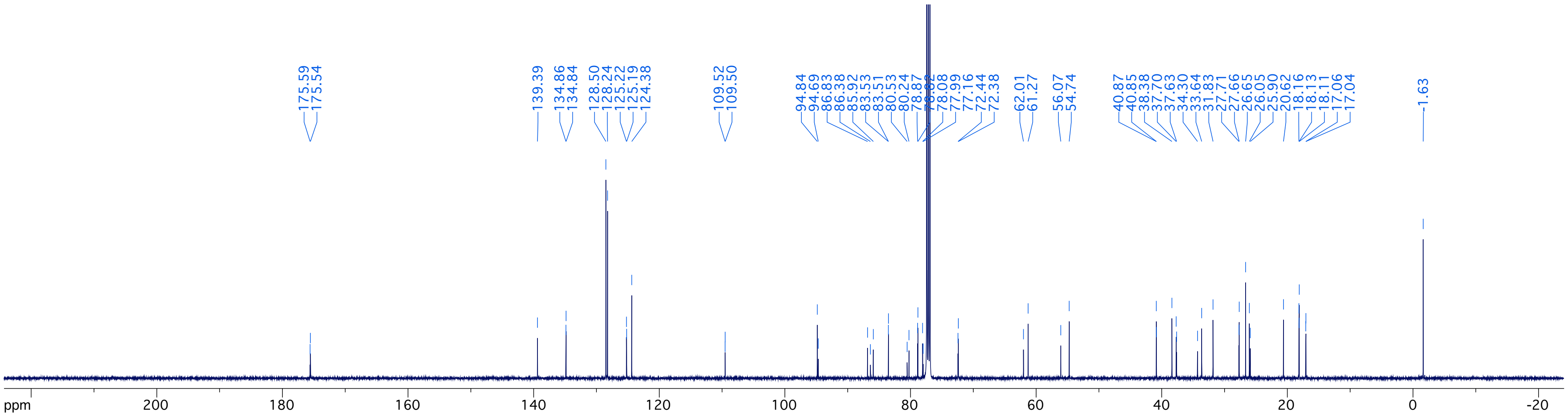

(3a*R*,5a*S*,7*S*,10*R*,13a*R*)-11-(benzyldimethylsilyl)-7-hydroxy-5,5-dimethyl-10-((*R*,*E*)-pent-3-en-2-yl)-3,3a,5,5a,6,7,8,9,10,13-decahydro-2*H*-furo[3,2-*b*]indeno[4',5':5,6]cyclohepta[1,2-*c*]furan-2-one, (7*S*)-25

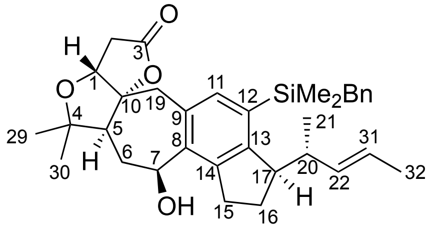

(7*S*)-25

^{1}H NMR (500 MHz, $CDCl_3$)

^{13}C NMR (125 MHz, $CDCl_3$)

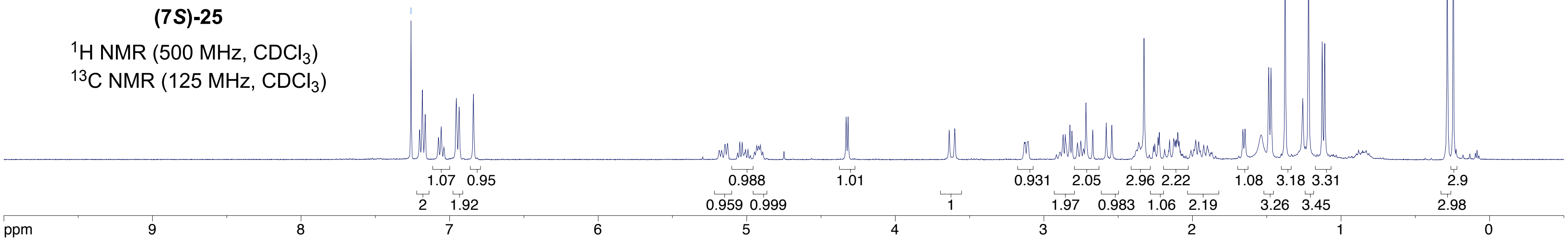

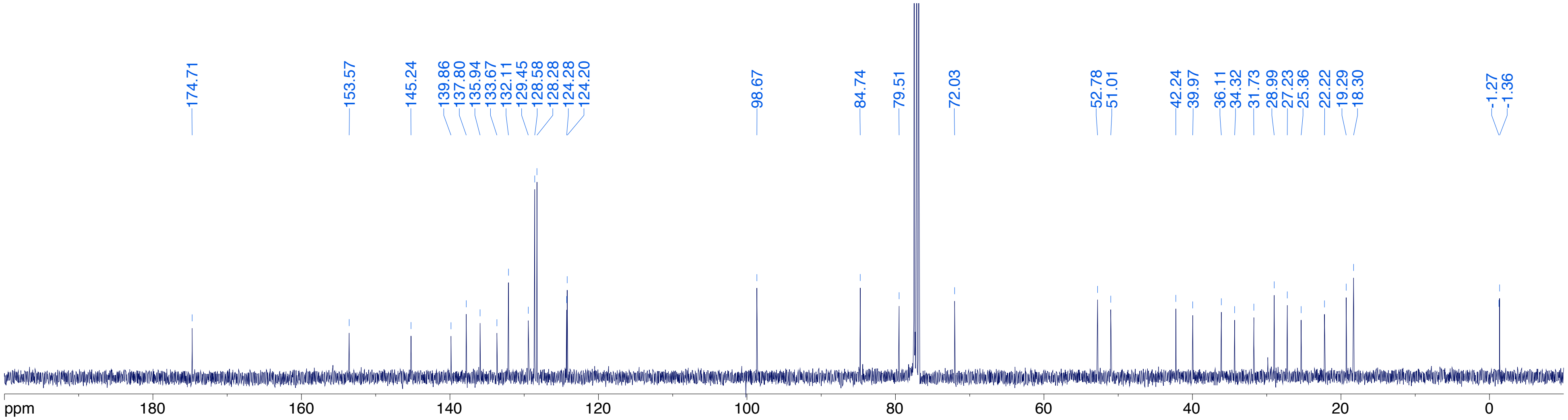

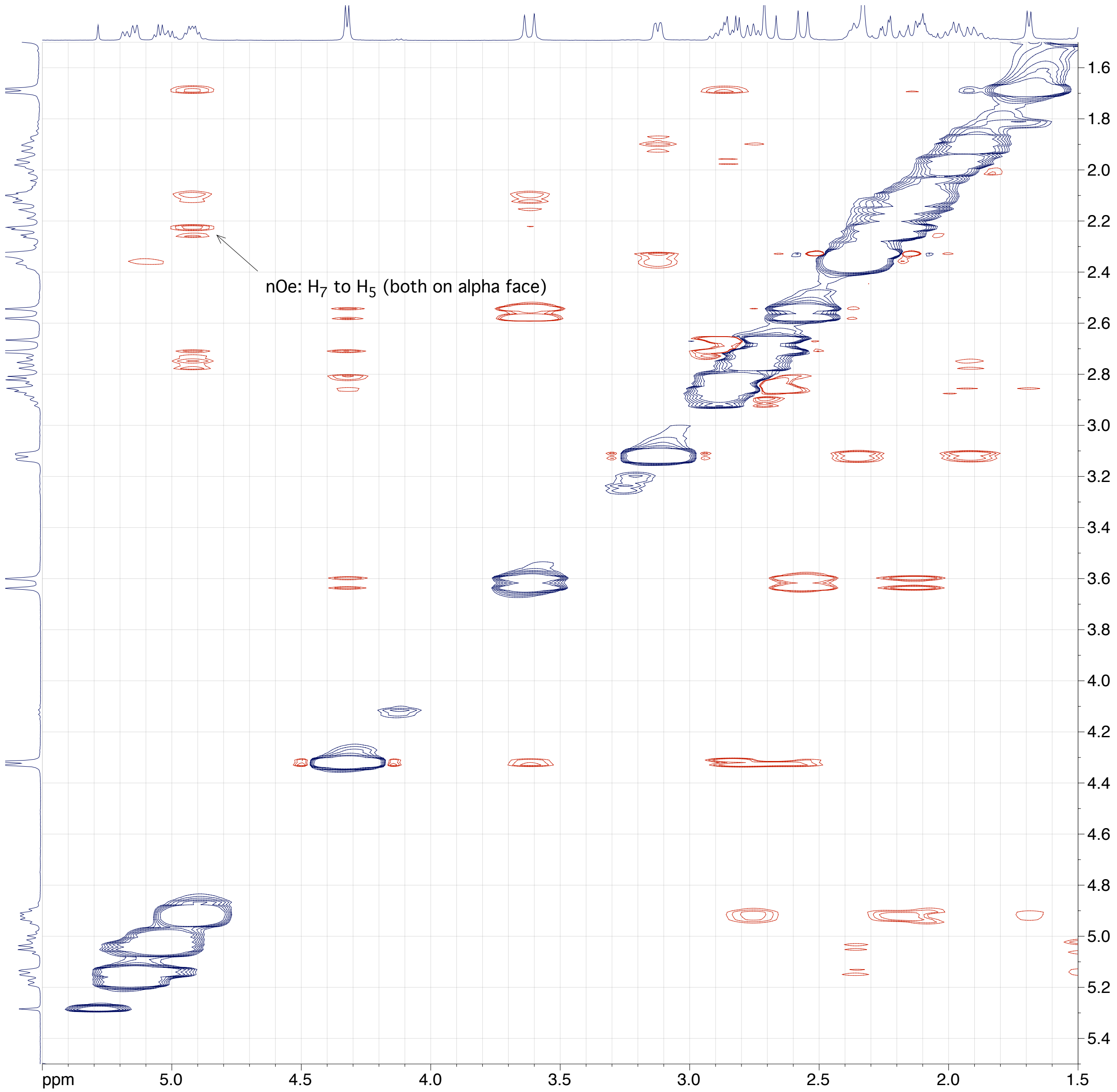
nOe: H_7 to H_5 (both on alpha face)
ppm
5.0
4.5
4.0
3.5
3.0
2.5
2.0
1.5
1.6
1.8
2.0
2.2
2.4
2.6
2.8
3.0
3.2
3.4
3.6
3.8
4.0
4.2
4.4
4.6
4.8
5.0
5.2
5.4

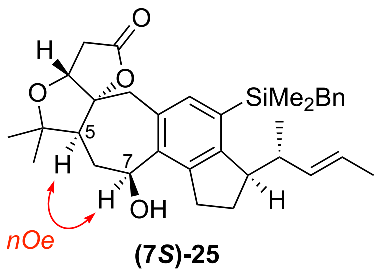
O
H
O
O
5
7
H
H
OH
$SiMe_2Bn$
H
nOe

(7*S*)-25

(3a*R*,5a*S*,7*R*,10*R*,13a*R*)-11-(benzyldimethylsilyl)-7-hydroxy-5,5-dimethyl-10-((*R*,*E*)-pent-3-en-2-yl)-3,3a,5,5a,6,7,8,9,10,13-decahydro-2*H*-furo[3,2-*b*]indeno[4',5':5,6]cyclohepta[1,2-*c*]furan-2-one, (7*R*)-25, 85:15 mixture with terminal alkene isomer

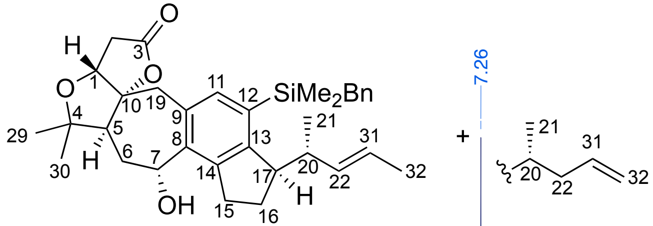

(7*R*)-25 + 25-ter (85:15)

^{1}H NMR (500 MHz, $CDCl_3$)

^{13}C NMR (125 MHz, $CDCl_3$)

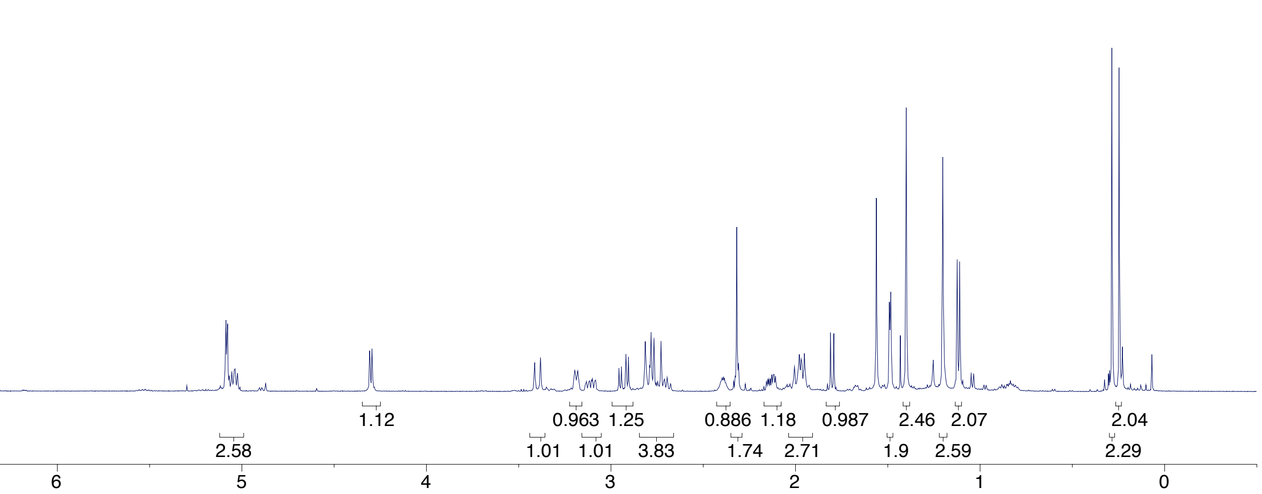

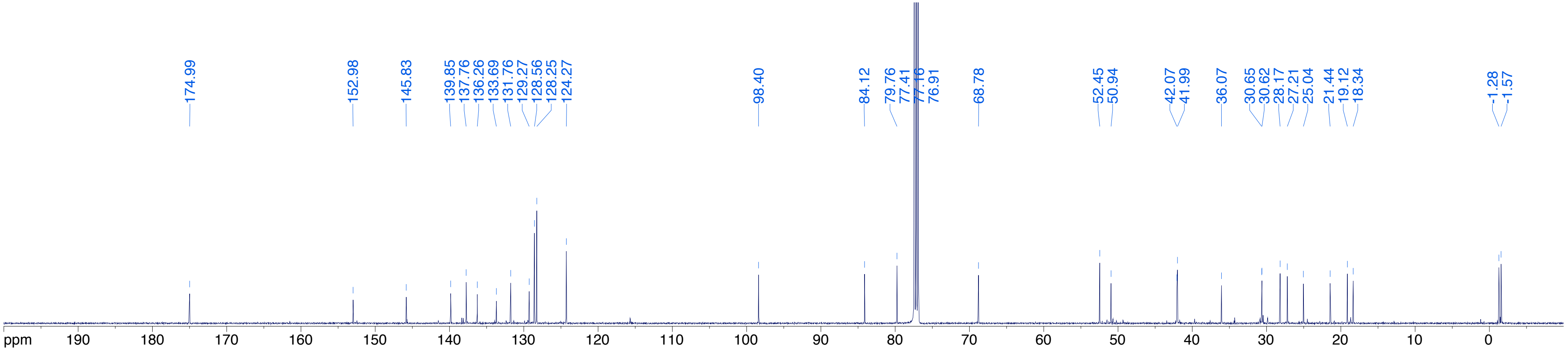

(3a*R*,5a*S*,7*S*,10*R*,13a*R*)-7,11-Dihydroxy-5,5-dimethyl-10-((*R*,*E*)-pent-3-en-2-yl)-3,3a,5,5a,6,7,8,9,10,13-decahydro-2*H*-furo[3,2-b]indeno[4',5':5,6]cyclohepta[1,2-c]furan-2-one, (7*S*)-S18

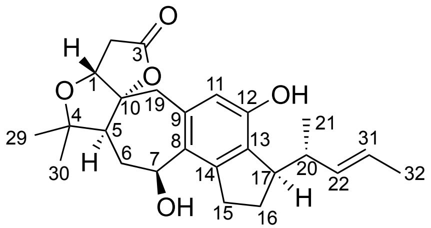

(7*S*)-S18

^{1}H NMR (500 MHz, $CDCl_3$)

^{13}C NMR (125 MHz, $CDCl_3$)

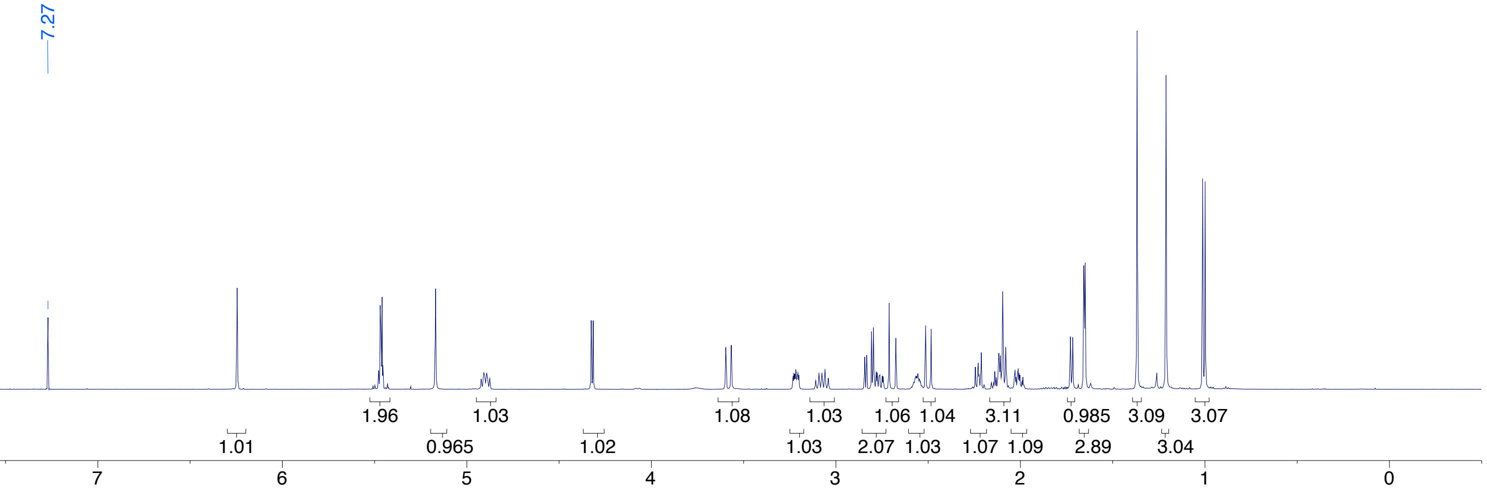

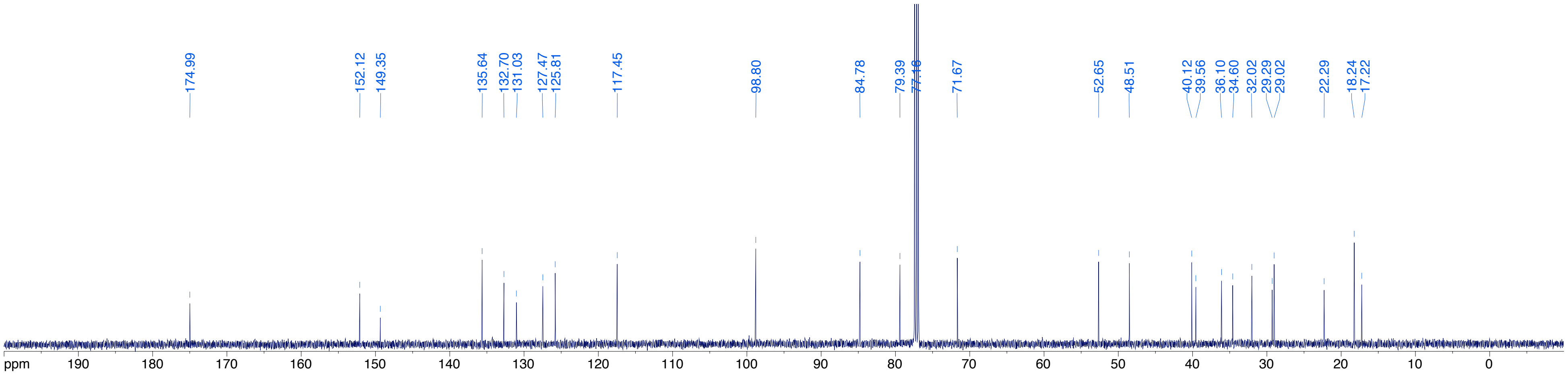

(3a*R*,5a*S*,7*R*,10*R*,13a*R*)-7,11-Dihydroxy-5,5-dimethyl-10-((*R*,*E*)-pent-3-en-2-yl)-3,3a,5,5a,6,7,8,9,10,13-decahydro-2*H*-furo[3,2-b]indeno[4',5':5,6]cyclohepta[1,2-c]furan-2-one, (7*R*)-S18, 85:15 mixture with terminal alkene isomer

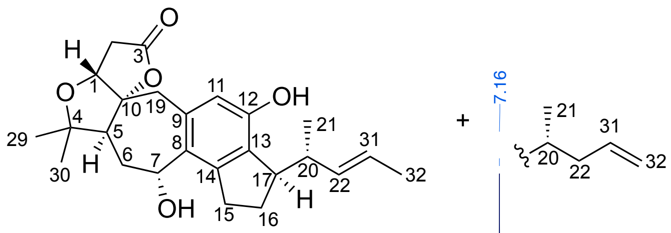

(7*R*)-S18 + S18-ter (85:15)

^{1}H NMR (500 MHz, $CDCl_3$)

^{13}C NMR (125 MHz, $CDCl_3$)

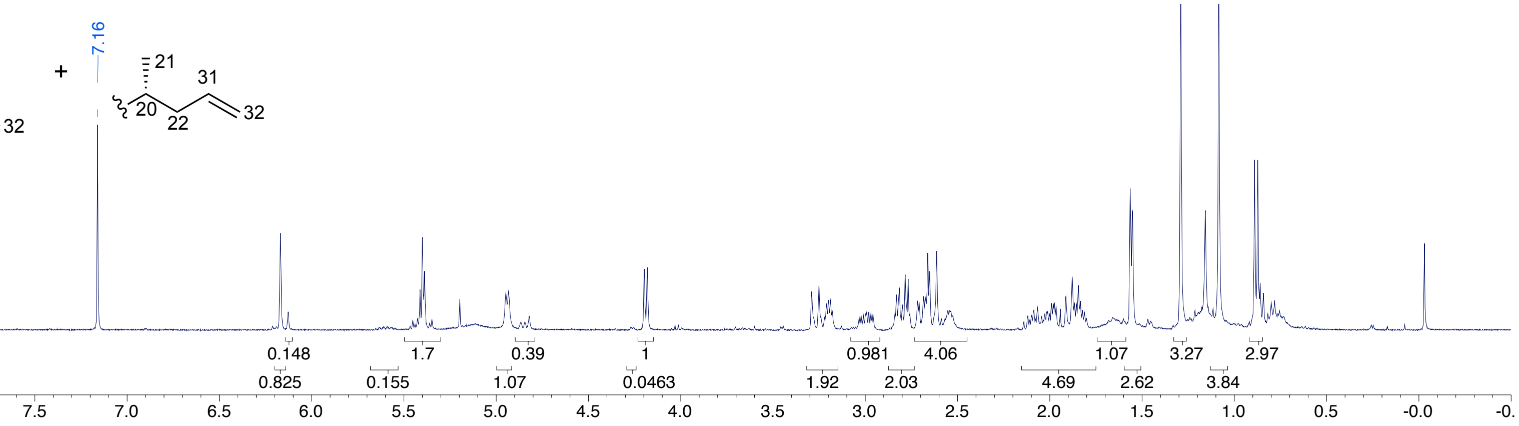

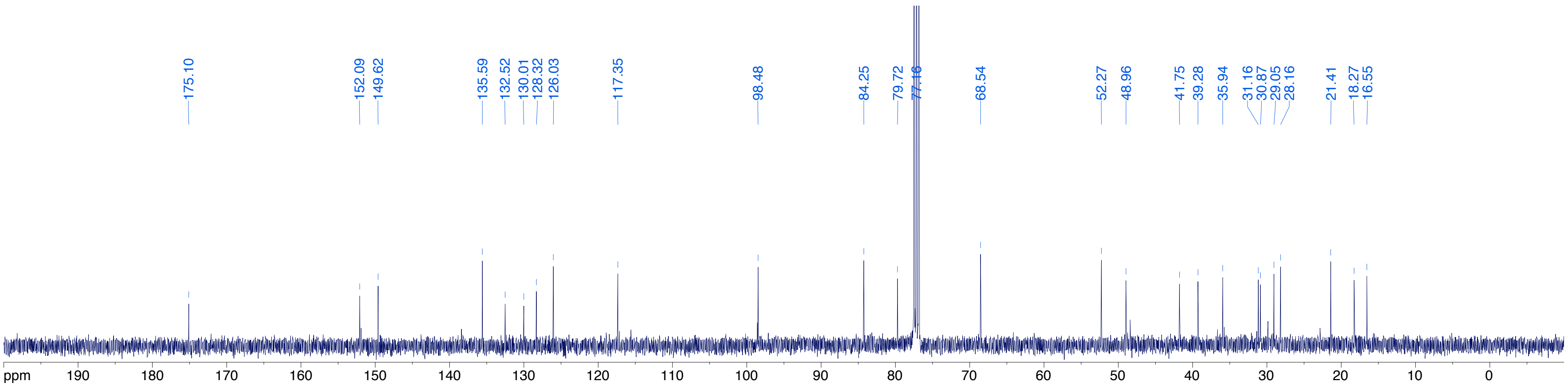

(3a*R*,6*S*,6a*R*)-6-((7*R*,8*R*,*E*)-7-((benzyldimethylsilyl)ethynyl)-2-((*tert*-butyldimethylsilyl)oxy)-8-methylundec-9-en-3-yn-1-yl)-5,5-dimethyl-6a-(prop-2-yn-1-yl)tetrahydrofuro[3,2-*b*]furan-2(5*H*)-one, S19

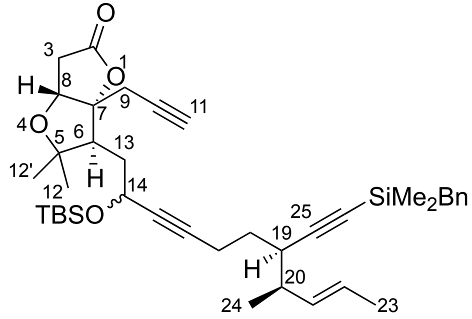

S19

^{1}H NMR (500 MHz, $CDCl_3$)

^{13}C NMR (125 MHz, $CDCl_3$)

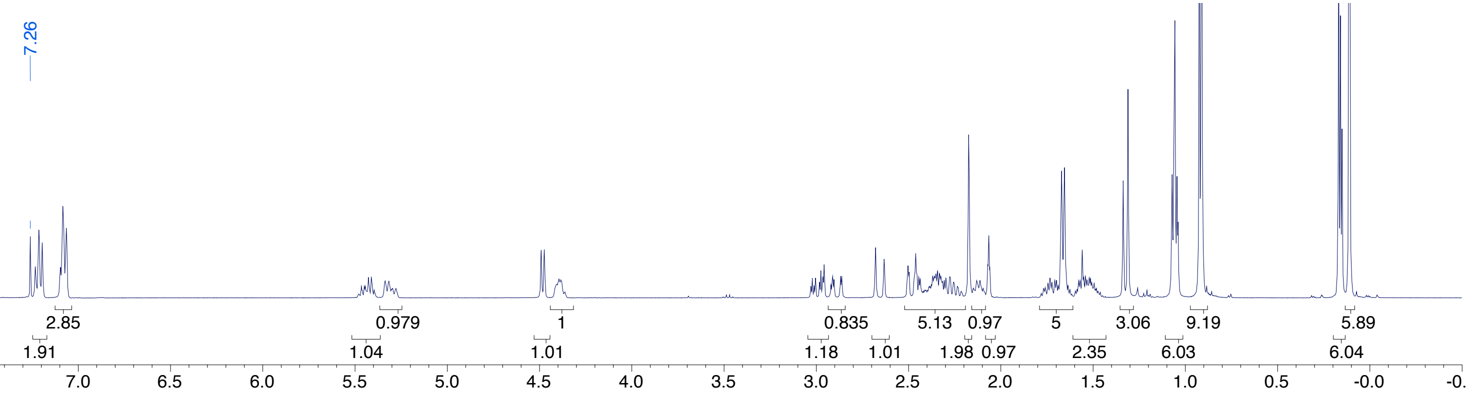

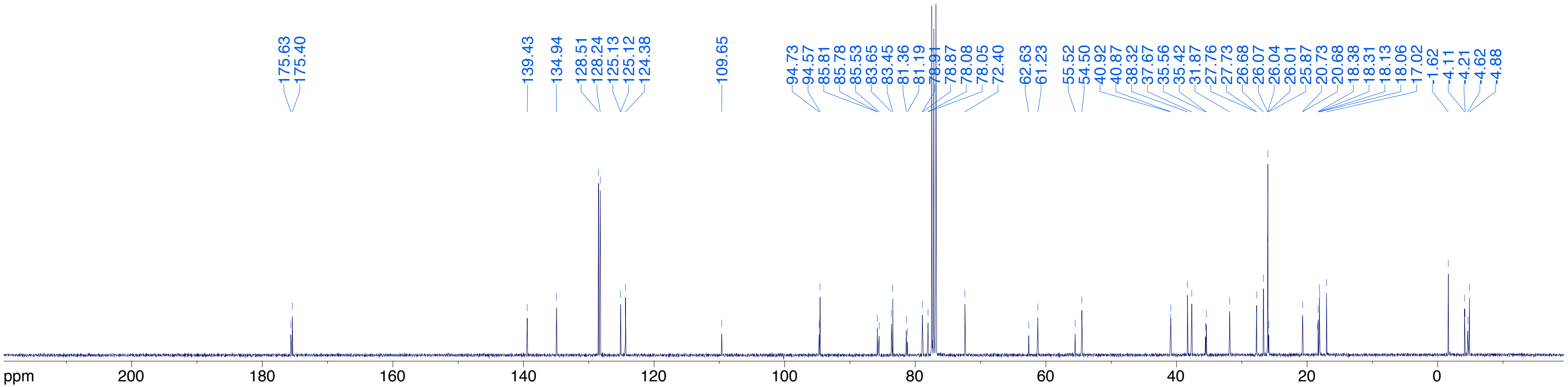

(3a*R*,5a*S*,10*R*,13a*R*)-10-((2*S*)-3,4-Dihydroxypentan-2-yl)-11-hydroxy-5,5-dimethyl-3,3a,5,5a,6,7,8,9,10,13-decahydro-2*H*-furo[3,2-b]indeno[4',5':5,6]cyclohepta[1,2-c]furan-2-one, S20, 'Diastereomer 1'

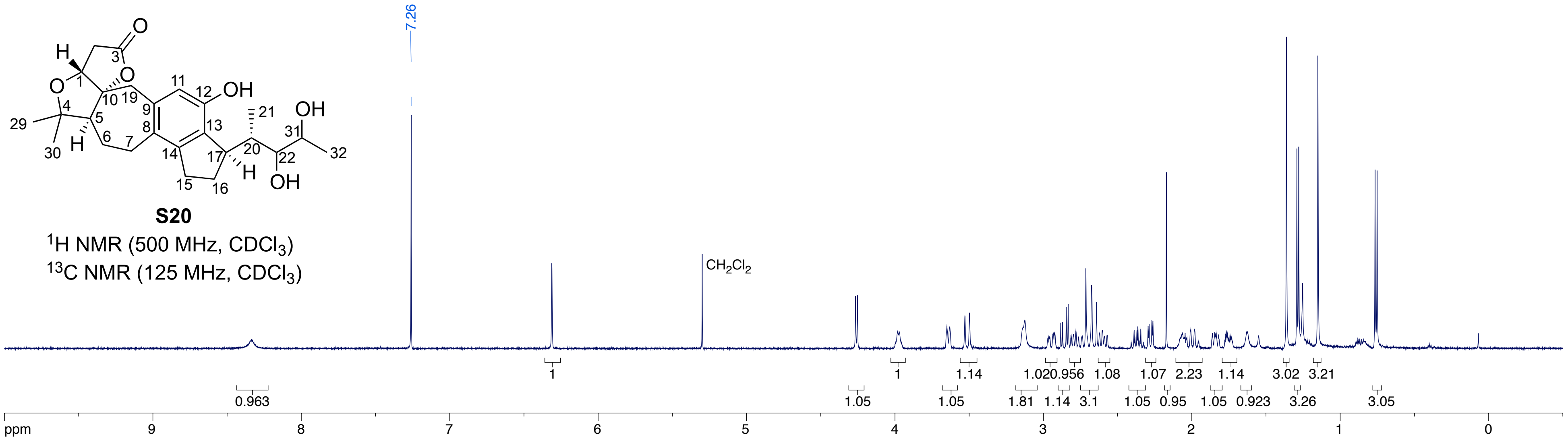

S20

^{1}H NMR (500 MHz, $CDCl_3$)

^{13}C NMR (125 MHz, $CDCl_3$)

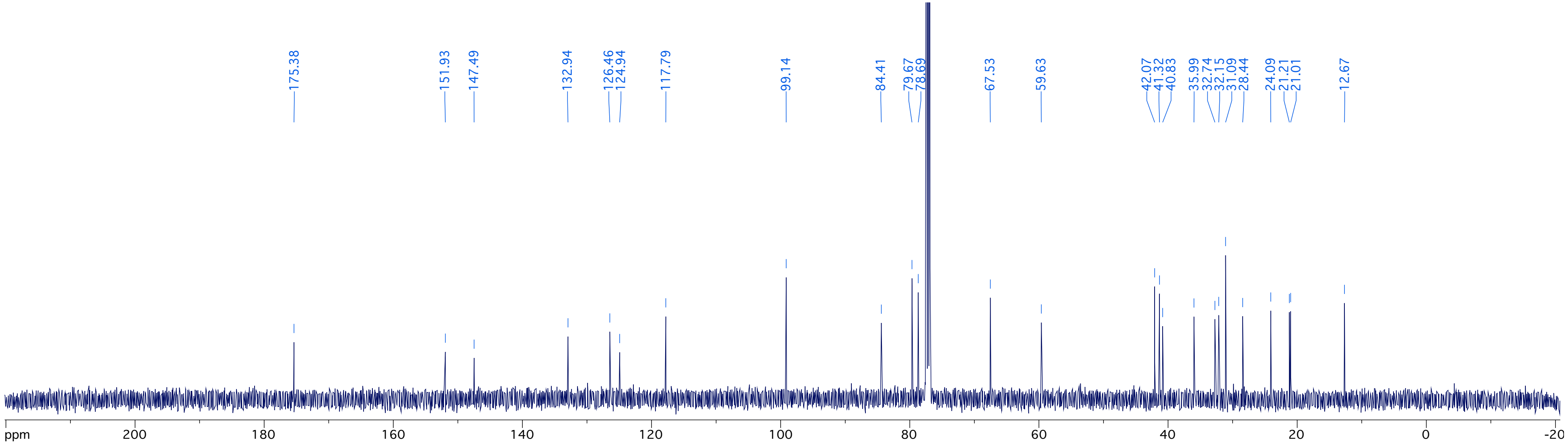

(3a*R*,5a*S*,10*R*,13a*R*)-10-((2*S*)-3,4-Dihydroxypentan-2-yl)-11-hydroxy-5,5-dimethyl-3,3a,5,5a,6,7,8,9,10,13-decahydro-2*H*-furo[3,2-b]indeno[4',5':5,6]cyclohepta[1,2-c]furan-2-one, S20, 'Diastereomer 2'

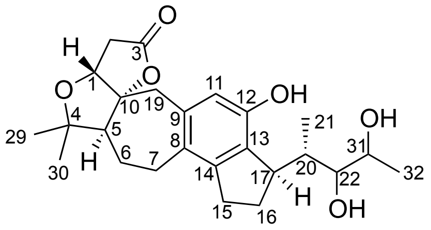

S20

^{1}H NMR (500 MHz, $CDCl_3$)

^{13}C NMR (125 MHz, $CDCl_3$)

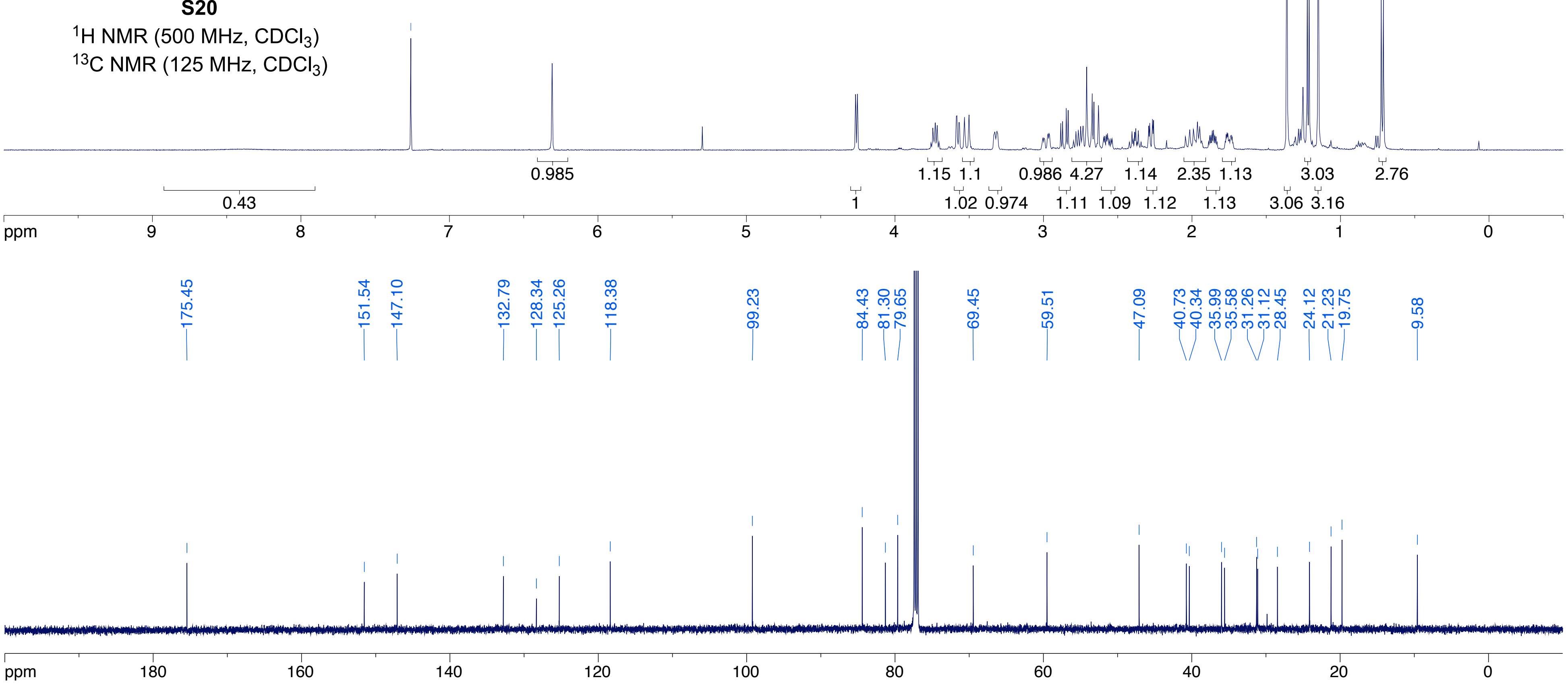

Equilibrium mixture of aldehyde (*S*)-2-((3a*R*,5a*S*,10*R*,13a*R*)-11-hydroxy-5,5-dimethyl-2-oxo-3,3a,5,5a,6,7,8,9,10,13-decahydro-2*H*-furo[3,2-b]indeno[4',5':5,6]cyclohepta[1,2-c]furan-10-yl)propanal, and lactols (3a*R*,5a*S*,9a*R*,10*S*,14a*R*)-11-Hydroxy-5,5,10-trimethyl-3,3a,5,5a,6,7,8,9,9a,10,11,14-dodecahydro-2*H*-cyclopenta[de]furo[3'',2'':2',3']furo[3',4':4,5] cyclohepta[1,2-g]chromen-2-one, 26

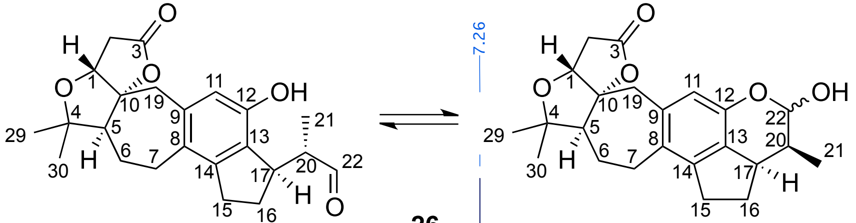

26

^{1}H NMR (500 MHz, $CDCl_3$)

^{13}C NMR (125 MHz, $CDCl_3$)

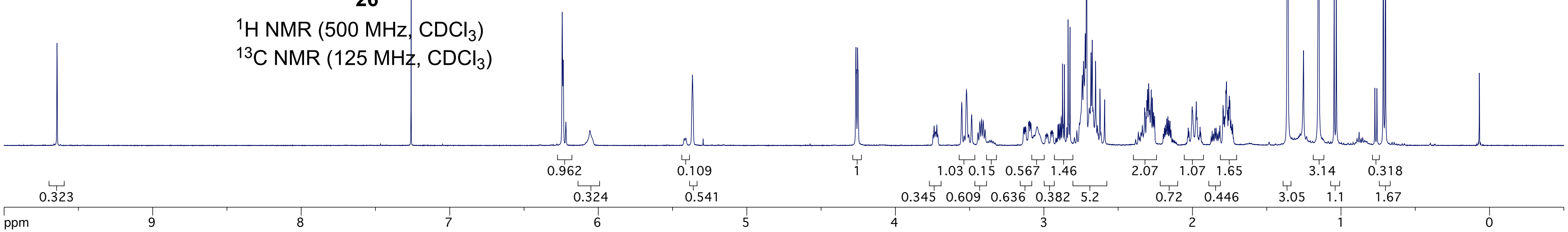

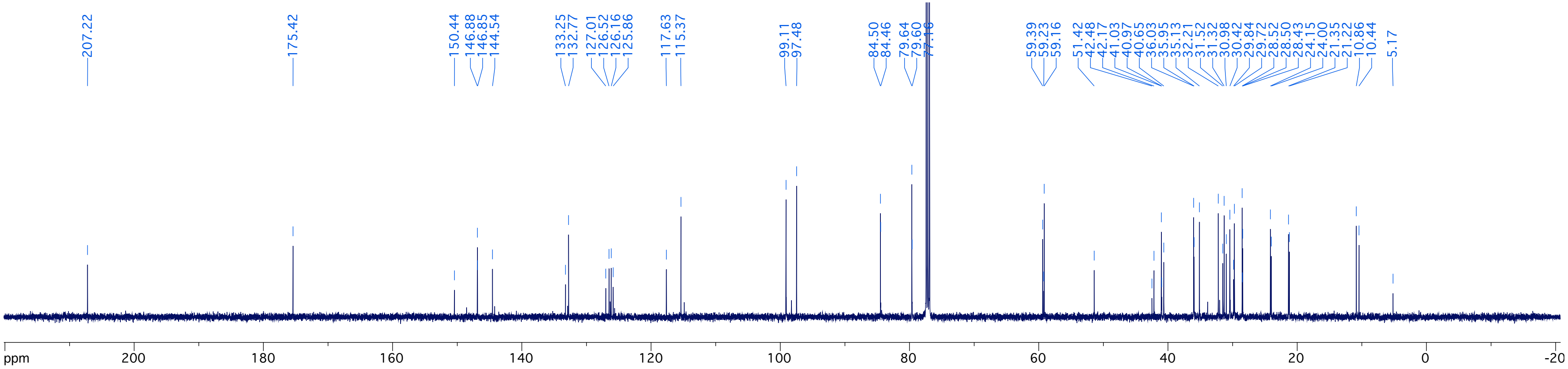

(3a*R*,5a*S*,9a*R*,10*S*,14a*R*)-11-Chloro-5,5,10-trimethyl-3,3a,5,5a,6,7,8,9,9a,10,11,14-dodecahydro-2*H*-cyclopenta[de]furo[3'',2'':2',3']furo[3',4':4,5]cyclohepta[1,2-g]chromen-2-one, 27

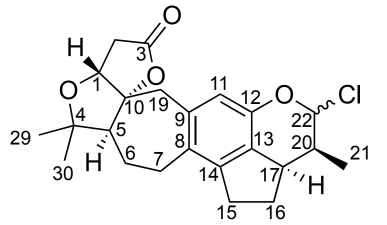

27

^{1}H NMR (500 MHz, $CDCl_3$)

^{13}C NMR (125 MHz, $CDCl_3$)

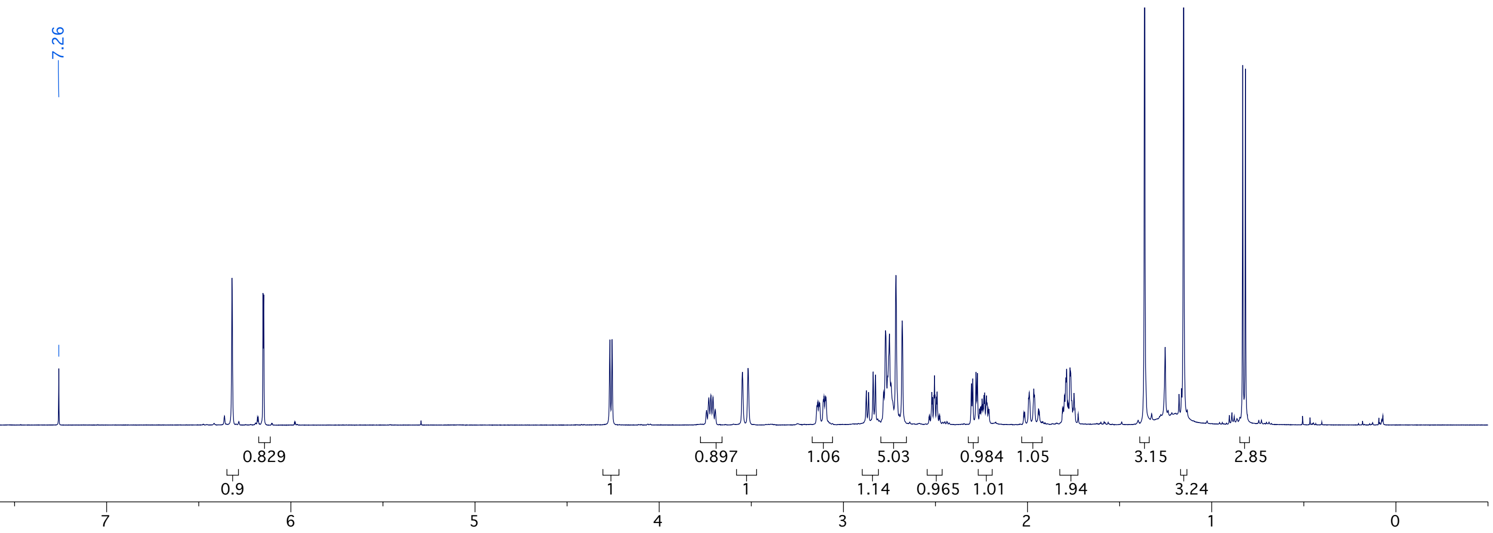

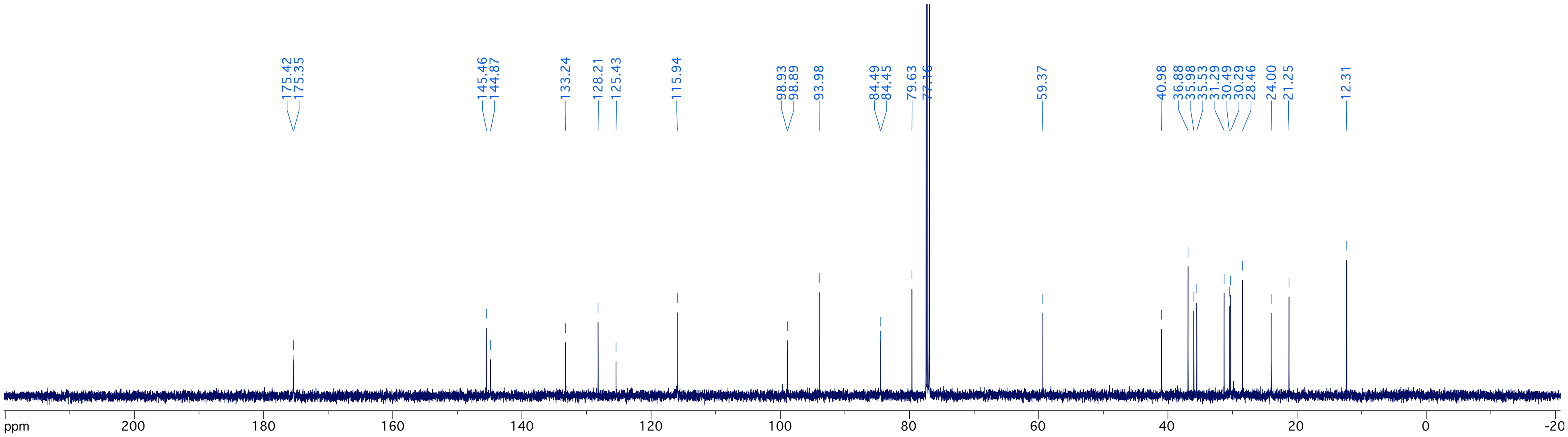

3.4 *Spectra for rubriflordilactone A and C23-epi- rubriflordilactone A*

Rubriflordilactone A, 1

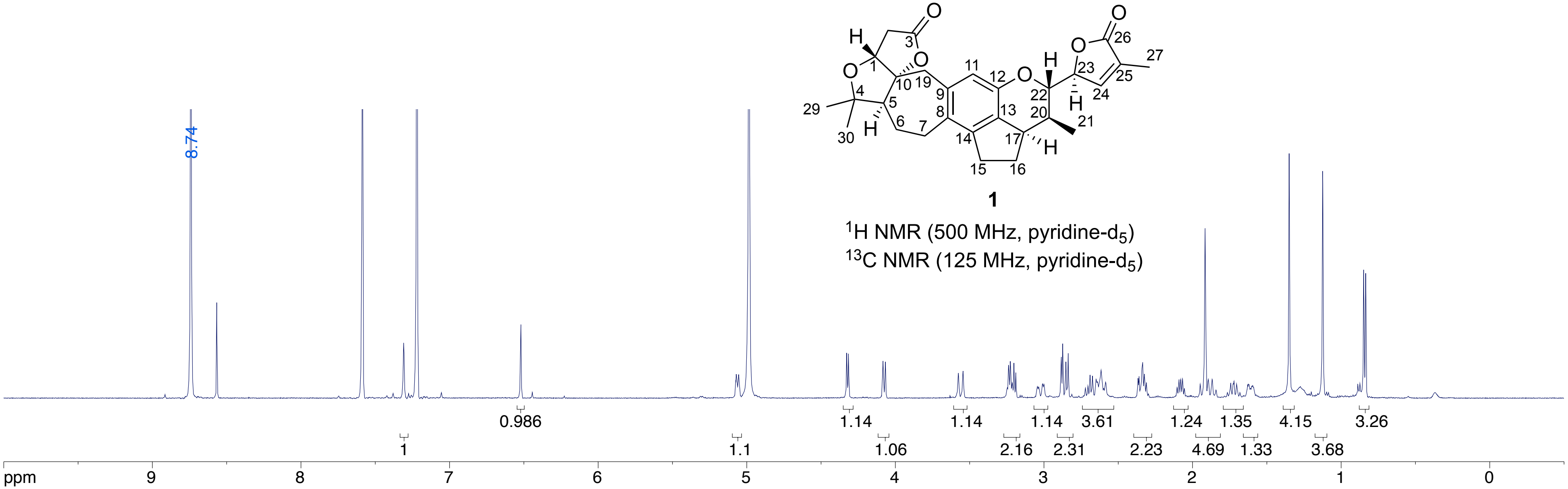

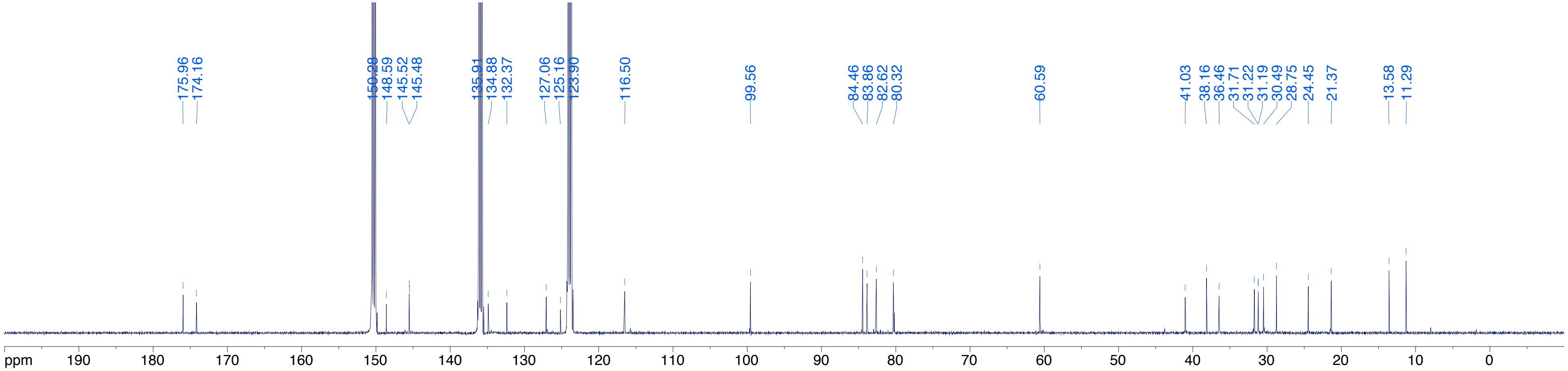

Rubriflordilactone A, HSQC spectrum (500 MHz, py). *This allows assignment of methylene pairs at H15, H16 and H7; and methines at H5, H20.*

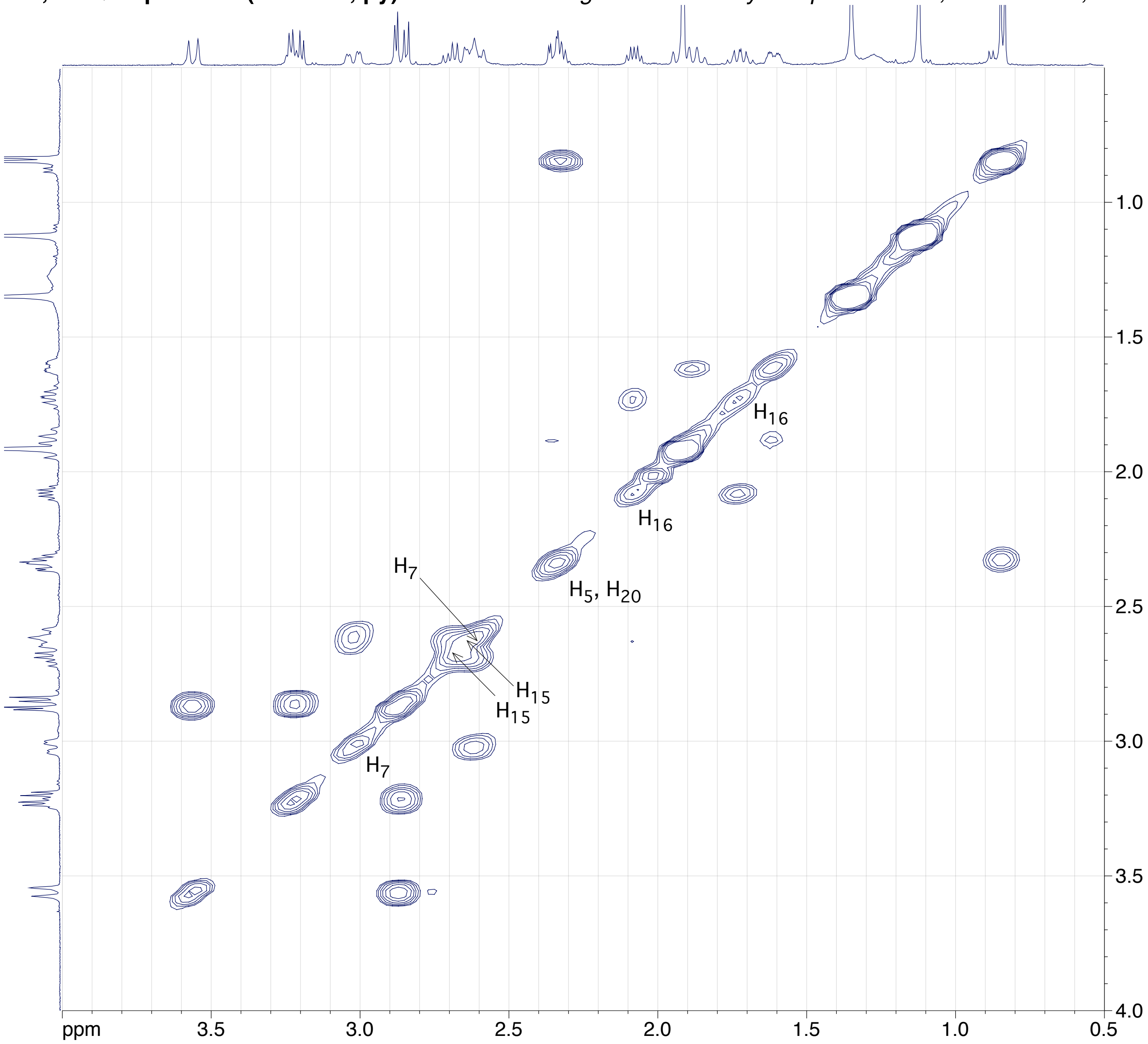

Rubriflordilactone A, HSQC spectrum (500 MHz, py). *This allows reassignment of 15α, 15β, 16α, 16β by correlations with C15 and C16.*

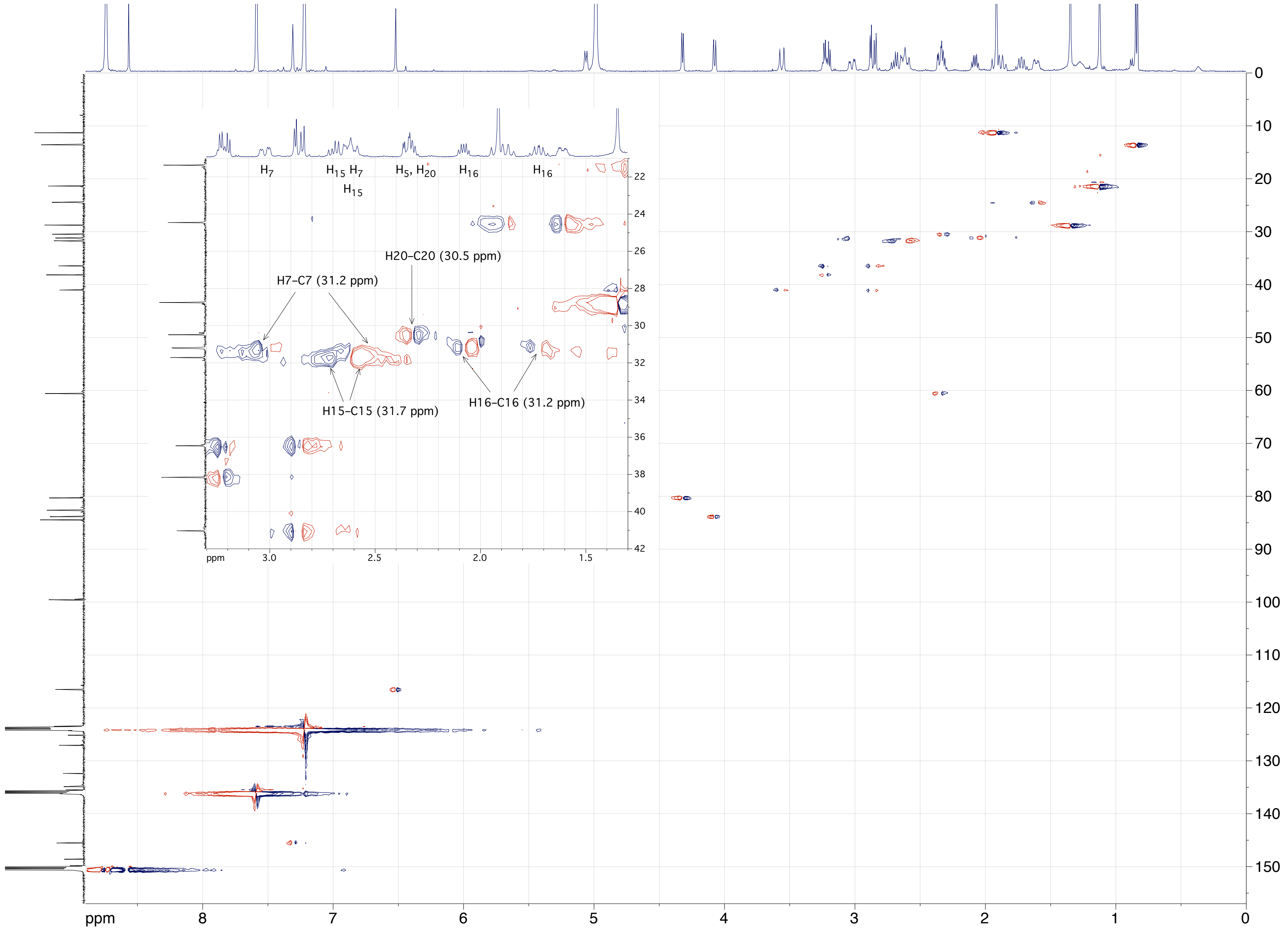

C23-*epi*-Rubriflordilactone A, 30 Note: this purified compound contains a small quantity of an additional inseparable diastereomer (at C22).

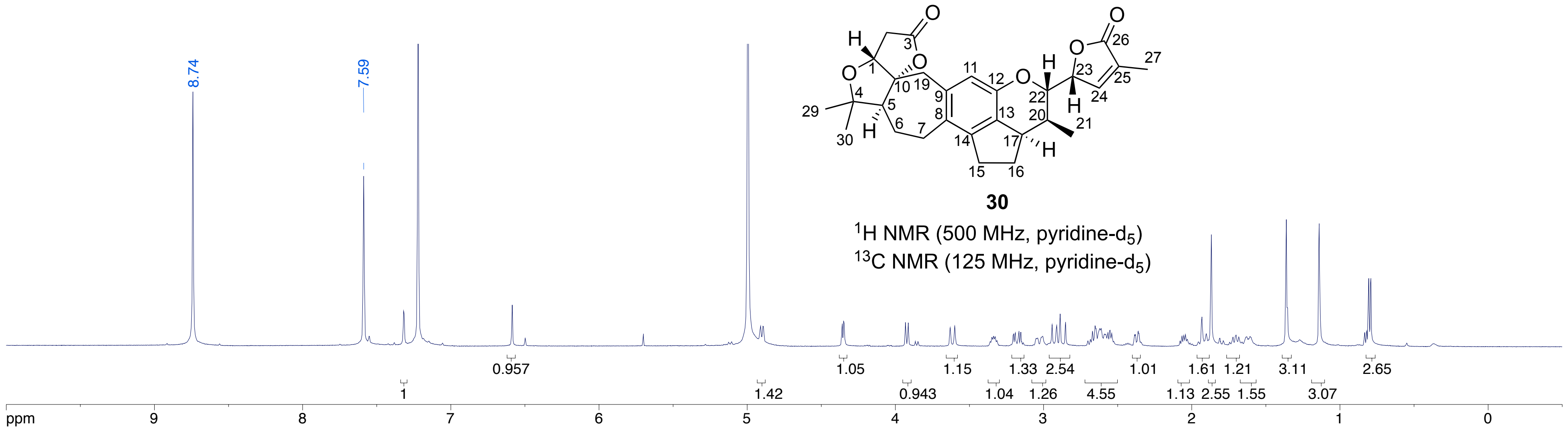

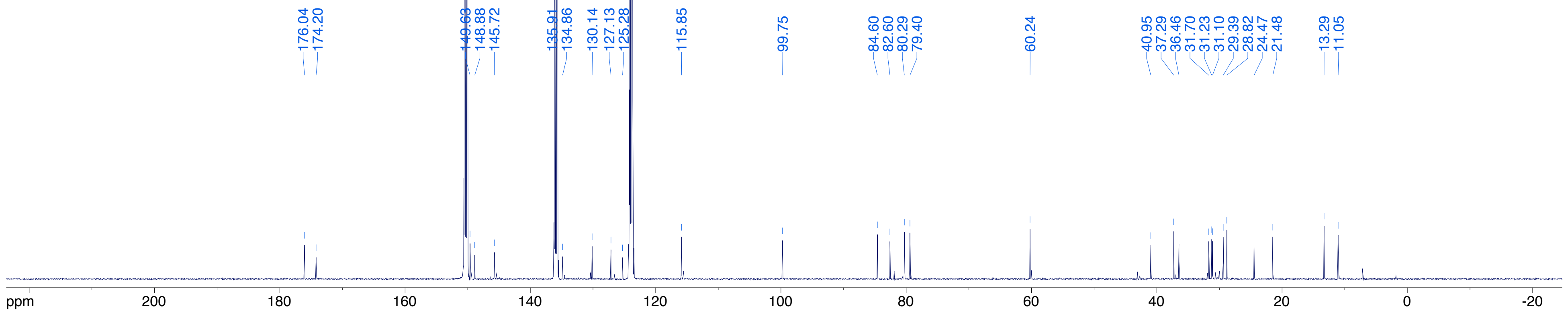

Supplement: Supplementary file 1 — miscellaneous_information [file anie0054-12618-sd1.pdf]
